# A high-throughput assay for screening natural products that boost NK cell-mediated killing of cancer cells

**DOI:** 10.1080/13880209.2020.1748661

**Published:** 2020-05-01

**Authors:** Zihang Xu, Xiaowen Zhu, Lin Su, Chunpu Zou, Xiao Chen, Yifei Hou, Chenyuan Gong, Wanyi Ng, Zhongya Ni, Lixin Wang, Xuewei Yan, Yangzhuangzhuang Zhu, Xiaoning Jiao, Chao Yao, Shiguo Zhu

**Affiliations:** a Laboratory of Integrative Medicine, School of Basic Medical Sciences, Shanghai University of Traditional Chinese Medicine, Shanghai, China; b Department of Immunology and Pathogenic Biology, School of Basic Medical Sciences, Shanghai University of Traditional Chinese Medicine, Shanghai, China

**Keywords:** Cytotoxicity, non-small cell lung cancer, 20-deoxyingenol 3-angelate, ingenol 3-angelate

## Abstract

**Context:**

Natural killer (NK) cells can eliminate malignant cells and play a vital role in immunosurveillance. Administration of natural compounds represents a promising approach for antitumor immunotherapy, which may enhance the NK cell activity via multiple mechanisms.

**Objective:**

Establishing approaches to evaluate the effect of select natural products on NK cell-mediated cytotoxicity.

**Materials and methods:**

We selected a natural product library containing 2880 pure compounds, which was provided by the National Centre for Drug Screening of China. 0.1% DMSO was employed as a negative control, and 100 U/mL human recombinant IL-2 was employed as a positive control. To evaluate the % of tumour cells which were killed by NK cells, expanded NK cells were co-cultured with tumour cells and then treated with natural products at the concentration of 10 μM. After 24-h co-incubation, luminescent signal was detected and percent lysis was calculated.

**Results:**

We report on the results of a three-round high-throughput screening effort that identified 20-deoxyingenol 3-angelate (DI3A) and its analogue ingenol 3-angelate (I3A) as immuno enhancers which boosts NK cell-mediated killing of non-small cell lung cancer cells (NSCLCs). Biophotonic cytotoxicity assay and calcein release assay were used as two well-established NK cell cytotoxicity detection assays to validate the immuno-enhancing effects of DI3A and I3A, which was achieved by increasing degranulation and interferon-gamma secretion of NK cells.

**Conclusions:**

Our newly established ATP-based method was a valuable and information-rich screening tool to investigate the biological effects of natural products on both NK cells and tumour cells.

## Introduction

Natural killer (NK) cells are an important component of the innate immune system, which can eliminate transformed cells and virally infected cells (Guillerey et al. 2016; Rezvani et al. 2017; Glasner et al. 2018). Recently, NK cells are considered to be the founding member of the innate lymphoid cell (ILC) family, as they are the predominant population endowed with cytolytic function. Functions of NK cells are regulated by a variety of activating and inhibitory receptors at the cell surface. The dynamic equilibrium between activating and inhibitory signals determines whether or not NK cells are activated to destroy target cells (Idorn and Hojman 2016; Bi and Tian 2019). The tumoricidal activity of NK cells is mediated by (a) release of a variety of cytokines (such as IFN-γ and TNF-α), (b) exocytosis of lytic granules containing granzymes a (Dotiwala et al. 2016) and (c) expression of some death receptor ligands (Pesce et al. 2017).

There have been innumerable attempts over years to use NK cell immunotherapy for the treatment of solid cancers without much success. NK cell-mediated targeting of solid tumour is usually not efficient, although the tumour cells express high amounts of activating ligands and low levels of inhibitory ligands (Grossenbacher et al. 2017). The escape of tumour cells from immunosuppressive cells leads to the failure of immunotherapy, especially for solid tumours in clinical settings. Therefore, it is urgently necessary to develop new drugs with the ability to block the immunosuppressive environment in order to efficiently prevent the escape of tumour cells from immunosuppressive cells and restore NK cell-mediated antitumor response (Ohs et al. 2017).

Natural products are a major source for the discovery and development of new drugs that can enhance NK cell antitumor activity. Recently, we have reported rocaglamide, a natural product extracted from *Aglaia elliptifolia* Merr. (Meliaceae), enhances NK cell-mediated killing of non-small cell lung cancer cells by inhibiting autophagy (Yao et al. 2018). However, the lack of simple and cost-efficient approaches for large-scale screening of the natural products has greatly impeded the development of novel drugs. Therefore, it is necessary to exploit a novel high-throughput assay for screening of novel and small molecules from a pool of thousands of natural products. A number of different assays are available for measuring NK cell cytotoxicity. Since 1968, the ***‘***gold standard***’*** assay for NK cell cytotoxicity has been the chromium 51 release assay (Fassy et al. 2017). However, the chromium release assay has many limitations, such as, hazardous radioactivity, high cost, short half-life, increased staff requirements for radiation safety training and licencing, and disposal of radioactive waste. Other established methods to assess NK cell cytotoxicity, such as CD107α degranulation assay (Gamerith et al. 2017; Zhang et al. 2017), ELISA detecting interferon (IFN)-γ secreted from NK cells (Gong et al. 2017), and intracellular staining of IFN-γ produced in NK cells, are also not available for high-throughput screening due to their requirements of complex and time-consuming operations.

**Figure 1.**
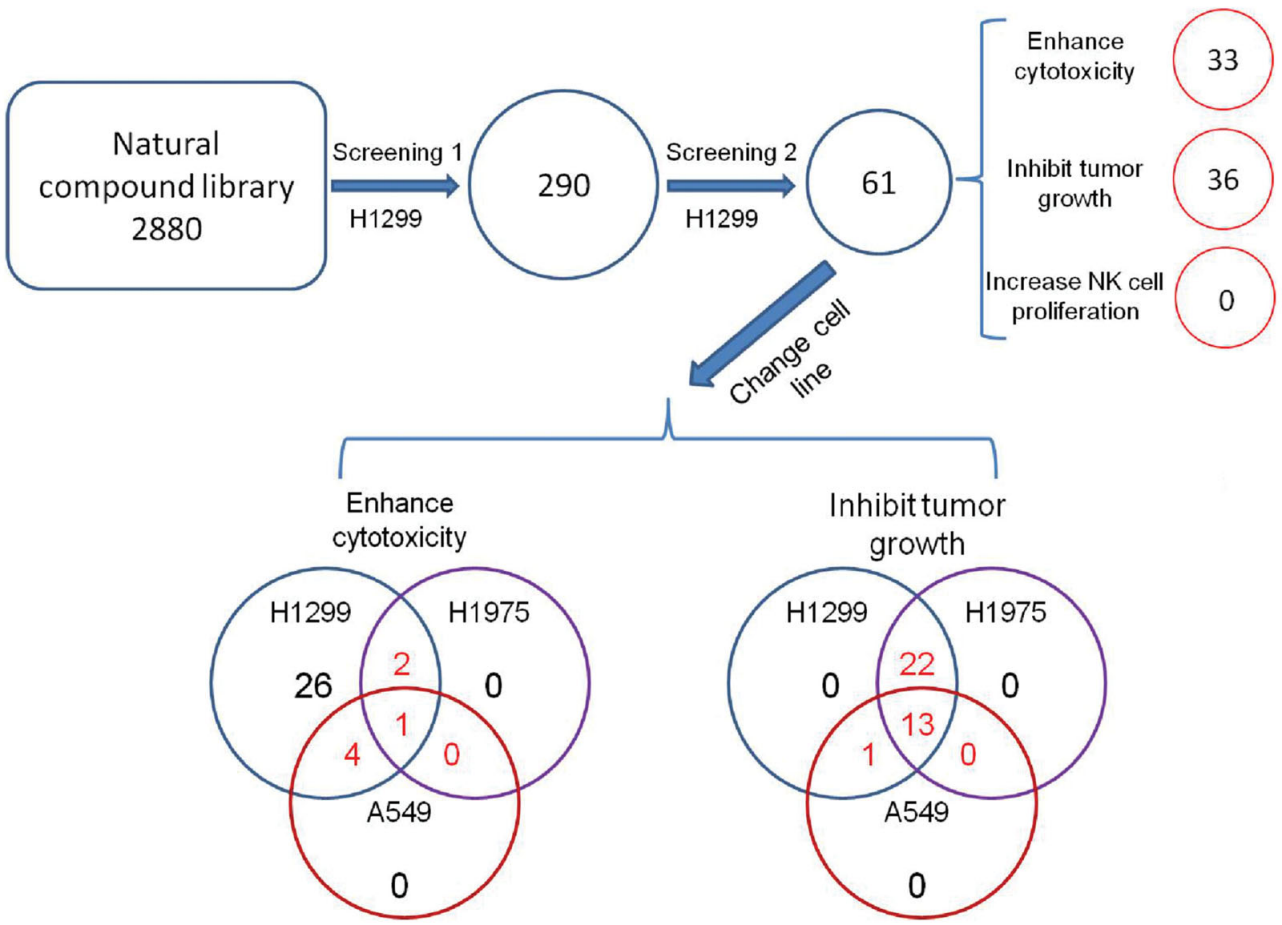
Schematic of the screen design and protocol.

In the present study, we developed a luminescence-based assay, which could concurrently assess NK cell-mediated cytotoxicity, NK cell viability and tumour cell viability. The cytotoxicity of NK cells was evaluated through the quantitation of ATP, which signals the proportion of metabolically active cells. We selected a natural product library containing 2880 natural products provided by The National Centre for Drug Screening (Shanghai, China) (http://www.screen.org.cn/index.shtml). Details of the tested natural products are listed in Supplementary Table 1. We found that 406 natural products could increase the cytotoxicity of NK cells by more than 20%, and 222 natural products could increase the proliferation of NK cells by more than 20%. Moreover, we also found that 42 natural products directly suppressed the proliferation of H1299 cells by more than 50%. Our test results are consistent with the report (Figure 1) (Zhang et al. 1999; Yadav et al. 2005; Lu and Chen 2010). Collectively, our findings demonstrated that such high-throughput screening could be used to find agents with potent effects on NK cell-mediated immunotherapy.

## Materials and methods

### Reagents

20-Deoxyingenol 3-angelate (DI3A) was purchased from BioBioPha (Yunnan, China). Ingenol 3-angelate (I3A) was obtained from AdipoGen (San Diego, CA, USA). Calcein-AM was supplied by Sigma-Aldrich (St. Louis, MO, USA). Recombinant human IL-2 protein was provided by PeproTech (Rehovot, Israel). PE anti-human CD56, FITC anti-human CD107α, APC anti-human IFN-γ, and murine isotype controls (IgG1κ-PE, IgG1κ-FITC and IgG1κ–APC) were purchased from BioLegend Inc. (San Diego, CA, USA). CellTiter-Glo Luminescent Cell Viability Assay Kit was obtained from Promega (Madison, WI, USA). Fixation/Permeabilization Solution Kit with BD GolgiStop™ was provided by BD Biosciences (CA, USA).

### NK cell expansion

**Figure 2.**
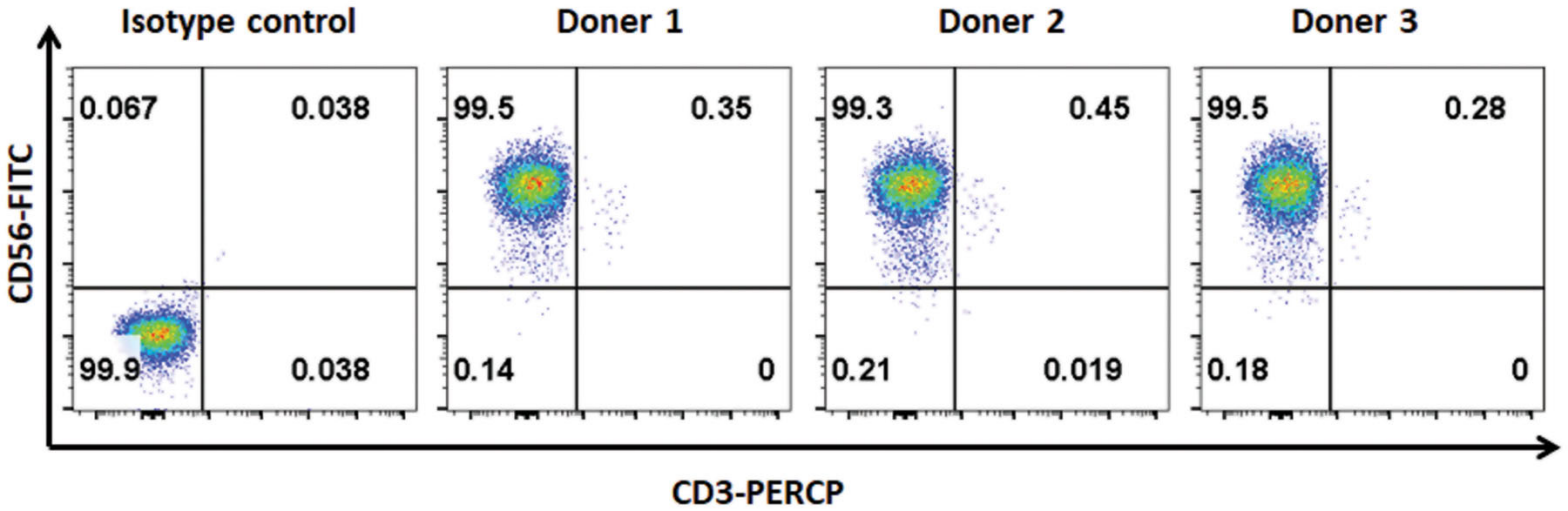
The purity of NK cells. To expand NK cells, fresh or frozen PBMCs from healthy donors were co-incubated with irradiated mbIL-21-CD137L-K562 cells for 2 weeks in RPMI-1640 complete medium at 37 °C in a humidified atmosphere containing 5% CO_2_. The purity of NK cells was tested by flow cytometry.

Human peripheral blood mononuclear cells (PBMCs) were obtained from the Shanghai Blood Centre under a research protocol approved by the Department of Shanghai Blood Administration. PBMCs were either freshly used or frozen in foetal bovine serum (FBS, Gibco) containing 10% DMSO. Frozen PBMCs were thawed and maintained in RPMI-1640 medium supplemented with 10% FCS, 1% penicillin–streptomycin, 2 mM L-glutamine and 200 U/mL IL-2 at 37 °C in a humidified atmosphere containing 5% CO_2_. NK cells were expanded as previously described (Wang et al. 2013; Yao et al. 2018). Briefly, fresh or frozen PBMCs were co-incubated with irradiated mbIL-21-CD137L-K562 cells for 2 weeks in RPMI-1640 complete medium at 37 °C in a humidified atmosphere containing 5% CO_2_. The flow cytometry for CD56^+^/CD3^−^ NK cells showed that the NK cell purity was above 99% (Figure 2).

### Luminescent cell viability assay (screening assay)

Chemical library containing 2880 natural products was obtained from China National Compound Resource Centre (NCRC). Cells were seeded into white 384-well opaque-walled plates (Corning, NY, USA), with 25 μL of culture medium per well containing 1000 NK cells, H1299 cells or the mixture of NK cells and H1299 cells. Then, cells were treated with natural products at a final concentration of 10 μM. Each natural product was tested in triplicate. In addition, 0.1% DMSO was employed as a negative control, and 200 U/mL IL-2 was employed as a positive control. CellTiter-Glo Reagent (Promega) was added after 24-h incubation. Luminescent signal was determined using Synergy 2 Multi-Mode Microplate Reader (BioTek Instruments, Inc., Winooski, VT, USA).

### Calculation of percent lysis

Percent viability was calculated as the mean luminescent signal of mixture of NK cells and H1299 cells (Mean_MIX_) minus that of NK cells (Mean_NK_) divided by the mean signal of H1299 cells (Mean_H1299_). % lysis was calculated as follows:

$$\%\;\text{lysis}=1-\frac{\left({\text{Mean}}_{\text{MIX}}-{\text{Mean}}_{\text{NK}}\right)}{{\text{Mean}}_{H1299}}\times 100\%$$

### Biophotonic cytotoxicity assay

Firefly luciferase-transfected H1299 (H1299-fLuc+) cells were seeded into 96-well opaque-walled plates (Corning, NY, USA) with 100 μL of culture medium per well. Target cells were then co-cultured with NK cells at the E:T ratio of 1:1 and treated with natural products in a final volume of 200 μL. After 4-h incubation, a final concentration of 0.14 mg/mL luciferin was added into each well. The plate was adapted in the dark for 10 min at 37 °C, and luminescent signal was determined from 8 mm above the bottom of the plate using Synergy 2 Multi-Mode Microplate Reader.

### Calcein release assay

Tumour cells were incubated with 2 μg/mL calcein-AM at 37 °C for 1 h with occasional shaking. NK cells and target cells were mixed at the E:T ratio of 1:1 (1 × 104:1 × 104) and co-cultured in 96-well U-bottom plates. After 4-h incubation, 100 μL of supernatant was transferred to a new 96-well flat-bottom plate. Fluorescence signal was determined using a Synergy 2 Multi-Mode Microplate Reader (BioTek Instruments, Inc., Winooski, VT) (excitation filter of 485 nm, emission filter of 538 nm). Percent lysis was calculated according to the formula [(experimental release − spontaneous release)/(maximum release −  spontaneous release)] × 100%.

### Flow cytometry analysis

Intracellular staining was performed using BD Cytofix/CytopermTM Fixation/Permeablization Kit (BD Biosciences) according to the manufacturer’s instructions. Briefly, NK cells were co-cultured with H1299 cells in 48-well plates. Then, FITC anti-human CD107α antibody and monensin were added into each well. After 4-h incubation, cells were harvested and incubated with PE anti-human CD56 antibody at 4 °C for 30 min in the dark. After brief washing with staining buffer (1% FBS in 1× PBS), cells were fixed and permeabilized using fixation/permeabilization solution at 4 °C for 20 min in the dark. Subsequently, cells were stained by APC anti-human IFN-γ antibody at 4 °C for 30 min in the dark. After washing two times, cells were resuspended in staining buffer. Data were acquired using BD Accuri C6 (BD Biosciences) and analysed using FlowJo software (Ashland, OR).

### Statistical analysis

Statistical comparison was performed by one-way ANOVA. Multiple comparison was performed using LSD *post-hoc* test. *p* < 0.05 was considered as statistically significant.

## Results

### Development of high-throughput screening assays

**Figure 3.**
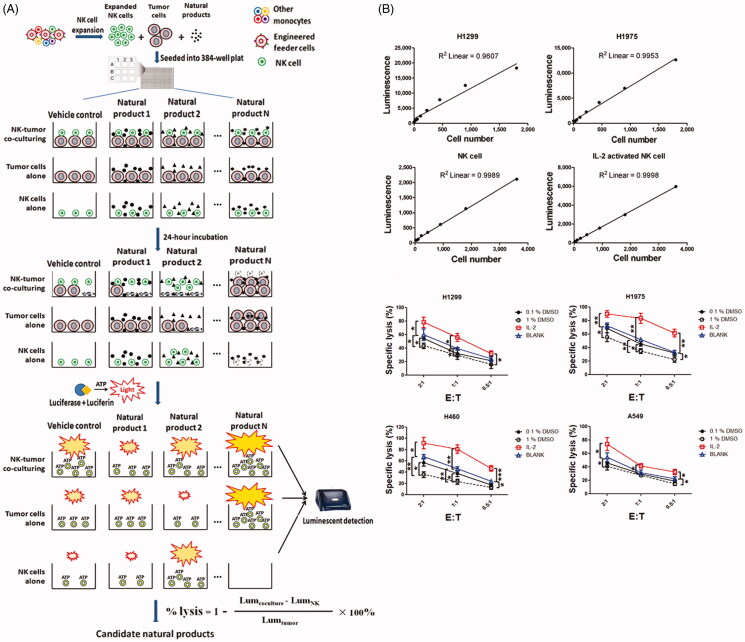
The schematic presentation of luminescent cell viability assay for screening of natural products and the sensitivity analysis of high-throughput screening system. (A) To identify potent natural products, expanded human NK cells were co-cultured with H1299 cells in 384-well plates in the presence or absence of natural products. Luminescent signal was detected after 24 h co-culturing. (B) The correlation between cell number and luminescent signal intensity was examined using linear regression analysis. (C) NK cells were co-cultured with H1299, H1975, A459 or H460 cells at the indicated effector to target (E: T) ratio and then treated them with 100 U IL-2, 0.1% DMSO or 1% DMSO. Untreated NK cells (blank) were regarded as control. Lysis of tumour cells was detected after 24 h. Data shown represent mean ± SD.

In the present study, we designed a luminescence-based cell viability assay to identify natural products that could enhance the tumoricidal activity of NK cells (Figure 3(A)). Expanded NK cells were co-cultured with tumour cells and then treated with candidate compounds. After 24-h co-incubation, luciferase and its substrate were added into the culture medium. Then, bioluminescence was generated during the catalytic process of luciferase, which requires O_2_ and ATP (Lundin 2000; Chen et al. 2017). Lysis of tumour cells was detected through the quantitation of ATP, which signals the proportion of metabolically active cells.

We first analysed the sensitivity of this high-throughput screening assay. Linear regression analysis revealed that the number of living cells was highly correlated with intensity of luminescent signal, and as few as 28 cells could be detected above background (Figure 3(B)). Moreover, using this ATP detection method, we could also discriminate between two different statuses of NK cells. Signal intensity of IL-2-activated NK cells was almost twice compared with the resting NK cells. To validate whether ATP could be used as a sensor to determine NK cell-mediated cytotoxicity, we evaluated the lysis of tumour cells by NK cells treated with either IL-2 or DMSO (Figure 3(C)). As expected, IL-2-activated NK cells showed enhanced cytotoxicity, while 0.1% DMSO-treated NK cells showed impaired cytotoxicity compare with the untreated NK cells. Our results demonstrated that this screening system could distinguish IL-2-activated NK cells from DMSO-challenged NK cells by measuring their antitumour activity.

### High-throughput screening of natural products for induction of NK cell tumoricidal activity

**Figure 4.**
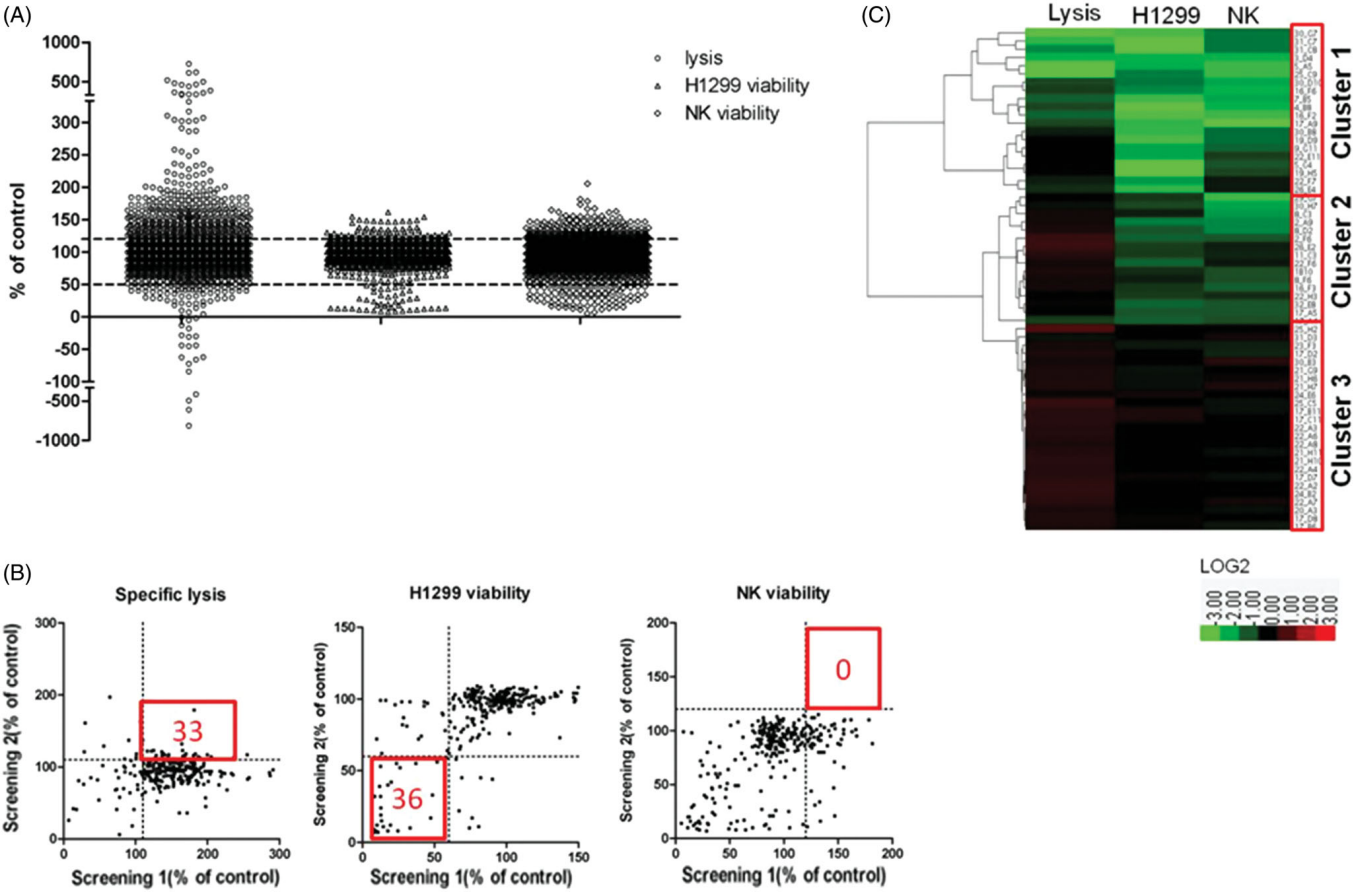
High-throughput screening of natural products. (A) A scatter plot indicates the percent lysis, H1299 cell viability and NK cell viability of each tested natural product. Dotted lines represent the cut-off ratio (120% for up-regulation and 50% for down-regulation). (B) XY-Scatter plots compare the factors from both replicate screens. (C) Hierarchical clustering of the three factors of each candidate. Clustering is based on Ward’s linkage criteria and the half Euclidean distance metric.

Through a high-throughput luminescence-based cell viability assay, we screened a chemical library consisting of 2880 natural products to identify compounds capable of enhancing antitumour activity of NK cells. In the primary screening, NK cells and H1299 cells were treated with compounds at 10 µM in 0.1% DMSO, and 200 U/mL IL-2 was employed as a positive control, which was able to enhance the NK cell cytotoxicity. Three factors of each natural product were evaluated, including lysis of tumour cells, tumour cell viability and NK cell viability (Figure 4(A)). Natural products that enhanced lysis or proliferation of NK cells by more than 20% were considered as candidates, and those capable of suppressing H1299 cell proliferation by greater than 50% were also regarded as candidates. We found that 406 (14.1%) compounds increased the cytotoxicity of NK cells by more than 20%, and 222 (7.7%) compounds increased the proliferation of NK cells by more than 20%. We also found that 42 (1.5%) compounds directly suppressed the proliferation of H1299 cells by more than 50%.

According to the variation of the experimental triplicates, we chose 290 compounds as candidates. These natural products were then tested in the confirmatory screening in the same manner as the primary screening. As a result, we retrieved 61 compounds from the confirmatory screening based on the same cut off. We found that 33 compounds increased the cytotoxicity of NK cells, and 36 compounds suppressed the proliferation of tumour cells (Figure 4(B)). For the above-mentioned factors, these 61 compounds were clustered using unsupervised hierarchical clustering (Figure 4(C)). Briefly, we divided them into three primary clusters. Natural products in cluster 1 showed great toxicity against both malignant and non-malignant cells, and they also decreased NK cell-mediated cytotoxicity. These natural products were not recommended to be developed into anticancer drugs because they might promote immune escape of tumour cells. Natural products in cluster 2 also showed toxicity against tumour and NK cells. However, these products were able to increase the cytotoxicity of NK cells. Natural products in cluster 3 showed promising potential in immune therapy because they enhanced the tumoricidal activity of NK cells without significant toxicity against NK cells.

### DI3A enhances NK cell cytotoxicity

Cancer cells often carry alterations in uncharacterised genes, leading to different responses to chemical stimuli. Therefore, we examined whether our candidates retrieved from confirmatory screening could also increase the cytotoxicity of NK cells against the other two NSCLC cell lines, A549 and H1975 cells (Figure 1). We found that only one natural product extracted from *Euphorbia peplus* L. (Euphorbiaceae), named 20-deoxyingenol 3-angelate (DI3A), enhanced the cytotoxicity of NK cells against all the three NSCLC cell lines.

**Figure 5.**
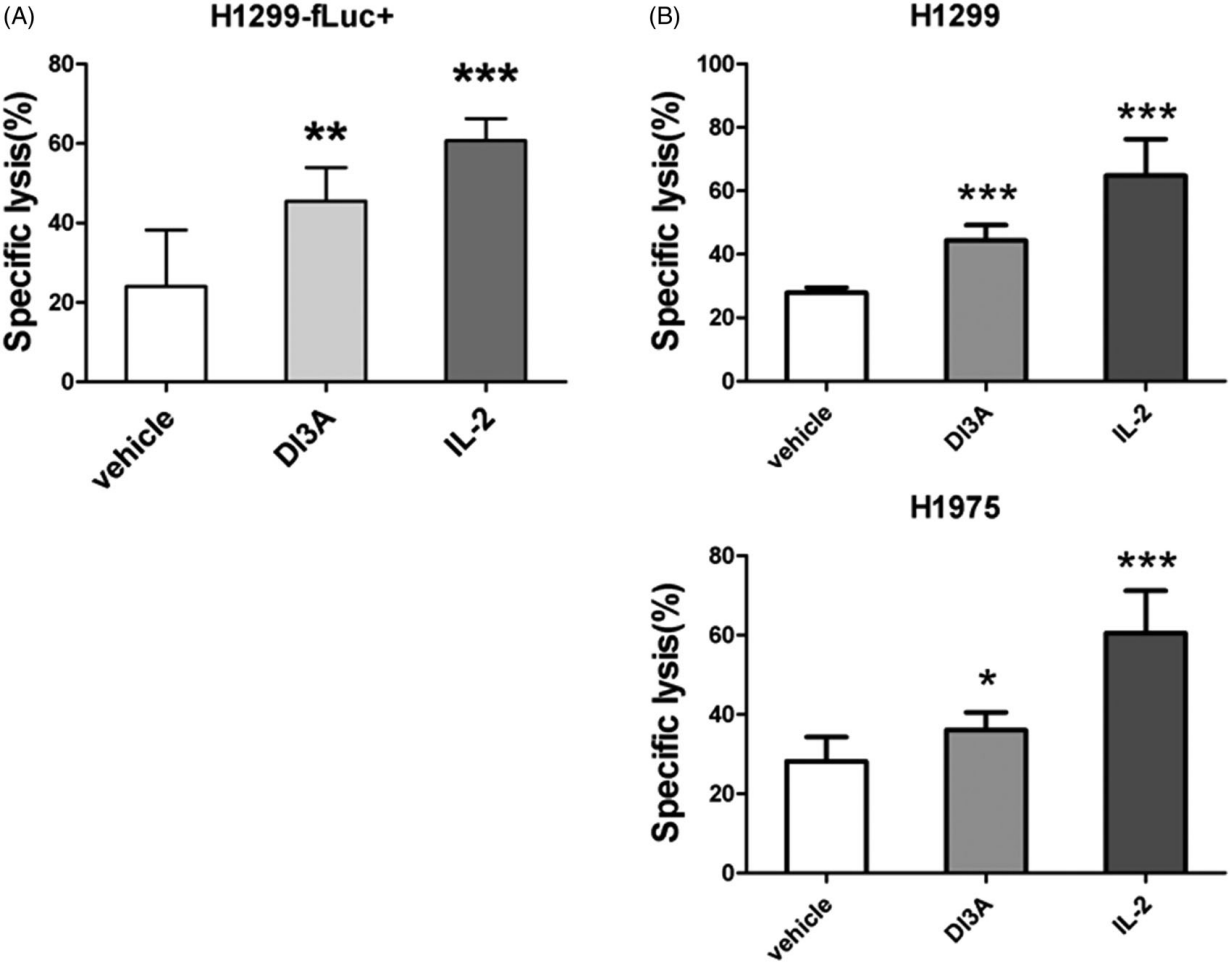
Confirmation of the cytotoxicity enhancing activity of DI3A. (A) H1299-fLuc + cells were co-cultured with NK cells in the presence or absence of 10 nM DI3A at the effector to target (E: T) ratio of 1:1. Lysis was assessed using biophotonic cytotoxicity assay after 24 h incubation. (B) H1299 or H1975 cells were co-cultured with NK cells in the presence or absence of 10 nM DI3A at the effector to target (E: T) ratio of 1:1. Lysis was assessed using calcein release assay after 4 h incubation. 100 U of IL-2 was employed as a positive control. Data shown represent means ± SD, *p < 0.05; **p < 0.01; ***p < 0.001.

To confirm the effect of DI3A on the tumoricidal activity of NK cells, we employed the other two detection systems to determine lysis of tumour cells, which are biophotonic cytotoxicity assay (Brown et al. 2005) and calcein release assay (Jang et al. 2012). NK cell-mediated cytotoxicity evaluated by biophotonic cytotoxicity assay showed that DI3A significantly (*p* = 0.002) increased the lysis of H1299-fLuc + cells (45.5 ± 8.5%) compare with the vehicle control (24.0 ± 14.2%) (Figure 5(A)). In the other detection system, DI3A significantly (*p* < 0.001) increased the lysis of H1299 cells (44.3 ± 4.8%) compared with the vehicle control (27.8 ± 1.7%) (Figure 5(B)). Similar results were observed in H1975 cells, DI3A also increased the cytotoxicity of NK cells against H1975 cells (36.0 ± 4.3% vs. 28.0 ± 6.2%, *p* = 0.012). Our results demonstrated that DI3A indeed enhanced NK cell tumoricidal activity. Therefore, we focussed on this natural product in our subsequent study.

### DI3A and I3A enhance the tumoricidal activity of NK cells in a dose-dependent manner

**Figure 6.**
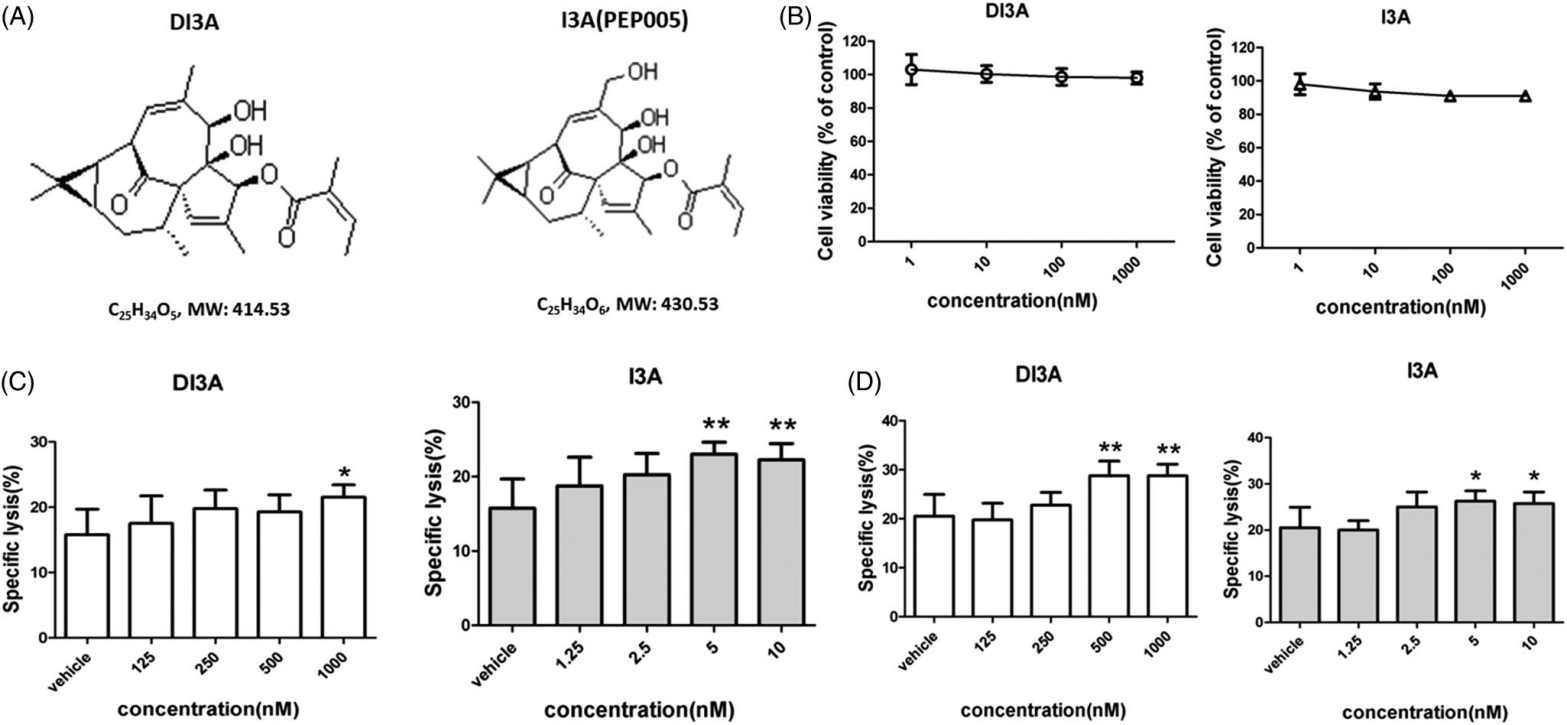
Cytotoxicity enhancing effects of DI3A and I3A on NK cells. (A) Chemical structures of DI3A and I3A. (B) H1299 cells were treated with DI3A or I3A at the indicated concentrations for 24 h. Cell viability was assessed by luminescent cell viability assay. (C) H1299 cells or (D) H1975 cells were co-cultured with NK cells at the E: T ratio of 1:1. Then cells were treated with DI3A or I3A at the indicated concentrations. Lysis was assessed using luminescent cell viability assay after 24 h incubation. Data shown represent means ± SD, *p < 0.05; **p < 0.01.

When we investigated the chemical structure of DI3A, we found that ingenol 3-angelate (I3A, also known as PEP005) was an analogue of DI3A (Figure 6(A)). I3A is known as a novel anticancer agent that activates protein kinase C (PKC), resulting in anti-proliferative and pro-apoptotic effects in several human cancer cell lines (Kedei et al. 2004). I3A also exerts immunomodulatory effects on both malignant cells and immune cells (Wang and Liu 2018). However, the effect of I3A on NK cells remains largely unexplored. Therefore, we aimed to investigate the effects of DI3A and I3A on NK cells. High concentration (micromolar) of I3A induces cell death in human breast and prostate cancer cells (Wang and Liu 2018). To exclude the direct killing of tumour cells by DI3A and I3A, we treated H1299 cells at lower concentrations (Figure 6(B)). Our results showed that both natural products rarely suppressed the proliferation of tumour cells at the nanomolar scale.

**Figure 7.**
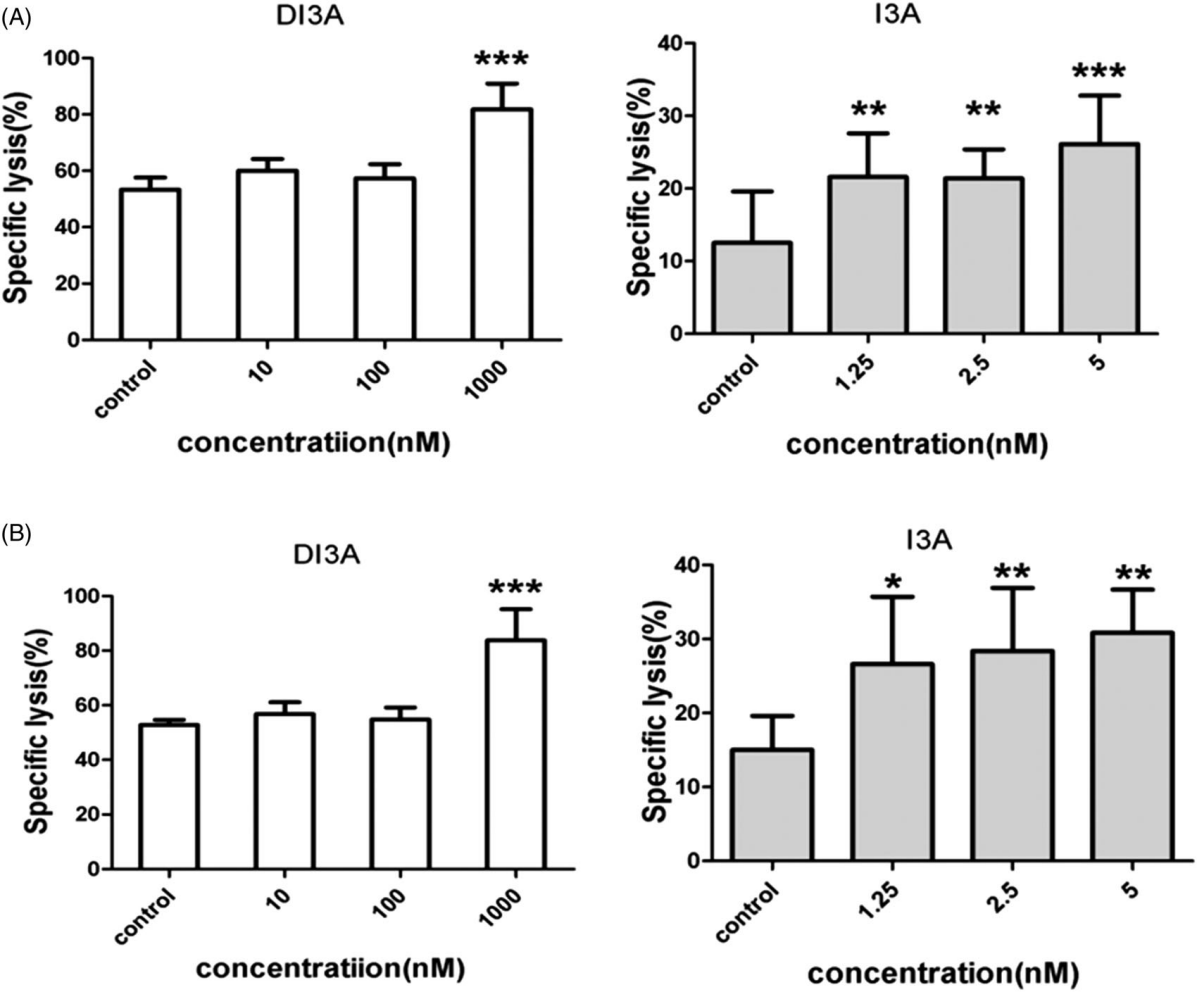
Cytotoxicity enhancing effects of DI3A and I3A on NK cells. (A) H1299 cells or (B) H1975 cells were co-cultured with NK cells at the E: T ratio of 1:1. Then cells were treated with DI3A or I3A at the indicated concentrations. Lysis was assessed using calcein release assay after 4 h incubation. Data shown represent means ± SD, *p < 0.05; **p < 0.01.

Because DI3A and I3A share a similar chemical structure, we hypothesized that I3A also increased the tumoricidal activity of NK cells. We co-cultured NK cells with target cells (H1299, H1975, A549 or H460 cells) and then treated them with DI3A or I3A. Lysis of tumour cells was detected after 24 h using our screening system. As shown in Figure 6(C and D), both natural products enhanced the tumoricidal activity of NK cells in a dose-dependent manner. Treatment of 1,000 nM DI3A increased the cytotoxicity of NK cells by 36.5% against H1299 cells (21.5 ± 1.9% vs. 15.8 ± 3.9%, *p* = 0.024) and 40.2% against H1975 cells (28.8 ± 2.4% vs. 20.5 ± 4.4%, *p* = 0.003). However, treatment of 5 nM I3A enhanced the cytotoxicity of NK cells by 46.0% against H1299 cells (23.0 ± 1.6% vs. 15.8 ± 3.9%, *p* = 0.004) and 28.0% against H1975 cells (26.3 ± 2.2% vs. 20.5 ± 4.4%, *p* = 0.017). Similar results were observed in calcein release assay (Figure 7(A,B)). These results demonstrated that both DI3A and I3A exerted powerful enhancing effects on the tumoricidal activity of NK cells even at nanomolar concentrations. Moreover, the comparison of biological activity between DI3A and I3A suggested that I3A was more reactive than DI3A due to its active hydroxyl at position 20.

### DI3A and I3A enhance the cytotoxicity of NK cells through increasing degranulation and IFN-γ secretion

**Figure 8.**
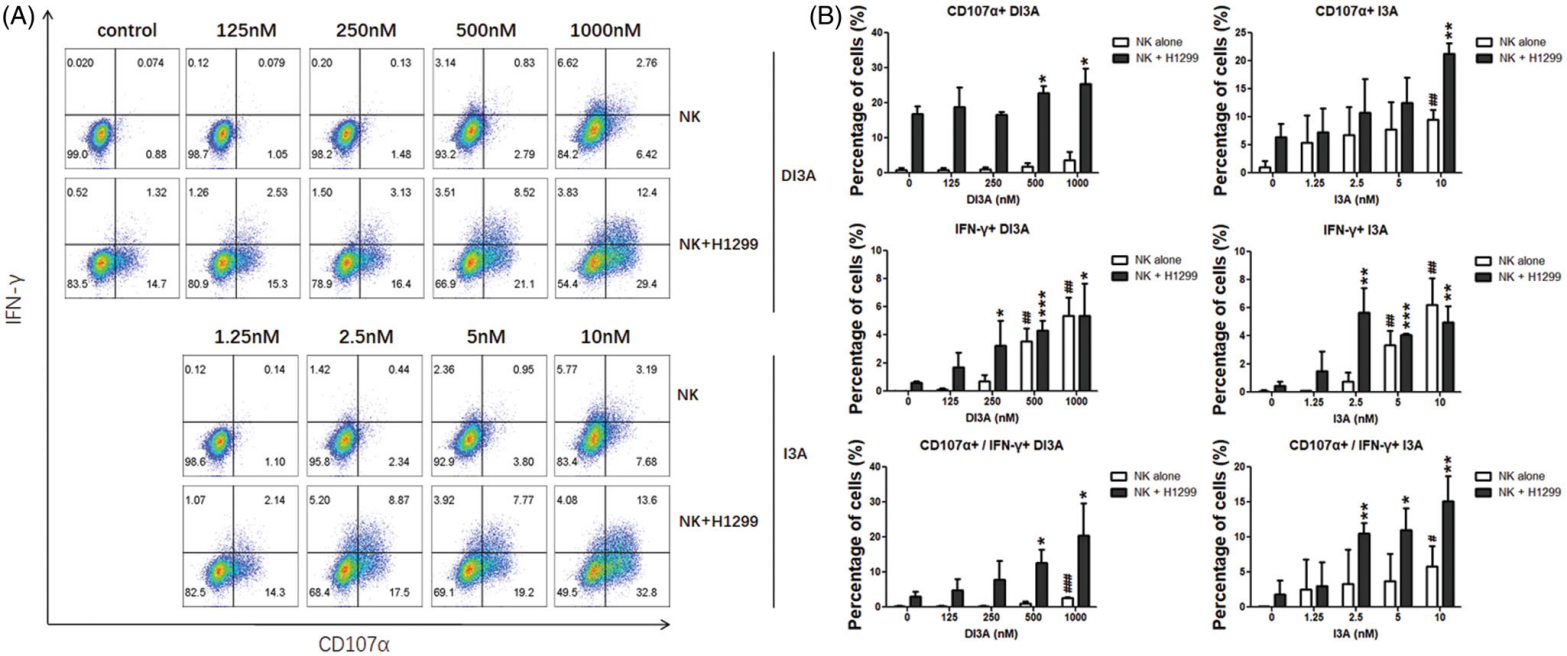
Role of DI3A andI3A in enhancing NK cells tumoricidal activity. (A,B) NK cells were treated with DI3A or I3A at the indicated concentrations, either being cultured alone or encountered with H1299 cells. CD107α expression and IFN-γ production by NK cells was measured after 4 h incubation using flow cytometry, and the analysis was applied by FlowJo software. NK cells were gated on CD56^+^ subpopulation. Data shown represent means ± SD, *p < 0.05; **p < 0.01; ***p < 0.001; ##, non-statistical significance, one-way ANOVA with Tukey’s multiple comparisons test.

We next investigated the underlying mechanism of DI3A and I3A in induction of NK cell cytotoxicity. Since NK cells destroy target cells mainly through degranulation of cytolytic granules and IFN-γ production, we evaluated the expression of CD107α (a marker of degranulation) (Zhao et al. 2015; Gamerith et al. 2017) and the production of IFN-γ by NK cells after DI3A and I3A treatment (Figure 8). The results showed that the CD107α expression and intracellular IFN-γ production were enhanced in a dose-dependent manner. The expression of CD107α and IFN-γ by NK cells treated with 1000 nM DI3A were 2.6- and 8.8-fold higher compared with the vehicle control, respectively. In addition, the expression of CD107α and IFN-γ by NK cells treated with 10 nM I3A were 2.9- and 9.6-fold higher compared with the vehicle control, respectively. Moreover, at the relatively lower concentrations (250 nM for DI3A and 2.5 nM for I3A), these two natural products increased the expression of CD107α and IFN-γ only when NK cells were co-cultured with target cells, suggesting that DI3A and I3A properly activated NK cells without unnecessary cytokine storms. Taken together, these results demonstrated that DI3A and I3A regulated NK cell-mediated cytotoxicity through enhancing degranulation of cytolytic granules and production of IFN-γ.

## Discussion

The currently available detection methods of NK cell activity can be mainly divided into two categories (Brown et al. 2005; Jang et al. 2012; Elsaid et al. 2018), evaluating the proportion of lysed target cells directly or detecting the alterations in NK-cell effector molecules to reflect the killing activity indirectly. The former category includes ^51^Cr release assay, calcein release assay, lactate dehydrogenase (LDH) assay and biophotonic cytotoxicity assay. The latter category mainly includes flow cytometry to detect CD107α expression, IFN-γ intracellular staining or ELISA to detect IFN-γ secretion. However, none of these methods can be applied to large-scale screening of natural products that enhance the antitumor effects of NK cells.

In this study, we established a high-throughput screening system based on the principle of bioluminescence. ATP is required for generating luminescence when luciferase catalyses the oxidation reaction of its substrate, and the rate of enzymatic reaction is proportional to the content of ATP in the system. Besides, ATP is the product of oxidative phosphorylation of living cells (Nath and Villadsen 2015). Therefore, the relative proportion of living cells in the system can be estimated by measuring the bioluminescence intensity. Using this high-throughput screening system, we screened 2880 natural products in a short period of time. We found that 406 natural products enhanced the antitumor activity of NK cells by more than 20%, and 222 natural products promoted the proliferation of NK cells by more than 20%. Furthermore, 42 natural products inhibited the proliferation of tumour cells by more than 50%. The effects of each natural product on NK cell-mediated antitumor activity, tumour cell proliferation and NK cell proliferation were analysed by cluster analysis.

There are several advantages in the high-throughput screening method based on ATP detection established in this study. First, in this system, neither target cells nor effector cells required additional labelling. And calculation of percent lysis of target cells just required some simply co-culturing procedures. Moreover, since target cells and effector cells do not require genetic engineering, researchers can arbitrarily replace the type of target cells and effector cells, making this ATP-based detection system extendable to other applications, such as detection of T cell-mediated antitumour effects. Second, this system possessed very high sensitivity and resolution. Only 28 tumour cells were required to produce a luminescent signal, which was significantly higher than the background. The screening system also had a broad linear range. Cell number is correlated with luminescent signal within the range of 28–1800 cells. High sensitivity and wide linear range resulted in high resolution of the system. Using this screening system, we could clearly distinguish the effects of 0.1% DMSO and IL-2 on the killing activity of NK cells. As was expected, we confirmed that 0.1% DMSO could inhibit NK cell-mediated killing of H1299 and H1975 in an E:T ratio-dependent manner using this luminescence-based assay. Therefore, even if the natural product has only a weak regulatory effect on the antitumour activity of NK cells, our system could clearly distinguish it. IL-2 is a well-known cytokine that activates NK cells, which can promote NK cell proliferation, degranulation and IFN-γ secretion. Therefore, in our system, IL-2 was used as a positive control, and this system verified that the antitumour effect of NK cells could be significantly enhanced by IL-2 once again. Therefore, the system was a simple, sensitive and reliable high-throughput screening system. Third, this screening assay is an information-rich system. Through this system, we could simultaneously investigate the antitumor effect of natural products on NK cells, the regulation of tumour cell proliferation and the proliferation of NK cells. In addition, unlike the indirect detection methods, such as CD107a and IFN-γ assay, our newly developed system directly detected the percent lysis of target cells, and it could more intuitively investigate the antitumour effect of NK cells. We also tested other screening systems (such as ELISA for IFN-γ and flow cytometry for MICA/B, HLA-ABC expression). These systems could also screen many candidate natural products. However, when we tried to validate the immune enhancing effects of the candidates, we are often disappointed by the negative results. This could be attributed to the immune escape characteristics of tumour cells. Sometimes, natural products do activate NK cells, while the immune escape of tumour cells is also simultaneously enhanced. Therefore, the product does not increase the percent lysis of tumour cells. For example, IFN-γ released by NK cells can up-regulate the expression of HLA-ABC in tumour cells, which reduces the activating signalling of NK cells. IFN-γ can also up-regulate PD-L1 expression, leading to exhaustion of PD-1^+^ NK cells. Therefore, directly examining the percent lysis of target cells can intuitively examine the final effect of antitumor effects of NK cells, which enables researchers to discover natural products that enhance the antitumor effect of NK cells by blocking immune escape.

Although the aforementioned advantages make this luminescence-based assay of great promise for application, there are still drawbacks within this high-throughput system. One critical point is that the luminescence-based system lacks imaging record procedures to allow researchers to inspect how the cells behave after treating with natural products visually. Recently, the development of high-throughput imaging flow cytometers provides an opportunity to record cellular behaviour and fluorescent signal simultaneously (Stavrakis et al. 2019). The imaging-based flow cytometry offers significant advantages since cellular behaviour analysis plays a critical role in the assessment of target cell lysis by immune cells, and fluorescent dye-conjugated antibodies offer a flexible scheme for multi-parameter analysis. Recent study has shown that high-throughput target cell visualisation assay operates with the same sensitivity as standard chromium release assays, indicating the reliance of high-throughput imaging assay (Welter et al. 2018). Thus, researchers can label immune effector cells and tumour cells with proper fluorescent dye or antibodies to visually investigate how natural products affect target cell killing, effector-target cell conjugation, and immune effective molecular production, such as CD56 antibody for human NK cells, carboxyfluorescein succinimidyl ester (CFSE) for tumour cells, propidium iodide (PI) for dead cells, and interferon antibody for immune-effective molecules. However, high-throughput imaging flow cytometry is still challenged by the costly, mechanically complex and large sample consuming characteristics. Combining high-throughput luminescent assay with high-throughput imaging flow cytometry may provide a cost saving and information-rich strategy, and leave data audit trails in the form of quality-controlled images, thus meeting requirements for screening natural products which may enhance NK cell-mediated killing of cancer cells.

## Supplementary Material

Supplemental Material
