# Supplementary material for: A high-throughput assay for screening natural products that boost NK cell-mediated killing of cancer cells
Source: Pharm Biol. 2020 May 1;58(1):357–66. doi: 10.1080/13880209.2020.1748661 (PMC7241510; doi:10.1080/13880209.2020.1748661)
Supplement: Supplemental Material [file IPHB_A_1748661_SM0978.pdf]

Library #	Cat. #	CAS#	Name	Locatio	M.W.	Conc.	Solvent
NCRC23393	BBP00001	509-15-9	Gelsemine	1-A2	322.401	10(μ)M	DMSO
NCRC23394	BBP00002	1358-76-5	Koumine	1-A3	306.401	10(μ)M	DMSO
NCRC23395	BBP00003	82354-38-9	Humantenmine	1-A4	326.39	10(μ)M	DMSO
NCRC23396	BBP00004	483-04-5	Ajmalicine	1-A5	352.427	10(μ)M	DMSO
NCRC23397	BBP00005	84847-50-7	Vasicinolone	1-A6	218.209	10(μ)M	DMSO
NCRC23398	BBP00006	1016983-51-9	Sulfocostunolide A	1-A7	312.381	10(μ)M	DMSO
NCRC23399	BBP00007	1059671-65-6	Sulfocostunolide B	1-A8	312.381	10(μ)M	DMSO
NCRC23400	BBP00008	1748-81-8	Carabrone	1-A9	248.318	10(μ)M	DMSO
NCRC23401	BBP00009	68832-39-3	4-Epi-isoinuviscolide	1-A10	248.318	10(μ)M	DMSO
NCRC23402	BBP00010	325686-49-5	Ascleposide E	1-A11	388.453	10(μ)M	DMSO
NCRC23403	BBP00011	82375-29-9	Humantenine	1-B2	354.443	10(μ)M	DMSO
NCRC23404	BBP00012	113973-31-2	(Z)-Akuammidine	1-B3	352.427	10(μ)M	DMSO
NCRC23405	BBP00013	72715-02-7	Ilicol	1-B4	238.366	10(μ)M	DMSO
NCRC23406	BBP00014	4290-13-5	Santamarine	1-B5	248.318	10(μ)M	DMSO
NCRC23407	BBP00015	1139-30-6	Caryophyllene oxide	1-B6	220.35	10(μ)M	DMSO
NCRC23408	BBP00016	553-21-9	Costunolide	1-B7	232.318	10(μ)M	DMSO
NCRC23409	BBP00017	477-43-0	Dehydrocostus lactone	1-B8	230.302	10(μ)M	DMSO
NCRC23410	BBP00018	6159-55-3	Vasicine	1-B9	188.226	10(μ)M	DMSO
NCRC23411	BBP00019	87441-73-4	11(13)-Dehydroivaxillin	1-B10	264.317	10(μ)M	DMSO
NCRC23412	BBP00020	57458-57-8	Stigmast-4-ene-3,6-dione	1-B11	426.674	10(μ)M	DMSO
NCRC23413	BBP00021	22149-69-5	Stigmastane-3,6-dione	1-C2	428.69	10(μ)M	DMSO
NCRC23414	BBP00022	295803-03-1	Gynuramide II	1-C3	682.112	10(μ)M	DMSO
NCRC23415	BBP00023	2182-14-1	Vindoline	1-C4	456.531	10(μ)M	DMSO
NCRC23416	BBP00024	115404-57-4	Niloticin	1-C5	456.7	10(μ)M	DMSO
NCRC23417	BBP00025	355143-38-3	6'-O-p-Hydroxybenzoylcatalposide	1-C6	602.54	10(μ)M	DMSO
NCRC23418	BBP00026	1016987-87-3	6-O-p-Hydroxybenzoylaucubin	1-C7	466.435	10(μ)M	DMSO
NCRC23419	BBP00027	1615-94-7	Simiarenol	1-C8	426.717	10(μ)M	DMSO
NCRC23420	BBP00028	3122-88-1	Eucalyptin	1-C9	326.343	10(μ)M	DMSO
NCRC23421	BBP00165	177602-14-1	Glycerol 1-(26-hydroxyhexacosanoate)	1-C10	486.768	10(μ)M	DMSO
NCRC23422	BBP00030	142698-60-0	Macrocarpal B	1-C11	472.614	10(μ)M	DMSO
NCRC23423	BBP00031	142628-53-3	Macrocarpal C	1-D2	454.598	10(μ)M	DMSO
NCRC23424	BBP00032	172617-99-1	Eucalyptone	1-D3	486.597	10(μ)M	DMSO
NCRC23425	BBP00033	145382-68-9	Sideroxylonal A	1-D4	500.495	10(μ)M	DMSO
NCRC23426	BBP00034	96087-10-4	Massoniresinol	1-D5	392.4	10(μ)M	DMSO
NCRC23427	BBP00035	118-34-3	Syringin	1-D6	372.367	10(μ)M	DMSO
NCRC23428	BBP00036	92-61-5	Scopoletin	1-D7	192.168	10(μ)M	DMSO
NCRC23429	BBP00038	77-52-1	Ursolic acid	1-D8	456.7	10(μ)M	DMSO
NCRC23430	BBP00039	35959-08-1	3-Acetoxy-11-ursen-28,13-olide	1-D9	496.721	10(μ)M	DMSO
NCRC23431	BBP00040	35959-05-8	3-Hydroxy-11-ursen-28,13-olide	1-D10	454.684	10(μ)M	DMSO
NCRC23432	BBP00041	13849-91-7	Pomolic acid	1-D11	472.7	10(μ)M	DMSO
NCRC23433	BBP00042	473-98-3	Betulin	1-E2	442.717	10(μ)M	DMSO
NCRC23434	BBP00043	472-15-1	Betulinic acid	1-E3	456.7	10(μ)M	DMSO
NCRC23435	BBP00044	149751-81-5	Tereticornate A	1-E4	630.853	10(μ)M	DMSO
NCRC23436	BBP00045	7372-30-7	Acetylursolic acid	1-E5	498.737	10(μ)M	DMSO
NCRC23437	BBP00046	55722-32-2	Stigmasta-4,22-dien-3-one	1-E6	410.675	10(μ)M	DMSO
NCRC23438	BBP00047	83-48-7	Stigmasterol	1-E7	412.691	10(μ)M	DMSO
NCRC23439	BBP00048	123690-76-6	Bongardol	1-E8	544.892	10(μ)M	DMSO
NCRC23440	BBP00049	65-85-0	Benzoic acid	1-E9	122.121	10(μ)M	DMSO
NCRC23441	BBP00050	552-41-0	Paeonol	1-E10	166.174	10(μ)M	DMSO
NCRC23442	BBP00051	99173-00-9	No	1-E11	182.216	10(μ)M	DMSO
NCRC23443	BBP00052	25279-15-6	20S,24R-Epoxy-dammar-12,25-diol-3-one	1-F2	474.716	10(μ)M	DMSO

NCRC23444	BBP00053	22549-21-9	Ocotillone	1-F3	458.716	10(μ)M	DMSO
NCRC23445	BBP00054	6474-90-4	Tetrahydroalstonine	1-F4	352.427	10(μ)M	DMSO
NCRC23446	BBP00055	474-58-8	Daucosterol	1-F5	576.847	10(μ)M	DMSO
NCRC23447	BBP00056	121-34-6	Vanillic acid	1-F6	168.147	10(μ)M	DMSO
NCRC23448	BBP00057	21978-49-4	Bombiprenone	1-F7	603.015	10(μ)M	DMSO
NCRC23449	BBP00058	771-50-6	1H-Indole-3-carboxylic acid	1-F8	161.157	10(μ)M	DMSO
NCRC23450	BBP00059	145-13-1	Pregnenolone	1-F9	316.478	10(μ)M	DMSO
NCRC23451	BBP00060	111-02-4	Squalene	1-F10	410.718	10(μ)M	DMSO
NCRC23452	BBP00061	479-43-6	Canthin-6-one	1-F11	220.226	10(μ)M	DMSO
NCRC23453	BBP00062	28594-00-5	22-Dehydroclerosteryl acetate	1-G2	452.712	10(μ)M	DMSO
NCRC23454	BBP00063	26187-80-4	Foliamenthoic acid	1-G3	184.232	10(μ)M	DMSO
NCRC23455	BBP00064	38990-03-3	Gelsevirine	1-G4	352.427	10(μ)M	DMSO
NCRC23456	BBP00065	6882-99-1	Sempervirine	1-G5	272.344	10(μ)M	DMSO
NCRC23457	BBP00066	36450-02-9	6-Hydroxystigmast-4-en-3-one	1-G6	428.69	10(μ)M	DMSO
NCRC23458	BBP00067	5081-51-6	Vasicinol	1-G7	204.225	10(μ)M	DMSO
NCRC23459	BBP00068	51419-51-3	Odonicin	1-G8	430.491	10(μ)M	DMSO
NCRC23460	BBP00069	66178-02-7	Malic acid 4-Me ester	1-G9	148.114	10(μ)M	DMSO
NCRC23461	BBP00070	1058-61-3	Sitostenone	1-G10	412.691	10(μ)M	DMSO
NCRC23462	BBP00071	110414-77-2	Gelsemiol	1-G11	200.232	10(μ)M	DMSO
NCRC23463	BBP00072	22255-40-9	Loganic acid	1-H2	376.356	10(μ)M	DMSO
NCRC23464	BBP00074	122872-03-1	2-Desoxy-4-epi-pulchellin	1-H3	250.333	10(μ)M	DMSO
NCRC23465	BBP00075	No	4-Acetoxy-11(13)-pseudoguaien-12,8-olide	1-H4	292.37	10(μ)M	DMSO
NCRC23466	BBP00076	1187925-31-0	Carabrolactone B	1-H5	266.333	10(μ)M	DMSO
NCRC23467	BBP00077	124111-47-3	2-Hydroxytetracosanoic acid ethyl ester	1-H6	412.689	10(μ)M	DMSO
NCRC23468	BBP00078	5231-60-7	Vindorosine	1-H7	426.505	10(μ)M	DMSO
NCRC23469	BBP00079	126724-95-6	Tilifodiolide	1-H8	336.338	10(μ)M	DMSO
NCRC23470	BBP00080	21671-00-1	Shoreic acid	1-H9	474.716	10(μ)M	DMSO
NCRC23471	BBP00081	20283-92-5	Rosmarinic acid	1-H10	360.315	10(μ)M	DMSO
NCRC23472	BBP00082	62218-55-7	4-Hydroxy-2,6,6-trimethyl-1-cyclohexenecarboxylic acid	1-H11	184.232	10(μ)M	DMSO
NCRC23473	BBP00083	1187925-30-9	Carabrolactone A	2-A2	282.332	10(μ)M	DMSO
NCRC23474	BBP00084	115321-32-9	Isosalvipuberulin	2-A3	334.322	10(μ)M	DMSO
NCRC23475	BBP00085	2061-64-5	Ergosterol peroxide	2-A4	428.647	10(μ)M	DMSO
NCRC23476	BBP00086	41590-29-8	4,R-ajmalicine N-oxide	2-A5	368.426	10(μ)M	DMSO
NCRC23477	BBP00087	263844-80-0	3-Acetoxy-27-hydroxy-20(29)-lupen-28-oic acid methyl ester	2-A6	528.763	10(μ)M	DMSO
NCRC23478	BBP00088	486-64-6	Vasicinone	2-A7	202.209	10(μ)M	DMSO
NCRC23479	BBP00089	80787-59-3	1-Hydroxycanthin-6-one	2-A8	236.226	10(μ)M	DMSO
NCRC23480	BBP00090	84745-95-9	Eriocalyxin B	2-A9	344.402	10(μ)M	DMSO
NCRC23481	BBP00091	28593-92-2	Docosyl caffeate	2-A10	488.742	10(μ)M	DMSO
NCRC23482	BBP00092	1159579-44-8	Alstonic acid A	2-A11	456.7	10(μ)M	DMSO
NCRC23483	BBP00093	115334-05-9	Dihydroniloticin	2-B2	458.716	10(μ)M	DMSO
NCRC23484	BBP00094	60796-64-7	Norbraylin	2-B3	244.243	10(μ)M	DMSO
NCRC23485	BBP00095	26585-14-8	Crenatine	2-B4	226.274	10(μ)M	DMSO
NCRC23486	BBP00096	442-51-3	Harmine	2-B5	212.247	10(μ)M	DMSO
NCRC23487	BBP00097	18786-24-8	Serpentine hydrochloride	2-B6	384.856	10(μ)M	DMSO
NCRC23488	BBP00098	480-10-4	Astragalin	2-B7	448.377	10(μ)M	DMSO
NCRC23489	BBP00099	No	7-Geranyloxy-5-methoxycoumarin	2-B8	328.402	10(μ)M	DMSO
NCRC23490	BBP00100	89915-39-9	Beta-Carboline-1-propanoic acid	2-B9	240.257	10(μ)M	DMSO
NCRC23491	BBP00101	96850-29-2	Maoecrystal B	2-B10	388.454	10(μ)M	DMSO
NCRC23492	BBP00102	304642-94-2	Xerophilusin G	2-B11	422.469	10(μ)M	DMSO
NCRC23493	BBP00103	2239-24-9	Serratenediol	2-C2	442.717	10(μ)M	DMSO
NCRC23494	BBP00104	3984-73-4	Methyllinderone	2-C3	300.306	10(μ)M	DMSO

NCRC23495	BBP00105	No	8-Geranyloxy-5,7-dimethoxycoumarin	2-C4	358.428	10(μ)M	DMSO
NCRC23496	BBP00106	210108-87-5	2,5,14-Triacetoxy-3-benzoyloxy-8,15-dihydroxy-7-isobutyroyloxy-9-nicotinoyloxyjatropha-6(17),11E-diene	2-C5	807.879	10(μ)M	DMSO
NCRC23497	BBP00107	981-15-7	Ailanthone	2-C6	376.4	10(μ)M	DMSO
NCRC23498	BBP00108	60796-65-8	5,7,8-Trimethoxycoumarin	2-C7	236.221	10(μ)M	DMSO
NCRC23499	BBP00109	1782-79-2	Linderone	2-C8	286.279	10(μ)M	DMSO
NCRC23500	BBP00110	82467-50-3	R(+)-Gomisin M1	2-C9	386.438	10(μ)M	DMSO
NCRC23501	BBP00111	210108-89-7	2,5,7,14-Tetraacetoxy-3-benzoyloxy-8,15-dihydroxy-9-nicotinoyloxyjatropha-6(17),11E-diene	2-C10	779.826	10(μ)M	DMSO
NCRC23502	BBP00112	42438-78-8	Pashanone	2-C11	300.306	10(μ)M	DMSO
NCRC23503	BBP00113	480-37-5	Pinostrobin	2-D2	270.28	10(μ)M	DMSO
NCRC23504	BBP00114	96917-26-9	Artanin	2-D3	290.311	10(μ)M	DMSO
NCRC23505	BBP00115	137182-37-7	Toddalosin	2-D4	562.607	10(μ)M	DMSO
NCRC23506	BBP00116	4547-24-4	Corosolic acid	2-D5	472.7	10(μ)M	DMSO
NCRC23507	BBP00117	27741-01-1	Geniposidic acid	2-D6	374.34	10(μ)M	DMSO
NCRC23508	BBP00118	19956-54-8	Methyllucidone	2-D7	270.28	10(μ)M	DMSO
NCRC23509	BBP00119	1158845-78-3	N^1-methoxymethyl picrinine	2-D8	382.453	10(μ)M	DMSO
NCRC23510	BBP00120	480-39-7	Pinocembrin	2-D9	256.253	10(μ)M	DMSO
NCRC23511	BBP00121	3162-45-6	5,7-Dihydroxy-6,8-dimethoxyflavone	2-D10	314.289	10(μ)M	DMSO
NCRC23512	BBP00122	480-20-6	Aromadendrin	2-D11	288.252	10(μ)M	DMSO
NCRC23513	BBP00123	102519-34-6	2,3,23-Trihydroxy-12-oleanen-28-oic acid	2-E2	488.699	10(μ)M	DMSO
NCRC23514	BBP00124	66322-34-7	Dihydroguaiaretic acid	2-E3	330.418	10(μ)M	DMSO
NCRC23515	BBP00126	1169805-98-4	Sculponeatin N	2-E4	404.583	10(μ)M	DMSO
NCRC23516	BBP00127	20045-06-1	Picralinal	2-E5	366.41	10(μ)M	DMSO
NCRC23517	BBP00128	530-53-0	Deoxyvasicinone	2-E6	186.21	10(μ)M	DMSO
NCRC23518	BBP00129	85287-60-1	Sculponeatin A	2-E7	360.401	10(μ)M	DMSO
NCRC23519	BBP00130	19956-53-7	Lucidone	2-E8	256.253	10(μ)M	DMSO
NCRC23520	BBP00131	56324-22-2	19,20-(E)-Isovallesamine	2-E9	340.416	10(μ)M	DMSO
NCRC23521	BBP00132	545-24-4	5-Glutinen-3-ol	2-E10	426.717	10(μ)M	DMSO
NCRC23522	BBP00133	1195233-59-0	Ethyllucidone	2-E11	284.307	10(μ)M	DMSO
NCRC23523	BBP00134	670257-89-3	Pepluanin A	2-F2	821.863	10(μ)M	DMSO
NCRC23524	BBP00135	191545-24-1	Epieriocalyxin A	2-F3	344.402	10(μ)M	DMSO
NCRC23525	BBP00136	140-10-3	Cinnamic acid	2-F4	148.159	10(μ)M	DMSO
NCRC23526	BBP00137	18956-15-5	Pinostrobin chalcone	2-F5	270.28	10(μ)M	DMSO
NCRC23527	BBP00138	41059-80-7	Lipiferolide	2-F6	306.354	10(μ)M	DMSO
NCRC23528	BBP00139	128397-09-1	Hyptadienic acid	2-F7	470.684	10(μ)M	DMSO
NCRC23529	BBP00140	210108-86-4	2,5,7,8,9,14-Hexaacetoxy-3-benzoyloxy-15-hydroxy-jatropha-6(17),11E-diene	2-F8	758.805	10(μ)M	DMSO
NCRC23530	BBP00141	919120-78-8	19-[(beta-D-glucopyranosyl)oxy]-19-oxo-ent-labda-8(17),13-dien-16,15-olide	2-F9	494.575	10(μ)M	DMSO
NCRC23531	BBP00142	475-75-2	Liriodenine	2-F10	275.258	10(μ)M	DMSO
NCRC23532	BBP00143	99882-10-7	Kaempferol 3-O-alfa-L-arabinoside	2-F11	418.351	10(μ)M	DMSO

NCRC23533	BBP00144	210108-88-6	2,5,9,14-Tetraacetoxy-3-benzoyloxy-8,15-dihydroxy-7-isobutyroyloxyjatropha-6(17),11E-diene (4.4708 g)	2-G2	744.822	10(μ)M	DMSO
NCRC23534	BBP00145	117-10-2	Dantron	2-G3	240.211	10(μ)M	DMSO
NCRC23535	BBP00146	61597-83-9	13-Hydroxy-8,11,13-podocarpatrien-18-oic acid	2-G4	274.355	10(μ)M	DMSO
NCRC23536	BBP00147	304-21-2	Harmaline	2-G5	214.263	10(μ)M	DMSO
NCRC23537	BBP00148	4684-32-6	Picrinine	2-G6	338.4	10(μ)M	DMSO
NCRC23538	BBP00149	28254-53-7	Reynosin	2-G7	248.318	10(μ)M	DMSO
NCRC23539	BBP00150	477529-70-7	Sculponeatin K	2-G8	346.417	10(μ)M	DMSO
NCRC23540	BBP00151	36417-86-4	N-p-trans-Coumaroyltyramine	2-G9	283.322	10(μ)M	DMSO
NCRC23541	BBP00152	1177-14-6	DL-Syringaresinol	2-G10	418.437	10(μ)M	DMSO
NCRC23542	BBP00153	263844-79-7	3,27-Dihydroxy-20(29)-lupen-28-oic acid methyl ester	2-G11	486.726	10(μ)M	DMSO
NCRC23543	BBP00154	1159579-45-9	Alstonic acid B	2-H2	454.684	10(μ)M	DMSO
NCRC23544	BBP00156	19013-03-7	Eupatoriochromene	2-H3	218.248	10(μ)M	DMSO
NCRC23545	BBP00157	No	3,4-O-Isopropylidene-2-methylbutane-1,2,3,4-tetrol	2-H4	176.21	10(μ)M	DMSO
NCRC23546	BBP00158	210108-91-1	5,8,9,10,14-Pentaacetoxy-3-benzoyloxy-15-hydroxypepluane	2-H5	700.769	10(μ)M	DMSO
NCRC23547	BBP00159	123-08-0	4-Hydroxybenzaldehyde	2-H6	122.121	10(μ)M	DMSO
NCRC23548	BBP00160	866111-14-0	Clausine Z	2-H7	227.215	10(μ)M	DMSO
NCRC23549	BBP00161	No	2-methylbutane-1,2,3,4-tetrol	2-H8	136.146	10(μ)M	DMSO
NCRC23550	BBP00162	6601-62-3	Cirsimaritin	2-H9	314.289	10(μ)M	DMSO
NCRC23551	BBP00163	6483-15-4	Sophocarpine	2-H10	246.348	10(μ)M	DMSO
NCRC23552	BBP00164	67667-62-3	De-O-	2-H11	218.248	10(μ)M	DMSO
NCRC23553	BBP00166	59979-57-6	Tagitinin F	3-A2	348.39	10(μ)M	DMSO
NCRC23554	BBP00167	20628-09-5	Encecalin	3-A3	232.275	10(μ)M	DMSO
NCRC23555	BBP00168	94285-22-0	Acetyldihydromicromelin A	3-A4	332.305	10(μ)M	DMSO
NCRC23556	BBP00169	109794-97-0	Harmalidine	3-A5	254.327	10(μ)M	DMSO
NCRC23557	BBP00170	623-05-2	4-Hydroxybenzyl alcohol	3-A6	124.137	10(μ)M	DMSO
NCRC23558	BBP00171	93710-27-1	Leuconolam	3-A7	326.39	10(μ)M	DMSO
NCRC23559	BBP00172	1169806-02-3	Sculponeatic acid	3-A8	470.684	10(μ)M	DMSO
NCRC23560	BBP00173	30484-88-9	Mearnsitrin	3-A9	478.403	10(μ)M	DMSO
NCRC23561	BBP00174	521-34-6	Sciadopitysin	3-A10	580.538	10(μ)M	DMSO
NCRC23562	BBP00175	54835-70-0	Roseoside	3-A11	386.437	10(μ)M	DMSO
NCRC23563	BBP00176	1570-09-8	5,7-Dihydroxy-3,4',8-trimethoxyflavone	3-B2	344.315	10(μ)M	DMSO
NCRC23564	BBP00177	52932-74-8	Neosophoramine	3-B3	244.332	10(μ)M	DMSO
NCRC23565	BBP00178	77784-22-6	Dehydrocrebanine	3-B4	337.369	10(μ)M	DMSO
NCRC23566	BBP00179	570-72-9	Stigmasta-5,8-dien-3-ol	3-B5	412.691	10(μ)M	DMSO
NCRC23567	BBP00180	26904-64-3	Sophocarpidine	3-B6	262.347	10(μ)M	DMSO
NCRC23568	BBP00181	30244-37-2	1-Hydroxybaccatin I	3-B7	652.683	10(μ)M	DMSO
NCRC23569	BBP00182	10284-63-6	D-Pinitol	3-B8	194.182	10(μ)M	DMSO
NCRC23570	BBP00183	7374-79-0	Acanthoside B	3-B9	580.578	10(μ)M	DMSO
NCRC23571	BBP00184	100201-57-8	2,3-dihydroxy-3-(4-hydroxyphenyl)propanoic acid	3-B10	198.173	10(μ)M	DMSO
NCRC23572	BBP00185	1169806-00-1	Sculponeatin O	3-B11	440.615	10(μ)M	DMSO
NCRC23573	BBP00186	59979-61-2	Tagitinin A	3-C2	368.421	10(μ)M	DMSO
NCRC23574	BBP00187	57627-75-5	3-Hydroxy-4-methoxy-benzenepropanol	3-C3	182.216	10(μ)M	DMSO
NCRC23575	BBP00188	550-90-3	Lupanine	3-C4	248.364	10(μ)M	DMSO
NCRC23576	BBP00189	125288-25-7	2,4,6-Trimethoxyphenol 1-O-beta-D-glucopyranoside	3-C5	346.33	10(μ)M	DMSO

NCRC23577	BBP00190	69251-96-3	(+)-Pinoresinol-4-O-beta-D-glucopyranoside	3-C6	520.526	10(μ)M	DMSO
NCRC23578	BBP00191	6882-68-4	Sophoridine	3-C7	248.364	10(μ)M	DMSO
NCRC23579	BBP00192	531-29-3	Coniferin	3-C8	342.341	10(μ)M	DMSO
NCRC23580	BBP00193	10210-17-0	3-(4-Hydroxyphenyl)-1-propanol	3-C9	152.19	10(μ)M	DMSO
NCRC23581	BBP00194	27548-93-2	Baccatin III	3-C10	586.627	10(μ)M	DMSO
NCRC23582	BBP00195	61152-62-3	4-(3,4-Dihydroxyphenyl)-2-butanone	3-C11	180.2	10(μ)M	DMSO
NCRC23583	BBP00196	3602-54-8	2,4-Dihydroxy-6-methoxyacetophenone	3-D2	182.173	10(μ)M	DMSO
NCRC23584	BBP00197	479-90-3	Artemetin	3-D3	388.368	10(μ)M	DMSO
NCRC23585	BBP00198	39815-40-2	Epitulipinolide diepoxide	3-D4	322.353	10(μ)M	DMSO
NCRC23586	BBP00199	99694-90-3	Scholaricine	3-D5	356.416	10(μ)M	DMSO
NCRC23587	BBP00200	17912-87-7	Myricitrin	3-D6	464.376	10(μ)M	DMSO
NCRC23588	BBP00201	517-63-5	Stephanine	3-D7	309.359	10(μ)M	DMSO
NCRC23589	BBP00202	705973-69-9	14beta-Benzoyloxy-2-deacetylbaccatin VI	3-D8	730.752	10(μ)M	DMSO
NCRC23590	BBP00203	203524-64-5	2,4,6,6-Tetramethyl-3(6H)-pyridinone	3-D9	151.206	10(μ)M	DMSO
NCRC23591	BBP00204	154-23-4	Catechin	3-D10	290.268	10(μ)M	DMSO
NCRC23592	BBP00205	153229-31-3	Taxayunnansin A	3-D11	672.716	10(μ)M	DMSO
NCRC23593	BBP00206	603-56-5	Chrysosplenetin	3-E2	374.341	10(μ)M	DMSO
NCRC23594	BBP00207	13408-56-5	Ponasterone A	3-E3	464.635	10(μ)M	DMSO
NCRC23595	BBP00208	15085-71-9	Micromelin	3-E4	288.252	10(μ)M	DMSO
NCRC23596	BBP00209	25127-29-1	Crebanine	3-E5	339.385	10(μ)M	DMSO
NCRC23597	BBP00210	99-96-7	4-Hydroxybenzoic acid	3-E6	138.121	10(μ)M	DMSO
NCRC23598	BBP00211	34086-50-5	Alpinumisoflavone	3-E7	336.338	10(μ)M	DMSO
NCRC23599	BBP00212	5257-08-9	Glycozolinine	3-E8	197.233	10(μ)M	DMSO
NCRC23600	BBP00213	4090-18-0	Sinoacutine	3-E9	327.374	10(μ)M	DMSO
NCRC23601	BBP00333	6054-10-0	Braylin	3-E10	258.269	10(μ)M	DMSO
NCRC23602	BBP00215	23624-21-7	Putraflavone	3-E11	566.511	10(μ)M	DMSO
NCRC23603	BBP00216	126594-73-8	Vallesamine N-oxide	3-F2	356.416	10(μ)M	DMSO
NCRC23604	BBP00217	501-36-0	Resveratrol	3-F3	228.243	10(μ)M	DMSO
NCRC23605	BBP00218	625096-18-6	Viniferol D	3-F4	680.698	10(μ)M	DMSO
NCRC23606	BBP00219	32981-86-5	10-Deacetylbaccatin III	3-F5	544.59	10(μ)M	DMSO
NCRC23607	BBP00220	62218-08-0	Viniferin	3-F6	454.471	10(μ)M	DMSO
NCRC23608	BBP00221	No	No	3-F7	416.378	10(μ)M	DMSO
NCRC23609	BBP00222	98770-70-8	3-(4-Hydroxy-3-methoxyphenyl)propyl tetracosanoate	3-F8	532.838	10(μ)M	DMSO
NCRC23610	BBP00223	18457-44-8	Taxinine B	3-F9	664.739	10(μ)M	DMSO
NCRC23611	BBP00224	191547-12-3	2-Deacetoxytaxinine B	3-F10	606.703	10(μ)M	DMSO
NCRC23612	BBP00225	62218-13-7	alfa-Viniferin	3-F11	678.682	10(μ)M	DMSO
NCRC23613	BBP00226	85699-62-3	alfa-Conidendrin	3-G2	356.369	10(μ)M	DMSO
NCRC23614	BBP00227	82425-45-4	Gomisin M2	3-G3	386.438	10(μ)M	DMSO
NCRC23615	BBP00228	129724-83-0	3-Hydroxylanost-9(11),24-dien-26-oic acid	3-G4	456.7	10(μ)M	DMSO
NCRC23616	BBP00229	107783-45-9	Coccinic acid	3-G5	454.684	10(μ)M	DMSO
NCRC23617	BBP00230	29700-22-9	Oxyresveratrol	3-G6	244.243	10(μ)M	DMSO
NCRC23618	BBP00231	10173-01-0	Jaceidin	3-G7	360.315	10(μ)M	DMSO
NCRC23619	BBP00232	78432-78-7	19-Hydroxybaccatin III	3-G8	602.626	10(μ)M	DMSO
NCRC23620	BBP00234	50461-86-4	Araneosol	3-G9	374.341	10(μ)M	DMSO
NCRC23621	BBP00235	6750-60-3	Spathulenol	3-G10	220.35	10(μ)M	DMSO
NCRC23622	BBP00236	35796-71-5	Zapoterin	3-G11	470.512	10(μ)M	DMSO
NCRC23623	BBP00237	17635-59-5	Dihydropinosylvin methyl ether	3-H2	228.286	10(μ)M	DMSO

NCRC23624	BBP00238	35302-70-6	(E)-3-Hydroxy-5-methoxystilbene	3-H3	226.27	10(μ)M	DMSO
NCRC23625	BBP00239	1189362-86-4	1,3-Dihydroxy-4-methoxy-10-methylacridin-9(10H)-one	3-H4	271.268	10(μ)M	DMSO
NCRC23626	BBP00240	3443-28-5	Prudomestin	3-H5	330.289	10(μ)M	DMSO
NCRC23627	BBP00241	740810-42-8	Cudratricusxanthone A	3-H6	396.433	10(μ)M	DMSO
NCRC23628	BBP00242	4586-68-9	Ilicic acid	3-H7	252.349	10(μ)M	DMSO
NCRC23629	BBP00243	520-18-3	Kaempferol	3-H8	286.236	10(μ)M	DMSO
NCRC23630	BBP00244	120-47-8	Ethylparaben	3-H9	166.174	10(μ)M	DMSO
NCRC23631	BBP00245	83864-70-4	Angeloylisogomisin O	3-H10	498.565	10(μ)M	DMSO
NCRC23632	BBP00246	220935-39-7	13-O-Deacetyltaxumairol Z	3-H11	604.642	10(μ)M	DMSO
NCRC23633	BBP00247	97931-41-4	Methyl 3-carbazolecarboxylate	4-A2	225.243	10(μ)M	DMSO
NCRC23634	BBP00248	62393-88-8	Kadsuric acid	4-A3	470.684	10(μ)M	DMSO
NCRC23635	BBP00249	5085-72-3	Friedelanol	4-A4	428.733	10(μ)M	DMSO
NCRC23636	BBP00250	508-71-4	Rosenonolactone	4-A5	316.435	10(μ)M	DMSO
NCRC23637	BBP00251	480-40-0	Chrysin	4-A6	254.238	10(μ)M	DMSO
NCRC23638	BBP00252	95135-98-1	Artemetin acetate	4-A7	430.405	10(μ)M	DMSO
NCRC23639	BBP00253	115028-67-6	Pseudolaric acid D	4-A8	318.45	10(μ)M	DMSO
NCRC23640	BBP00254	150-19-6	m-Methoxyphenol	4-A9	124.137	10(μ)M	DMSO
NCRC23641	BBP00255	354553-35-8	Caraphenol A	4-A10	676.666	10(μ)M	DMSO
NCRC23642	BBP00334	56421-13-7	Eichlerianic acid	4-A11	474.716	10(μ)M	DMSO
NCRC23643	BBP00257	62615-63-8	4,6,7-Trimethoxy-5-methylcoumarin	4-B2	250.247	10(μ)M	DMSO
NCRC23644	BBP00258	24808-04-6	(-)-Epiafzelechin	4-B3	274.269	10(μ)M	DMSO
NCRC23645	BBP00259	82508-33-6	Methyl pseudolarate A	4-B4	402.481	10(μ)M	DMSO
NCRC23646	BBP00260	84955-05-5	Cudraxanthone B	4-B5	394.417	10(μ)M	DMSO
NCRC23647	BBP00261	82508-34-7	Methylpseudolarate B	4-B6	446.49	10(μ)M	DMSO
NCRC23648	BBP00262	1016260-22-2	Kadsuracoccinic acid A	4-B7	468.668	10(μ)M	DMSO
NCRC23649	BBP00263	20554-84-1	Parthenolide	4-B8	248.318	10(μ)M	DMSO
NCRC23650	BBP00264	33708-72-4	Pachypodol	4-B9	344.315	10(μ)M	DMSO
NCRC23651	BBP00265	548-82-3	Pinobanksin	4-B10	272.253	10(μ)M	DMSO
NCRC23652	BBP00267	211799-56-3	2',4',5'-Trimethoxy-2",2"-dimethylpyrano[5",6":6,7]isoflavone	4-B11	394.417	10(μ)M	DMSO
NCRC23653	BBP00268	93888-59-6	9-Oxo-2,7-bisaboladien-15-oic acid	4-C2	250.333	10(μ)M	DMSO
NCRC23654	BBP00269	482-39-3	Afzelin	4-C3	432.378	10(μ)M	DMSO
NCRC23655	BBP00270	870456-88-5	3,23-dioxo-9,19-Cyclolanost-24-en-26-oic acid	4-C4	468.668	10(μ)M	DMSO
NCRC23656	BBP00271	250293-31-3	Excavatin M	4-C5	360.358	10(μ)M	DMSO
NCRC23657	BBP00272	42830-48-8	Catechin 7-xyloside	4-C6	422.383	10(μ)M	DMSO
NCRC23658	BBP00273	89-84-9	2',4'-Dihydroxyacetophenone	4-C7	152.147	10(μ)M	DMSO
NCRC23659	BBP00274	128255-08-3	Pachyaximine A	4-C8	359.588	10(μ)M	DMSO
NCRC23660	BBP00275	3484-61-5	12-Hydroxyabietic acid	4-C9	318.45	10(μ)M	DMSO
NCRC23661	BBP00276	101959-37-9	Octacosyl (E)-ferulate	4-C10	586.928	10(μ)M	DMSO
NCRC23662	BBP00277	2196-18-1	beta-Hydroxypropiovanillone	4-C11	196.2	10(μ)M	DMSO
NCRC23663	BBP00278	1859-87-6	Echinulin	4-D2	461.639	10(μ)M	DMSO
NCRC23664	BBP00279	35833-62-6	Cabraleadiol 3-acetate	4-D3	502.769	10(μ)M	DMSO
NCRC23665	BBP00280	57672-77-2	Baccatin IV	4-D4	652.683	10(μ)M	DMSO
NCRC23666	BBP00281	62394-00-7	Alboctalol	4-D5	488.485	10(μ)M	DMSO
NCRC23667	BBP00283	99-93-4	4'-Hydroxyacetophenone	4-D6	136.148	10(μ)M	DMSO
NCRC23668	BBP00284	289054-34-8	Ergosta-5,24(28)-diene-3,7,16-triol	4-D7	430.663	10(μ)M	DMSO
NCRC23669	BBP00285	51551-29-2	Neoechinulin A	4-D8	323.389	10(μ)M	DMSO
NCRC23670	BBP00286	37126-91-3	Murrangatin	4-D9	276.285	10(μ)M	DMSO
NCRC23671	BBP00288	60125-23-7	3-(2-Hydroxyphenyl)-2-propenal	4-D10	148.159	10(μ)M	DMSO

NCRC23672	BBP00289	132342-55-3	Methyl isocostate	4-D11	248.361	10(μ)M	DMSO
NCRC23673	BBP00290	522-12-3	Quercetin 3-O-alpha-L-	4-E2	448.377	10(μ)M	DMSO
NCRC23674	BBP00291	1033288-92-4	Pre-schisanartanin B	4-E3	590.659	10(μ)M	DMSO
NCRC23675	BBP00292	7432-28-2	Schizandrin	4-E4	432.507	10(μ)M	DMSO
NCRC23676	BBP00293	97938-31-3	Leachianone A	4-E5	438.513	10(μ)M	DMSO
NCRC23677	BBP00294	13850-16-3	Tormentic acid	4-E6	488.699	10(μ)M	DMSO
NCRC23678	BBP00295	252333-71-4	9,9'-O-isopropyllidene-isolariciresinol	4-E7	400.465	10(μ)M	DMSO
NCRC23679	BBP00296	3650-43-9	beta-Costic acid	4-E8	234.334	10(μ)M	DMSO
NCRC23680	BBP00297	135820-80-3	Blumenol C glucoside	4-E9	372.453	10(μ)M	DMSO
NCRC23681	BBP00298	114297-20-0	Soyacerebroside I	4-E10	714.025	10(μ)M	DMSO
NCRC23682	BBP00299	5128-43-8	5,7-diacetoxy-3,4',8-trimethoxyflavone	4-E11	428.389	10(μ)M	DMSO
NCRC23683	BBP00300	185845-89-0	Pterodontic acid	4-F2	234.334	10(μ)M	DMSO
NCRC23684	BBP00301	135541-40-1	Cudraxanthone L	4-F3	396.433	10(μ)M	DMSO
NCRC23685	BBP00302	109592-60-1	Pinocembrin 7-acetate	4-F4	298.29	10(μ)M	DMSO
NCRC23686	BBP00303	132185-83-2	5alpha-Hydroxycostic acid	4-F5	250.333	10(μ)M	DMSO
NCRC23687	BBP00304	548-83-4	Galangin	4-F6	270.237	10(μ)M	DMSO
NCRC23688	BBP00305	No	No	4-F7	262.344	10(μ)M	DMSO
NCRC23689	BBP00306	18422-83-8	Dihydromorin	4-F8	304.252	10(μ)M	DMSO
NCRC23690	BBP00307	101312-79-2	Formosanol	4-F9	372.412	10(μ)M	DMSO
NCRC23691	BBP00308	128255-16-3	Axillaridine A	4-F10	462.667	10(μ)M	DMSO
NCRC23692	BBP00309	482-36-0	Hyperin	4-F11	464.376	10(μ)M	DMSO
NCRC23693	BBP00310	69586-96-5	Tupichilignan A	4-G2	402.438	10(μ)M	DMSO
NCRC23694	BBP00311	22798-98-7	Ecdysterone 2,3:20,22-diacetonide	4-G3	560.762	10(μ)M	DMSO
NCRC23695	BBP00312	2035-15-6	(-)-Maackiain	4-G4	284.263	10(μ)M	DMSO
NCRC23696	BBP00313	52117-69-8	3-O-Acetylpinobanksin	4-G5	314.289	10(μ)M	DMSO
NCRC23697	BBP00314	76754-24-0	Lupalbigenin	4-G6	406.471	10(μ)M	DMSO
NCRC23698	BBP00315	No	Cudraxanthone L triacetate	4-G7	522.543	10(μ)M	DMSO
NCRC23699	BBP00318	2545-00-8	(+)-Afzelechin	4-G8	274.269	10(μ)M	DMSO
NCRC23700	BBP00319	94285-06-0	Dihydromicromelin B	4-G9	290.268	10(μ)M	DMSO
NCRC23701	BBP00320	120-08-1	Scoparone	4-G10	206.195	10(μ)M	DMSO
NCRC23702	BBP00321	58865-88-6	9-Oxonerolidol	4-G11	236.35	10(μ)M	DMSO
NCRC23703	BBP00323	6674-40-4	5-hydroxy-7-acetoxyflavone	4-H2	296.274	10(μ)M	DMSO
NCRC23704	BBP00324	226561-02-0	Amaronol B	4-H3	334.278	10(μ)M	DMSO
NCRC23705	BBP00325	27200-12-0	Ampelopsin	4-H4	320.251	10(μ)M	DMSO
NCRC23706	BBP00326	132185-84-3	5beta-Hydroxycostic acid	4-H5	250.333	10(μ)M	DMSO
NCRC23707	BBP00327	117-39-5	Quercetin	4-H6	302.236	10(μ)M	DMSO
NCRC23708	BBP00328	705-15-7	2'-Hydroxy-5'-methoxyacetophenone	4-H7	166.174	10(μ)M	DMSO
NCRC23709	BBP00335	25488-59-9	2,3-Bis(3,4-dimethoxybenzyl)butyrolactone	4-H8	386.438	10(μ)M	DMSO
NCRC23710	BBP00330	73281-83-1	1,4-Dihydro-1,2-dimethyl-4-oxo-3-quinolinecarboxylic acid	4-H9	217.221	10(μ)M	DMSO
NCRC23711	BBP00331	6665-78-7	5,7-Diacetoxyflavone	4-H10	338.311	10(μ)M	DMSO
NCRC23712	BBP00332	22798-96-5	Ecdysterone 20,22-monoacetonide	4-H11	520.698	10(μ)M	DMSO
NCRC23713	BBP00336	22139-77-1	Pinosylvin	5-A2	212.244	10(μ)M	DMSO
NCRC23714	BBP00337	No	No	5-A3	552.524	10(μ)M	DMSO
NCRC23715	BBP00338	632-85-9	Wogonin	5-A4	284.263	10(μ)M	DMSO
NCRC23716	BBP00339	226560-96-9	Amaronol A	5-A5	320.251	10(μ)M	DMSO
NCRC23717	BBP00341	659738-08-6	Micranoic acid A	5-A6	344.488	10(μ)M	DMSO
NCRC23718	BBP00342	14531-52-3	Dihydropinosylvin	5-A7	214.26	10(μ)M	DMSO
NCRC23719	BBP00343	3420-57-3	3-Methoxyfuran	5-A8	98.0999	10(μ)M	DMSO
NCRC23720	BBP00344	60132-35-6	Pterodondiol	5-A9	240.382	10(μ)M	DMSO
NCRC23721	BBP00345	132351-58-7	5-Acetoxy-7-hydroxyflavone	5-A10	296.274	10(μ)M	DMSO

NCRC23722	BBP00346	No	No	5-A11	270.323	10(μ)M	DMSO
NCRC23723	BBP00347	487-36-5	(+)-Pinoresinol	5-B2	358.385	10(μ)M	DMSO
NCRC23724	BBP00348	152243-70-4	Uncargenin C	5-B3	488.699	10(μ)M	DMSO
NCRC23725	BBP00349	23246-80-2	5,7-Diacetoxy-8-methoxyflavone	5-B4	368.337	10(μ)M	DMSO
NCRC23726	BBP00350	95480-80-1	5-Hydroxy-7-acetoxy-8-methoxyflavone	5-B5	326.3	10(μ)M	DMSO
NCRC23727	BBP00351	480-41-1	Naringenin	5-B6	272.253	10(μ)M	DMSO
NCRC23728	BBP00352	74892-45-8	5-Acetoxymatairesinol dimethyl ether	5-B7	444.474	10(μ)M	DMSO
NCRC23729	BBP00353	103553-98-6	3,7-O-Diacetylpinobanksin	5-B8	356.326	10(μ)M	DMSO
NCRC23730	BBP00354	62394-04-1	Cinnzeylanol	5-B9	384.464	10(μ)M	DMSO
NCRC23731	BBP00355	84-26-4	Rutaecarpine	5-B10	287.315	10(μ)M	DMSO
NCRC23732	BBP00356	2215-96-5	2,6-Dimethoxy-1-acetonylquinol	5-B11	226.226	10(μ)M	DMSO
NCRC23733	BBP00357	520-36-5	Apigenin	5-C2	270.237	10(μ)M	DMSO
NCRC23734	BBP00358	180961-65-3	Spiramilactone B	5-C3	330.418	10(μ)M	DMSO
NCRC23735	BBP00359	24164-13-4	Epitulipinolide	5-C4	290.354	10(μ)M	DMSO
NCRC23736	BBP00361	1909-91-7	Isocupressic acid	5-C5	320.466	10(μ)M	DMSO
NCRC23737	BBP00362	619326-74-8	Deoxycalyciphylline B	5-C6	341.487	10(μ)M	DMSO
NCRC23738	BBP00364	150-86-7	Phytol	5-C7	296.531	10(μ)M	DMSO
NCRC23739	BBP00365	18196-13-9	Naringenin-4',7-diacetate	5-C8	356.326	10(μ)M	DMSO
NCRC23740	BBP00366	486-28-2	Fraxinol	5-C9	222.194	10(μ)M	DMSO
NCRC23741	BBP00367	6545-99-9	Phebalosin	5-C10	258.269	10(μ)M	DMSO
NCRC23742	BBP00368	32971-25-8	(+)-Pinoresinol diacetate	5-C11	442.458	10(μ)M	DMSO
NCRC23743	BBP00370	484-12-8	Osthol	5-D2	244.286	10(μ)M	DMSO
NCRC23744	BBP00371	3682-04-0	Naringenin triacetate	5-D3	398.363	10(μ)M	DMSO
NCRC23745	BBP00372	154801-30-6	2-(2'-Hydroxytetracosanoylamino)-octadecane-1,3,4-triol	5-D4	684.128	10(μ)M	DMSO
NCRC23746	BBP00373	82508-36-9	Demethoxydeacetoxypseudolaric acid B	5-D5	376.4	10(μ)M	DMSO
NCRC23747	BBP00374	881388-87-0	Daphniyunnine A	5-D6	369.497	10(μ)M	DMSO
NCRC23748	BBP00375	99026-99-0	Limonexic acid	5-D7	502.51	10(μ)M	DMSO
NCRC23749	BBP00376	20248-08-2	13(18)-Oleanen-3-one	5-D8	424.702	10(μ)M	DMSO
NCRC23750	BBP00377	134476-74-7	Richenoic acid	5-D9	456.7	10(μ)M	DMSO
NCRC23751	BBP00378	96552-41-9	Cudraxanthone D	5-D10	410.46	10(μ)M	DMSO
NCRC23752	BBP00379	40433-82-7	Pinusolidic acid	5-D11	332.434	10(μ)M	DMSO
NCRC23753	BBP00380	520-28-5	Tectochrysin	5-E2	268.264	10(μ)M	DMSO
NCRC23754	BBP00381	559-74-0	Friedelin	5-E3	426.717	10(μ)M	DMSO
NCRC23755	BBP00382	501-98-4	trans-4-Hydroxycinnamic acid	5-E4	164.158	10(μ)M	DMSO
NCRC23756	BBP00383	16198-01-9	Catechin pentaacetate	5-E5	500.451	10(μ)M	DMSO
NCRC23757	BBP00384	53846-49-4	Flavaprin	5-E6	502.51	10(μ)M	DMSO
NCRC23758	BBP00385	488-44-8	Allitol	5-E7	182.172	10(μ)M	DMSO
NCRC23759	BBP00386	750649-07-1	Daphnilongeranin C	5-E8	355.471	10(μ)M	DMSO
NCRC23760	BBP00387	53377-61-0	1,3,5-Trihydroxy-4-	5-E9	312.317	10(μ)M	DMSO
NCRC23761	BBP00388	480-18-2	Taxifolin	5-E10	304.252	10(μ)M	DMSO
NCRC23762	BBP00389	114531-28-1	Spiramine A	5-E11	399.523	10(μ)M	DMSO
NCRC23763	BBP00390	127-40-2	Lutein	5-F2	568.871	10(μ)M	DMSO
NCRC23764	BBP00391	246868-97-3	Aglinin A	5-F3	490.715	10(μ)M	DMSO
NCRC23765	BBP00392	23971-42-8	Meranzin	5-F4	260.285	10(μ)M	DMSO
NCRC23766	BBP00393	15051-81-7	epi-Eudesmol	5-F5	222.366	10(μ)M	DMSO
NCRC23767	BBP00394	21040-64-2	Spiradine F	5-F6	399.523	10(μ)M	DMSO
NCRC23768	BBP00395	27832-84-4	Serratenediol diacetate	5-F7	526.79	10(μ)M	DMSO
NCRC23769	BBP00396	67253-01-4	Cabraleadiol	5-F8	460.732	10(μ)M	DMSO
NCRC23770	BBP00397	548-89-0	Gyrophoric acid	5-F9	468.41	10(μ)M	DMSO
NCRC23771	BBP00398	80604-16-6	2',5,6',7-Tetrahydroxyflavanone	5-F10	288.252	10(μ)M	DMSO

NCRC23772	BBP00399	64165-98-6	Echinoynethiophene A	5-F11	230.282	10(μ)M	DMSO
NCRC23773	BBP00400	185821-32-3	2,3-Dihydroxypterodontic acid	5-G2	266.333	10(μ)M	DMSO
NCRC23774	BBP00401	70110-60-0	5-Tricosyl-1,3-benzenediol	5-G3	432.722	10(μ)M	DMSO
NCRC23775	BBP00402	138-59-0	Shikimic acid	5-G4	174.151	10(μ)M	DMSO
NCRC23776	BBP00403	38302-15-7	Naringenin trimethyl ether	5-G5	314.333	10(μ)M	DMSO
NCRC23777	BBP00404	17245-25-9	Coumurrayin	5-G6	274.312	10(μ)M	DMSO
NCRC23778	BBP00405	1990-77-8	Syringaresinol diacetate	5-G7	502.51	10(μ)M	DMSO
NCRC23779	BBP00406	56795-51-8	Scopoletin acetate	5-G8	234.205	10(μ)M	DMSO
NCRC23780	BBP00407	34427-61-7	Ikshusterol	5-G9	430.706	10(μ)M	DMSO
NCRC23781	BBP00408	568-72-9	Tanshinone II	5-G10	294.344	10(μ)M	DMSO
NCRC23782	BBP00409	482-91-7	Aricine	5-G11	382.453	10(μ)M	DMSO
NCRC23783	BBP00410	No	2',6',7-Triacetoxy-5-hydroxyflavanone	5-H2	414.362	10(μ)M	DMSO
NCRC23784	BBP00411	109741-38-0	Murraol	5-H3	260.285	10(μ)M	DMSO
NCRC23785	BBP00412	530-55-2	2,6-Dimethoxy-1,4-benzoquinone	5-H4	168.147	10(μ)M	DMSO
NCRC23786	BBP00413	25532-45-0	Mayumbine	5-H5	352.427	10(μ)M	DMSO
NCRC23787	BBP00414	113557-95-2	Magnoloside A	5-H6	624.587	10(μ)M	DMSO
NCRC23788	BBP00415	17245-30-6	3',4',5',3,5,6,7-Heptamethoxyflavone	5-H7	432.421	10(μ)M	DMSO
NCRC23789	BBP00416	508-04-3	13(18)-Oleanen-3-ol	5-H8	426.717	10(μ)M	DMSO
NCRC23790	BBP00417	186374-63-0	enantio-7(11)-Eudesmen-4-ol	5-H9	222.366	10(μ)M	DMSO
NCRC23791	BBP00418	67560-68-3	Lariciresinol dimethyl ether	5-H10	388.454	10(μ)M	DMSO
NCRC23792	BBP00419	17391-09-2	Isocarapanaubine	5-H11	428.478	10(μ)M	DMSO
NCRC23793	BBP00420	221666-27-9	(+)-Conocarpan	6-A2	266.334	10(μ)M	DMSO
NCRC23794	BBP00421	1486-70-0	3-O-Methylquercetin	6-A3	316.262	10(μ)M	DMSO
NCRC23795	BBP00422	260397-58-8	Dihydroisotanshinone II	6-A4	278.302	10(μ)M	DMSO
NCRC23796	BBP00423	97399-93-4	Paniculidine A	6-A5	231.29	10(μ)M	DMSO
NCRC23797	BBP00424	16274-11-6	Kaempferol tetraacetate	6-A6	454.383	10(μ)M	DMSO
NCRC23798	BBP00425	520-26-3	Hesperidin	6-A7	610.561	10(μ)M	DMSO
NCRC23799	BBP00426	35825-57-1	Cryptotanshinone	6-A8	296.36	10(μ)M	DMSO
NCRC23800	BBP00427	160436-10-2	3',5,5',7-Tetrahydroxyflavanone	6-A9	288.252	10(μ)M	DMSO
NCRC23801	BBP00428	143724-69-0	Kaempferol 3,4',7-triacetate	6-A10	412.346	10(μ)M	DMSO
NCRC23802	BBP00429	92519-91-0	Viscidulin III	6-A11	346.288	10(μ)M	DMSO
NCRC23803	BBP00430	110-17-8	Fumaric acid	6-B2	116.072	10(μ)M	DMSO
NCRC23804	BBP00431	35833-69-3	Cabraleahydroxylactone	6-B3	416.636	10(μ)M	DMSO
NCRC23805	BBP00432	80604-17-7	2',5,6',7-Tetraacetoxyflavanone	6-B4	456.399	10(μ)M	DMSO
NCRC23806	BBP00433	69978-82-1	Isocostic acid	6-B5	234.334	10(μ)M	DMSO
NCRC23807	BBP00434	53851-13-1	Caboxine A	6-B6	398.452	10(μ)M	DMSO
NCRC23808	BBP00435	99946-04-0	Isoboonein	6-B7	170.206	10(μ)M	DMSO
NCRC23809	BBP00436	41514-64-1	Koaburaside monomethyl ether	6-B8	346.33	10(μ)M	DMSO
NCRC23810	BBP00437	20835-91-0	Stigmastane-3,5,6-triol	6-B9	448.721	10(μ)M	DMSO
NCRC23811	BBP00438	82508-37-0	Deacetylpseudolaric acid A	6-B10	346.417	10(μ)M	DMSO
NCRC23812	BBP00439	73354-15-1	Lariciresinol-4,4'-dimethyl ether-9-acetate	6-B11	430.491	10(μ)M	DMSO
NCRC23813	BBP00440	2202-01-9	Eichlerialactone	6-C2	430.62	10(μ)M	DMSO
NCRC23814	BBP00441	6426-43-3	Taraxasterol acetate	6-C3	468.754	10(μ)M	DMSO
NCRC23815	BBP00442	119400-87-2	Przewalskin	6-C4	272.382	10(μ)M	DMSO
NCRC23816	BBP00443	86838-54-2	2,2,5,5-Tetramethylcyclohexane-1,4-dione	6-C5	168.233	10(μ)M	DMSO
NCRC23817	BBP00444	96684-81-0	Viscidulin III tetraacetate	6-C6	514.435	10(μ)M	DMSO
NCRC23818	BBP00445	97399-95-6	Paniculidine C	6-C7	203.28	10(μ)M	DMSO
NCRC23819	BBP00446	21634-52-6	3',4',5',3,5,7,8-Heptamethoxyflavone	6-C8	432.421	10(μ)M	DMSO
NCRC23820	BBP00447	No	2alpha-Hydroxy pterodontic acid methyl ester	6-C9	264.36	10(μ)M	DMSO

NCRC23821	BBP00448	102054-39-7	Arctinol B	6-C10	264.363	10(μ)M	DMSO
NCRC23822	BBP00449	530-57-4	Syringic acid	6-C11	198.173	10(μ)M	DMSO
NCRC23823	BBP00450	630057-39-5	Neoprzewaquinone A	6-D2	556.604	10(μ)M	DMSO
NCRC23824	BBP00451	26315-07-1	22-Dehydroclerosterol	6-D3	410.675	10(μ)M	DMSO
NCRC23825	BBP00452	88546-96-7	Minumicrolin	6-D4	276.285	10(μ)M	DMSO
NCRC23826	BBP00453	No	3',5,5',7-Tetraacetoxyflavanone	6-D5	456.399	10(μ)M	DMSO
NCRC23827	BBP00454	1186496-68-3	11-Hydroxycodaphniphylline	6-D6	485.698	10(μ)M	DMSO
NCRC23828	BBP00455	71144-78-0	(E)-3-Acetoxy-5-methoxystilbene	6-D7	268.307	10(μ)M	DMSO
NCRC23829	BBP00457	13241-28-6	Chrysophanol 8-O-glucoside	6-D8	416.378	10(μ)M	DMSO
NCRC23830	BBP00458	No	No	6-D9	414.452	10(μ)M	DMSO
NCRC23831	BBP00459	56121-42-7	Asperglaucide	6-D10	444.522	10(μ)M	DMSO
NCRC23832	BBP00460	491-70-3	Luteolin	6-D11	286.236	10(μ)M	DMSO
NCRC23833	BBP00461	10388-48-4	Cycloart-25-ene-3,24-diol	6-E2	442.717	10(μ)M	DMSO
NCRC23834	BBP00462	13364-94-8	Exoticin	6-E3	462.447	10(μ)M	DMSO
NCRC23835	BBP00463	88478-44-8	Murraxocin	6-E4	304.338	10(μ)M	DMSO
NCRC23836	BBP00464	14004-35-4	Eucalyptin acetate	6-E5	368.38	10(μ)M	DMSO
NCRC23837	BBP00465	No	No	6-E6	468.453	10(μ)M	DMSO
NCRC23838	BBP00466	57576-31-5	(24S)-Cycloartane-3,24,25-triol 24,25-acetonide	6-E7	500.796	10(μ)M	DMSO
NCRC23839	BBP00467	97399-94-5	Paniculidine B	6-E8	233.306	10(μ)M	DMSO
NCRC23840	BBP00468	568-73-0	Tanshinone I	6-E9	276.286	10(μ)M	DMSO
NCRC23841	BBP00469	143815-99-0	22-Dehydroclerosterol glucoside	6-E10	572.816	10(μ)M	DMSO
NCRC23842	BBP00470	2308-85-2	Sitosteryl palmitate	6-E11	653.116	10(μ)M	DMSO
NCRC23843	BBP00471	99-76-3	Methyl 4-hydroxybenzoate	6-F2	152.147	10(μ)M	DMSO
NCRC23844	BBP00472	51650-59-0	Murrangatin diacetate	6-F3	360.358	10(μ)M	DMSO
NCRC23845	BBP00473	56319-04-1	(+)-Conocarpan acetate	6-F4	308.371	10(μ)M	DMSO
NCRC23846	BBP00474	51361-60-5	delta-Amyrin acetate	6-F5	468.754	10(μ)M	DMSO
NCRC23847	BBP00475	80366-15-0	2',3,5,6',7-Pentahydroxyflavanone	6-F6	304.252	10(μ)M	DMSO
NCRC23848	BBP00476	92519-95-4	Viscidulin I	6-F7	302.236	10(μ)M	DMSO
NCRC23849	BBP00477	58546-54-6	Gomisin A	6-F8	416.464	10(μ)M	DMSO
NCRC23850	BBP00478	83916-76-1	Isogomisin O	6-F9	416.464	10(μ)M	DMSO
NCRC23851	BBP00479	114916-00-6	No	6-F10	282.809	10(μ)M	DMSO
NCRC23852	BBP00480	121700-26-3	Moluccanin	6-F11	386.352	10(μ)M	DMSO
NCRC23853	BBP00482	131652-35-2	No	6-G2	294.73	10(μ)M	DMSO
NCRC23854	BBP00483	95360-22-8	Hainanmurpanin	6-G3	318.321	10(μ)M	DMSO
NCRC23855	BBP00484	1486-69-7	3-O-Methylquercetin tetraacetate	6-G4	484.409	10(μ)M	DMSO
NCRC23856	BBP00485	32179-18-3	1,10:4,5-Diepoxy-7(11)-germacren-8-one	6-G5	250.333	10(μ)M	DMSO
NCRC23857	BBP00486	5875-49-0	Meranzin hydrate	6-G6	278.3	10(μ)M	DMSO
NCRC23858	BBP00487	88585-86-8	Isomexoticin	6-G7	308.326	10(μ)M	DMSO
NCRC23859	BBP00488	No	Quercitrin hexaacetate	6-G8	700.597	10(μ)M	DMSO
NCRC23860	BBP00489	121700-27-4	Moluccanin diacetate	6-G9	470.425	10(μ)M	DMSO
NCRC23861	BBP00490	6665-67-4	4',5-Dihydroxyflavone	6-G10	254.238	10(μ)M	DMSO
NCRC23862	BBP00491	61276-17-3	Acteoside	6-G11	624.587	10(μ)M	DMSO
NCRC23863	BBP00493	202846-95-5	8-Hydroxy-3,5,7,3',4',5'-hexamethoxyflavone	6-H2	418.394	10(μ)M	DMSO
NCRC23864	BBP00494	105351-70-0	Tanshinlactone	6-H3	264.275	10(μ)M	DMSO
NCRC23865	BBP00495	92233-55-1	Syringin pentaacetate	6-H4	582.55	10(μ)M	DMSO
NCRC23866	BBP00496	99891-77-7	Isoboonein acetate	6-H5	212.242	10(μ)M	DMSO
NCRC23867	BBP00497	165074-00-0	6-Epidemethylesquirolin D	6-H6	348.433	10(μ)M	DMSO
NCRC23868	BBP00498	78876-52-5	No	6-H7	248.728	10(μ)M	DMSO
NCRC23869	BBP00499	26905-70-4	2-(4-Chloro-3-hydroxybuten-1-yl)-5-(pentadien-1,3-yl)thiophene	6-H8	248.728	10(μ)M	DMSO
NCRC23870	BBP00500	78876-53-6	No	6-H9	234.701	10(μ)M	DMSO
NCRC23871	BBP00501	14397-69-4	Jaceidin triacetate	6-H10	486.425	10(μ)M	DMSO

NCRC23872	BBP00502	5309-35-3	9,13-Epidioxy-8(14)-abieten-18-oic acid	6-H11	334.45	10(μ)M	DMSO
NCRC23873	BBP00503	17397-93-2	Tanshinone IIB	7-A2	310.344	10(μ)M	DMSO
NCRC23874	BBP00504	19626-92-7	Indole-3-acrylic acid methyl ester	7-A3	201.221	10(μ)M	DMSO
NCRC23875	BBP00505	95188-34-4	Sibiricin	7-A4	290.311	10(μ)M	DMSO
NCRC23876	BBP00506	79955-41-2	Quinovic acid 3-O-beta-D-glucoside	7-A5	648.824	10(μ)M	DMSO
NCRC23877	BBP00507	27003-73-2	(+)-Lariciresinol	7-A6	360.401	10(μ)M	DMSO
NCRC23878	BBP00508	54113-95-0	15-Hydroxydehydroabietic acid	7-A7	316.435	10(μ)M	DMSO
NCRC23879	BBP00509	53011-72-6	Murralongin	7-A8	258.269	10(μ)M	DMSO
NCRC23880	BBP00510	483-09-0	Isorauhimbine	7-A9	354.443	10(μ)M	DMSO
NCRC23881	BBP00511	53155-25-2	Euscaphic acid	7-A10	488.699	10(μ)M	DMSO
NCRC23882	BBP00513	87440-75-3	Viteralone	7-A11	242.27	10(μ)M	DMSO
NCRC23883	BBP00514	107870-05-3	Quinovin	7-B2	632.824	10(μ)M	DMSO
NCRC23884	BBP00515	189264-47-9	Cleroindicin F	7-B3	154.163	10(μ)M	DMSO
NCRC23885	BBP00516	102227-61-2	Epoxyparvinolide	7-B4	250.333	10(μ)M	DMSO
NCRC23886	BBP00517	885315-96-8	Kongensin A	7-B5	374.471	10(μ)M	DMSO
NCRC23887	BBP00518	59219-65-7	Darutoside	7-B6	484.623	10(μ)M	DMSO
NCRC23888	BBP00519	52589-11-4	Phellamurin	7-B7	518.51	10(μ)M	DMSO
NCRC23889	BBP00520	111441-88-4	Pinocembrin diacetate	7-B8	340.327	10(μ)M	DMSO
NCRC23890	BBP00521	87205-99-0	15,16-Dihydrotanshinone I	7-B9	278.302	10(μ)M	DMSO
NCRC23891	BBP00522	107389-91-3	Cleroindicin B	7-B10	158.195	10(μ)M	DMSO
NCRC23892	BBP00523	61263-49-8	Vitexilactone	7-B11	378.502	10(μ)M	DMSO
NCRC23893	BBP00524	289056-24-2	3,7,16-Trihydroxystigmast-5-ene	7-C2	446.705	10(μ)M	DMSO
NCRC23894	BBP00525	118916-57-7	Balanophonin	7-C3	356.369	10(μ)M	DMSO
NCRC23895	BBP00526	2880-49-1	Heraclenin	7-C4	286.279	10(μ)M	DMSO
NCRC23896	BBP00527	7437-55-0	8-Geranyloxypsoralen	7-C5	338.397	10(μ)M	DMSO
NCRC23897	BBP00528	53600-24-1	1-Hydroxyrutaecarpine	7-C6	303.315	10(μ)M	DMSO
NCRC23898	BBP00529	32383-76-9	Medicarpin	7-C7	270.28	10(μ)M	DMSO
NCRC23899	BBP00530	520-12-7	Pectolinarigenin	7-C8	314.289	10(μ)M	DMSO
NCRC23900	BBP00531	5940-00-1	Darutigenol	7-C9	322.482	10(μ)M	DMSO
NCRC23901	BBP00532	125072-68-6	No	7-C10	306.354	10(μ)M	DMSO
NCRC23902	BBP00533	No	Kongensin A acetate	7-C11	416.507	10(μ)M	DMSO
NCRC23903	BBP00534	124727-10-2	Quinovic acid 3-O-(6-deoxy-beta-D-glucopyranoside) 28-O-beta-D-glucopyranosyl ester	7-D2	794.965	10(μ)M	DMSO
NCRC23904	BBP00535	1447-88-7	Hispidulin	7-D3	300.263	10(μ)M	DMSO
NCRC23905	BBP00536	531-44-2	Scopolin	7-D4	354.309	10(μ)M	DMSO
NCRC23906	BBP00537	63543-52-2	24,25-Epoxy-dammar-20(21)-en-3-one	7-D5	440.701	10(μ)M	DMSO
NCRC23907	BBP00538	98755-25-0	Isothymusin	7-D6	330.289	10(μ)M	DMSO
NCRC23908	BBP00539	482-44-0	Imperatorin	7-D7	270.28	10(μ)M	DMSO
NCRC23909	BBP00540	581-31-7	Suberosin	7-D8	244.286	10(μ)M	DMSO
NCRC23910	BBP00541	160927-81-1	Villosin C	7-D9	360.401	10(μ)M	DMSO
NCRC23911	BBP00542	143839-02-5	2,24-Dihydroxyursolic acid	7-D10	488.699	10(μ)M	DMSO
NCRC23912	BBP00543	42438-89-1	Pinostilbene	7-D11	242.27	10(μ)M	DMSO
NCRC23913	BBP00544	55050-69-6	Dammar-20(21)-en-3,24,25-triol	7-E2	460.732	10(μ)M	DMSO
NCRC23914	BBP00674	37921-38-3	Cimifugin	7-E3	306.311	10(μ)M	DMSO
NCRC23915	BBP00546	145918-59-8	7,8,9,9-Tetradehydroisolariciresinol	7-E4	356.369	10(μ)M	DMSO
NCRC23916	BBP00547	99353-00-1	Methyl rosmarinate	7-E5	374.341	10(μ)M	DMSO
NCRC23917	BBP00548	464-98-2	Pseudotaraxasterol	7-E6	426.717	10(μ)M	DMSO
NCRC23918	BBP00549	483-66-9	Sphondin	7-E7	216.19	10(μ)M	DMSO
NCRC23919	BBP00550	189264-44-6	Cleroindicin C	7-E8	156.179	10(μ)M	DMSO
NCRC23920	BBP00551	559-70-6	beta-Amyrin	7-E9	426.717	10(μ)M	DMSO

NCRC23921	BBP00553	482-27-9	Isopimpinellin	7-E10	246.215	10(μ)M	DMSO
NCRC23922	BBP00554	56317-15-8	5,6,7,8-Tetramethoxycoumarin	7-E11	266.247	10(μ)M	DMSO
NCRC23923	BBP00555	1059-14-9	Taraxasterol	7-F2	426.717	10(μ)M	DMSO
NCRC23924	BBP00556	624-45-3	Methyl levulinate	7-F3	130.142	10(μ)M	DMSO
NCRC23925	BBP00557	1188282-01-0	16-O-Acetyldarutigenol	7-F4	364.519	10(μ)M	DMSO
NCRC23926	BBP00558	22318-10-1	Evodol	7-F5	484.495	10(μ)M	DMSO
NCRC23927	BBP00559	1188281-98-2	ent-14,16-Epoxy-8-pimarene-3,15-diol	7-F6	320.466	10(μ)M	DMSO
NCRC23928	BBP00560	545-47-1	Lupeol	7-F7	426.717	10(μ)M	DMSO
NCRC23929	BBP00561	225110-25-8	Falcarindiol	7-F8	260.371	10(μ)M	DMSO
NCRC23930	BBP00562	580-72-3	Matairesinol	7-F9	358.385	10(μ)M	DMSO
NCRC23931	BBP00563	17388-39-5	Swertiamarin	7-F10	374.34	10(μ)M	DMSO
NCRC23932	BBP00564	115458-73-6	Glaucin B	7-F11	528.548	10(μ)M	DMSO
NCRC23933	BBP00565	6035-49-0	6,7,8-Trimethoxycoumarin	7-G2	236.221	10(μ)M	DMSO
NCRC23934	BBP00566	142299-73-8	Teuvincenone H	7-G3	356.369	10(μ)M	DMSO
NCRC23935	BBP00567	514-07-8	Taraxerone	7-G4	424.702	10(μ)M	DMSO
NCRC23936	BBP00568	71933-54-5	Sarmentosin	7-G5	275.255	10(μ)M	DMSO
NCRC23937	BBP00570	72755-20-5	Odorine	7-G6	300.395	10(μ)M	DMSO
NCRC23938	BBP00571	298-81-7	Xanthotoxin	7-G7	216.19	10(μ)M	DMSO
NCRC23939	BBP00572	83905-81-1	12-Acetoxyabietic acid	7-G8	360.487	10(μ)M	DMSO
NCRC23940	BBP00573	989-61-7	Limonol	7-G9	472.527	10(μ)M	DMSO
NCRC23941	BBP00574	482-38-2	Kaempferitrin	7-G10	578.519	10(μ)M	DMSO
NCRC23942	BBP00575	52483-19-9	5-(Z-heptadec-8-enyl) resorcinol	7-G11	346.547	10(μ)M	DMSO
NCRC23943	BBP00576	17020-04-1	Bauerenol acetate	7-H2	468.754	10(μ)M	DMSO
NCRC23944	BBP00577	4439-68-3	Norkhellol	7-H3	232.189	10(μ)M	DMSO
NCRC23945	BBP00578	28217-60-9	Phlorin	7-H4	288.251	10(μ)M	DMSO
NCRC23946	BBP00579	72755-22-7	Odorinol	7-H5	316.395	10(μ)M	DMSO
NCRC23947	BBP00580	52992-82-2	Acetylisocupressic acid	7-H6	362.503	10(μ)M	DMSO
NCRC23948	BBP00581	151561-88-5	ent-3-Oxokauran-17-oic acid	7-H7	318.45	10(μ)M	DMSO
NCRC23949	BBP00582	165197-71-7	Cleroindicin E	7-H8	158.195	10(μ)M	DMSO
NCRC23950	BBP00584	189264-45-7	Cleroindicin D	7-H9	172.178	10(μ)M	DMSO
NCRC23951	BBP00585	14050-92-1	Methyl chanofruticosinate	7-H10	410.463	10(μ)M	DMSO
NCRC23952	BBP00586	57096-02-3	4'-Hydroxywogonin	7-H11	300.263	10(μ)M	DMSO
NCRC23953	BBP00587	24022-13-7	Agathadiol diacetate	8-A2	390.556	10(μ)M	DMSO
NCRC23954	BBP00588	150821-16-2	2,3,24-Trihydroxyolean-12-en-28-oic acid	8-A3	488.699	10(μ)M	DMSO
NCRC23955	BBP00589	36519-42-3	Serpentinine	8-A4	684.823	10(μ)M	DMSO
NCRC23956	BBP00590	144765-80-0	Brachynoside heptaacetate	8-A5	946.897	10(μ)M	DMSO
NCRC23957	BBP00591	524-15-2	gamma-Fagarine	8-A6	229.231	10(μ)M	DMSO
NCRC23958	BBP00593	89786-83-4	2,3,24-Trihydroxy-12-ursen-28-oic acid	8-A7	488.699	10(μ)M	DMSO
NCRC23959	BBP00594	82-57-5	Visnagin	8-A8	230.216	10(μ)M	DMSO
NCRC23960	BBP00595	439-89-4	Gentianine	8-A9	175.184	10(μ)M	DMSO
NCRC23961	BBP00596	480-44-4	Acacetin	8-A10	284.263	10(μ)M	DMSO
NCRC23962	BBP00597	32451-85-7	20(29)-Lupene-3,23-diol	8-A11	442.717	10(μ)M	DMSO
NCRC23963	BBP00598	103974-74-9	Esculentic acid	8-B2	488.699	10(μ)M	DMSO
NCRC23964	BBP00599	1188281-99-3	7-Hydroxydarutigenol	8-B3	338.482	10(μ)M	DMSO
NCRC23965	BBP00600	135683-73-7	ent-3-Oxokaurane-16,17-diol	8-B4	320.466	10(μ)M	DMSO
NCRC23966	BBP00601	121521-90-2	Salvianolic acid B	8-B5	718.614	10(μ)M	DMSO
NCRC23967	BBP00602	93675-85-5	Rengyol	8-B6	160.211	10(μ)M	DMSO
NCRC23968	BBP00603	65236-62-6	Dehydrocrenatidine	8-B7	254.284	10(μ)M	DMSO
NCRC23969	BBP00604	76948-72-6	Cleomiscosin A	8-B8	386.352	10(μ)M	DMSO
NCRC23970	BBP00605	31575-93-6	Heraclenol	8-B9	304.295	10(μ)M	DMSO
NCRC23971	BBP00606	131-07-7	Serpentine	8-B10	348.395	10(μ)M	DMSO

NCRC23972	BBP00607	508-02-1	Oleanolic acid	8-B11	456.7	10(μ)M	DMSO
NCRC23973	BBP00608	189322-69-8	3'-Methoxyrocaglamide	8-C2	535.585	10(μ)M	DMSO
NCRC23974	BBP00609	84573-16-0	Rocaglamide	8-C3	505.559	10(μ)M	DMSO
NCRC23975	BBP00610	11027-63-7	Agnuside	8-C4	466.435	10(μ)M	DMSO
NCRC23976	BBP00611	63543-53-3	24,25-Dihydroxydammar-20-en-3-one	8-C5	458.716	10(μ)M	DMSO
NCRC23977	BBP00612	49624-66-0	Angelicain	8-C6	292.284	10(μ)M	DMSO
NCRC23978	BBP00675	18642-44-9	Actein	8-C7	676.834	10(μ)M	DMSO
NCRC23979	BBP00614	4373-41-5	Crategolic acid	8-C8	472.7	10(μ)M	DMSO
NCRC23980	BBP00615	483-14-7	Tetrahydropalmatine	8-C9	355.428	10(μ)M	DMSO
NCRC23981	BBP00616	18110-86-6	5-Hydroxy-4-methoxycanthin-6-one	8-C10	266.252	10(μ)M	DMSO
NCRC23982	BBP00617	970-73-0	3,3',4',5,5',7-Hexahydroxyflavan	8-C11	306.267	10(μ)M	DMSO
NCRC23983	BBP00618	26791-73-1	Xanthatin	8-D2	246.302	10(μ)M	DMSO
NCRC23984	BBP00619	569-80-2	Penduletin	8-D3	344.315	10(μ)M	DMSO
NCRC23985	BBP00620	66465-24-5	Kaempferol 3-O-(6''-O-acetyl)glucoside-7-O-rhamnoside	8-D4	636.555	10(μ)M	DMSO
NCRC23986	BBP00621	52659-56-0	Kirenol	8-D5	338.482	10(μ)M	DMSO
NCRC23987	BBP00622	3464-66-2	1-Methoxycarbonyl-beta-carboline	8-D6	226.231	10(μ)M	DMSO
NCRC23988	BBP00623	41447-15-8	(-)-N-Methylsedridine	8-D7	157.253	10(μ)M	DMSO
NCRC23989	BBP00624	41447-16-9	(+)-N-Methylallosedridine	8-D8	157.253	10(μ)M	DMSO
NCRC23990	BBP00626	60761-79-7	ent-kauran-17,19-dioic acid	8-D9	334.4	10(μ)M	DMSO
NCRC23991	BBP00627	21887-01-4	Horminone	8-D10	332.434	10(μ)M	DMSO
NCRC23992	BBP00628	32207-10-6	Heraclenol 3'-O-beta-D-glucopyranoside	8-D11	466.435	10(μ)M	DMSO
NCRC23993	BBP00629	1188282-00-9	9-Hydroxydarutigenol	8-E2	338.482	10(μ)M	DMSO
NCRC23994	BBP00630	606125-07-9	Momor-cerebroside I	8-E3	844.253	10(μ)M	DMSO
NCRC23995	BBP00631	1782-65-6	Hardwickiic acid	8-E4	316.435	10(μ)M	DMSO
NCRC23996	BBP00632	60807-25-2	4-Methoxy-1-methoxycarbonyl-beta-carboline	8-E5	256.257	10(μ)M	DMSO
NCRC23997	BBP00633	5973-06-8	beta-Amyrin palmitate	8-E6	665.126	10(μ)M	DMSO
NCRC23998	BBP00634	171674-89-8	Nitidanin	8-E7	404.41	10(μ)M	DMSO
NCRC23999	BBP00635	2239-88-5	3,3'-Di-O-methylellagic acid	8-E8	330.246	10(μ)M	DMSO
NCRC24000	BBP00636	19103-54-9	Salvigenin	8-E9	328.316	10(μ)M	DMSO
NCRC24001	BBP00637	31008-18-1	Magnolin	8-E10	416.464	10(μ)M	DMSO
NCRC24002	BBP00638	26585-13-7	Dehydrocrenatine	8-E11	224.258	10(μ)M	DMSO
NCRC24003	BBP00640	52213-27-1	2,3-Dihydroxy-12-ursen-28-oic acid	8-F2	472.7	10(μ)M	DMSO
NCRC24004	BBP00641	340702-68-3	No	8-F3	852.275	10(μ)M	DMSO
NCRC24005	BBP00642	1617-49-8	2,3,8-Tri-O-methylellagic acid	8-F4	344.272	10(μ)M	DMSO
NCRC24006	BBP00676	131-03-3	alpha-Yohimbine	8-F5	354.443	10(μ)M	DMSO
NCRC24007	BBP00644	189322-67-6	3'-Hydroxyrocaglamide	8-F6	521.558	10(μ)M	DMSO
NCRC24008	BBP00645	549-84-8	beta-Yohimbine	8-F7	354.443	10(μ)M	DMSO
NCRC24009	BBP00648	82-02-0	Khellin	8-F8	260.242	10(μ)M	DMSO
NCRC24010	BBP00649	87592-77-6	Smyrindioloside	8-F9	424.399	10(μ)M	DMSO
NCRC24011	BBP00650	1188282-02-1	15,16-Di-O-acetyldarutoside	8-F10	568.696	10(μ)M	DMSO
NCRC24012	BBP00651	41653-72-9	Lacinilene C	8-F11	246.302	10(μ)M	DMSO
NCRC24013	BBP00652	57457-97-3	p-Menth-8-ene-1,2-diol	8-G2	170.249	10(μ)M	DMSO
NCRC24014	BBP00653	6610-56-6	Glochidiol	8-G3	442.717	10(μ)M	DMSO
NCRC24015	BBP00654	147059-46-9	Rocaglaol	8-G4	434.481	10(μ)M	DMSO
NCRC24016	BBP00655	390362-53-5	Buxbodine D	8-G5	426.678	10(μ)M	DMSO
NCRC24017	BBP00656	80151-89-9	Methyl demethoxycarbonylchanofruticosinate	8-G6	352.427	10(μ)M	DMSO
NCRC24018	BBP00657	927812-23-5	Glochicoccin D	8-G7	436.409	10(μ)M	DMSO

NCRC24019	BBP00658	111-20-6	Decanedioic acid	8-G8	202.248	10(μ)M	DMSO
NCRC24020	BBP00659	960589-81-5	ent-17-Hydroxykauran-3-one	8-G9	304.467	10(μ)M	DMSO
NCRC24021	BBP00661	153483-31-9	Xanthinin	8-G10	306.354	10(μ)M	DMSO
NCRC24022	BBP00662	100079-34-3	7-Hydroxy-2',5,8-trimethoxyflavanone	8-G11	330.332	10(μ)M	DMSO
NCRC24023	BBP00663	80554-58-1	1-Methyl-2-heptyl-4(1H)-quinolinone	8-H2	257.371	10(μ)M	DMSO
NCRC24024	BBP00664	22048-98-2	1-Methyl-2-pentyl-4(1H)-quinolinone	8-H3	229.317	10(μ)M	DMSO
NCRC24025	BBP00665	76792-94-4	3-Hydroxy-4',5,7-trimethoxyflavanone	8-H4	330.332	10(μ)M	DMSO
NCRC24026	BBP00666	1007125-14-5	3-(Hydroxymethyl)cyclopentanol	8-H5	116.158	10(μ)M	DMSO
NCRC24027	BBP00667	18110-87-7	4,5-Dimethoxycanthin-6-one	8-H6	280.278	10(μ)M	DMSO
NCRC24028	BBP00668	140631-27-2	12-Hydroxyjasmonic acid	8-H7	226.269	10(μ)M	DMSO
NCRC24029	BBP00669	193969-08-3	10-O-Vanilloylaucubin	8-H8	496.461	10(μ)M	DMSO
NCRC24030	BBP00670	24513-41-5	Monomethyl kolavate	8-H9	348.476	10(μ)M	DMSO
NCRC24031	BBP00672	73815-21-1	2,6-Dimethyl-7-octene-2,3,6-triol	8-H10	188.264	10(μ)M	DMSO
NCRC24032	BBP00673	19716-66-6	Pseudopalmatine	8-H11	352.404	10(μ)M	DMSO
NCRC24033	BBP00677	18524-94-2	Loganin	9-A2	390.382	10(μ)M	DMSO
NCRC24034	BBP00678	63399-37-1	3-Acetoxy-8(17),13E-labdadien-15-oic acid	9-A3	362.503	10(μ)M	DMSO
NCRC24035	BBP00680	23526-45-6	Vomifoliol	9-A4	224.296	10(μ)M	DMSO
NCRC24036	BBP00682	19833-13-7	Erythrodiol 3-palmitate	9-A5	681.126	10(μ)M	DMSO
NCRC24037	BBP00683	41756-77-8	Dihydrophaseic acid	9-A6	282.332	10(μ)M	DMSO
NCRC24038	BBP00684	131-12-4	Pimpinellin	9-A7	246.215	10(μ)M	DMSO
NCRC24039	BBP00685	582315-55-7	Betulin palmitate	9-A8	681.126	10(μ)M	DMSO
NCRC24040	BBP00686	113681-11-1	3-(hydroxymethyl)cyclopentanone	9-A9	114.142	10(μ)M	DMSO
NCRC24041	BBP00687	86654-26-4	7-Hydroxy-3-prenylcoumarin	9-A10	230.259	10(μ)M	DMSO
NCRC24042	BBP00689	488-82-4	D-arabinitol	9-A11	152.146	10(μ)M	DMSO
NCRC24043	BBP00690	1617-70-5	Lupenone	9-B2	424.702	10(μ)M	DMSO
NCRC24044	BBP00691	100234-62-6	Picrasidine J	9-B3	242.273	10(μ)M	DMSO
NCRC24045	BBP00692	155348-06-4	p-Menthan-1,3,8-triol	9-B4	188.264	10(μ)M	DMSO
NCRC24046	BBP00693	58493-71-3	ent-16beta,17-Isopropylidenedioxykaurane	9-B5	346.547	10(μ)M	DMSO
NCRC24047	BBP00694	64998-19-2	7alpha-Hydroxystigmasterol	9-B6	428.69	10(μ)M	DMSO
NCRC24048	BBP00866	831-61-8	Ethyl gallate	9-B7	198.173	10(μ)M	DMSO
NCRC24049	BBP00697	853267-91-1	Hythiemoside A	9-B8	526.659	10(μ)M	DMSO
NCRC24050	BBP00698	20649-43-8	Sinapaldehyde	9-B9	208.211	10(μ)M	DMSO
NCRC24051	BBP00699	28587-43-1	7-Geranyloxy-6-methoxycoumarin	9-B10	328.402	10(μ)M	DMSO
NCRC24052	BBP00700	100079-39-8	2',4'-Dihydroxy-2,3',6'-trimethoxychalcone	9-B11	330.332	10(μ)M	DMSO
NCRC24053	BBP00701	942-24-5	Methyl 3-indolecarboxylate	9-C2	175.184	10(μ)M	DMSO
NCRC24054	BBP00702	112503-87-4	Picrasidine S	9-C3	509.576	10(μ)M	DMSO
NCRC24055	BBP00703	638203-32-4	Phyllostadimer A	9-C4	810.837	10(μ)M	DMSO
NCRC24056	BBP00704	4382-56-3	Perakine	9-C5	350.411	10(μ)M	DMSO
NCRC24057	BBP00705	61012-31-5	Ferulamide	9-C6	193.199	10(μ)M	DMSO
NCRC24058	BBP00706	7559-04-8	alpha-Tocopherolquinone	9-C7	446.705	10(μ)M	DMSO
NCRC24059	BBP00707	1207181-63-2	Scutebata G	9-C8	679.755	10(μ)M	DMSO
NCRC24060	BBP00708	29424-96-2	4',7-Di-O-methylnaringenin	9-C9	300.306	10(μ)M	DMSO
NCRC24061	BBP00710	84812-00-0	3,4-Dimethoxyphenyl beta-D-glucoside	9-C10	316.304	10(μ)M	DMSO
NCRC24062	BBP00713	25522-33-2	Isoferulic acid	9-C11	194.184	10(μ)M	DMSO
NCRC24063	BBP00714	132915-47-0	9-Hydroxy-13E-labden-15-oic acid	9-D2	322.482	10(μ)M	DMSO
NCRC24064	BBP00716	268214-50-2	Coronalolide methyl ester	9-D3	496.678	10(μ)M	DMSO

NCRC24065	BBP00717	64790-68-7	Heraclenol acetonide	9-D4	344.358	10(μ)M	DMSO
NCRC24066	BBP00718	3301-61-9	ent-16beta,17-Dihydroxy-19-kauranoic acid	9-D5	336.466	10(μ)M	DMSO
NCRC24067	BBP00719	126176-79-2	Icariside E5	9-D6	522.542	10(μ)M	DMSO
NCRC24068	BBP00720	3489-06-3	Vineridine	9-D7	398.452	10(μ)M	DMSO
NCRC24069	BBP00721	132339-37-8	4-Epialyxialactone	9-D8	200.232	10(μ)M	DMSO
NCRC24070	BBP00722	70897-14-2	No	9-D9	340.37	10(μ)M	DMSO
NCRC24071	BBP00723	194145-29-4	Rivulobirin B	9-D10	432.336	10(μ)M	DMSO
NCRC24072	BBP00724	19013-07-1	Demethoxyencecalin	9-D11	202.249	10(μ)M	DMSO
NCRC24073	BBP00725	256925-92-5	Cimiracemoside C	9-E2	620.814	10(μ)M	DMSO
NCRC24074	BBP00726	53823-02-2	Onitin	9-E3	248.318	10(μ)M	DMSO
NCRC24075	BBP00727	63399-38-2	Alepterolic acid	9-E4	320.466	10(μ)M	DMSO
NCRC24076	BBP00729	884-35-5	Methyl syringate	9-E5	212.199	10(μ)M	DMSO
NCRC24077	BBP00730	170809-24-2	No	9-E6	318.45	10(μ)M	DMSO
NCRC24078	BBP00732	17650-84-9	Nicotiflorin	9-E7	594.518	10(μ)M	DMSO
NCRC24079	BBP00733	24480-45-3	Bryonolic acid	9-E8	456.7	10(μ)M	DMSO
NCRC24080	BBP00735	55732-36-0	No	9-E9	202.248	10(μ)M	DMSO
NCRC24081	BBP00736	94942-49-1	6-Methoxynaringenin	9-E10	302.279	10(μ)M	DMSO
NCRC24082	BBP00737	437-64-9	Genkwanin	9-E11	284.263	10(μ)M	DMSO
NCRC24083	BBP00738	121748-11-6	threo-1-C-Syringylglycerol	9-F2	244.241	10(μ)M	DMSO
NCRC24084	BBP00739	1207181-62-1	Scutebata F	9-F3	555.616	10(μ)M	DMSO
NCRC24085	BBP00740	2009-24-7	Xanthotoxol	9-F4	202.163	10(μ)M	DMSO
NCRC24086	BBP00741	425680-98-4	8-Hydroxy-5-O-beta-D-glucopyranosylpsoralen	9-F5	380.303	10(μ)M	DMSO
NCRC24087	BBP00742	299159-90-3	7-Hydroxy-6-methoxy-3-prenylcoumarin	9-F6	260.285	10(μ)M	DMSO
NCRC24088	BBP00743	132237-63-9	Alyxialactone	9-F7	200.232	10(μ)M	DMSO
NCRC24089	BBP00744	35761-54-7	Cabraleone	9-F8	458.716	10(μ)M	DMSO
NCRC24090	BBP00745	55481-86-2	Isosaxalin	9-F9	322.74	10(μ)M	DMSO
NCRC24091	BBP00746	22934-99-2	Desmethoxycentaureidin	9-F10	330.289	10(μ)M	DMSO
NCRC24092	BBP00747	471-69-2	Dipterocarpol	9-F11	442.717	10(μ)M	DMSO
NCRC24093	BBP00748	90-01-7	Salicyl alcohol	9-G2	124.137	10(μ)M	DMSO
NCRC24094	BBP00751	93-35-6	Umbelliferone	9-G3	162.142	10(μ)M	DMSO
NCRC24095	BBP00752	78510-19-7	N-trans-Feruloyl-3-methoxytyramine	9-G4	343.374	10(μ)M	DMSO
NCRC24096	BBP00753	87827-55-2	Alismol	9-G5	220.35	10(μ)M	DMSO
NCRC24097	BBP00754	58-96-8	Uridine	9-G6	244.201	10(μ)M	DMSO
NCRC24098	BBP00755	888482-17-5	11,12-De(methylenedioxy)danuphylline	9-G7	426.462	10(μ)M	DMSO
NCRC24099	BBP00756	68799-38-2	4-Oxobedfordiaic acid	9-G8	250.333	10(μ)M	DMSO
NCRC24100	BBP00867	145163-97-9	3beta-Hydroxyporiferast-5-en-7-one	9-G9	428.69	10(μ)M	DMSO
NCRC24101	BBP00758	310433-44-4	Echinophyllin C	9-G10	331.449	10(μ)M	DMSO
NCRC24102	BBP00759	111917-59-0	Adenanthin	9-G11	490.543	10(μ)M	DMSO
NCRC24103	BBP00760	84575-10-0	Cleomiscosin C	9-H2	416.378	10(μ)M	DMSO
NCRC24104	BBP00869	929881-46-9	Cucumegastigmane I	9-H3	240.296	10(μ)M	DMSO
NCRC24105	BBP00765	53319-52-1	Isogosferol	9-H4	286.279	10(μ)M	DMSO
NCRC24106	BBP00766	27994-11-2	Cimigenoside	9-H5	620.814	10(μ)M	DMSO
NCRC24107	BBP00767	149252-87-9	1-Dehydroxy-23-deoxojessic acid	9-H6	470.727	10(μ)M	DMSO
NCRC24108	BBP00768	85889-15-2	prim-O-Glucosylangelicain	9-H7	454.425	10(μ)M	DMSO
NCRC24109	BBP00769	123135-05-7	Uncaric acid	9-H8	488.699	10(μ)M	DMSO
NCRC24110	BBP00770	931114-98-6	(S,E)-Deca-2,9-dien-4,6-diyn-1,8-diol	9-H9	162.185	10(μ)M	DMSO
NCRC24111	BBP00772	35214-82-5	Neobyakangelicol	9-H10	316.305	10(μ)M	DMSO

NCRC24112	BBP00773	112408-71-6	2',5,7-Trihydroxy-8-methoxyflavanone	9-H11	302.279	10(μ)M	DMSO
NCRC24113	BBP00774	925932-10-1	Secaubrytriol	10-A2	490.715	10(μ)M	DMSO
NCRC24114	BBP00775	476-66-4	Ellagic acid	10-A3	302.193	10(μ)M	DMSO
NCRC24115	BBP00776	1207181-58-5	Scutebata B	10-A4	633.685	10(μ)M	DMSO
NCRC24116	BBP00777	765316-44-7	Heraclenol 3'-O-[beta-D-apiofuranosyl-(1-6)-beta-D-glucopyranoside]	10-A5	598.55	10(μ)M	DMSO
NCRC24117	BBP00778	102919-76-6	Scutellaric acid	10-A6	472.7	10(μ)M	DMSO
NCRC24118	BBP00779	38710-26-8	Seneciphylline N-oxide	10-A7	349.378	10(μ)M	DMSO
NCRC24119	BBP00780	2465-11-4	Stellasterol	10-A8	398.664	10(μ)M	DMSO
NCRC24120	BBP00781	268214-52-4	Coronalolic acid	10-A9	470.684	10(μ)M	DMSO
NCRC24121	BBP00782	108124-75-0	6-(beta-D-glucopyranosyloxy)-Salicylic acid methyl ester	10-A10	330.287	10(μ)M	DMSO
NCRC24122	BBP00784	117591-81-8	Coronarin E	10-A11	284.436	10(μ)M	DMSO
NCRC24123	BBP00785	205115-75-9	Lansiumarin C	10-B2	354.396	10(μ)M	DMSO
NCRC24124	BBP00787	114567-47-4	Ganoderiol F	10-B3	454.684	10(μ)M	DMSO
NCRC24125	BBP00788	72021-23-9	Henryoside	10-B4	584.523	10(μ)M	DMSO
NCRC24126	BBP00790	6871-44-9	Echitamine	10-B5	385.477	10(μ)M	DMSO
NCRC24127	BBP00791	480-56-8	Lecanoric acid	10-B6	318.278	10(μ)M	DMSO
NCRC24128	BBP00792	18747-42-7	Methylisopelletierine	10-B7	155.237	10(μ)M	DMSO
NCRC24129	BBP00793	10351-88-9	Phyllanthin	10-B8	418.523	10(μ)M	DMSO
NCRC24130	BBP00794	6519-26-2	(16R)-Dihydrositsirikine	10-B9	356.459	10(μ)M	DMSO
NCRC24131	BBP00795	147-85-3	Proline	10-B10	115.13	10(μ)M	DMSO
NCRC24132	BBP00796	56755-22-7	Phenylalanine betaine	10-B11	207.269	10(μ)M	DMSO
NCRC24133	BBP00797	104759-35-5	Ganoderic acid S	10-C2	452.669	10(μ)M	DMSO
NCRC24134	BBP00798	58115-31-4	Aurantiamide	10-C3	402.486	10(μ)M	DMSO
NCRC24135	BBP00799	106623-23-8	3,10-Dihydroxy-5,11-dielmenthadiene-4,9-dione	10-C4	332.434	10(μ)M	DMSO
NCRC24136	BBP00800	113626-76-9	Stigmast-4-ene-3,6-diol	10-C5	430.706	10(μ)M	DMSO
NCRC24137	BBP00801	50656-77-4	Niranthin	10-C6	432.507	10(μ)M	DMSO
NCRC24138	BBP00802	2571-22-4	Tutin	10-C7	294.3	10(μ)M	DMSO
NCRC24139	BBP00803	6832-60-6	Imbricatolic acid	10-C8	322.482	10(μ)M	DMSO
NCRC24140	BBP00804	151121-39-0	(E)-8-(6-Hydroperoxy-3,7-dimethylocta-2,7-dienyloxy)psoralen	10-C9	370.396	10(μ)M	DMSO
NCRC24141	BBP00805	33676-00-5	Hypophyllanthin	10-C10	430.491	10(μ)M	DMSO
NCRC24142	BBP00806	268214-51-3	Coronalolide	10-C11	482.651	10(μ)M	DMSO
NCRC24143	BBP00808	106518-63-2	Ganodermanontriol	10-D2	472.7	10(μ)M	DMSO
NCRC24144	BBP00809	176665-78-4	Ducheside A	10-D3	448.334	10(μ)M	DMSO
NCRC24145	BBP00810	283174-18-5	11beta-Hydroxycedrelone	10-D4	438.513	10(μ)M	DMSO
NCRC24146	BBP00811	138-52-3	Salicin	10-D5	286.278	10(μ)M	DMSO
NCRC24147	BBP00812	194027-11-7	6,19-Dihydroxyurs-12-en-3-oxo-28-oic acid	10-D6	486.683	10(μ)M	DMSO
NCRC24148	BBP00813	139-85-5	3,4-Dihydroxybenzaldehyde	10-D7	138.121	10(μ)M	DMSO
NCRC24149	BBP00814	390362-51-3	Buxbodine B	10-D8	399.609	10(μ)M	DMSO
NCRC24150	BBP00816	20649-42-7	Coniferaldehyde	10-D9	178.185	10(μ)M	DMSO
NCRC24151	BBP00817	35481-77-7	Corianin	10-D10	294.3	10(μ)M	DMSO
NCRC24152	BBP00818	91653-75-7	Coriatin	10-D11	296.316	10(μ)M	DMSO
NCRC24153	BBP00819	481-74-3	Chrysophanol	10-E2	254.238	10(μ)M	DMSO
NCRC24154	BBP00821	173075-45-1	Ganoderic acid DM	10-E3	468.668	10(μ)M	DMSO
NCRC24155	BBP00822	518-82-1	Emodin	10-E4	270.237	10(μ)M	DMSO
NCRC24156	BBP00823	87797-84-0	6beta-Hydroxyipolamiide	10-E5	422.381	10(μ)M	DMSO
NCRC24157	BBP00824	1207181-61-0	Scutebata E	10-E6	520.612	10(μ)M	DMSO
NCRC24158	BBP00825	67650-47-9	Clerodermic acid methyl ester	10-E7	346.461	10(μ)M	DMSO

NCRC24159	BBP00826	905929-95-5	Scutebarbatine B	10-E8	557.634	10(μ)M	DMSO
NCRC24160	BBP00827	925932-08-7	Secaubryenol	10-E9	456.7	10(μ)M	DMSO
NCRC24161	BBP00828	104700-97-2	Ganoderol A	10-E10	438.685	10(μ)M	DMSO
NCRC24162	BBP00830	480-81-9	Seneciphylline	10-E11	333.379	10(μ)M	DMSO
NCRC24163	BBP00832	167875-39-0	Rubelloside B	10-F2	794.965	10(μ)M	DMSO
NCRC24164	BBP00833	942582-15-2	Walsuronoid B	10-F3	438.513	10(μ)M	DMSO
NCRC24165	BBP00834	89498-91-9	Picrasinol B	10-F4	392.486	10(μ)M	DMSO
NCRC24166	BBP00835	No	Ducheside A pentaacetate	10-F5	658.517	10(μ)M	DMSO
NCRC24167	BBP00836	16929-95-6	Tyromycic acid	10-F6	452.669	10(μ)M	DMSO
NCRC24168	BBP00837	No	15-Ethoxychinensine A	10-F7	344.488	10(μ)M	DMSO
NCRC24169	BBP00839	113808-03-0	Picrasidine T	10-F8	481.522	10(μ)M	DMSO
NCRC24170	BBP00840	23141-25-5	Strictosamide	10-F9	498.525	10(μ)M	DMSO
NCRC24171	BBP00841	41983-91-9	Glabranin	10-F10	324.37	10(μ)M	DMSO
NCRC24172	BBP00842	237407-59-9	Rivulobirin E	10-F11	590.574	10(μ)M	DMSO
NCRC24173	BBP00843	83015-88-7	1,6-Dioxaspiro[4.5]decan-2-methanol	10-G2	172.222	10(μ)M	DMSO
NCRC24174	BBP00844	No	No	10-G3	214.258	10(μ)M	DMSO
NCRC24175	BBP00845	76497-69-3	15,16-Dinor-8(17),11-labdadien-13-one	10-G4	260.414	10(μ)M	DMSO
NCRC24176	BBP00846	2957-21-3	Sakuranetin	10-G5	286.279	10(μ)M	DMSO
NCRC24177	BBP00847	160598-92-5	Villosin	10-G6	300.435	10(μ)M	DMSO
NCRC24178	BBP00848	107900-76-5	Ganodermanondiol	10-G7	456.7	10(μ)M	DMSO
NCRC24179	BBP00849	25018-67-1	1,2:4,5-Di-O-isopropylidene-beta-D-fructopyranose	10-G8	260.284	10(μ)M	DMSO
NCRC24180	BBP00850	855-96-9	Eupatorin	10-G9	344.315	10(μ)M	DMSO
NCRC24181	BBP00851	215609-93-1	23-deoxojessic acid	10-G10	486.726	10(μ)M	DMSO
NCRC24182	BBP00852	70110-61-1	5-Pentacosylresorcinol	10-G11	460.775	10(μ)M	DMSO
NCRC24183	BBP00853	176520-13-1	Scutebarbatine A	10-H2	558.622	10(μ)M	DMSO
NCRC24184	BBP00854	481-73-2	Citreorosein	10-H3	286.236	10(μ)M	DMSO
NCRC24185	BBP00868	97465-82-2	Dehydrodiconiferyl alcohol	10-H4	358.385	10(μ)M	DMSO
NCRC24186	BBP00856	19417-00-6	Isohyenanchin	10-H5	312.315	10(μ)M	DMSO
NCRC24187	BBP00857	152685-91-1	Cimiside B	10-H6	752.928	10(μ)M	DMSO
NCRC24188	BBP00858	213905-35-2	11(S),16(R)-Dihydroxyoctadeca-9Z,17-diene-12,14-diyn-1-yl acetate	10-H7	332.434	10(μ)M	DMSO
NCRC24189	BBP00859	211238-60-7	9(Z),17-Octadecadiene-12,14-diyne-1,11,16-triol	10-H8	290.397	10(μ)M	DMSO
NCRC24190	BBP00861	2749-28-2	2-O-Acetyltutin	10-H9	336.337	10(μ)M	DMSO
NCRC24191	BBP00862	37831-70-2	Phaseollidin	10-H10	324.37	10(μ)M	DMSO
NCRC24192	BBP00864	99-50-3	3,4-Dihydroxybenzoic acid	10-H11	154.12	10(μ)M	DMSO
NCRC24193	BBP00870	607-80-7	Sesamin	11-A2	354.353	10(μ)M	DMSO
NCRC24194	BBP00871	88191-14-4	3,5,9-Trihydroxyergosta-7,22-dien-6-one	11-A3	444.647	10(μ)M	DMSO
NCRC24195	BBP00872	21302-79-4	Ceanothic acid	11-A4	486.683	10(μ)M	DMSO
NCRC24196	BBP00873	14858-07-2	3,5-Dihydroxyergosta-7,22-dien-6-one	11-A5	428.647	10(μ)M	DMSO
NCRC24197	BBP00874	119188-38-4	Coronarin B	11-A6	334.45	10(μ)M	DMSO
NCRC24198	BBP00875	1399-49-1	Globularin	11-A7	492.473	10(μ)M	DMSO
NCRC24199	BBP00876	2415-24-9	Catalpol	11-A8	362.329	10(μ)M	DMSO
NCRC24200	BBP00877	516-37-0	Cerevisterol	11-A9	430.663	10(μ)M	DMSO
NCRC24201	BBP00878	104700-96-1	Ganoderol B	11-A10	440.701	10(μ)M	DMSO
NCRC24202	BBP00879	86989-18-6	No	11-A11	378.375	10(μ)M	DMSO
NCRC24203	BBP00880	No	6-O-Acetylcoriatin	11-B2	338.352	10(μ)M	DMSO

NCRC24204	BBP00881	131984-82-2	3,6,19-Trihydroxy-23-oxo-12-ursen-28-oic acid	11-B3	502.683	10(μ)M	DMSO
NCRC24205	BBP00882	155759-02-7	Hierochin D	11-B4	344.358	10(μ)M	DMSO
NCRC24206	BBP00883	119188-33-9	Coronarin A	11-B5	300.435	10(μ)M	DMSO
NCRC24207	BBP00884	18749-71-8	Sitoindoside I	11-B6	815.256	10(μ)M	DMSO
NCRC24208	BBP00885	288248-46-4	Phlorigidoside B	11-B7	464.418	10(μ)M	DMSO
NCRC24209	BBP00886	3681-93-4	Vitexin	11-B8	432.378	10(μ)M	DMSO
NCRC24210	BBP00887	71035-06-8	Griselinoside	11-B9	432.376	10(μ)M	DMSO
NCRC24211	BBP00888	465-74-7	Quinovic acid	11-B10	486.683	10(μ)M	DMSO
NCRC24212	BBP00889	17306-46-6	Rhoifolin	11-B11	578.519	10(μ)M	DMSO
NCRC24213	BBP00890	572-30-5	Avicularin	11-C2	434.35	10(μ)M	DMSO
NCRC24214	BBP00891	60263-06-1	Jacaranone ethyl ester	11-C3	196.2	10(μ)M	DMSO
NCRC24215	BBP00893	260393-05-3	Zamanic acid	11-C4	618.842	10(μ)M	DMSO
NCRC24216	BBP00894	80489-65-2	Rubifolic acid	11-C5	472.7	10(μ)M	DMSO
NCRC24217	BBP00895	120722-04-5	12alpha-Hydroxyevodol	11-C6	500.495	10(μ)M	DMSO
NCRC24218	BBP00896	125537-92-0	3',5,5',7-Tetrahydroxy-4',6-dimethoxyflavone	11-C7	346.288	10(μ)M	DMSO
NCRC24219	BBP00897	165338-27-2	Hemiphroside A	11-C8	668.64	10(μ)M	DMSO
NCRC24220	BBP00898	No	No	11-C9	416.4	10(μ)M	DMSO
NCRC24221	BBP00899	604-99-9	Tombozine	11-C10	294.391	10(μ)M	DMSO
NCRC24222	BBP00900	931116-24-4	(R,E)-Deca-2-ene-4,6-diyn-1,8-diol	11-C11	164.201	10(μ)M	DMSO
NCRC24223	BBP00902	154461-65-1	Sinapaldehyde glucoside	11-D2	370.351	10(μ)M	DMSO
NCRC24224	BBP00904	63034-29-7	Hexacosyl (E)-ferulate	11-D3	558.875	10(μ)M	DMSO
NCRC24225	BBP00905	165338-28-3	Hemiphroside B	11-D4	682.623	10(μ)M	DMSO
NCRC24226	BBP00907	1093207-99-8	3-Hydroxy-4,15-dinor-1(5)-xanthen-12,8-olide	11-D5	224.296	10(μ)M	DMSO
NCRC24227	BBP00908	3301-49-3	Kumatakenin	11-D6	314.289	10(μ)M	DMSO
NCRC24228	BBP00909	1207181-57-4	Scutebata A	11-D7	632.697	10(μ)M	DMSO
NCRC24229	BBP00910	No	2,6-Di-O-acetylisohyenanchin	11-D8	396.388	10(μ)M	DMSO
NCRC24230	BBP00912	51225-30-0	Wighteone	11-D9	338.354	10(μ)M	DMSO
NCRC24231	BBP00913	261768-88-1	3,19-Dihydroxy-6,23-dioxo-12-ursen-28-oic acid	11-D10	500.667	10(μ)M	DMSO
NCRC24232	BBP00914	47418-70-2	N-Methylsarpagine methosalt	11-D11	339.451	10(μ)M	DMSO
NCRC24233	BBP00915	890317-92-7	erythro-Guaiacylglycerol beta-coniferyl ether	11-E2	376.4	10(μ)M	DMSO
NCRC24234	BBP00918	1254-85-9	Cedrelone	11-E3	422.513	10(μ)M	DMSO
NCRC24235	BBP00920	511-01-3	alpha-Onocerol	11-E4	442.717	10(μ)M	DMSO
NCRC24236	BBP00921	112137-81-2	Ikshusterol 3-O-beta-D-glucopyranoside	11-E5	592.847	10(μ)M	DMSO
NCRC24237	BBP00922	849245-34-7	Chinensine B	11-E6	316.435	10(μ)M	DMSO
NCRC24238	BBP00923	104055-76-7	Quinovic acid 3-O-.alpha.-L-rhamnopyranoside	11-E7	632.824	10(μ)M	DMSO
NCRC24239	BBP00924	115783-35-2	15,16-Dihydro-15-methoxy-16-oxohardwickiic acid	11-E8	362.46	10(μ)M	DMSO
NCRC24240	BBP00925	No	15-Methoxychinensine A	11-E9	330.461	10(μ)M	DMSO
NCRC24241	BBP00927	486-66-8	Daidzein	11-E10	254.238	10(μ)M	DMSO
NCRC24242	BBP00928	108887-44-1	13-O-Acetylcorianin	11-E11	336.337	10(μ)M	DMSO
NCRC24243	BBP00929	66648-43-9	N-trans-Feruloyltyramine	11-F2	313.348	10(μ)M	DMSO
NCRC24244	BBP00930	54087-32-0	No	11-F3	274.269	10(μ)M	DMSO
NCRC24245	BBP00931	869799-76-8	threo-Guaiacylglycerol beta-coniferyl ether	11-F4	376.4	10(μ)M	DMSO
NCRC24246	BBP00932	7562-61-0	Usnic acid	11-F5	344.315	10(μ)M	DMSO
NCRC24247	BBP00934	132302-25-1	No	11-F6	1060.95	10(μ)M	DMSO
NCRC24248	BBP00935	61665-08-5	11-Methoxyuncarine C	11-F7	398.452	10(μ)M	DMSO

NCRC24249	BBP00937	1021945-29-8	3-O-Methyltirotundin	11-F8	366.449	10(μ)M	DMSO
NCRC24250	BBP00938	4429-63-4	Tabersonine	11-F9	336.427	10(μ)M	DMSO
NCRC24251	BBP00939	1156-78-1	2'-Hydroxygenistein	11-F10	286.236	10(μ)M	DMSO
NCRC24252	BBP00940	145400-03-9	Homalomenol A	11-F11	238.366	10(μ)M	DMSO
NCRC24253	BBP00942	97399-91-2	Aristolactam AIIIa	11-G2	281.263	10(μ)M	DMSO
NCRC24254	BBP00943	13902-62-0	Oplodiol	11-G3	238.366	10(μ)M	DMSO
NCRC24255	BBP00944	No	No	11-G4	1004.93	10(μ)M	DMSO
NCRC24256	BBP00945	256445-68-8	Schleicheol 2	11-G5	444.733	10(μ)M	DMSO
NCRC24257	BBP00946	28449-62-9	Tomentin	11-G6	222.194	10(μ)M	DMSO
NCRC24258	BBP00947	61597-55-5	15,16-Epoxy-12R-hydroxylabda-8(17),13(16),14-triene	11-G7	302.451	10(μ)M	DMSO
NCRC24259	BBP00948	125124-68-7	26-Nor-8-oxo-alpha-onocerin	11-G8	444.69	10(μ)M	DMSO
NCRC24260	BBP00949	38953-85-4	Isovitexin	11-G9	432.378	10(μ)M	DMSO
NCRC24261	BBP00950	56377-67-4	Tirotundin	11-G10	352.422	10(μ)M	DMSO
NCRC24262	BBP00951	27773-39-3	Ervamycine	11-G11	366.453	10(μ)M	DMSO
NCRC24263	BBP00952	182132-59-8	Quinovic acid 3-O-(3',4'-O-isopropylidene)-beta-D-fucopyranoside	11-H2	672.888	10(μ)M	DMSO
NCRC24264	BBP00953	63807-85-2	Erythrinin C	11-H3	354.353	10(μ)M	DMSO
NCRC24265	BBP00954	90341-45-0	Seneciphyllinine	11-H4	375.416	10(μ)M	DMSO
NCRC24266	BBP01054	60547-63-9	1-Acetyltagitinin A	11-H5	410.458	10(μ)M	DMSO
NCRC24267	BBP00956	66900-93-4	1,2-O-Isopropylidene-beta-D-fructopyranose	11-H6	341.401	10(μ)M	DMSO
NCRC24268	BBP00957	30413-84-4	Corydalmine	11-H7	256.381	10(μ)M	DMSO
NCRC24269	BBP00958	99933-32-1	Bullatantriol	11-H8	362.417	10(μ)M	DMSO
NCRC24270	BBP00959	110382-37-1	3-O-Methyltagitinin F	11-H9	424.49	10(μ)M	DMSO
NCRC24271	BBP00960	No	19(S)-Acetoxy-11-methoxytabersonine	11-H10	424.702	10(μ)M	DMSO
NCRC24272	BBP00961	508-09-8	Glutinone	11-H11	325.358	10(μ)M	DMSO
NCRC24273	BBP00962	86537-66-8	N-Methylcalycinine	12-A2	538.458	10(μ)M	DMSO
NCRC24274	BBP00963	1617-53-4	Amentoflavone	12-A3	268.35	10(μ)M	DMSO
NCRC24275	BBP00964	133453-58-4	Heudelotinone	12-A4	300.435	10(μ)M	DMSO
NCRC24276	BBP00965	162762-93-8	Yunnancoronarin A	12-A5	474.588	10(μ)M	DMSO
NCRC24277	BBP00966	1207181-35-8	Psidial A	12-A6	472.7	10(μ)M	DMSO
NCRC24278	BBP00967	93372-87-3	20-Hydroxy-3-oxo-28-lupanoic acid	12-A7	352.337	10(μ)M	DMSO
NCRC24279	BBP00968	221150-19-2	Erysubin B	12-A8	320.385	10(μ)M	DMSO
NCRC24280	BBP00969	28645-27-4	Meloscandonine	12-A9	274.398	10(μ)M	DMSO
NCRC24281	BBP00970	769140-74-1	13-Methyl-8,11,13-podocarpatriene-3,12-diol	12-A10	154.163	10(μ)M	DMSO
NCRC24282	BBP00971	10597-60-1	2-(3,4-Dihydroxyphenyl)ethanol	12-A11	440.744	10(μ)M	DMSO
NCRC24283	BBP00972	1449-09-8	24-Methylenecycloartan-3-ol	12-B2	504.699	10(μ)M	DMSO
NCRC24284	BBP00973	91095-51-1	3,6,19,23-Tetrahydroxy-12-ursen-28-oic acid	12-B3	228.2	10(μ)M	DMSO
NCRC24285	BBP00974	529-61-3	Euxanthone	12-B4	252.308	10(μ)M	DMSO
NCRC24286	BBP00975	17676-24-3	trans-Hinokiresinol	12-B5	529.579	10(μ)M	DMSO
NCRC24287	BBP00979	1207181-59-6	Scutebata C	12-B6	484.753	10(μ)M	DMSO
NCRC24288	BBP00980	1260-05-5	Phlegmanol C	12-B7	464.55	10(μ)M	DMSO
NCRC24289	BBP00981	659747-28-1	Isogarciniaxanthone E	12-B8	464.6	10(μ)M	DMSO
NCRC24290	BBP00982	14031-37-9	Squalene-2,3-diol	12-B9	444.733	10(μ)M	DMSO
NCRC24291	BBP00983	51276-34-7	2,6-Dimethyl-3,7-octadiene-2,6-diol	12-B10	170.249	10(μ)M	DMSO
NCRC24292	BBP00984	160623-47-2	1,4,6-Trihydroxy-5-methoxy-7-prenylxanthone	12-B11	342.343	10(μ)M	DMSO
NCRC24293	BBP00985	23417-92-7	7-Isopentenyloxy-gamma-fagarine	12-C2	313.348	10(μ)M	DMSO

NCRC24294	BBP00987	162473-22-5	Subelliptenone G	12-C3	244.2	10(μ)M	DMSO
NCRC24295	BBP00989	16503-32-5	Brevilin A	12-C4	346.417	10(μ)M	DMSO
NCRC24296	BBP00990	103744-84-9	Rehmaglutin D	12-C5	220.65	10(μ)M	DMSO
NCRC24297	BBP00991	25368-01-8	Litseglutine B	12-C6	341.401	10(μ)M	DMSO
NCRC24298	BBP00992	14464-90-5	Lyoniresinol	12-C7	420.453	10(μ)M	DMSO
NCRC24299	BBP00993	2169-44-0	Lauroscholtzine	12-C8	341.401	10(μ)M	DMSO
NCRC24300	BBP00994	1449-06-5	21-Episerratenediol	12-C9	442.717	10(μ)M	DMSO
NCRC24301	BBP00995	83324-51-0	8(17),13-Labdadien-15,16-olide	12-C10	302.451	10(μ)M	DMSO
NCRC24302	BBP00996	136055-64-6	No	12-C11	506.499	10(μ)M	DMSO
NCRC24303	BBP00997	1013-69-0	Noreugenin	12-D2	192.168	10(μ)M	DMSO
NCRC24304	BBP00998	130837-92-2	7-Oxohinokinin	12-D3	368.337	10(μ)M	DMSO
NCRC24305	BBP01000	3155-43-9	1,18-Octadecanediol	12-D4	286.493	10(μ)M	DMSO
NCRC24306	BBP01001	216011-55-1	15,16-Epoxy-12S-hydroxylabda-8(17),13(16),14-triene	12-D5	302.451	10(μ)M	DMSO
NCRC24307	BBP01002	2034-74-4	7-Oxo-beta-sitosterol	12-D6	428.69	10(μ)M	DMSO
NCRC24308	BBP01003	156767-69-0	3beta-Hydroxyergost-5-en-7-one	12-D7	414.664	10(μ)M	DMSO
NCRC24309	BBP01004	74161-25-4	2,3-Dehydrokievitone	12-D8	354.353	10(μ)M	DMSO
NCRC24310	BBP01005	41743-56-0	Luteone	12-D9	354.353	10(μ)M	DMSO
NCRC24311	BBP01006	5890-28-8	Cassythicine	12-D10	325.358	10(μ)M	DMSO
NCRC24312	BBP01007	57498-96-1	Carpachromene	12-D11	336.338	10(μ)M	DMSO
NCRC24313	BBP01008	476-70-0	Boldine	12-E2	327.374	10(μ)M	DMSO
NCRC24314	BBP01009	522-11-2	Evoxine	12-E3	347.362	10(μ)M	DMSO
NCRC24315	BBP01010	776325-66-7	1,4,5,6-Tetrahydroxy-7,8-diprenylxanthone	12-E4	396.433	10(μ)M	DMSO
NCRC24316	BBP01011	56218-46-3	Methyl lycernuate A	12-E5	486.726	10(μ)M	DMSO
NCRC24317	BBP01012	50838-11-4	14,15-Didehydroisoeburnamine	12-E6	294.391	10(μ)M	DMSO
NCRC24318	BBP01013	22255-13-6	Guaijaverin	12-E7	434.35	10(μ)M	DMSO
NCRC24319	BBP01014	22149-28-6	11-Hydroxytabersonine	12-E8	352.427	10(μ)M	DMSO
NCRC24320	BBP01015	57576-41-7	Norcepharadione B	12-E9	307.3	10(μ)M	DMSO
NCRC24321	BBP01016	41530-90-9	2,6,16-Kauranetriol	12-E10	322.482	10(μ)M	DMSO
NCRC24322	BBP01017	53948-09-7	Aristolactam BII	12-E11	279.29	10(μ)M	DMSO
NCRC24323	BBP01020	112501-42-5	Aristolactam FI	12-F2	265.263	10(μ)M	DMSO
NCRC24324	BBP01022	112219-48-4	14,15-Didehydrovincamenine	12-F3	276.375	10(μ)M	DMSO
NCRC24325	BBP01023	219861-73-1	2,2',3'-Trihydroxy-4,6-dimethoxybenzophenone	12-F4	290.268	10(μ)M	DMSO
NCRC24326	BBP01025	97456-49-0	Cycloart-22-ene-3,25-diol	12-F5	442.717	10(μ)M	DMSO
NCRC24327	BBP01026	50656-92-3	Vandrikidine	12-F6	382.453	10(μ)M	DMSO
NCRC24328	BBP01027	61135-91-9	3,9-Dihydroxypterocarpan	12-F7	256.253	10(μ)M	DMSO
NCRC24329	BBP01028	36151-01-6	Blumenol B	12-F8	226.312	10(μ)M	DMSO
NCRC24330	BBP01029	22841-42-5	9-Epiblumenol B	12-F9	226.312	10(μ)M	DMSO
NCRC24331	BBP01030	520-32-1	Tricin	12-F10	330.289	10(μ)M	DMSO
NCRC24332	BBP01031	24314-59-8	Scandine	12-F11	350.411	10(μ)M	DMSO
NCRC24333	BBP01033	55604-88-1	1-Oxo-4-hydroxy-2-en-4-ethylcyclohexa-5,8-olide	12-G2	168.147	10(μ)M	DMSO
NCRC24334	BBP01034	672336-50-4	10(14)-Cadinene-4,5-diol	12-G3	238.366	10(μ)M	DMSO
NCRC24335	BBP01035	2141-09-5	Magnoflorine	12-G4	342.409	10(μ)M	DMSO
NCRC24336	BBP01036	23455-44-9	alpha-Spinasterone	12-G5	410.675	10(μ)M	DMSO
NCRC24337	BBP01038	53823-03-3	Onitisin	12-G6	264.317	10(μ)M	DMSO
NCRC24338	BBP01039	150710-72-8	Calyxamine B	12-G7	195.301	10(μ)M	DMSO
NCRC24339	BBP01040	129724-43-2	2',4'-Dihydroxy-3',6'-dimethoxychalcone	12-G8	300.306	10(μ)M	DMSO
NCRC24340	BBP01041	3561-81-7	Mesuaxanthone A	12-G9	258.226	10(μ)M	DMSO
NCRC24341	BBP01042	57566-47-9	Isofuranodiene	12-G10	216.319	10(μ)M	DMSO
NCRC24342	BBP01043	184046-40-0	Dimeric coniferyl acetate	12-G11	442.458	10(μ)M	DMSO
NCRC24343	BBP01044	213769-80-3	6,8-Cyclo-1,4-eudesmanediol	12-H2	238.366	10(μ)M	DMSO

NCRC24344	BBP01045	14028-97-8	N-Methyllindcarpine	12-H3	327.374	10(μ)M	DMSO
NCRC24345	BBP01055	34302-37-9	2,16-Kauranediol	12-H4	306.483	10(μ)M	DMSO
NCRC24346	BBP01047	33973-59-0	Triacetonamine hydrochloride	12-H5	191.698	10(μ)M	DMSO
NCRC24347	BBP01048	No	No	12-H6	752.779	10(μ)M	DMSO
NCRC24348	BBP01049	227471-20-7	Mucrolidin	12-H7	256.381	10(μ)M	DMSO
NCRC24349	BBP01050	119188-37-3	Coronarin D	12-H8	318.45	10(μ)M	DMSO
NCRC24350	BBP01051	20086-07-1	Diosbulbin C	12-H9	362.374	10(μ)M	DMSO
NCRC24351	BBP01052	19533-92-7	Alphitolic acid	12-H10	472.7	10(μ)M	DMSO
NCRC24352	BBP01053	67884-12-2	Martynoside	12-H11	652.64	10(μ)M	DMSO
NCRC24353	BBP01056	833-52-3	7-Hydroxycoumarin-6-carboxylic acid	13-A2	206.152	10(μ)M	DMSO
NCRC24354	BBP01057	53948-10-0	Aristolactam BIII	13-A3	309.316	10(μ)M	DMSO
NCRC24355	BBP01058	4707-47-5	Atraric acid	13-A4	196.2	10(μ)M	DMSO
NCRC24356	BBP01059	1154518-97-4	Sootepin D	13-A5	484.71	10(μ)M	DMSO
NCRC24357	BBP01060	35286-59-0	Ziyuglycoside II	13-A6	604.814	10(μ)M	DMSO
NCRC24358	BBP01061	19309-14-9	Cardamonin	13-A7	270.28	10(μ)M	DMSO
NCRC24359	BBP01062	119533-63-0	Ceanothic acid acetate	13-A8	528.72	10(μ)M	DMSO
NCRC24360	BBP01063	13956-51-9	Lycoclavanol	13-A9	458.716	10(μ)M	DMSO
NCRC24361	BBP01064	214150-74-0	1-Decarboxy-3-oxo-ceanothic acid	13-A10	440.658	10(μ)M	DMSO
NCRC24362	BBP01065	157528-81-9	Coronarin D methyl ether	13-A11	332.477	10(μ)M	DMSO
NCRC24363	BBP01066	111537-41-8	3',5-Dihydroxy-4',5',6,7-tetramethoxyflavone	13-B2	374.341	10(μ)M	DMSO
NCRC24364	BBP01068	2649-68-5	Clovanediol diacetate	13-B3	322.439	10(μ)M	DMSO
NCRC24365	BBP01069	155488-34-9	1,9-Caryolanediol 9-acetate	13-B4	280.402	10(μ)M	DMSO
NCRC24366	BBP01070	145400-02-8	1,4,7-Eudesmanetriol	13-B5	256.381	10(μ)M	DMSO
NCRC24367	BBP01071	5986-49-2	Palustrol	13-B6	222.366	10(μ)M	DMSO
NCRC24368	BBP01072	481-18-5	alpha-Spinasterol	13-B7	412.691	10(μ)M	DMSO
NCRC24369	BBP01073	No	No	13-B8	500.776	10(μ)M	DMSO
NCRC24370	BBP01074	120-80-9	1,2-Benzenediol	13-B9	110.111	10(μ)M	DMSO
NCRC24371	BBP01075	310888-07-4	5,7,3'-Trihydroxy-6,4',5'-trimethoxyflavanone	13-B10	362.331	10(μ)M	DMSO
NCRC24372	BBP01076	260968-11-4	Reneilmol	13-B11	256.381	10(μ)M	DMSO
NCRC24373	BBP01077	183075-03-8	3,4-O-Isopropylidene shikimic acid	13-C2	214.215	10(μ)M	DMSO
NCRC24374	BBP01078	57-87-4	Ergosterol	13-C3	396.648	10(μ)M	DMSO
NCRC24375	BBP01079	No	1,6-Diacetoxy-4(15),11(13)-eudesmadien-12-oic acid methyl ester	13-C4	364.433	10(μ)M	DMSO
NCRC24376	BBP01080	1001424-68-5	1,4,5,6-Tetrahydroxy-7-prenylxanthone	13-C5	328.316	10(μ)M	DMSO
NCRC24377	BBP01081	81053-14-7	4-Hydroxy-4-(methoxycarbonylmethyl)cyclohexanone	13-C6	186.205	10(μ)M	DMSO
NCRC24378	BBP00329	97914-19-7	3,4'-Dihydroxy-3',5,7-trimethoxyflavan	13-C7	332.348	10(μ)M	DMSO
NCRC24379	BBP01083	1911-78-0	Oplopanone	13-C8	238.366	10(μ)M	DMSO
NCRC24380	BBP01084	119188-47-5	10-Hydroxyscandine	13-C9	366.41	10(μ)M	DMSO
NCRC24381	BBP01085	691009-85-5	16-Hydroxy-8(17),13-labdadien-15,16-olid-19-oic acid	13-C10	348.433	10(μ)M	DMSO
NCRC24382	BBP01087	517883-38-4	Rengynic acid	13-C11	174.194	10(μ)M	DMSO
NCRC24383	BBP01088	28757-27-9	Apigenin 5-O-beta-D-glucopyranoside	13-D2	432.378	10(μ)M	DMSO
NCRC24384	BBP01089	490-46-0	Epicatechin	13-D3	290.268	10(μ)M	DMSO
NCRC24385	BBP01091	68097-13-2	4',5,7-Trihydroxy-6-prenylflavone	13-D4	338.354	10(μ)M	DMSO
NCRC24386	BBP01094	53452-32-7	16-Kaurene-2,6,15-triol	13-D5	320.466	10(μ)M	DMSO
NCRC24387	BBP01095	155485-76-0	3,6-Caryolanediol	13-D6	238.366	10(μ)M	DMSO

NCRC24388	BBP01096	2649-64-1	Clovanediol	13-D7	238.366	10(μ)M	DMSO
NCRC24389	BBP01097	4407-36-7	3-Phenyl-2-propen-1-ol	13-D8	134.175	10(μ)M	DMSO
NCRC24390	BBP01098	No	10-Acetoxyscandine	13-D9	408.447	10(μ)M	DMSO
NCRC24391	BBP01101	2955-23-9	Olivil	13-D10	376.4	10(μ)M	DMSO
NCRC24392	BBP01102	486430-93-7	O-Acetylcyclocalopin A	13-D11	338.352	10(μ)M	DMSO
NCRC24393	BBP01103	87-66-1	Pyrogallol	13-E2	126.11	10(μ)M	DMSO
NCRC24394	BBP01104	40768-81-8	1,7-Diepi-8,15-cedranediol	13-E3	238.366	10(μ)M	DMSO
NCRC24395	BBP01105	138965-88-5	Isocoronarin D	13-E4	318.45	10(μ)M	DMSO
NCRC24396	BBP01106	4728-30-7	8(14),15-Isopimaradien-3-ol	13-E5	288.467	10(μ)M	DMSO
NCRC24397	BBP01107	136133-08-9	Ellagic acid 7-O-beta-D-xylopyranoside-2,3,8-trimethyl ether	13-E6	476.387	10(μ)M	DMSO
NCRC24398	BBP01109	486430-94-8	Cyclocalopin A	13-E7	296.316	10(μ)M	DMSO
NCRC24399	BBP01110	30828-09-2	4-Oxododecanedioic acid	13-E8	244.284	10(μ)M	DMSO
NCRC24400	BBP01112	88642-46-0	Cerebroside B	13-E9	728.051	10(μ)M	DMSO
NCRC24401	BBP01113	66-22-8	Uracil	13-E10	112.087	10(μ)M	DMSO
NCRC24402	BBP01114	6890-88-6	Eburicol	13-E11	440.744	10(μ)M	DMSO
NCRC24403	BBP01115	138965-89-6	Coronarin D ethyl ether	13-F2	346.504	10(μ)M	DMSO
NCRC24404	BBP01116	No	Isocoronarin D methylthiomethyl ether	13-F3	378.569	10(μ)M	DMSO
NCRC24405	BBP01117	143120-46-1	Neotuberostemonine	13-F4	375.502	10(μ)M	DMSO
NCRC24406	BBP01118	173294-74-1	Garciniaxanthone E	13-F5	464.55	10(μ)M	DMSO
NCRC24407	BBP01119	484-20-8	Bergapten	13-F6	216.19	10(μ)M	DMSO
NCRC24408	BBP01121	No	No	13-F7	323.427	10(μ)M	DMSO
NCRC24409	BBP01123	58-61-7	Adenosine	13-F8	267.241	10(μ)M	DMSO
NCRC24410	BBP01124	446-72-0	Genistein	13-F9	270.237	10(μ)M	DMSO
NCRC24411	BBP01125	90-24-4	Xanthoxylin	13-F10	196.2	10(μ)M	DMSO
NCRC24412	BBP01126	20086-06-0	Diosbulbin B	13-F11	344.358	10(μ)M	DMSO
NCRC24413	BBP01129	474893-07-7	2,16-Kauranediol 2-O-beta-D-allopyranoside	13-G2	468.623	10(μ)M	DMSO
NCRC24414	BBP01130	523-50-2	Angelicin	13-G3	186.164	10(μ)M	DMSO
NCRC24415	BBP01131	67-47-0	5-Hydroxymethylfurfural	13-G4	126.11	10(μ)M	DMSO
NCRC24416	BBP01132	144424-80-6	3-O-(E)-p-Coumaroylbetulin	13-G5	588.86	10(μ)M	DMSO
NCRC24417	BBP01134	10309-37-2	Bakuchiol	13-G6	256.383	10(μ)M	DMSO
NCRC24418	BBP01135	83725-24-0	Pomolic acid 28-O-beta-D-glucopyranosyl ester	13-G7	634.84	10(μ)M	DMSO
NCRC24419	BBP01136	943136-39-8	Chlorahololide D	13-G8	676.749	10(μ)M	DMSO
NCRC24420	BBP01138	103630-03-1	Catechin 3-rhamnoside	13-G9	436.409	10(μ)M	DMSO
NCRC24421	BBP01139	60048-88-6	N-Demethylechitamine	13-G10	370.442	10(μ)M	DMSO
NCRC24422	BBP01140	66-97-7	Psoralen	13-G11	186.164	10(μ)M	DMSO
NCRC24423	BBP01141	769928-72-5	15-Methoxypinusolidic acid	13-H2	362.46	10(μ)M	DMSO
NCRC24424	BBP01142	58762-96-2	Pinostilbenoside	13-H3	404.41	10(μ)M	DMSO
NCRC24425	BBP01143	958885-86-4	12-Hydroxy-8(17),13-labdadien-16,15-olide	13-H4	318.45	10(μ)M	DMSO
NCRC24426	BBP60059	160047-56-3	Sambutoxin	13-H5	453.614	10(μ)M	DMSO
NCRC24427	BBP01145	106861-40-9	Bisdehydrotuberostemonine	13-H6	371.47	10(μ)M	DMSO
NCRC24428	BBP01146	521-61-9	Physcion	13-H7	284.263	10(μ)M	DMSO
NCRC24429	BBP01147	826-36-8	Triacetonamine	13-H8	155.237	10(μ)M	DMSO
NCRC24430	BBP01148	477953-07-4	Neostenine	13-H9	277.402	10(μ)M	DMSO
NCRC24431	BBP01149	528-43-8	Magnolol	13-H10	266.334	10(μ)M	DMSO
NCRC24432	BBP01150	153-18-4	Rutin	13-H11	610.518	10(μ)M	DMSO
NCRC24433	BBP01153	2457-80-9	5'-S-Methyl-5'-thioadenosine	14-A2	297.334	10(μ)M	DMSO
NCRC24434	BBP01154	112237-71-5	16-O-Methyl-14,15-didehydroisovincanol	14-A3	308.417	10(μ)M	DMSO
NCRC24435	BBP01155	22255-10-3	alpha-Amyrin palmitate	14-A4	665.126	10(μ)M	DMSO

NCRC24436	BBP01156	53755-76-3	Lyclaninol	14-A5	474.716	10(μ)M	DMSO
NCRC24437	BBP01159	485-19-8	Reticuline	14-A6	329.39	10(μ)M	DMSO
NCRC24438	BBP01160	19879-30-2	Bavachinin	14-A7	338.397	10(μ)M	DMSO
NCRC24439	BBP01161	2086-83-1	Berberine	14-A8	336.361	10(μ)M	DMSO
NCRC24440	BBP01164	81264-00-8	ent-6,9-Dihydroxy-15-oxo-16-kauren-19-oic acid	14-A9	348.433	10(μ)M	DMSO
NCRC24441	BBP01166	24513-51-7	3,21-Dihydroxy-14-serraten-16-one	14-A10	456.7	10(μ)M	DMSO
NCRC24442	BBP01167	27740-43-8	Erysotrine	14-A11	313.391	10(μ)M	DMSO
NCRC24443	BBP01168	83011-43-2	Methyl 3-hydroxy-4,5-dimethoxybenzoate	14-B2	212.199	10(μ)M	DMSO
NCRC24444	BBP01170	24352-51-0	3,5-Cycloergosta-6,8(14),22-	14-B3	378.633	10(μ)M	DMSO
NCRC24445	BBP01171	221150-18-1	Erysubin A	14-B4	352.337	10(μ)M	DMSO
NCRC24446	BBP01172	1014974-98-1	No	14-B5	658.602	10(μ)M	DMSO
NCRC24447	BBP01173	642-71-7	Antiarol	14-B6	184.189	10(μ)M	DMSO
NCRC24448	BBP01174	20784-60-5	4'-O-Methylbroussochalcone B	14-B7	338.397	10(μ)M	DMSO
NCRC24449	BBP01175	53947-92-5	Corylin	14-B8	320.339	10(μ)M	DMSO
NCRC24450	BBP01176	929637-35-4	Sessilifoline A	14-B9	389.485	10(μ)M	DMSO
NCRC24451	BBP01177	573-44-4	Liriodendrin	14-B10	742.718	10(μ)M	DMSO
NCRC24452	BBP01178	220880-90-0	2,3-O-Isopropylidenyl euscaphic acid	14-B11	528.763	10(μ)M	DMSO
NCRC24453	BBP01179	1190225-47-8	Sarcandrone A	14-C2	554.587	10(μ)M	DMSO
NCRC24454	BBP01181	14351-29-2	Dammarenediol II	14-C3	444.733	10(μ)M	DMSO
NCRC24455	BBP01183	57586-98-8	Cycloartane-3,24,25-triol	14-C4	460.732	10(μ)M	DMSO
NCRC24456	BBP01184	66107-60-6	14,17-Epidioxy-28-nor-15-taraxerene-2,3-diol	14-C5	458.673	10(μ)M	DMSO
NCRC24457	BBP01185	24513-57-3	21-Episerratriol	14-C6	458.716	10(μ)M	DMSO
NCRC24458	BBP01186	31524-62-6	Isobavachin	14-C7	324.37	10(μ)M	DMSO
NCRC24459	BBP01187	19879-32-4	Bavachin	14-C8	324.37	10(μ)M	DMSO
NCRC24460	BBP01188	13956-52-0	Serratriol	14-C9	458.716	10(μ)M	DMSO
NCRC24461	BBP01189	1180-71-8	Limonin	14-C10	470.512	10(μ)M	DMSO
NCRC24462	BBP01190	863-76-3	alpha-Amyrin acetate	14-C11	468.754	10(μ)M	DMSO
NCRC24463	BBP01191	59219-64-6	8(14),15-Isopimaradiene-3,18-	14-D2	304.467	10(μ)M	DMSO
NCRC24464	BBP01193	1187951-05-8	Diosbulbin I	14-D3	506.544	10(μ)M	DMSO
NCRC24465	BBP01194	19942-04-2	3-Epicabraleadiol	14-D4	460.732	10(μ)M	DMSO
NCRC24466	BBP01195	66756-57-8	Diosbulbin D	14-D5	344.358	10(μ)M	DMSO
NCRC24467	BBP01196	6009-12-7	Confluentic acid	14-D6	500.581	10(μ)M	DMSO
NCRC24468	BBP01197	126882-53-9	Ssioriside	14-D7	554.583	10(μ)M	DMSO
NCRC24469	BBP01199	94410-22-7	Isomartynoside	14-D8	652.64	10(μ)M	DMSO
NCRC24470	BBP01200	219649-95-3	Griffipavixanthone	14-D9	652.6	10(μ)M	DMSO
NCRC24471	BBP01201	595-15-3	Soyasapogenol B	14-D10	458.716	10(μ)M	DMSO
NCRC24472	BBP01202	149-91-7	Gallic acid	14-D11	170.12	10(μ)M	DMSO
NCRC24473	BBP01204	55-21-0	Benzamide	14-E2	121.137	10(μ)M	DMSO
NCRC24474	BBP01205	504-15-4	Orcinol	14-E3	124.137	10(μ)M	DMSO
NCRC24475	BBP01206	751-03-1	Obacunone	14-E4	454.512	10(μ)M	DMSO
NCRC24476	BBP01207	261351-23-9	Antiarol rutinoside	14-E5	492.471	10(μ)M	DMSO
NCRC24477	BBP01208	35286-58-9	Ziyuglycoside I	14-E6	766.955	10(μ)M	DMSO
NCRC24478	BBP01209	87562-76-3	Kelampayoside A	14-E7	478.444	10(μ)M	DMSO
NCRC24479	BBP01213	No	3-Epiwightianol A tetraacetate	14-E8	660.878	10(μ)M	DMSO
NCRC24480	BBP01214	478-01-3	Nobiletin	14-E9	402.395	10(μ)M	DMSO
NCRC24481	BBP01215	23518-30-1	Licarin A	14-E10	326.386	10(μ)M	DMSO
NCRC24482	BBP01216	465-00-9	Arjunolic acid	14-E11	488.699	10(μ)M	DMSO
NCRC24483	BBP01217	144868-43-9	Garjasmin	14-F2	224.21	10(μ)M	DMSO
NCRC24484	BBP01218	139682-36-3	Gardenine	14-F3	223.225	10(μ)M	DMSO
NCRC24485	BBP01220	No	Diosbulbin C ethyl ester	14-F4	390.427	10(μ)M	DMSO

NCRC24486	BBP01221	114916-05-1	N1,N10-Bis(p-coumaroyl)spermidine	14-F5	437.531	10(μ)M	DMSO
NCRC24487	BBP01222	53452-34-9	Creticoside C	14-F6	484.623	10(μ)M	DMSO
NCRC24488	BBP01223	60-35-5	Acetamide	14-F7	59.0672	10(μ)M	DMSO
NCRC24489	BBP01226	3368-87-4	19,20-(E)-Vallesamine	14-F8	340.416	10(μ)M	DMSO
NCRC24490	BBP01227	1466-76-8	2,6-Dimethoxybenzoic acid	14-F9	182.173	10(μ)M	DMSO
NCRC24491	BBP01228	37239-47-7	Wilforgine	14-F10	857.807	10(μ)M	DMSO
NCRC24492	BBP01229	11088-09-8	Wilforine	14-F11	867.845	10(μ)M	DMSO
NCRC24493	BBP01230	1194-98-5	2,5-Dihydroxybenzaldehyde	14-G2	138.121	10(μ)M	DMSO
NCRC24494	BBP01231	67567-15-1	Diosbulbin G	14-G3	346.374	10(μ)M	DMSO
NCRC24495	BBP01232	83-74-9	Ibogaine	14-G4	310.433	10(μ)M	DMSO
NCRC24496	BBP01233	24512-63-8	Geniposide	14-G5	388.366	10(μ)M	DMSO
NCRC24497	BBP01234	525-21-3	Fraxidin	14-G6	222.194	10(μ)M	DMSO
NCRC24498	BBP01235	115753-79-2	Galanolactone	14-G7	318.45	10(μ)M	DMSO
NCRC24499	BBP01238	28619-41-2	Erythristemine	14-G8	343.417	10(μ)M	DMSO
NCRC24500	BBP01239	1033747-78-2	Itol A	14-G9	368.464	10(μ)M	DMSO
NCRC24501	BBP01240	134-96-3	4-Hydroxy-3,5-dimethoxybenzaldehyde	14-G10	182.173	10(μ)M	DMSO
NCRC24502	BBP01242	22570-53-2	Zeorin	14-G11	444.733	10(μ)M	DMSO
NCRC24503	BBP01243	10283-68-8	Dihydrocinchonamine	14-H2	298.423	10(μ)M	DMSO
NCRC24504	BBP01245	564-73-8	Hinokiol	14-H3	302.451	10(μ)M	DMSO
NCRC24505	BBP01247	No	Nyasicol 1,2-acetonide	14-H4	356.369	10(μ)M	DMSO
NCRC24506	BBP01248	111518-95-7	Nyasicol	14-H5	316.305	10(μ)M	DMSO
NCRC24507	BBP01249	142937-50-6	Triptoquinone B	14-H6	330.418	10(μ)M	DMSO
NCRC24508	BBP01250	142741-24-0	Conophylline	14-H7	794.889	10(μ)M	DMSO
NCRC24509	BBP01252	144735-57-9	2-(4-Hydroxy-3-methoxyphenyl)-7-methoxy-5-benzofuranpropanol	14-H8	328.359	10(μ)M	DMSO
NCRC24510	BBP01254	195735-16-1	2,6,16-Kauranetriol 2-O-beta-D-allopyranoside	14-H9	484.623	10(μ)M	DMSO
NCRC24511	BBP01255	491-54-3	Kaempferide	14-H10	300.263	10(μ)M	DMSO
NCRC24512	BBP01256	56973-66-1	9,9'-Di-O-(E)-feruloylsecoisolariciresinol	14-H11	714.754	10(μ)M	DMSO
NCRC24513	BBP01257	22031-64-7	Cinnamamide	15-A2	147.174	10(μ)M	DMSO
NCRC24514	BBP01259	52358-58-4	Erysotramidine	15-A3	327.374	10(μ)M	DMSO
NCRC24515	BBP01261	166322-14-1	9-O-Feruloyl-5,5'-dimethoxylariciresinol	15-A4	596.622	10(μ)M	DMSO
NCRC24516	BBP01262	1187951-06-9	Diosbulbin J	15-A5	378.373	10(μ)M	DMSO
NCRC24517	BBP01263	No	Glochionionol A	15-A6	240.296	10(μ)M	DMSO
NCRC24518	BBP01264	1136932-34-7	Shizukanolide H	15-A7	304.338	10(μ)M	DMSO
NCRC24519	BBP01265	828935-47-3	3-Oxo-24,25,26,27-tetranortirucall-7-en-23,21-olide	15-A8	398.578	10(μ)M	DMSO
NCRC24520	BBP01266	163513-81-3	Triptoquinonide	15-A9	326.386	10(μ)M	DMSO
NCRC24521	BBP01267	No	14-O-methylthiomethylitol A	15-A10	428.583	10(μ)M	DMSO
NCRC24522	BBP01269	51666-26-3	Erythrartine	15-A11	329.39	10(μ)M	DMSO
NCRC24523	BBP01270	20869-95-8	Ermanin	15-B2	314.289	10(μ)M	DMSO
NCRC24524	BBP01271	131-11-3	Dimethyl phthalate	15-B3	194.184	10(μ)M	DMSO
NCRC24525	BBP01272	464-92-6	Asiatic acid	15-B4	488.699	10(μ)M	DMSO
NCRC24526	BBP01273	83133-17-9	4',5-Dihydroxy-3',5',6,7-tetramethoxyflavone	15-B5	374.341	10(μ)M	DMSO
NCRC24527	BBP01274	625-04-7	Diacetonamine	15-B6	115.174	10(μ)M	DMSO
NCRC24528	BBP01275	57-50-1	Sucrose	15-B7	342.296	10(μ)M	DMSO
NCRC24529	BBP01276	31685-80-0	Pinusolide	15-B8	346.461	10(μ)M	DMSO
NCRC24530	BBP01277	2033-89-8	3,4-Dimethoxyphenol	15-B9	154.163	10(μ)M	DMSO
NCRC24531	BBP01278	41653-73-0	Koaburaside	15-B10	332.303	10(μ)M	DMSO
NCRC24532	BBP01280	53755-77-4	Lycernuic acid A	15-B11	472.7	10(μ)M	DMSO

NCRC24533	BBP01283	No	6-Ethoxygeniposide	15-C2	432.419	10(μ)M	DMSO
NCRC24534	BBP01284	15486-33-6	3,5-Dihydroxy-4',7-dimethoxyflavone	15-C3	314.289	10(μ)M	DMSO
NCRC24535	BBP01285	62470-46-6	p-Vinylphenyl O-beta-D-glucopyranoside	15-C4	282.289	10(μ)M	DMSO
NCRC24536	BBP01286	426821-85-4	4-Hydroxy-3-methoxyphenyl O-beta-D-(6'-O-syringate)glucopyranoside	15-C5	482.435	10(μ)M	DMSO
NCRC24537	BBP01287	73584-67-5	27-p-Coumaroyloxyursolic acid	15-C6	618.842	10(μ)M	DMSO
NCRC24538	BBP01288	No	18-O-Demethyldiosbulbin F	15-C7	362.374	10(μ)M	DMSO
NCRC24539	BBP01289	65597-42-4	Cerbinal	15-C8	204.179	10(μ)M	DMSO
NCRC24540	BBP01290	548-29-8	Isolariciresinol	15-C9	360.401	10(μ)M	DMSO
NCRC24541	BBP01291	No	8-Hydroxy-17-chloro-12-labden-16,15-olide	15-C10	354.911	10(μ)M	DMSO
NCRC24542	BBP01292	26652-12-0	Salirepin	15-C11	302.277	10(μ)M	DMSO
NCRC24543	BBP01293	136172-60-6	6-O-Caffeoylarbutin	15-D2	434.393	10(μ)M	DMSO
NCRC24544	BBP01294	58822-47-2	Secoxyloganin	15-D3	404.366	10(μ)M	DMSO
NCRC24545	BBP01297	23132-13-0	Heveaflavone	15-D4	580.538	10(μ)M	DMSO
NCRC24546	BBP01299	111518-94-6	Nyasicoside	15-D5	478.446	10(μ)M	DMSO
NCRC24547	BBP01300	66547-92-0	Dehydroheliobuphthalmin	15-D6	412.389	10(μ)M	DMSO
NCRC24548	BBP01301	64421-27-8	Mussaenoside	15-D7	390.382	10(μ)M	DMSO
NCRC24549	BBP01302	954379-68-1	Neotuberostemonone	15-D8	405.485	10(μ)M	DMSO
NCRC24550	BBP01303	18463-25-7	Nigracin	15-D9	406.383	10(μ)M	DMSO
NCRC24551	BBP01304	73002-86-5	15-Isopimarene-8,18-diol	15-D10	306.483	10(μ)M	DMSO
NCRC24552	BBP01305	3187-58-4	Methyl orsellinate	15-D11	182.173	10(μ)M	DMSO
NCRC24553	BBP01306	27530-67-2	Feretoside	15-E2	404.366	10(μ)M	DMSO
NCRC24554	BBP01307	54377-24-1	7-Hydroxy-5,8-dimethoxyflavanone	15-E3	300.306	10(μ)M	DMSO
NCRC24555	BBP01308	3650-31-5	Agatholal	15-E4	304.467	10(μ)M	DMSO
NCRC24556	BBP01309	5128-44-9	5-Hydroxy-4',7-dimethoxyflavone	15-E5	298.29	10(μ)M	DMSO
NCRC24557	BBP01310	69768-97-4	4-(3,4-Dimethoxyphenyl)-3-buten-1-ol	15-E6	208.254	10(μ)M	DMSO
NCRC24558	BBP01312	145544-91-8	Eucamalol	15-E7	168.233	10(μ)M	DMSO
NCRC24559	BBP01314	195723-38-7	2,16,19-Kauranetriol 2-O-beta-D-allopyranoside	15-E8	484.623	10(μ)M	DMSO
NCRC24560	BBP01315	95416-25-4	15-Hydroxy-7-oxo-8,11,13-abietatrien-18-oic acid	15-E9	330.418	10(μ)M	DMSO
NCRC24561	BBP01316	1038922-95-0	Mannioside A	15-E10	738.902	10(μ)M	DMSO
NCRC24562	BBP01317	1107620-67-6	Garcinexanthone A	15-E11	342.343	10(μ)M	DMSO
NCRC24563	BBP01318	149155-19-1	Homaloside D	15-F2	544.504	10(μ)M	DMSO
NCRC24564	BBP01320	173429-83-9	Ficusin A	15-F3	404.455	10(μ)M	DMSO
NCRC24565	BBP01321	81263-96-9	ent-9-Hydroxy-15-oxo-16-kauren-19-oic acid beta-D-glucopyranosyl ester	15-F4	494.575	10(μ)M	DMSO
NCRC24566	BBP01322	23407-76-3	Theviridoside	15-F5	404.366	10(μ)M	DMSO
NCRC24567	BBP01324	469-39-6	Cycloeucalenol	15-F6	426.717	10(μ)M	DMSO
NCRC24568	BBP01325	942480-13-9	Nemoralisin	15-F7	332.434	10(μ)M	DMSO
NCRC24569	BBP01326	36150-04-6	Mullilam diol	15-F8	188.264	10(μ)M	DMSO
NCRC24570	BBP01327	142279-42-3	Shizukaol D	15-F9	578.649	10(μ)M	DMSO
NCRC24571	BBP01328	29080-58-8	5-Hydroxy-3',4',7-trimethoxyflavone	15-F10	328.316	10(μ)M	DMSO
NCRC24572	BBP01329	50906-56-4	Arteannuin B	15-F11	248.318	10(μ)M	DMSO
NCRC24573	BBP01330	74560-05-7	Isomedicarpin	15-G2	270.28	10(μ)M	DMSO
NCRC24574	BBP01332	100198-09-2	Piscidinol A	15-G3	474.716	10(μ)M	DMSO
NCRC24575	BBP01333	117-81-7	Bis(2-ethylhexyl) phthalate	15-G4	390.556	10(μ)M	DMSO
NCRC24576	BBP01334	103917-26-6	Lupeol caffeate	15-G5	588.86	10(μ)M	DMSO

NCRC24577	BBP01335	943989-68-2	Iriflophenone 2-O-alpha-L-rhamnopyranoside	15-G6	392.357	10(μ)M	DMSO
NCRC24578	BBP01336	244204-40-8	Celaphanol A	15-G7	288.338	10(μ)M	DMSO
NCRC24579	BBP01337	34444-37-6	Nortrachelogenin	15-G8	374.384	10(μ)M	DMSO
NCRC24580	BBP01338	76-78-8	Quassin	15-G9	388.454	10(μ)M	DMSO
NCRC24581	BBP01339	60129-64-8	Paniculoside II	15-G10	496.59	10(μ)M	DMSO
NCRC24582	BBP01340	77658-45-8	ent-9-Hydroxy-15-oxo-19-kauranoic acid	15-G11	334.45	10(μ)M	DMSO
NCRC24583	BBP01341	77658-39-0	ent-9-Hydroxy-15-oxo-16-kauren-19-oic acid	15-H2	332.434	10(μ)M	DMSO
NCRC24584	BBP01342	68436-47-5	Isowighteone	15-H3	338.354	10(μ)M	DMSO
NCRC24585	BBP01343	545-46-0	Uvaol	15-H4	442.717	10(μ)M	DMSO
NCRC24586	BBP01344	93-07-2	3,4-Dimethoxybenzoic acid	15-H5	182.173	10(μ)M	DMSO
NCRC24587	BBP01345	115334-04-8	Phellochin	15-H6	488.742	10(μ)M	DMSO
NCRC24588	BBP01346	56324-54-0	Hedychenone	15-H7	298.419	10(μ)M	DMSO
NCRC24589	BBP01347	144881-21-0	4',9,9'-Trihydroxy-3'-methoxy-3,7'-epoxy-4,8'-oxyneolignan	15-H8	346.374	10(μ)M	DMSO
NCRC24590	BBP01348	100234-59-1	Picrasidine I	15-H9	240.257	10(μ)M	DMSO
NCRC24591	BBP01349	20316-62-5	Tiliroside	15-H10	594.52	10(μ)M	DMSO
NCRC24592	BBP01350	No	No	15-H11	414.448	10(μ)M	DMSO
NCRC24593	BBP01351	No	No	16-A2	442.458	10(μ)M	DMSO
NCRC24594	BBP01352	57719-76-3	11,15-Dihydroxy-16-kauren-19-oic acid	16-A3	334.45	10(μ)M	DMSO
NCRC24595	BBP01353	226562-47-6	3-Hydroxy-12-oleanene-23,28-dioic acid	16-A4	486.683	10(μ)M	DMSO
NCRC24596	BBP01355	23963-54-4	Glochidonol	16-A5	440.701	10(μ)M	DMSO
NCRC24597	BBP01358	89130-86-9	Betulin caffeate	16-A6	604.859	10(μ)M	DMSO
NCRC24598	BBP01359	72826-63-2	Deoxyartemisinin	16-A7	266.333	10(μ)M	DMSO
NCRC24599	BBP01360	126882-76-6	3-Methoxy-5-heneicosylphenol	16-A8	418.695	10(μ)M	DMSO
NCRC24600	BBP01361	39024-15-2	3-Epiwilsonine	16-A9	343.417	10(μ)M	DMSO
NCRC24601	BBP01362	39024-12-9	Wilsonine	16-A10	343.417	10(μ)M	DMSO
NCRC24602	BBP01363	80286-58-4	Artemisinic acid	16-A11	234.334	10(μ)M	DMSO
NCRC24603	BBP01367	52591-10-3	Iriflophenone	16-B2	246.215	10(μ)M	DMSO
NCRC24604	BBP01368	466-01-3	Hederagonic acid	16-B3	470.684	10(μ)M	DMSO
NCRC24605	BBP01369	16962-90-6	Odoratone	16-B4	472.7	10(μ)M	DMSO
NCRC24606	BBP01370	537-98-4	Ferulic acid	16-B5	194.184	10(μ)M	DMSO
NCRC24607	BBP01371	33228-65-8	Glucosyringic acid	16-B6	360.313	10(μ)M	DMSO
NCRC24608	BBP01372	20175-84-2	Isodiospyrin	16-B7	374.343	10(μ)M	DMSO
NCRC24609	BBP01373	40716-66-3	Nerolidol	16-B8	222.366	10(μ)M	DMSO
NCRC24610	BBP01374	140447-22-9	Ergosterol peroxide 3-O-beta-D-glucopyranoside	16-B9	590.788	10(μ)M	DMSO
NCRC24611	BBP01375	51804-68-3	Oxoepistephamiersine	16-B10	403.426	10(μ)M	DMSO
NCRC24612	BBP01376	51804-69-4	Dihydrooxoepistephamiersine	16-B11	405.442	10(μ)M	DMSO
NCRC24613	BBP01377	209115-67-3	Mangochinine	16-C2	328.382	10(μ)M	DMSO
NCRC24614	BBP01378	6985-35-9	Bourjotinolone A	16-C3	472.7	10(μ)M	DMSO
NCRC24615	BBP01380	No	23-Nor-3-oxo-12-oleanen-28-oic acid	16-C4	440.658	10(μ)M	DMSO
NCRC24616	BBP01381	578-74-5	Cosmosiin	16-C5	432.378	10(μ)M	DMSO
NCRC24617	BBP01382	25739-41-7	Velutin	16-C6	314.289	10(μ)M	DMSO
NCRC24618	BBP01383	73891-72-2	Hispidone	16-C7	472.7	10(μ)M	DMSO
NCRC24619	BBP01384	30315-04-9	Picrasin B acetate	16-C8	418.48	10(μ)M	DMSO
NCRC24620	BBP01385	97372-53-7	8-Hydroxy-4-cadinen-3-one	16-C9	236.35	10(μ)M	DMSO
NCRC24621	BBP01387	58-95-7	alpha-Tocopherol acetate	16-C10	472.743	10(μ)M	DMSO
NCRC24622	BBP01388	79491-71-7	9-Oxo-10,11-dehydroageraphorone	16-C11	232.318	10(μ)M	DMSO

NCRC24623	BBP01389	74713-15-8	Secoxyloganin methyl ester	16-D2	418.392	10(μ)M	DMSO
NCRC24624	BBP01391	337527-10-3	8,11,13-Abietatriene-7,15,18-triol	16-D3	318.45	10(μ)M	DMSO
NCRC24625	BBP01392	24274-60-0	Acetylcephalotaxine	16-D4	357.4	10(μ)M	DMSO
NCRC24626	BBP01393	No	Simiarenol methylthiomethyl	16-D5	486.836	10(μ)M	DMSO
NCRC24627	BBP01394	29028-10-2	3-Epiglochidiol	16-D6	442.717	10(μ)M	DMSO
NCRC24628	BBP01397	51095-85-3	2,7-Dihydrohomoerysotrine	16-D7	329.433	10(μ)M	DMSO
NCRC24629	BBP01398	139726-29-7	Dunnianol	16-D8	398.494	10(μ)M	DMSO
NCRC24630	BBP01399	104975-02-2	3-Acetoxy-4,7(11)-cadinadien-8-one	16-D9	276.371	10(μ)M	DMSO
NCRC24631	BBP01400	33900-74-2	2,3,4'-Trihydroxy-3',5'-dimethoxypropiophenone	16-D10	242.225	10(μ)M	DMSO
NCRC24632	BBP60001	55481-88-4	Mollugin	16-D11	284.307	10(μ)M	DMSO
NCRC24633	BBP60002	61658-41-1	Furomollugin	16-E2	242.227	10(μ)M	DMSO
NCRC24634	BBP60003	15291-75-5	Ginkgolide A	16-E3	408.399	10(μ)M	DMSO
NCRC24635	BBP60004	15291-77-7	Ginkgolide B	16-E4	424.399	10(μ)M	DMSO
NCRC24636	BBP60005	15291-76-6	Ginkgolide C	16-E5	440.398	10(μ)M	DMSO
NCRC24637	BBP60006	6807-83-6	Trifolirhizin	16-E6	446.404	10(μ)M	DMSO
NCRC24638	BBP60007	519-02-8	Matrine	16-E7	248.364	10(μ)M	DMSO
NCRC24639	BBP60008	16837-52-8	Oxymatrine	16-E8	264.363	10(μ)M	DMSO
NCRC24640	BBP60009	3621-38-3	Jatrorrhizine	16-E9	338.377	10(μ)M	DMSO
NCRC24641	BBP60010	3486-67-7	Palmatine	16-E10	352.404	10(μ)M	DMSO
NCRC24642	BBP60011	481-72-1	Aloeemodin	16-E11	270.237	10(μ)M	DMSO
NCRC24643	BBP60012	2447-54-3	Sanguinarine	16-F2	332.329	10(μ)M	DMSO
NCRC24644	BBP60013	7689-03-4	Camptothecin	16-F3	348.352	10(μ)M	DMSO
NCRC24645	BBP60014	58316-41-9	Saikosaponin B2	16-F4	780.982	10(μ)M	DMSO
NCRC24646	BBP60015	29883-15-6	Amygdalin	16-F5	457.428	10(μ)M	DMSO
NCRC24647	BBP60016	50773-41-6	Chonglou Saponin I	16-F6	855.017	10(μ)M	DMSO
NCRC24648	BBP60017	68124-04-9	Chonglou Saponin VII	16-F7	1031.18	10(μ)M	DMSO
NCRC24649	BBP60018	21082-33-7	Sakakin	16-F8	286.278	10(μ)M	DMSO
NCRC24650	BBP60019	19254-69-4	Ergosta-4,6,8(14),22-tetraen-3-	16-F9	392.617	10(μ)M	DMSO
NCRC24651	BBP60020	22144-77-0	Cytochalasin D	16-F10	507.618	10(μ)M	DMSO
NCRC24652	BBP60021	477-90-7	Bergenin	16-F11	328.271	10(μ)M	DMSO
NCRC24653	BBP60022	14531-47-6	Penta-acetate bergenin	16-G2	538.455	10(μ)M	DMSO
NCRC24654	BBP60023	33815-57-5	Di-O-methylbergenin	16-G3	356.325	10(μ)M	DMSO
NCRC24655	BBP60024	No	11-O-(3-nitrobenzoyl)bergenin	16-G4	477.375	10(μ)M	DMSO
NCRC24656	BBP60025	85643-19-2	Curculigoside	16-G5	466.435	10(μ)M	DMSO
NCRC24657	BBP60026	146905-24-0	1,2-Diacetoxy-4,7,8-trihydroxy-3-(4-hydroxyphenyl)dibenzofuran	16-G6	424.357	10(μ)M	DMSO
NCRC24658	BBP60027	479-20-9	Atranorin	16-G7	374.341	10(μ)M	DMSO
NCRC24659	BBP60028	63968-64-9	Artemisinin	16-G8	282.332	10(μ)M	DMSO
NCRC24660	BBP60029	569-83-5	Xanthohumol	16-G9	354.396	10(μ)M	DMSO
NCRC24661	BBP60030	274675-25-1	Xanthohumol D	16-G10	370.396	10(μ)M	DMSO
NCRC24662	BBP60031	647853-82-5	No	16-G11	478.446	10(μ)M	DMSO
NCRC24663	BBP60032	60-81-1	Phloridzin	16-H2	436.409	10(μ)M	DMSO
NCRC24664	BBP60033	4192-90-9	p-Phlorizin	16-H3	436.409	10(μ)M	DMSO
NCRC24665	BBP60034	80557-12-6	Grifolic acid	16-H4	372.498	10(μ)M	DMSO
NCRC24666	BBP60035	329975-47-5	3,4-Secocucurbita-4,24-diene-3,26,29-trioic acid	16-H5	502.683	10(μ)M	DMSO
NCRC24667	BBP60036	50-89-5	Thymidine	16-H6	242.229	10(μ)M	DMSO
NCRC24668	BBP60037	38395-02-7	Caudatin	16-H7	490.629	10(μ)M	DMSO
NCRC24669	BBP60038	84745-94-8	Qingyanshengenin	16-H8	500.581	10(μ)M	DMSO
NCRC24670	BBP60039	152175-76-3	Forrestin A	16-H9	578.648	10(μ)M	DMSO
NCRC24671	BBP60040	269742-39-4	16-Acetoxy-7-O-acetylhorminone	16-H10	432.507	10(μ)M	DMSO
NCRC24672	BBP60041	120462-42-2	Lophanthoidin B	16-H11	448.506	10(μ)M	DMSO
NCRC24673	BBP60042	120462-45-5	Lophanthoidin E	17-A2	406.469	10(μ)M	DMSO

NCRC24674	BBP60043	120462-46-6	Lophanthoidin F	17-A3	434.523	10(μ)M	DMSO
NCRC24675	BBP60044	125164-55-8	Rosthornin A	17-A4	376.486	10(μ)M	DMSO
NCRC24676	BBP60045	125181-21-7	Rosthornin B	17-A5	434.523	10(μ)M	DMSO
NCRC24677	BBP60046	64657-21-2	Coleonol B	17-A6	410.501	10(μ)M	DMSO
NCRC24678	BBP60047	473981-11-2	Forskolin G	17-A7	436.538	10(μ)M	DMSO
NCRC24679	BBP60048	81873-08-7	Forskolin J	17-A8	452.538	10(μ)M	DMSO
NCRC24680	BBP60049	123043-54-9	Bulleyanin	17-A9	534.595	10(μ)M	DMSO
NCRC24681	BBP60050	122717-54-8	Liangshanin A	17-A10	330.418	10(μ)M	DMSO
NCRC24682	BBP60051	78536-36-4	Excisanin B	17-A11	392.486	10(μ)M	DMSO
NCRC24683	BBP60052	39388-57-3	Kamebanin	17-B2	334.45	10(μ)M	DMSO
NCRC24684	BBP60053	85329-59-5	Trichorabdal A	17-B3	346.417	10(μ)M	DMSO
NCRC24685	BBP60054	959860-49-2	Guajadial	17-B4	474.588	10(μ)M	DMSO
NCRC24686	BBP60055	491-67-8	Baicalein	17-B5	270.237	10(μ)M	DMSO
NCRC24687	BBP60056	5508-58-7	Andrographolide	17-B6	350.449	10(μ)M	DMSO
NCRC24688	BBP60057	28955-30-8	Cassiachromone	17-B7	232.232	10(μ)M	DMSO
NCRC24689	BBP60058	No	Dihydrocassiachromone	17-B8	234.248	10(μ)M	DMSO
NCRC24690	BBP01401	168254-95-3	Wilforol C	17-B9	472.7	10(μ)M	DMSO
NCRC24691	BBP01402	116498-58-9	5,5'-Dimethoxylariciresinol	17-B10	420.453	10(μ)M	DMSO
NCRC24692	BBP01403	2318-78-7	Simiarenone	17-B11	424.702	10(μ)M	DMSO
NCRC24693	BBP01404	4965-99-5	Simiarenol acetate	17-C2	468.754	10(μ)M	DMSO
NCRC24694	BBP01405	20831-76-9	Gentiopicroside	17-C3	356.325	10(μ)M	DMSO
NCRC24695	BBP01406	890928-81-1	24,25-Epoxytirucall-7-en-3,23-dione	17-C4	454.684	10(μ)M	DMSO
NCRC24696	BBP01407	No	Simplidin butyl ether	17-C5	446.49	10(μ)M	DMSO
NCRC24697	BBP01408	458-37-7	Curcumin	17-C6	368.38	10(μ)M	DMSO
NCRC24698	BBP01409	29388-59-8	Secoisolariciresinol	17-C7	362.417	10(μ)M	DMSO
NCRC24699	BBP01410	305364-91-4	5'-Demethylaquillochin	17-C8	402.352	10(μ)M	DMSO
NCRC24700	BBP01411	421583-14-4	Cephalocyclidin A	17-C9	317.336	10(μ)M	DMSO
NCRC24701	BBP01412	57361-74-7	Dihydroepistephamiersine 6-acetate	17-C10	433.495	10(μ)M	DMSO
NCRC24702	BBP01415	596799-30-3	Calyciphylline A	17-C11	385.497	10(μ)M	DMSO
NCRC24703	BBP01416	7044-31-7	17alpha-Neriifolin	17-D2	534.681	10(μ)M	DMSO
NCRC24704	BBP01417	52389-15-8	Epistephamiersine	17-D3	389.442	10(μ)M	DMSO
NCRC24705	BBP01418	139122-81-9	Tripterifordin	17-D4	318.45	10(μ)M	DMSO
NCRC24706	BBP01419	3772-56-3	Totaradiol	17-D5	302.451	10(μ)M	DMSO
NCRC24707	BBP01420	63807-90-9	Dihydroalpinumisoflavone	17-D6	338.354	10(μ)M	DMSO
NCRC24708	BBP01421	60761-00-4	2-Hydroxy-1,8-cineole	17-D7	170.249	10(μ)M	DMSO
NCRC24709	BBP01422	55511-08-5	Biondinin C	17-D8	300.392	10(μ)M	DMSO
NCRC24710	BBP01423	155709-41-4	Isomagnolone	17-D9	282.334	10(μ)M	DMSO
NCRC24711	BBP01425	139726-30-0	Isodunnianol	17-D10	398.494	10(μ)M	DMSO
NCRC24712	BBP01426	65388-03-6	alpha-Isowighteone	17-D11	338.354	10(μ)M	DMSO
NCRC24713	BBP01427	28199-69-1	Dihydrodehydrodiconiferyl alcohol	17-E2	360.401	10(μ)M	DMSO
NCRC24714	BBP01428	114586-47-9	Thevebioside	17-E3	696.822	10(μ)M	DMSO
NCRC24715	BBP01429	21401-21-8	Taxiphyllin	17-E4	311.287	10(μ)M	DMSO
NCRC24716	BBP01430	529-44-2	Myricetin	17-E5	318.235	10(μ)M	DMSO
NCRC24717	BBP01431	16844-71-6	Epifriedelanol	17-E6	428.733	10(μ)M	DMSO
NCRC24718	BBP01432	247036-52-8	6-Deoxy-9alpha-hydroxycedrodorin	17-E7	502.553	10(μ)M	DMSO
NCRC24719	BBP01433	123621-00-1	Clerosterol glucoside	17-E8	574.831	10(μ)M	DMSO
NCRC24720	BBP01434	262355-96-4	7,15-Dihydroxy-8(14)-podocarpen-13-one	17-E9	278.387	10(μ)M	DMSO
NCRC24721	BBP01435	115074-93-6	Soyacerebroside II	17-E10	714.025	10(μ)M	DMSO
NCRC24722	BBP01436	2761-77-5	Communic acid	17-E11	302.451	10(μ)M	DMSO

NCRC24723	BBP01438	164661-12-5	4-(3,4-Dimethoxyphenyl)-3-butene-1,2-diol	17-F2	224.253	10(μ)M	DMSO
NCRC24724	BBP01440	74683-19-5	3-Methoxy-4,5-methylenedioxycinnamaldehyde	17-F3	206.195	10(μ)M	DMSO
NCRC24725	BBP01441	70389-88-7	Voleneol	17-F4	238.366	10(μ)M	DMSO
NCRC24726	BBP01442	14694-15-6	Codaphniphylline	17-F5	469.699	10(μ)M	DMSO
NCRC24727	BBP01443	6610-55-5	Glochidone	17-F6	422.686	10(μ)M	DMSO
NCRC24728	BBP01444	52914-31-5	Dammaradienyl acetate	17-F7	468.754	10(μ)M	DMSO
NCRC24729	BBP01445	84567-08-8	4-Hydroxycephalotaxine	17-F8	331.363	10(μ)M	DMSO
NCRC24730	BBP01446	545-48-2	Erythrodiol	17-F9	442.717	10(μ)M	DMSO
NCRC24731	BBP01447	1202-41-1	3,4-Dihydroxycinnamamide	17-F10	179.173	10(μ)M	DMSO
NCRC24732	BBP01448	140447-14-9	11-Hydroxyjasmonic acid	17-F11	226.269	10(μ)M	DMSO
NCRC24733	BBP01449	217810-46-3	9,16-Dioxo-10,12,14-octadecatrienoic acid	17-G2	306.397	10(μ)M	DMSO
NCRC24734	BBP01450	1745-36-4	alpha-Spinasterol glucoside	17-G3	574.831	10(μ)M	DMSO
NCRC24735	BBP01451	4651-46-1	alpha-Spinasterol acetate	17-G4	454.728	10(μ)M	DMSO
NCRC24736	BBP01452	84104-71-2	Wilforlide A	17-G5	454.684	10(μ)M	DMSO
NCRC24737	BBP01453	114613-59-1	17alpha-Thevebioside	17-G6	696.822	10(μ)M	DMSO
NCRC24738	BBP01455	1092555-03-7	Paxiphylline E	17-G7	399.48	10(μ)M	DMSO
NCRC24739	BBP01456	155051-85-7	4-Hydroxy-3-(3-methyl-2-butenoyl)-5-(3-methyl-2-butenyl)benzoic acid	17-G8	288.338	10(μ)M	DMSO
NCRC24740	BBP01457	81263-97-0	ent-6,11-Dihydroxy-15-oxo-16-kauren-19-oic acid beta-D-glucopyranosyl ester	17-G9	510.574	10(μ)M	DMSO
NCRC24741	BBP01458	65894-41-9	Daturabietatriene	17-G10	302.451	10(μ)M	DMSO
NCRC24742	BBP01459	203455-81-6	18-nor-8,11,13-Abietatriene-4,15-diol	17-G11	288.424	10(μ)M	DMSO
NCRC24743	BBP01461	127-27-5	Pimaric acid	17-H2	302.451	10(μ)M	DMSO
NCRC24744	BBP01462	112244-29-8	Stigmastane-3,6-diol	17-H3	432.722	10(μ)M	DMSO
NCRC24745	BBP01464	1259-94-5	24-Methylenecycloartanol acetate	17-H4	482.781	10(μ)M	DMSO
NCRC24746	BBP01465	39729-21-0	Daphmacropodine	17-H5	513.752	10(μ)M	DMSO
NCRC24747	BBP01467	97-59-6	Allantoin	17-H6	158.115	10(μ)M	DMSO
NCRC24748	BBP01468	126594-64-7	Cerberidol	17-H7	172.222	10(μ)M	DMSO
NCRC24749	BBP01470	380487-65-0	alpha-Epoxydihydroartemisinic acid	17-H8	252.349	10(μ)M	DMSO
NCRC24750	BBP01471	59014-02-7	8-Hydroxyhyperforin 8,1-hemiacetal	17-H9	552.784	10(μ)M	DMSO
NCRC24751	BBP01474	6246-46-4	Ursonic acid	17-H10	454.684	10(μ)M	DMSO
NCRC24752	BBP01475	81263-98-1	ent-6,9-Dihydroxy-15-oxo-16-kauren-19-oic acid beta-D-glucopyranosyl ester	17-H11	510.574	10(μ)M	DMSO
NCRC24753	BBP01476	37687-34-6	6-Acetonyldihydrosanguinarine	18-A2	389.401	10(μ)M	DMSO
NCRC24754	BBP01477	13403-14-0	Methyl beta-D-fructofuranoside	18-A3	194.182	10(μ)M	DMSO
NCRC24755	BBP01478	1820-84-4	Ethyl beta-D-fructofuranoside	18-A4	208.209	10(μ)M	DMSO
NCRC24756	BBP01479	126594-66-9	Cyclocerberidol	18-A5	188.221	10(μ)M	DMSO
NCRC24757	BBP01480	84108-17-8	Triptotriterpenic acid A	18-A6	472.7	10(μ)M	DMSO
NCRC24758	BBP01481	217650-27-6	4-Cadinen-7-ol	18-A7	222.366	10(μ)M	DMSO
NCRC24759	BBP01482	207446-92-2	Arteannuin N	18-A8	250.333	10(μ)M	DMSO
NCRC24760	BBP01483	207446-89-7	Arteannuin L	18-A9	250.333	10(μ)M	DMSO
NCRC24761	BBP01485	81371-54-2	Momordicoside G	18-A10	632.867	10(μ)M	DMSO
NCRC24762	BBP01486	30273-62-2	3,4,5-Trimethoxycinnamyl	18-A11	224.253	10(μ)M	DMSO
NCRC24763	BBP01487	640289-58-3	4(15)-Oppositene-1,7-diol	18-B2	238.366	10(μ)M	DMSO

NCRC24764	BBP01488	38968-07-9	2'-O-Methylperlatolic acid	18-B3	458.544	10(μ)M	DMSO
NCRC24765	BBP01489	22864-92-2	6-Acetonyldihydrochelerythrine	18-B4	405.443	10(μ)M	DMSO
NCRC24766	BBP01491	66648-50-8	Ethyl caffeate	18-B5	208.211	10(μ)M	DMSO
NCRC24767	BBP01492	60820-94-2	Phytolaccoside B	18-B6	664.823	10(μ)M	DMSO
NCRC24768	BBP01493	104669-02-5	Iriflophenone 3-C-beta-D-glucopyranoside	18-B7	408.356	10(μ)M	DMSO
NCRC24769	BBP01494	249916-07-2	Borreriagenin	18-B8	214.215	10(μ)M	DMSO
NCRC24770	BBP01495	480-19-3	Isorhamnetin	18-B9	316.262	10(μ)M	DMSO
NCRC24771	BBP01498	57576-34-8	(2,4-Dihydroxyphenyl)acetonitrile	18-B10	149.147	10(μ)M	DMSO
NCRC24772	BBP01499	923950-05-4	3-Acetoxy-4-cadinen-8-one	18-B11	278.387	10(μ)M	DMSO
NCRC24773	BBP01500	6872-88-4	Xanthoplanine	18-C2	356.435	10(μ)M	DMSO
NCRC24774	BBP01501	7224-61-5	Laurifoline	18-C3	342.409	10(μ)M	DMSO
NCRC24775	BBP01502	881388-88-1	Daphniyunnine B	18-C4	343.46	10(μ)M	DMSO
NCRC24776	BBP01503	197018-71-6	5-Hydroxy-4',7-dimethoxyflavone 5-O-beta-D-glucopyranoside	18-C5	460.431	10(μ)M	DMSO
NCRC24777	BBP01504	28420-25-9	6-Hydroxy-2,6-dimethyl-2,7-octadienoic acid	18-C6	184.232	10(μ)M	DMSO
NCRC24778	BBP01506	280565-85-7	Rediocide A	18-C7	794.924	10(μ)M	DMSO
NCRC24779	BBP01507	81910-41-0	Momordicoside I aglycone	18-C8	456.7	10(μ)M	DMSO
NCRC24780	BBP01508	213329-45-4	15,18-Dihydroxy-8,11,13-abietatrien-7-one	18-C9	316.435	10(μ)M	DMSO
NCRC24781	BBP01509	85372-72-1	5,19-Epoxy-19,25-dimethoxycucurbita-6,23-dien-3-	18-C10	500.753	10(μ)M	DMSO
NCRC24782	BBP01510	87095-74-7	5-Hydroxy-1,7-diphenyl-6-hepten-3-one	18-C11	280.361	10(μ)M	DMSO
NCRC24783	BBP01511	912329-03-4	Karavilagenin A	18-D2	486.769	10(μ)M	DMSO
NCRC24784	BBP01512	677277-98-4	Rediocide C	18-D3	814.913	10(μ)M	DMSO
NCRC24785	BBP01513	1210347-50-4	Abiesadine I	18-D4	402.524	10(μ)M	DMSO
NCRC24786	BBP01514	491-71-4	Chrysoeriol	18-D5	300.263	10(μ)M	DMSO
NCRC24787	BBP01515	143519-04-4	3-Acetoxy-24-hydroxydammara-20,25-diene	18-D6	484.753	10(μ)M	DMSO
NCRC24788	BBP01516	21040-45-9	Cinnamyl acetate	18-D7	176.212	10(μ)M	DMSO
NCRC24789	BBP01517	127350-68-9	Coclauril	18-D8	151.163	10(μ)M	DMSO
NCRC24790	BBP01518	2688-49-5	6-Hydroxybenzofuran-2(3H)-one	18-D9	150.131	10(μ)M	DMSO
NCRC24791	BBP01519	No	alpha-Spinasterol methylthiomethyl ether	18-D10	472.809	10(μ)M	DMSO
NCRC24792	BBP01520	155709-40-3	Simonsinol	18-D11	398.494	10(μ)M	DMSO
NCRC24793	BBP01521	92466-31-4	4,5-Epoxyartemisinic acid	18-E2	250.333	10(μ)M	DMSO
NCRC24794	BBP01522	24240-04-8	Allocryptopine	18-E3	369.411	10(μ)M	DMSO
NCRC24795	BBP01523	33116-33-5	Stephavanine	18-E4	497.494	10(μ)M	DMSO
NCRC24796	BBP01524	194940-15-3	4-Hydroxycinnamamide	18-E5	163.173	10(μ)M	DMSO
NCRC24797	BBP01525	486-39-5	Coclaurine	18-E6	285.338	10(μ)M	DMSO
NCRC24798	BBP01528	619-57-8	4-Hydroxybenzamide	18-E7	137.136	10(μ)M	DMSO
NCRC24799	BBP01529	221289-20-9	Lethedoside A	18-E8	490.457	10(μ)M	DMSO
NCRC24800	BBP01530	14260-99-2	Daphylloside	18-E9	446.403	10(μ)M	DMSO
NCRC24801	BBP01531	109194-60-7	Tachioside	18-E10	302.277	10(μ)M	DMSO
NCRC24802	BBP01532	58738-31-1	16-Oxoprometaphanine	18-E11	373.4	10(μ)M	DMSO
NCRC24803	BBP01533	155205-65-5	7,15-Dihydroxydehydroabietic acid methyl ester	18-F2	346.461	10(μ)M	DMSO
NCRC24804	BBP01534	40672-47-7	Taxifolin 3-O-beta-D-xylopyranoside	18-F3	436.366	10(μ)M	DMSO
NCRC24805	BBP01535	14371-10-9	Cinnamaldehyde	18-F4	132.159	10(μ)M	DMSO
NCRC24806	BBP01536	614-82-4	2,4-Dihydroxyphenylacetic acid	18-F5	168.147	10(μ)M	DMSO
NCRC24807	BBP01537	130-86-9	Protopine	18-F6	353.369	10(μ)M	DMSO
NCRC24808	BBP01538	213329-46-5	4,15-Dihydroxy-18-nor-8,11,13-abietatrien-7-one	18-F7	302.408	10(μ)M	DMSO

NCRC24809	BBP01539	4481-62-3	Betulonic acid	18-F8	454.684	10(μ)M	DMSO
NCRC24810	BBP00099	No	7-Geranyloxy-5-methoxycoumarin	18-F9	328.402	10(μ)M	DMSO
NCRC24811	BBP00105	1228175-65-2	8-Geranyloxy-5,7-dimethoxycoumarin	18-F10	358.428	10(μ)M	DMSO
NCRC24812	BBP01541	827319-50-6	Perforatumone	18-F11	552.784	10(μ)M	DMSO
NCRC24813	BBP01542	189109-45-3	Byzantionoside B	18-G2	372.453	10(μ)M	DMSO
NCRC24814	BBP01543	1138156-77-0	N-p-coumaroyl-N'-caffeoylputrescine	18-G3	396.436	10(μ)M	DMSO
NCRC24815	BBP01544	21499-24-1	Agrimonolide	18-G4	314.333	10(μ)M	DMSO
NCRC24816	BBP01545	132951-90-7	Macrocarpal A	18-G5	472.614	10(μ)M	DMSO
NCRC24817	BBP01546	105181-06-4	9-Oxoageraphorone	18-G6	234.334	10(μ)M	DMSO
NCRC24818	BBP01547	62356-47-2	Isotschimgin	18-G7	274.355	10(μ)M	DMSO
NCRC24819	BBP01548	1042143-83-8	Yunnandaphninine G	18-G8	469.699	10(μ)M	DMSO
NCRC24820	BBP01549	82442-48-6	Arteannuin A	18-G9	206.281	10(μ)M	DMSO
NCRC24821	BBP01550	64929-59-5	Sendanolactone	18-G10	466.652	10(μ)M	DMSO
NCRC24822	BBP01551	6858-85-1	Prometaphanine	18-G11	359.416	10(μ)M	DMSO
NCRC24823	BBP01552	96552-59-9	5,7-Dihydroxy-2-isopropylchromone	18-H2	220.221	10(μ)M	DMSO
NCRC24824	BBP01554	162602-04-2	3-Hydroxy-5,7-dimethoxy-3',4'-methylenedioxyflavan	18-H3	330.332	10(μ)M	DMSO
NCRC24825	BBP01555	10083-24-6	Piceatannol	18-H4	244.243	10(μ)M	DMSO
NCRC24826	BBP01556	No	No	18-H5	510.575	10(μ)M	DMSO
NCRC24827	BBP01557	No	No	18-H6	482.565	10(μ)M	DMSO
NCRC24828	BBP01558	509077-91-2	Taiwanhomoflavone B	18-H7	568.527	10(μ)M	DMSO
NCRC24829	BBP01559	17622-86-5	Nervogenic acid	18-H8	274.355	10(μ)M	DMSO
NCRC24830	BBP01560	26121-56-2	Picrasin B	18-H9	376.443	10(μ)M	DMSO
NCRC24831	BBP01561	104777-61-9	3alpha-Akebonoic acid	18-H10	440.658	10(μ)M	DMSO
NCRC24832	BBP01562	246266-38-6	3-Geranyl-4-methoxybenzoic acid	18-H11	288.381	10(μ)M	DMSO
NCRC40355	BBP01563	151731-50-9	2,2-Dimethyl-8-prenylchromene 6-carboxylic acid	19-A2	272.339	10(μ)M	DMSO
NCRC40356	BBP01564	19775-48-5	Daphmacrine	19-A3	511.736	10(μ)M	DMSO
NCRC40357	BBP01565	619326-75-9	Deoxyisocalyciphylline B	19-A4	341.487	10(μ)M	DMSO
NCRC40358	BBP01566	341971-45-7	Actinidic acid	19-A5	486.683	10(μ)M	DMSO
NCRC40359	BBP01568	149-32-6	Erythritol	19-A6	122.12	10(μ)M	DMSO
NCRC40360	BBP01569	95732-59-5	Hedyotisol A	19-A7	810.837	10(μ)M	DMSO
NCRC40361	BBP01570	60008-01-7	Oblongine	19-A8	314.398	10(μ)M	DMSO
NCRC40362	BBP01573	104055-79-0	No	19-A9	324.37	10(μ)M	DMSO
NCRC40363	BBP01574	206757-32-6	Pedatisectine F	19-A10	214.218	10(μ)M	DMSO
NCRC40364	BBP01575	6147-11-1	alpha-Mangostin	19-A11	410.46	10(μ)M	DMSO
NCRC40365	BBP01576	315236-68-1	Sutherlandin trans-p-coumarate	19-B2	421.398	10(μ)M	DMSO
NCRC40366	BBP01578	501-94-0	2-(4-Hydroxyphenyl)ethanol	19-B3	138.164	10(μ)M	DMSO
NCRC40367	BBP01580	188300-19-8	Massonianoside B	19-B4	492.516	10(μ)M	DMSO
NCRC40368	BBP01582	222629-77-8	Antidesmone	19-B5	319.438	10(μ)M	DMSO
NCRC40369	BBP01583	81678-46-8	3-Dehydro-15-deoxoeucosterol	19-B6	456.657	10(μ)M	DMSO
NCRC40370	BBP01584	53538-13-9	Apigenin 7-O-methylglucuronide	19-B7	460.388	10(μ)M	DMSO
NCRC40371	BBP01585	101140-06-1	3,8''-Biapigenin	19-B8	538.458	10(μ)M	DMSO
NCRC40372	BBP01586	75775-36-9	Cedrusin	19-B9	346.374	10(μ)M	DMSO
NCRC40373	BBP01588	128397-41-1	Hydroprotopine	19-B10	354.376	10(μ)M	DMSO
NCRC40374	BBP01589	31271-07-5	gamma-Mangostin	19-B11	396.433	10(μ)M	DMSO
NCRC40375	BBP01590	81241-53-4	15-Deoxoeucosterol	19-C2	458.673	10(μ)M	DMSO
NCRC40376	BBP01591	26063-95-6	1-Isomangostin hydrate	19-C3	428.475	10(μ)M	DMSO
NCRC40377	BBP01592	76689-98-0	1-Deacetylnimbolinin B	19-C4	584.697	10(μ)M	DMSO
NCRC40378	BBP01593	33390-42-0	Gartanin	19-C5	396.433	10(μ)M	DMSO
NCRC40379	BBP01594	62498-83-3	3-O-Acetyloleanderolide	19-C6	514.736	10(μ)M	DMSO
NCRC40380	BBP01595	27391-16-8	threo-Guaiacylglycerol	19-C7	214.215	10(μ)M	DMSO

NCRC40381	BBP01596	247078-43-9	Daphnezomine B	19-C8	375.545	10(μ)M	DMSO
NCRC40382	BBP01597	57420-46-9	8-O-Acetylshanzhiside methyl ester	19-C9	448.418	10(μ)M	DMSO
NCRC40383	BBP01598	26063-96-7	3-Isomangostin hydrate	19-C10	428.475	10(μ)M	DMSO
NCRC40384	BBP01600	61775-19-7	5-O-Methylnaringenin	19-C11	286.279	10(μ)M	DMSO
NCRC40385	BBP01601	72396-01-1	Yuheinoside	19-D2	360.356	10(μ)M	DMSO
NCRC40386	BBP01602	34366-34-2	Isoapetalic acid	19-D3	388.454	10(μ)M	DMSO
NCRC40387	BBP01603	925705-36-8	3-Isomangostin hydrate formate	19-D4	456.485	10(μ)M	DMSO
NCRC40388	BBP01604	5273-86-9	beta-Asarone	19-D5	208.254	10(μ)M	DMSO
NCRC40389	BBP01605	21698-44-2	Shyobunone	19-D6	220.35	10(μ)M	DMSO
NCRC40390	BBP01606	52706-07-7	Scillascillin	19-D7	312.274	10(μ)M	DMSO
NCRC40391	BBP01607	52096-50-1	2-Hydroxy-7-O-methylscillascillin	19-D8	342.3	10(μ)M	DMSO
NCRC40392	BBP01608	30220-43-0	Effusanin A	19-D9	348.433	10(μ)M	DMSO
NCRC40393	BBP01609	1126-61-0	4-Allylpyrocatechol	19-D10	150.174	10(μ)M	DMSO
NCRC40394	BBP01610	119725-20-1	Fupenzic acid	19-D11	484.667	10(μ)M	DMSO
NCRC40395	BBP01611	217466-37-0	1-Hydroxy-2-oxopomolic acid	19-E2	502.683	10(μ)M	DMSO
NCRC40396	BBP01612	259653-54-8	5-Hydroxy-7-methoxy-3-(4-hydroxybenzylidene)chroman-4-one	19-E3	298.29	10(μ)M	DMSO
NCRC40397	BBP01614	303008-81-3	Pandamarilactonine B	19-E4	317.38	10(μ)M	DMSO
NCRC40398	BBP01754	38412-82-7	3,4,4',7-Tetrahydroxyflavan	19-E5	274.269	10(μ)M	DMSO
NCRC40399	BBP01616	90582-44-8	Sorghumol	19-E6	426.717	10(μ)M	DMSO
NCRC40400	BBP01617	90582-47-1	Sorghumol acetate	19-E7	468.754	10(μ)M	DMSO
NCRC40401	BBP01618	34168-56-4	Catalponol	19-E8	230.302	10(μ)M	DMSO
NCRC40402	BBP01620	1221262-77-6	Meliasenin B	19-E9	468.668	10(μ)M	DMSO
NCRC40403	BBP01621	No	12-Ethoxyabietic acid	19-E10	346.504	10(μ)M	DMSO
NCRC40404	BBP01623	34274-91-4	Gardneramine	19-E11	412.479	10(μ)M	DMSO
NCRC40405	BBP01624	20716-98-7	Norlichexanthone	19-F2	258.226	10(μ)M	DMSO
NCRC40406	BBP01625	24173-71-5	Kobusone	19-F3	222.323	10(μ)M	DMSO
NCRC40407	BBP01627	22478-65-5	18-Norabieta-8,11,13-trien-4-ol	19-F4	272.425	10(μ)M	DMSO
NCRC40408	BBP01628	107160-24-7	Pyrocincholic acid methyl ester	19-F5	456.7	10(μ)M	DMSO
NCRC40409	BBP01629	4339-72-4	3-O-Acetyloleanolic acid	19-F6	498.737	10(μ)M	DMSO
NCRC40410	BBP01630	6909-19-9	2-Caren-10-ol	19-F7	152.233	10(μ)M	DMSO
NCRC40411	BBP01631	142647-71-0	Macrocarpal D	19-F8	472.614	10(μ)M	DMSO
NCRC40412	BBP01632	773850-91-2	Drimiopsin D	19-F9	318.278	10(μ)M	DMSO
NCRC40413	BBP01633	3682-02-8	Isohemiphloin	19-F10	434.393	10(μ)M	DMSO
NCRC40414	BBP01635	2259-07-6	Epifriedelanol acetate	19-F11	470.77	10(μ)M	DMSO
NCRC40415	BBP01636	3772-55-2	Dehydroabietinol	19-G2	286.452	10(μ)M	DMSO
NCRC40416	BBP01637	119725-19-8	2-Epitormentic acid	19-G3	488.699	10(μ)M	DMSO
NCRC40417	BBP01639	85051-41-8	Norviburtinal	19-G4	146.143	10(μ)M	DMSO
NCRC40418	BBP01640	33980-71-1	7-Oxodehydroabietinol	19-G5	300.435	10(μ)M	DMSO
NCRC40419	BBP01641	34818-83-2	4'-Demethyleucomin	19-G6	284.263	10(μ)M	DMSO
NCRC40420	BBP01642	107585-77-3	4'-Demethyl-3,9-dihydroeucomin	19-G7	286.279	10(μ)M	DMSO
NCRC40421	BBP01643	1585-68-8	Catalpalactone	19-G8	258.269	10(μ)M	DMSO
NCRC40422	BBP01644	221899-21-4	Macrocarpal N	19-G9	486.597	10(μ)M	DMSO
NCRC40423	BBP01645	142628-54-4	Macrocarpal E	19-G10	472.614	10(μ)M	DMSO
NCRC40424	BBP01646	773850-90-1	Drimiopsin C	19-G11	288.252	10(μ)M	DMSO
NCRC40425	BBP01647	1740-19-8	Dehydroabietic acid	19-H2	300.435	10(μ)M	DMSO
NCRC40426	BBP01648	35897-92-8	Ligustroside	19-H3	524.514	10(μ)M	DMSO
NCRC40427	BBP01649	25645-19-6	Preisocalamendiol	19-H4	220.35	10(μ)M	DMSO
NCRC40428	BBP01650	56473-67-7	4,9-Dihydroxy-alpha-lapachone	19-H5	274.269	10(μ)M	DMSO
NCRC40429	BBP01651	35241-80-6	9-Methoxy-alpha-lapachone	19-H6	272.296	10(μ)M	DMSO
NCRC40430	BBP01652	57906-31-7	19-Nor-4-hydroxyabieta-8,11,13-trien-7-one	19-H7	286.409	10(μ)M	DMSO
NCRC40431	BBP01653	6380-24-1	cis-Methylisoeugenol	19-H8	178.228	10(μ)M	DMSO

NCRC40432	BBP01654	32602-81-6	Kaempferol 3-neohesperidoside	19-H9	594.518	10(μ)M	DMSO
NCRC40433	BBP01655	857672-34-5	Longistylumphylline A	19-H10	367.481	10(μ)M	DMSO
NCRC40434	BBP01656	770721-33-0	6-Feruloylcatalpol	19-H11	538.498	10(μ)M	DMSO
NCRC40435	BBP01657	2189-80-2	Taraxeryl acetate	20-A2	468.754	10(μ)M	DMSO
NCRC40436	BBP01658	107585-75-1	3'-Hydroxy-3,9-dihydroeucomin	20-A3	316.305	10(μ)M	DMSO
NCRC40437	BBP01659	7362-39-2	p-Coumaric acid ethyl ester	20-A4	192.211	10(μ)M	DMSO
NCRC40438	BBP01660	874201-05-5	Daphnilongeranin A	20-A5	383.481	10(μ)M	DMSO
NCRC40439	BBP01661	175556-08-8	Longifloroside A	20-A6	534.552	10(μ)M	DMSO
NCRC40440	BBP01662	1159913-80-0	Abiesadine N	20-A7	330.461	10(μ)M	DMSO
NCRC40441	BBP01663	922522-15-4	Daphnilongeridine	20-A8	513.752	10(μ)M	DMSO
NCRC40442	BBP01665	4666-84-6	Cryptomeridiol	20-A9	240.382	10(μ)M	DMSO
NCRC40443	BBP01666	6736-85-2	Catalposide	20-A10	482.435	10(μ)M	DMSO
NCRC40444	BBP01667	179388-53-5	Macrocarpal H	20-A11	472.614	10(μ)M	DMSO
NCRC40445	BBP01669	No	Quercitrin 3',4',2",3",4"-pentaacetate	20-B2	658.56	10(μ)M	DMSO
NCRC40446	BBP01670	38916-91-5	erythro-Guaiacylglycerol	20-B3	214.215	10(μ)M	DMSO
NCRC40447	BBP01671	524-12-9	Wedelolactone	20-B4	314.246	10(μ)M	DMSO
NCRC40448	BBP01672	129488-34-2	3,4-Diacetoxycinnamamide	20-B5	263.246	10(μ)M	DMSO
NCRC40449	BBP01673	70389-96-7	4(15),11-Oppositadien-1-ol	20-B6	220.35	10(μ)M	DMSO
NCRC40450	BBP01674	19716-26-8	Stigmasterol glucoside	20-B7	574.831	10(μ)M	DMSO
NCRC40451	BBP01675	15486-24-5	Eleutheroside C	20-B8	208.209	10(μ)M	DMSO
NCRC40452	BBP01676	638-97-1	β-Amyrone	20-B9	424.702	10(μ)M	DMSO
NCRC40453	BBP01677	16566-88-4	Methyl 6-acetoxyangolensate	20-B10	528.591	10(μ)M	DMSO
NCRC40454	BBP01678	67600-94-6	Hydrangenol 8-O-glucoside	20-B11	418.394	10(μ)M	DMSO
NCRC40455	BBP01679	480-47-7	Hydrangenol	20-C2	256.253	10(μ)M	DMSO
NCRC40456	BBP01680	113270-98-7	(3R)-Hydrangenol 8-O-glucoside pentaacetate	20-C3	628.577	10(μ)M	DMSO
NCRC40457	BBP01682	112047-91-3	p-Vinylphenyl O-[beta-D-apiofuranosyl-(1-6)]-beta-D-glucopyranoside	20-C4	414.404	10(μ)M	DMSO
NCRC40458	BBP01683	81968-62-9	4(15),5,10(14)-Germacratrien-1-	20-C5	220.35	10(μ)M	DMSO
NCRC40459	BBP01684	19865-87-3	Cabralealactone	20-C6	414.621	10(μ)M	DMSO
NCRC40460	BBP01685	32619-42-4	Oleuropein	20-C7	540.514	10(μ)M	DMSO
NCRC40461	BBP01686	22255-07-8	Methyl 6-hydroxyangolensate	20-C8	486.554	10(μ)M	DMSO
NCRC40462	BBP01687	70206-70-1	8alpha-Hydroxy-alpha-gurjunene	20-C9	220.35	10(μ)M	DMSO
NCRC40463	BBP01688	29836-27-9	Shanzhiside	20-C10	392.355	10(μ)M	DMSO
NCRC40464	BBP01689	1220508-29-1	Khayalenoid E	20-C11	526.575	10(μ)M	DMSO
NCRC40465	BBP01690	118627-52-4	Epivogeloside	20-D2	388.366	10(μ)M	DMSO
NCRC40466	BBP01691	327601-97-8	Macrocarpal L	20-D3	472.614	10(μ)M	DMSO
NCRC40467	BBP01692	13059-93-3	alpha-Terthienylmethanol	20-D4	278.413	10(μ)M	DMSO
NCRC40468	BBP01694	4707-33-9	alpha-Lapachone	20-D5	242.27	10(μ)M	DMSO
NCRC40469	BBP01695	15297-92-4	Dehydro-alpha-lapachone	20-D6	240.254	10(μ)M	DMSO
NCRC40470	BBP01696	93078-83-2	8-O-Demethyl-7-O-methyl-3,9-dihydropunctatin	20-D7	316.305	10(μ)M	DMSO
NCRC40471	BBP01697	1229005-35-9	Tatarinoid A	20-D8	240.252	10(μ)M	DMSO
NCRC40472	BBP01700	680617-50-9	Megastigm-7-ene-3,5,6,9-tetraol	20-D9	244.327	10(μ)M	DMSO
NCRC40473	BBP01701	1390-72-3	Catalpin	20-D10	322.31	10(μ)M	DMSO
NCRC40474	BBP01703	4460-86-0	2,4,5-Trimethoxybenzaldehyde	20-D11	196.2	10(μ)M	DMSO
NCRC40475	BBP01705	326594-34-7	Fraxamoside	20-E2	538.498	10(μ)M	DMSO
NCRC40476	BBP01706	178600-68-5	Oleoside	20-E3	390.339	10(μ)M	DMSO
NCRC40477	BBP01707	1092555-02-6	Paxiphylline D	20-E4	383.481	10(μ)M	DMSO
NCRC40478	BBP01708	35833-70-6	Cabraleahydroxylactone acetate	20-E5	458.673	10(μ)M	DMSO
NCRC40479	BBP01709	480-23-9	Orobol	20-E6	286.236	10(μ)M	DMSO
NCRC40480	BBP01710	477-57-6	Isotetrandrine	20-E7	622.75	10(μ)M	DMSO
NCRC40481	BBP01711	478-61-5	Berbamine	20-E8	608.723	10(μ)M	DMSO

NCRC40482	BBP01712	1220891-22-4	3,4-Dihydro-3,4-dihydroxynaphthalen-1(2H)-one	20-E9	178.185	10(μ)M	DMSO
NCRC40483	BBP01713	22333-58-0	9-Hydroxy-alpha-lapachone	20-E10	258.269	10(μ)M	DMSO
NCRC40484	BBP01714	1226-22-8	Garbanzol	20-E11	272.253	10(μ)M	DMSO
NCRC40485	BBP01717	16274-33-2	3,4-Dihydro-2,2-dimethyl-2H-naphtho[1,2-b]pyran	20-F2	212.287	10(μ)M	DMSO
NCRC40486	BBP01718	16274-34-3	1-Hydroxy-2-prenylnaphthalene	20-F3	212.287	10(μ)M	DMSO
NCRC40487	BBP01719	1189801-51-1	1-O-Deacetyl-2alpha-hydroxykhayanolide E	20-F4	532.536	10(μ)M	DMSO
NCRC40488	BBP01720	185414-25-9	Corchoionoside C	20-F5	386.437	10(μ)M	DMSO
NCRC40489	BBP01721	133538-77-9	Apigenin 4'-O-rhamnoside	20-F6	416.378	10(μ)M	DMSO
NCRC40490	BBP01722	No	Chloranthol ide B	20-F7	246.302	10(μ)M	DMSO
NCRC40491	BBP01725	173991-81-6	22-Hydroxy-3-oxo-12-ursen-30-oic acid	20-F8	470.684	10(μ)M	DMSO
NCRC40492	BBP01726	89199-94-0	Fraxiresinol 1-O-glucoside	20-F9	566.551	10(μ)M	DMSO
NCRC40493	BBP01727	142542-89-0	Cimidahurinine	20-F10	316.304	10(μ)M	DMSO
NCRC40494	BBP01728	577976-26-2	4-(2-Hydroxy-1-methoxyethyl)-1,2-benzenediol	20-F11	184.189	10(μ)M	DMSO
NCRC40495	BBP01729	218290-59-6	Macrocarpal K	20-G2	472.614	10(μ)M	DMSO
NCRC40496	BBP01730	911714-91-5	Chrysothol	20-G3	238.366	10(μ)M	DMSO
NCRC40497	BBP01731	483-91-0	Calycanthoside	20-G4	384.335	10(μ)M	DMSO
NCRC40498	BBP01732	13040-46-5	Paulownin	20-G5	370.353	10(μ)M	DMSO
NCRC40499	BBP01733	265644-24-4	3-Hydroxycatalponol	20-G6	246.302	10(μ)M	DMSO
NCRC40500	BBP01734	6713-27-5	Moronic acid	20-G7	454.684	10(μ)M	DMSO
NCRC40501	BBP01735	93-39-0	Skimmin	20-G8	324.283	10(μ)M	DMSO
NCRC40502	BBP01737	152110-17-3	Teuclatriol	20-G9	256.381	10(μ)M	DMSO
NCRC40503	BBP01738	10391-09-0	Nodosin	20-G10	362.417	10(μ)M	DMSO
NCRC40504	BBP01740	3198-49-0	Ethyl glucoside	20-G11	208.209	10(μ)M	DMSO
NCRC40505	BBP01741	301530-12-1	Seneganolide	20-H2	470.512	10(μ)M	DMSO
NCRC40506	BBP01742	90852-99-6	Ptelatoside B	20-H3	428.43	10(μ)M	DMSO
NCRC40507	BBP01743	941227-27-6	1,3,5-Cadinatriene-3,8-diol	20-H4	234.334	10(μ)M	DMSO
NCRC40508	BBP01744	77949-42-9	Longikaurin E	20-H5	390.47	10(μ)M	DMSO
NCRC40509	BBP01745	276870-26-9	Megastigm-7-ene-3,5,6,9-tetraol	20-H6	244.327	10(μ)M	DMSO
NCRC40510	BBP01746	No	Catalponol methylthiomethyl	20-H7	290.42	10(μ)M	DMSO
NCRC40511	BBP01747	3568-90-9	Deoxylapachol	20-H8	226.27	10(μ)M	DMSO
NCRC40512	BBP01750	72514-90-0	Specioside	20-H9	508.472	10(μ)M	DMSO
NCRC40513	BBP01751	476682-97-0	6,9,10-Trihydroxy-7-megastigmen-3-one	20-H10	242.311	10(μ)M	DMSO
NCRC40514	BBP01752	179388-54-6	Macrocarpal I	20-H11	490.629	10(μ)M	DMSO
NCRC40515	BBP01756	179603-47-5	Macrocarpal J	21-A2	490.629	10(μ)M	DMSO
NCRC40516	BBP01758	80434-33-9	gamma-Diasarone	21-A3	416.507	10(μ)M	DMSO
NCRC40517	BBP01760	130855-22-0	ent-kaurane-3,16,17-triol	21-A4	322.482	10(μ)M	DMSO
NCRC40518	BBP01761	1043629-23-7	Tetrahydroxysqualene	21-A5	474.716	10(μ)M	DMSO
NCRC40519	BBP01764	54081-48-0	Isoastilbin	21-A6	450.393	10(μ)M	DMSO
NCRC40520	BBP01765	1802-12-6	Phytolaccagenin	21-A7	532.709	10(μ)M	DMSO
NCRC40521	BBP01766	112667-09-1	Erigeside C	21-A8	360.313	10(μ)M	DMSO
NCRC40522	BBP01767	137941-45-8	Arillatose B	21-A9	518.465	10(μ)M	DMSO
NCRC40523	BBP01768	54354-62-0	Decarine	21-A10	319.311	10(μ)M	DMSO
NCRC40524	BBP01769	No	Glochidionionol C	21-A11	224.296	10(μ)M	DMSO
NCRC40525	BBP01771	5027-76-9	Oleuropeic acid	21-B2	184.232	10(μ)M	DMSO
NCRC40526	BBP01772	1187303-40-7	Cuniloside B	21-B3	512.59	10(μ)M	DMSO
NCRC40527	BBP01774	577-56-0	2-Acetylbenzoic acid	21-B4	164.158	10(μ)M	DMSO
NCRC40528	BBP01775	54299-52-4	2',4'-Dihydroxy-3',6'-dimethoxydihydrochalcone	21-B5	302.322	10(μ)M	DMSO
NCRC40529	BBP01776	35878-41-2	Vestitol	21-B6	272.296	10(μ)M	DMSO

NCRC40530	BBP01778	58436-28-5	Dihydroresveratrol	21-B7	230.259	10(μ)M	DMSO
NCRC40531	BBP01779	100432-87-9	Dihydroresveratrol 3-O-glucoside	21-B8	392.4	10(μ)M	DMSO
NCRC40532	BBP01780	67023-81-8	Ohchinin acetate	21-B9	644.751	10(μ)M	DMSO
NCRC40533	BBP01781	25330-21-6	Isocalamendiol	21-B10	238.366	10(μ)M	DMSO
NCRC40534	BBP01782	493-95-8	Savinin	21-B11	352.337	10(μ)M	DMSO
NCRC40535	BBP01785	137887-25-3	6-O-Feruloylglucose	21-C2	356.325	10(μ)M	DMSO
NCRC40536	BBP01786	155418-97-6	4,5-Dihydroblumenol A	21-C3	226.312	10(μ)M	DMSO
NCRC40537	BBP01788	510-30-5	Echinocystic acid	21-C4	472.7	10(μ)M	DMSO
NCRC40538	BBP01789	93767-25-0	Jangomolide	21-C5	468.496	10(μ)M	DMSO
NCRC40539	BBP01790	36190-95-1	3'-O-Methylorobol	21-C6	300.263	10(μ)M	DMSO
NCRC40540	BBP01791	75590-33-9	Kaerophyllin	21-C7	368.38	10(μ)M	DMSO
NCRC40541	BBP01792	No	Chloranthlolide A	21-C8	244.286	10(μ)M	DMSO
NCRC40542	BBP01793	164022-75-7	Clerodenoside A	21-C9	736.714	10(μ)M	DMSO
NCRC40543	BBP01794	29838-67-3	Astilbin	21-C10	450.393	10(μ)M	DMSO
NCRC40544	BBP01795	147517-06-4	Thunberginol C	21-C11	272.253	10(μ)M	DMSO
NCRC40545	BBP01797	35833-72-8	3-Epicabraleahydroxylactone	21-D2	416.636	10(μ)M	DMSO
NCRC40546	BBP01799	155060-48-3	24,25-Dihydroxycycloartan-3-one	21-D3	458.716	10(μ)M	DMSO
NCRC40547	BBP01800	479-91-4	Casticin	21-D4	374.341	10(μ)M	DMSO
NCRC40548	BBP01801	831222-78-7	5-Hydroxymethyl-7-methoxybenzofuran	21-D5	178.185	10(μ)M	DMSO
NCRC40549	BBP01802	118930-92-0	5-(3-Hydroxypropyl)-7-methoxybenzofuran	21-D6	206.238	10(μ)M	DMSO
NCRC40550	BBP01803	48236-96-0	Tetrahydroamentoflavone	21-D7	542.49	10(μ)M	DMSO
NCRC40551	BBP01804	No	5,6-O-Isopropylidene-phlorigidoside B	21-D8	504.482	10(μ)M	DMSO
NCRC40552	BBP01805	36052-37-6	Alpinetin	21-D9	270.28	10(μ)M	DMSO
NCRC40553	BBP01806	No	Chloranthlolide D	21-D10	262.301	10(μ)M	DMSO
NCRC40554	BBP01808	1231208-53-9	4,8-Dihydroxyeudesm-7(11)-en-12,8-olide	21-D11	266.333	10(μ)M	DMSO
NCRC40555	BBP01810	1020074-97-8	20,24-Epoxy-24-methoxy-23(24-25)abeo-dammaran-3-one	21-E2	472.743	10(μ)M	DMSO
NCRC40556	BBP01811	501-96-2	Rhododendrol	21-E3	166.217	10(μ)M	DMSO
NCRC40557	BBP01813	5096-57-1	Canadine	21-E4	339.385	10(μ)M	DMSO
NCRC40558	BBP01814	No	10-O-Acetylisocalamendiol	21-E5	280.402	10(μ)M	DMSO
NCRC40559	BBP01816	157659-20-6	Prenylpiperitol	21-E6	424.486	10(μ)M	DMSO
NCRC40560	BBP01819	64421-28-9	Shanzhiside methyl ester	21-E7	406.382	10(μ)M	DMSO
NCRC40561	BBP01820	70191-83-2	Isotetrandrine N-2'-oxide	21-E8	638.749	10(μ)M	DMSO
NCRC40562	BBP01821	678138-59-5	2,3,2",3"-Tetrahydroochnaflavone	21-E9	542.49	10(μ)M	DMSO
NCRC40563	BBP01823	182138-70-1	Nyssoside	21-E10	490.371	10(μ)M	DMSO
NCRC40564	BBP01825	14965-20-9	Chrysosplenol D	21-E11	360.315	10(μ)M	DMSO
NCRC40565	BBP01826	366450-46-6	Semialactone	21-F2	468.668	10(μ)M	DMSO
NCRC40566	BBP01827	72061-63-3	Sepinol	21-F3	318.278	10(μ)M	DMSO
NCRC40567	BBP01828	61186-24-1	Grandifloroside	21-F4	538.498	10(μ)M	DMSO
NCRC40568	BBP01829	126223-29-8	Agrimonolide 6-O-glucoside	21-F5	476.473	10(μ)M	DMSO
NCRC40569	BBP01830	197307-49-6	11-Dehydroxygrevilloside B	21-F6	342.384	10(μ)M	DMSO
NCRC40570	BBP01831	57586-98-8	Cycloartane-3,24,25-triol	21-F7	460.732	10(μ)M	DMSO
NCRC40571	BBP01832	20725-03-5	Fustin	21-F8	288.252	10(μ)M	DMSO
NCRC40572	BBP01834	29748-10-5	Loganetin	21-F9	228.242	10(μ)M	DMSO
NCRC40573	BBP01836	1253740-09-8	6-Acetonyl-N-methyl-dihydrodecarine	21-F10	391.417	10(μ)M	DMSO
NCRC40574	BBP01838	67828-62-0	Ethyl 2,4-dihydroxyphenylacetate	21-F11	196.2	10(μ)M	DMSO
NCRC40575	BBP01839	117479-87-5	Sesamoside	21-G2	420.365	10(μ)M	DMSO
NCRC40576	BBP01841	20243-59-8	Hydroxygenkwanin	21-G3	300.263	10(μ)M	DMSO
NCRC40577	BBP01842	1242085-06-8	12-Ursene-3,16,22-triol	21-G4	458.716	10(μ)M	DMSO
NCRC40578	BBP01843	33606-81-4	Myricanol	21-G5	358.428	10(μ)M	DMSO

NCRC40579	BBP01844	127-22-0	Taraxerol	21-G6	426.717	10(μ)M	DMSO
NCRC40580	BBP01845	4382-33-6	Dihydrorobinetin	21-G7	304.252	10(μ)M	DMSO
NCRC40581	BBP01846	520-33-2	Hesperetin	21-G8	302.279	10(μ)M	DMSO
NCRC40582	BBP01847	75069-59-9	20,24-Dihydroxydammar-25-en-3-one	21-G9	458.716	10(μ)M	DMSO
NCRC40583	BBP01848	4382-34-7	Robtin	21-G10	288.252	10(μ)M	DMSO
NCRC40584	BBP01849	83529-71-9	Anisofolin A	21-G11	724.663	10(μ)M	DMSO
NCRC40585	BBP01850	84-79-7	Lapachol	21-H2	242.27	10(μ)M	DMSO
NCRC40586	BBP01851	82003-90-5	Daurichromenic acid	21-H3	370.482	10(μ)M	DMSO
NCRC40587	BBP01853	20133-19-1	1-(3,4-Dimethoxyphenyl)propane-1,2-diol	21-H4	212.242	10(μ)M	DMSO
NCRC40588	BBP01854	35349-68-9	9-Hydroxycalabaxanthone	21-H5	408.444	10(μ)M	DMSO
NCRC40589	BBP01855	85372-70-9	5,19-Epoxy-19,25-dimethoxycucurbita-6,23-dien-3-	21-H6	500.753	10(μ)M	DMSO
NCRC40590	BBP01856	104953-08-4	Ethyl beta-D-ribo-hex-3-ulopyranoside	21-H7	206.193	10(μ)M	DMSO
NCRC40591	BBP01857	119767-00-9	3-Furfuryl 2-pyrrolecarboxylate	21-H8	191.183	10(μ)M	DMSO
NCRC40592	BBP01858	1079941-35-7	Myricananin A	21-H9	344.402	10(μ)M	DMSO
NCRC40593	BBP01859	26296-50-4	Ficaprenol 11	21-H10	767.303	10(μ)M	DMSO
NCRC40594	BBP01860	268541-26-0	Triptohypol F	21-H11	456.743	10(μ)M	DMSO
NCRC40595	BBP01861	168293-10-5	C-Veratroylglycol	22-A2	212.199	10(μ)M	DMSO
NCRC40596	BBP01862	No	9,11,13-Octadecatriynoic acid methyl ester	22-A3	286.409	10(μ)M	DMSO
NCRC40597	BBP01863	585534-03-8	Confluentin	22-A4	326.472	10(μ)M	DMSO
NCRC40598	BBP01864	202596-22-3	Calyxin H	22-A5	566.64	10(μ)M	DMSO
NCRC40599	BBP01865	1190225-48-9	Sarcandrone B	22-A6	554.587	10(μ)M	DMSO
NCRC40600	BBP01866	113558-03-5	1,2,3,19-Tetrahydroxy-12-ursen-28-oic acid	22-A7	504.699	10(μ)M	DMSO
NCRC40601	BBP01867	94530-87-7	9(11),12-Oleanadien-3-ol	22-A8	424.702	10(μ)M	DMSO
NCRC40602	BBP01869	62014-81-7	p-Menthane-1,2,8-triol	22-A9	188.264	10(μ)M	DMSO
NCRC40603	BBP01871	41137-85-3	Platyphyllonol	22-A10	314.376	10(μ)M	DMSO
NCRC40604	BBP01872	207446-90-0	Arteannuin M	22-A11	268.349	10(μ)M	DMSO
NCRC40605	BBP01874	944804-58-4	Rhuscholide A	22-B2	462.663	10(μ)M	DMSO
NCRC40606	BBP01875	No	Chloranthólide E	22-B3	278.3	10(μ)M	DMSO
NCRC40607	BBP01876	875585-30-1	ent-14,15-Dinor-13-oxolabda-8(17),11-dien-18-oic acid	22-B4	290.397	10(μ)M	DMSO
NCRC40608	BBP01877	83915-59-7	13-Hydroxylabda-8(17),14-dien-18-oic acid	22-B5	320.466	10(μ)M	DMSO
NCRC40609	BBP01878	1039673-32-9	15-Nor-14-oxolabda-8(17),12-dien-18-oic acid	22-B6	304.424	10(μ)M	DMSO
NCRC40610	BBP01879	30359-01-4	Centrolobol	22-B7	300.392	10(μ)M	DMSO
NCRC40611	BBP01880	56973-65-0	Platyphyllenone	22-B8	296.36	10(μ)M	DMSO
NCRC40612	BBP01881	34316-15-9	Chelerythrine	22-B9	348.371	10(μ)M	DMSO
NCRC40613	BBP01882	58-08-2	Caffeine	22-B10	194.191	10(μ)M	DMSO
NCRC40614	BBP01883	154418-16-3	5,5'-Dimethoxylariciresinol 4-O-glucoside	22-B11	582.594	10(μ)M	DMSO
NCRC40615	BBP01884	22805-15-8	3-(4-Hydroxy-3,5-dimethoxyphenyl) -1,2-propanediol	22-C2	228.242	10(μ)M	DMSO
NCRC40616	BBP01885	116384-26-0	3',4',7-Trimethoxyflavan	22-C3	300.349	10(μ)M	DMSO
NCRC40617	BBP01887	60337-67-9	9-O-Feruloyllariciresinol	22-C4	536.57	10(μ)M	DMSO
NCRC40618	BBP01888	5373-11-5	Luteolin 7-glucoside	22-C5	448.377	10(μ)M	DMSO
NCRC40619	BBP01889	61303-13-7	Isoacteoside	22-C6	624.587	10(μ)M	DMSO
NCRC40620	BBP01890	14215-86-2	Sweroside	22-C7	358.34	10(μ)M	DMSO
NCRC40621	BBP01891	5289-74-7	20-Hydroxyecdysone	22-C8	480.634	10(μ)M	DMSO
NCRC40622	BBP01892	848669-09-0	Stigmasta-4,22,25-trien-3-one	22-C9	408.659	10(μ)M	DMSO

NCRC40623	BBP01893	848669-08-9	Stigmasta-4,25-dien-3-one	22-C10	410.675	10(μ)M	DMSO
NCRC40624	BBP01895	23141-27-7	Vincosamide	22-C11	498.525	10(μ)M	DMSO
NCRC40625	BBP01896	852638-61-0	Heteronoside	22-D2	790.675	10(μ)M	DMSO
NCRC40626	BBP01897	148044-47-7	25-Hydroxycycloart-23-en-3-one	22-D3	440.701	10(μ)M	DMSO
NCRC40627	BBP01898	490-31-3	Robinetin	22-D4	302.236	10(μ)M	DMSO
NCRC40628	BBP01899	32492-74-3	Myricanone	22-D5	356.412	10(μ)M	DMSO
NCRC40629	BBP01900	17884-88-7	Myricadiol	22-D6	442.717	10(μ)M	DMSO
NCRC40630	BBP01901	70051-38-6	4,10-Aromadendranediol	22-D7	238.366	10(μ)M	DMSO
NCRC40631	BBP01904	81910-39-6	5,19-Epoxy-25-methoxycucurbita-6,23-dien-3-ol	22-D8	470.727	10(μ)M	DMSO
NCRC40632	BBP01905	934739-29-4	Karavilagenin D	22-D9	470.684	10(μ)M	DMSO
NCRC40633	BBP01906	7608-44-8	Artocarpin	22-D10	436.497	10(μ)M	DMSO
NCRC40634	BBP01907	159623-48-0	3,4-Secotirucalla-4(28),7,24-triene-3,26-dioic acid	22-D11	470.684	10(μ)M	DMSO
NCRC40635	BBP01908	33390-41-9	8-Deoxygartanin	22-E2	380.434	10(μ)M	DMSO
NCRC40636	BBP01909	41137-87-5	Hirsutenone	22-E3	328.359	10(μ)M	DMSO
NCRC40637	BBP01910	76035-62-6	3-Epikatonic acid	22-E4	456.7	10(μ)M	DMSO
NCRC40638	BBP01911	55497-79-5	Myriceric acid B	22-E5	634.842	10(μ)M	DMSO
NCRC40639	BBP01912	31298-06-3	Arjunic acid	22-E6	488.699	10(μ)M	DMSO
NCRC40640	BBP01913	164991-53-1	Calyxin B	22-E7	582.64	10(μ)M	DMSO
NCRC40641	BBP01914	906-33-2	Neochlorogenic acid	22-E8	354.309	10(μ)M	DMSO
NCRC40642	BBP01915	69120-07-6	Moracin D	22-E9	308.328	10(μ)M	DMSO
NCRC40643	BBP01916	144629-84-5	22-Hydroxy-3-oxoolean-12-en-29-oic acid	22-E10	470.684	10(μ)M	DMSO
NCRC40644	BBP01917	465-13-4	Neritaloside	22-E11	592.718	10(μ)M	DMSO
NCRC40645	BBP01918	142279-41-2	Shizukaol C	22-F2	634.713	10(μ)M	DMSO
NCRC40646	BBP01919	40957-99-1	Medioresinol	22-F3	388.411	10(μ)M	DMSO
NCRC40647	BBP01921	482-35-9	Isoquercitrin	22-F4	464.376	10(μ)M	DMSO
NCRC40648	BBP01922	No	2,3-Di-O-methylthiomethyleuscaphic acid	22-F5	608.935	10(μ)M	DMSO
NCRC40649	BBP01923	61671-56-5	Neridienone B	22-F6	344.445	10(μ)M	DMSO
NCRC40650	BBP01924	18810-25-8	Odoroside H	22-F7	534.681	10(μ)M	DMSO
NCRC40651	BBP01925	129212-92-6	(2S,3S)-(-)-Glucodistylin	22-F8	466.392	10(μ)M	DMSO
NCRC40652	BBP01926	27661-51-4	Leucoside	22-F9	580.492	10(μ)M	DMSO
NCRC40653	BBP01927	405281-76-7	Dadahol A	22-F10	698.712	10(μ)M	DMSO
NCRC40654	BBP01928	59204-61-4	beta-Dihydroplumericinic acid	22-F11	278.257	10(μ)M	DMSO
NCRC40655	BBP01929	52525-35-6	Quercetin 3-O-robinobioside	22-G2	610.518	10(μ)M	DMSO
NCRC40656	BBP01930	480-36-4	Linarin	22-G3	592.545	10(μ)M	DMSO
NCRC40657	BBP01931	6018-40-2	Corypalmine	22-G4	341.401	10(μ)M	DMSO
NCRC40658	BBP01932	No	Chlorantholide F	22-G5	278.3	10(μ)M	DMSO
NCRC40659	BBP01933	51005-44-8	Minecoside	22-G6	538.498	10(μ)M	DMSO
NCRC40660	BBP01935	56222-03-8	Porson	22-G7	386.438	10(μ)M	DMSO
NCRC40661	BBP01936	34509-52-9	Myricanol triacetate	22-G8	484.538	10(μ)M	DMSO
NCRC40662	BBP01938	136807-41-5	6-O-Cinnamoylcatalpol	22-G9	492.473	10(μ)M	DMSO
NCRC40663	BBP01939	211126-61-3	Rubranol	22-G10	332.391	10(μ)M	DMSO
NCRC40664	BBP01940	956869-95-7	Euscaphin B	22-G11	258.354	10(μ)M	DMSO
NCRC40665	BBP01942	552-57-8	Isorhoifolin	22-H2	578.519	10(μ)M	DMSO
NCRC40666	BBP01943	41137-86-4	Hirsutanonol	22-H3	346.374	10(μ)M	DMSO
NCRC40667	BBP01944	572-31-6	Engeletin	22-H4	434.393	10(μ)M	DMSO
NCRC40668	BBP01945	156368-84-2	Ehretioside B	22-H5	311.287	10(μ)M	DMSO
NCRC40669	BBP01946	144049-72-9	6-O-Syringoylajugol	22-H6	528.503	10(μ)M	DMSO
NCRC40670	BBP01947	62312-55-4	5-Hydroxy-2-pyrrolidinone	22-H7	101.104	10(μ)M	DMSO
NCRC40671	BBP01948	31712-49-9	Hesperetin 7-O-glucoside	22-H8	464.419	10(μ)M	DMSO
NCRC40672	BBP01949	564-14-7	Hop-17(21)-en-3-ol	22-H9	426.717	10(μ)M	DMSO
NCRC40673	BBP01950	511-89-7	Plumieride	22-H10	470.424	10(μ)M	DMSO

NCRC40674	BBP01951	84799-31-5	5-Epilithospermoside	22-H11	329.303	10(μ)M	DMSO
NCRC40675	BBP01952	80416-52-0	13-O-p-Coumaroylplumieride	23-A2	616.567	10(μ)M	DMSO
NCRC40676	BBP01954	15404-80-5	Isonormangostin	23-A3	396.433	10(μ)M	DMSO
NCRC40677	BBP01955	112649-48-6	BR-Xanthone A	23-A4	396.433	10(μ)M	DMSO
NCRC40678	BBP01956	120051-54-9	Meridinol	23-A5	370.353	10(μ)M	DMSO
NCRC40679	BBP01957	147714-71-4	6-O-(3'',4''-Dimethoxycinnamoyl)catalpol	23-A6	552.524	10(μ)M	DMSO
NCRC40680	BBP01958	121710-02-9	6-O-p-Methoxycinnamoylcatalpol	23-A7	522.499	10(μ)M	DMSO
NCRC40681	BBP01959	No	Chlorantholide C	23-A8	246.302	10(μ)M	DMSO
NCRC40682	BBP01960	936006-11-0	5-(6-Hydroxybenzofuran-2-yl)-2-(3-methylbut-1-enyl)benzene-1,3-diol	23-A9	310.344	10(μ)M	DMSO
NCRC40683	BBP01961	39011-92-2	Nuezhenide	23-A10	686.655	10(μ)M	DMSO
NCRC40684	BBP01962	77658-46-9	ent-11,16-Epoxy-15-hydroxykauran-19-oic acid	23-A11	334.45	10(μ)M	DMSO
NCRC40685	BBP01963	149250-48-6	De-4'-O-methylyangambin	23-B2	432.464	10(μ)M	DMSO
NCRC40686	BBP01965	162059-94-1	Myriceric acid C	23-B3	796.984	10(μ)M	DMSO
NCRC40687	BBP01966	6619-95-0	Deacetylxylopic acid	23-B4	318.45	10(μ)M	DMSO
NCRC40688	BBP01967	17526-15-7	Xanthorin	23-B5	300.263	10(μ)M	DMSO
NCRC40689	BBP01969	50932-19-9	Verminoside	23-B6	524.471	10(μ)M	DMSO
NCRC40690	BBP01971	108-46-3	Resorcinol	23-B7	110.111	10(μ)M	DMSO
NCRC40691	BBP01972	1007387-95-2	1-O-Deacetylkhayanolide E	23-B8	516.537	10(μ)M	DMSO
NCRC40692	BBP01973	526-87-4	Conduritol A	23-B9	146.141	10(μ)M	DMSO
NCRC40693	BBP01974	54963-52-9	2-Oxopomolic acid	23-B10	486.683	10(μ)M	DMSO
NCRC40694	BBP01975	69120-06-5	Moracin C	23-B11	310.344	10(μ)M	DMSO
NCRC40695	BBP01976	301-19-9	Robinin	23-C2	740.659	10(μ)M	DMSO
NCRC40696	BBP01977	No	7-O-Demethyl-3-isomangostin hydrate	23-C3	414.448	10(μ)M	DMSO
NCRC40697	BBP01978	90332-92-6	Shizukolidol	23-C4	248.318	10(μ)M	DMSO
NCRC40698	BBP01979	112693-21-7	Oleonuezhenide	23-C5	1073.01	10(μ)M	DMSO
NCRC40699	BBP01983	56486-94-3	Steppogenin	23-C6	288.252	10(μ)M	DMSO
NCRC40700	BBP01984	142474-52-0	Glyasperin A	23-C7	422.47	10(μ)M	DMSO
NCRC40701	BBP01985	13956-29-1	Cannabidiol	23-C8	314.462	10(μ)M	DMSO
NCRC40702	BBP01986	168254-96-4	Evofolin B	23-C9	318.321	10(μ)M	DMSO
NCRC40703	BBP01987	477-84-9	Damnacanthal	23-C10	282.248	10(μ)M	DMSO
NCRC40704	BBP01988	62949-79-5	Mulberrin	23-C11	422.47	10(μ)M	DMSO
NCRC40705	BBP01989	119318-15-9	Olean-12-ene-3,24-diol	23-D2	442.717	10(μ)M	DMSO
NCRC40706	BBP01990	471-66-9	α-Boswellic acid	23-D3	456.7	10(μ)M	DMSO
NCRC40707	BBP01991	189351-15-3	Corchoionol C	23-D4	224.296	10(μ)M	DMSO
NCRC40708	BBP01992	1616-93-9	β-Amyrin acetate	23-D5	468.754	10(μ)M	DMSO
NCRC40709	BBP01993	130838-00-5	Scoparinol	23-D6	426.588	10(μ)M	DMSO
NCRC40710	BBP01994	76472-88-3	Morachalcone A	23-D7	340.37	10(μ)M	DMSO
NCRC40711	BBP01995	39903-21-4	29-Hydroxyfriedelan-3-one	23-D8	442.717	10(μ)M	DMSO
NCRC40712	BBP01996	480-43-3	Isosakuranetin	23-D9	286.279	10(μ)M	DMSO
NCRC40713	BBP01997	7460-43-7	Rubiadin 1-methyl ether	23-D10	268.264	10(μ)M	DMSO
NCRC40714	BBP01999	472-30-0	Masticadienolic acid	23-D11	456.7	10(μ)M	DMSO
NCRC40715	BBP02000	56421-12-6	Methyl eichlerianate	23-E2	488.742	10(μ)M	DMSO
NCRC40716	BBP02001	442851-27-6	Methyl isodrimeninol	23-E3	250.376	10(μ)M	DMSO
NCRC40717	BBP02002	62218-23-9	3-O-Methylducheside A	23-E4	462.36	10(μ)M	DMSO
NCRC40718	BBP02003	532-91-2	Coixol	23-E5	165.146	10(μ)M	DMSO
NCRC40719	BBP02005	6926-14-3	8-O-Acetylharpagide	23-E6	406.382	10(μ)M	DMSO
NCRC40720	BBP02006	77741-58-3	1,7-Dihydroxy-3-methoxy-2-prenylxanthone	23-E7	326.343	10(μ)M	DMSO
NCRC40721	BBP02007	3542-72-1	Norathyriol	23-E8	260.199	10(μ)M	DMSO
NCRC40722	BBP02009	72537-20-3	Polygonal	23-E9	222.323	10(μ)M	DMSO

NCRC40723	BBP02010	117-02-2	Rubiadin	23-E10	254.238	10(μ)M	DMSO
NCRC40724	BBP02011	87480-84-0	Dihydroajugapitin	23-E11	552.654	10(μ)M	DMSO
NCRC40725	BBP02014	62596-29-6	Morusin	23-F2	420.454	10(μ)M	DMSO
NCRC40726	BBP02015	1180-35-4	Acetylepipodophyllotoxin	23-F3	456.442	10(μ)M	DMSO
NCRC40727	BBP02016	5282-14-4	Olean-12-ene-3,11-diol	23-F4	442.717	10(μ)M	DMSO
NCRC40728	BBP02017	124168-04-3	6-O-Vanilloylajugol	23-F5	498.477	10(μ)M	DMSO
NCRC40729	BBP02018	83945-57-7	4-Epicommunic acid	23-F6	302.451	10(μ)M	DMSO
NCRC40730	BBP02019	120-14-9	Veratraldehyde	23-F7	166.174	10(μ)M	DMSO
NCRC40731	BBP02020	3420-72-2	Flavokawain A	23-F8	314.333	10(μ)M	DMSO
NCRC40732	BBP02021	961-29-5	Isoliquiritigenin	23-F9	256.253	10(μ)M	DMSO
NCRC40733	BBP02022	20245-39-0	1,3,7-Trihydroxy-2-	23-F10	312.317	10(μ)M	DMSO
NCRC40734	BBP02023	816456-90-3	Canusesnol A	23-F11	250.333	10(μ)M	DMSO
NCRC40735	BBP02024	60129-63-7	Paniculoside I	23-G2	480.591	10(μ)M	DMSO
NCRC40736	BBP02025	93796-20-4	3-Prenyl-2,4,6-trihydroxybenzophenone	23-G3	298.333	10(μ)M	DMSO
NCRC40737	BBP02026	132586-69-7	15-Demethylplumieride	23-G4	456.397	10(μ)M	DMSO
NCRC40738	BBP02027	3570-40-9	Albaspidin AA	23-G5	404.41	10(μ)M	DMSO
NCRC40739	BBP02031	466-09-1	Uzarigenin	23-G6	374.514	10(μ)M	DMSO
NCRC40740	BBP02032	19186-35-7	Deoxypodophyllotoxin	23-G7	398.406	10(μ)M	DMSO
NCRC40741	BBP02033	479-21-0	Cotoin	23-G8	244.243	10(μ)M	DMSO
NCRC40742	BBP02034	610778-85-3	Isobonducellin	23-G9	282.291	10(μ)M	DMSO
NCRC40743	BBP02035	80508-42-5	Tenacigenin B	23-G10	364.476	10(μ)M	DMSO
NCRC40744	BBP02037	1174017-37-8	Artoheterophyllin B	23-G11	504.571	10(μ)M	DMSO
NCRC40745	BBP02038	No	9-Hydroxycalabaxanthone hydrate	23-H2	426.459	10(μ)M	DMSO
NCRC40746	BBP02039	495-02-3	Auraptene	23-H3	298.376	10(μ)M	DMSO
NCRC40747	BBP02040	90996-27-3	8-Methoxybonducellin	23-H4	312.317	10(μ)M	DMSO
NCRC40748	BBP02041	40456-50-6	Yatein	23-H5	400.422	10(μ)M	DMSO
NCRC40749	BBP02042	26271-33-0	2,3',4,6-Tetrahydroxybenzophenone	23-H6	246.215	10(μ)M	DMSO
NCRC40750	BBP02043	808769-54-2	3,22-Dihydroxyolean-12-en-29-oic acid	23-H7	472.7	10(μ)M	DMSO
NCRC40751	BBP02044	19131-13-6	6-Deoxy-3-O-methyl-β-allopyranosyl (1→4)-β-cymaronic acid δ-lactone	23-H8	320.336	10(μ)M	DMSO
NCRC40752	BBP02045	110-15-6	Succinic acid	23-H9	118.088	10(μ)M	DMSO
NCRC40753	BBP02046	201534-09-0	Triptocallic acid D	23-H10	472.7	10(μ)M	DMSO
NCRC40754	BBP02047	112652-46-7	Fragransin A2	23-H11	344.402	10(μ)M	DMSO
NCRC40755	BBP02048	184587-72-2	Mangostanol	24-A2	426.459	10(μ)M	DMSO
NCRC40756	BBP02050	No	8-Isomulberrin hydrate	24-A3	440.486	10(μ)M	DMSO
NCRC40757	BBP02052	27013-91-8	α-Hederin	24-A4	750.956	10(μ)M	DMSO
NCRC40758	BBP02053	488-76-6	vibo-Quercitol	24-A5	164.156	10(μ)M	DMSO
NCRC40759	BBP02054	3080-20-4	β-Anhydrouzarigenin	24-A6	356.498	10(μ)M	DMSO
NCRC40760	BBP02055	60-82-2	Phloretin	24-A7	274.269	10(μ)M	DMSO
NCRC40761	BBP02059	85122-21-0	2-Methoxystypandrone	24-A8	260.242	10(μ)M	DMSO
NCRC40762	BBP02060	92609-77-3	O-Demethylforbexanthone	24-A9	326.3	10(μ)M	DMSO
NCRC40763	BBP02061	16100-84-8	Dihydroperaksine	24-A10	312.406	10(μ)M	DMSO
NCRC40764	BBP02062	942609-65-6	1,5,15-Tri-O-methylmorindol	24-A11	328.316	10(μ)M	DMSO
NCRC40765	BBP02063	74336-91-7	4',4'''-Di-O-	24-B2	566.511	10(μ)M	DMSO
NCRC40766	BBP02065	51995-99-4	Dehydroespeletone	24-B3	232.275	10(μ)M	DMSO
NCRC40767	BBP02066	6750-10-3	Fuegin	24-B4	266.333	10(μ)M	DMSO
NCRC40768	BBP02067	465-16-7	Oleandrin	24-B5	576.718	10(μ)M	DMSO
NCRC40769	BBP02068	864516-31-4	Nigrolineaxanthone V	24-B6	408.444	10(μ)M	DMSO
NCRC40770	BBP02070	519-34-6	Maclurin	24-B7	262.215	10(μ)M	DMSO
NCRC40771	BBP02071	25577-04-2	1,6,7-Trihydroxyxanthone	24-B8	244.2	10(μ)M	DMSO
NCRC40772	BBP02073	451478-47-0	10-Hydroxydihydroperaksine	24-B9	328.405	10(μ)M	DMSO

NCRC40773	BBP02074	68799-41-7	6-Acetyl-2,2-dimethylchroman-4-one	24-B10	218.248	10(μ)M	DMSO
NCRC40774	BBP02076	118169-27-0	6''-O-Acetylastragalin	24-B11	490.414	10(μ)M	DMSO
NCRC40775	BBP02077	86606-14-6	6-Benzoyl-5,7-dihydroxy-2,2-dimethylchromane	24-C2	298.333	10(μ)M	DMSO
NCRC40776	BBP02078	No	3-(3-Hydroxy-3-methylbutanyl)-2,4,6-trihydroxybenzophenone	24-C3	316.348	10(μ)M	DMSO
NCRC40777	BBP02079	63565-07-1	8-Benzoyl-5,7-dihydroxy-2,2-dimethylchromane	24-C4	298.333	10(μ)M	DMSO
NCRC40778	BBP02080	22136-74-9	Podocarpusflavone A	24-C5	552.485	10(μ)M	DMSO
NCRC40779	BBP02081	62949-93-3	Morusinol	24-C6	438.47	10(μ)M	DMSO
NCRC40780	BBP02082	58469-06-0	N-(2-Hydroxy-4-methoxyphenyl)acetamide	24-C7	181.189	10(μ)M	DMSO
NCRC40781	BBP02083	7121-99-5	Erythroxytriol P	24-C8	324.498	10(μ)M	DMSO
NCRC40782	BBP02085	64032-49-1	Torachrysone 8-O-glucoside	24-C9	408.399	10(μ)M	DMSO
NCRC40783	BBP02091	617722-55-1	Methyl 2-(5-acetyl-2,3-dihydrobenzofuran-2-yl)propenoate	24-C10	246.259	10(μ)M	DMSO
NCRC40784	BBP02092	35943-38-5	Pteroside D	24-C11	410.458	10(μ)M	DMSO
NCRC40785	BBP02093	20675-51-8	Cannabichromene	24-D2	314.462	10(μ)M	DMSO
NCRC40786	BBP02095	22649-04-3	Torachrysone	24-D3	246.259	10(μ)M	DMSO
NCRC40787	BBP02097	617722-56-2	Methyl 2-(6-acetyl-5-hydroxy-2,3-dihydrobenzofuran-2-yl)propenoate	24-D4	262.258	10(μ)M	DMSO
NCRC40788	BBP02099	36450-01-8	6-Hydroxystigmasta-4,22-dien-3-one	24-D5	426.674	10(μ)M	DMSO
NCRC40789	BBP02100	218780-16-6	12-Hydroxyisodrimenin	24-D6	250.333	10(μ)M	DMSO
NCRC40790	BBP02101	22415-24-3	3-Epiturraeanthin	24-D7	514.736	10(μ)M	DMSO
NCRC40791	BBP02103	1025023-05-5	Periglaucine B	24-D8	373.4	10(μ)M	DMSO
NCRC40792	BBP02104	42895-58-9	14-Deoxy-11,12-didehydroandrographolide	24-D9	332.434	10(μ)M	DMSO
NCRC40793	BBP02105	970-74-1	Epigallocatechin	24-D10	306.267	10(μ)M	DMSO
NCRC40794	BBP02106	5631-68-5	3-(2,4-Dihydroxyphenyl)propionic acid	24-D11	182.173	10(μ)M	DMSO
NCRC40795	BBP02107	51828-10-5	2'-O-Methylisoliquiritigenin	24-E2	270.28	10(μ)M	DMSO
NCRC40796	BBP02108	465-99-6	Hederagenin	24-E3	472.7	10(μ)M	DMSO
NCRC40797	BBP02109	110064-50-1	7-Hydroxy-3-(4-hydroxybenzylidene)chroman-4-one	24-E4	268.264	10(μ)M	DMSO
NCRC40798	BBP02110	19888-34-7	Humulene epoxide II	24-E5	220.35	10(μ)M	DMSO
NCRC40799	BBP02112	17422-90-1	Methyl 3-(2,4-dihydroxyphenyl)propionate	24-E6	196.2	10(μ)M	DMSO
NCRC40800	BBP02113	52611-75-3	Epipterosin L	24-E7	264.317	10(μ)M	DMSO
NCRC40801	BBP02114	25654-31-3	Cannabigerol	24-E8	316.478	10(μ)M	DMSO
NCRC40802	BBP02115	144881-19-6	Junipediol B	24-E9	196.2	10(μ)M	DMSO
NCRC40803	BBP02116	3570-62-5	Moslosooflavone	24-E10	298.29	10(μ)M	DMSO
NCRC40804	BBP02117	113981-49-0	5-Hydroxy-7,8-dimethoxyflavanone	24-E11	300.306	10(μ)M	DMSO
NCRC40805	BBP02118	62014-87-3	Helichrysetin	24-F2	286.279	10(μ)M	DMSO
NCRC40806	BBP02119	69804-59-7	2,7-Dihydroxy-2H-1,4-benzoxazin-3(4H)-one	24-F3	181.145	10(μ)M	DMSO
NCRC40807	BBP02120	139561-95-8	Epicannabidiol hydrate	24-F4	332.477	10(μ)M	DMSO
NCRC40808	BBP02122	1083200-79-6	1,7-Bis(4-hydroxyphenyl)hept-1-en-3-one	24-F5	296.36	10(μ)M	DMSO
NCRC40809	BBP02123	158500-59-5	Cnidioside B methyl ester	24-F6	412.388	10(μ)M	DMSO
NCRC40810	BBP02124	No	6'-Hydroxy-7'-ethoxybergamottin	24-F7	400.465	10(μ)M	DMSO

NCRC40811	BBP02125	213552-47-7	Drim-7-ene-11,12-diol acetonide	24-F8	278.43	10(μ)M	DMSO
NCRC40812	BBP02128	221257-06-3	7,4'-Di-O-methylapigenin 5-O-xylosylglucoside	24-F9	592.545	10(μ)M	DMSO
NCRC40813	BBP02129	1088-17-1	Isomeranzin	24-F10	260.285	10(μ)M	DMSO
NCRC40814	BBP02130	74048-71-8	Lancifolin C	24-F11	372.455	10(μ)M	DMSO
NCRC40815	BBP02132	34169-70-5	Pterosin D	24-G2	248.318	10(μ)M	DMSO
NCRC40816	BBP02133	190906-61-7	Triptocallic acid A	24-G3	472.7	10(μ)M	DMSO
NCRC40817	BBP02134	904665-71-0	Daturataturin A aglycone	24-G4	454.598	10(μ)M	DMSO
NCRC40818	BBP02135	221289-31-2	Lethedioside A	24-G5	622.571	10(μ)M	DMSO
NCRC40819	BBP02136	920502-42-7	Tenacigenoside A	24-G6	668.812	10(μ)M	DMSO
NCRC40820	BBP02137	113122-54-6	3-Deoxysappanone B	24-G7	286.279	10(μ)M	DMSO
NCRC40821	BBP02138	495-32-9	Nodakenetin	24-G8	246.259	10(μ)M	DMSO
NCRC40822	BBP02139	449729-89-9	(+)-S-Myricanol glucoside	24-G9	520.569	10(μ)M	DMSO
NCRC40823	BBP02140	6587-37-7	3-Epiglochidiol diacetate	24-G10	526.79	10(μ)M	DMSO
NCRC40824	BBP02141	51857-11-5	7-O-Methyleriodictyol	24-G11	302.279	10(μ)M	DMSO
NCRC40825	BBP02142	60102-29-6	Isosativan	24-H2	286.322	10(μ)M	DMSO
NCRC40826	BBP02143	41347-49-3	Anhydrotuberosin	24-H3	320.339	10(μ)M	DMSO
NCRC40827	BBP02145	5928-26-7	Sissotrin	24-H4	446.404	10(μ)M	DMSO
NCRC40828	BBP02146	142566-61-8	Calanolide E	24-H5	388.454	10(μ)M	DMSO
NCRC40829	BBP02147	350986-74-2	Dendocarbin A	24-H6	250.333	10(μ)M	DMSO
NCRC40830	BBP02148	146450-83-1	Camaric acid	24-H7	568.784	10(μ)M	DMSO
NCRC40831	BBP02149	31427-08-4	Isotachioside	24-H8	302.277	10(μ)M	DMSO
NCRC40832	BBP02150	No	7'-O-Ethylmarmin	24-H9	360.444	10(μ)M	DMSO
NCRC40833	BBP02152	70411-27-7	Dihydrotamarixetin	24-H10	318.278	10(μ)M	DMSO
NCRC40834	BBP02153	130288-60-7	Rubiarbonol B	24-H11	458.716	10(μ)M	DMSO
NCRC40835	BBP00363	No	No	25-A2	384.379	10(μ)M	DMSO
NCRC40836	BBP01390	No	No	25-A3	478.532	10(μ)M	DMSO
NCRC40837	BBP01668	No	Astragalin 4',2",3",4",6"-pentaacetate	25-A4	658.56	10(μ)M	DMSO
NCRC40838	BBP01739	487-52-5	Butein	25-A5	272.253	10(μ)M	DMSO
NCRC40839	BBP01980	93915-36-7	Hirsutanonol 5-O-glucoside	25-A6	508.515	10(μ)M	DMSO
NCRC40840	BBP02049	76996-27-5	Garcinone C	25-A7	414.448	10(μ)M	DMSO
NCRC40841	BBP02056	104778-16-7	4-O-Methylsappanol	25-A8	318.321	10(μ)M	DMSO
NCRC40842	BBP02094	474-07-7	Brazilin	25-A9	286.279	10(μ)M	DMSO
NCRC40843	BBP02144	1223097-20-8	6"-O-acetylisovitexin	25-A10	474.414	10(μ)M	DMSO
NCRC40844	BBP02151	1111897-60-9	7,3',4'-Trihydroxy-3-benzyl-2H-chromene	25-A11	270.28	10(μ)M	DMSO
NCRC40845	BBP02154	144223-70-1	Dehydroadynerigenin glucosyldigitaloside	25-B2	692.79	10(μ)M	DMSO
NCRC40846	BBP02155	41744-39-2	Acuminatin	25-B3	340.413	10(μ)M	DMSO
NCRC40847	BBP02156	603-61-2	Tamarixetin	25-B4	316.262	10(μ)M	DMSO
NCRC40848	BBP02157	79995-67-8	Blumeatin B	25-B5	332.305	10(μ)M	DMSO
NCRC40849	BBP02158	924910-83-8	21-Deoxyneridienone B	25-B6	328.445	10(μ)M	DMSO
NCRC40850	BBP02159	4674-50-4	Nootkatone	25-B7	218.335	10(μ)M	DMSO
NCRC40851	BBP02160	4773-96-0	Mangiferin	25-B8	422.34	10(μ)M	DMSO
NCRC40852	BBP02161	72944-06-0	30-Hydroxylup-20(29)-en-3-one	25-B9	440.701	10(μ)M	DMSO
NCRC40853	BBP02162	34425-25-7	Lyoniside	25-B10	552.568	10(μ)M	DMSO
NCRC40854	BBP02163	34169-69-2	Pterosin Z	25-B11	232.318	10(μ)M	DMSO
NCRC40855	BBP02164	94344-54-4	Sappanchalcone	25-C2	286.279	10(μ)M	DMSO
NCRC40856	BBP02165	1025023-04-4	Periglaucine A	25-C3	373.4	10(μ)M	DMSO
NCRC40857	BBP02166	80453-44-7	Padmatin	25-C4	318.278	10(μ)M	DMSO
NCRC40858	BBP02167	945259-61-0	4-Hydroxy-2-methoxyphenol 1-O-(6-O-syringoyl)glucoside	25-C5	482.435	10(μ)M	DMSO
NCRC40859	BBP02168	57296-22-7	Boehmenan	25-C6	712.738	10(μ)M	DMSO
NCRC40860	BBP02169	61262-81-5	Cannabispiran	25-C7	246.302	10(μ)M	DMSO

NCRC40861	BBP02170	482-68-8	Sarpagine	25-C8	310.39	10(μ)M	DMSO
NCRC40862	BBP02173	176519-75-8	8-Hydroxyodoroside A	25-C9	534.681	10(μ)M	DMSO
NCRC40863	BBP02174	118024-26-3	Blumeatin	25-C10	302.279	10(μ)M	DMSO
NCRC40864	BBP02175	529-40-8	Ombuin	25-C11	330.289	10(μ)M	DMSO
NCRC40865	BBP02176	486-60-2	Bergaptol	25-D2	202.163	10(μ)M	DMSO
NCRC40866	BBP02177	84104-80-3	Wilforlide A acetate	25-D3	496.721	10(μ)M	DMSO
NCRC40867	BBP02178	99-18-3	Prunasin	25-D4	295.288	10(μ)M	DMSO
NCRC40868	BBP02181	No	6'-O-Acetylpaniculoside II	25-D5	538.627	10(μ)M	DMSO
NCRC40869	BBP02182	14957-38-1	Marmin	25-D6	332.391	10(μ)M	DMSO
NCRC40870	BBP02183	70677-47-3	Canniprene	25-D7	342.429	10(μ)M	DMSO
NCRC40871	BBP02184	64125-60-6	Longistylin C	25-D8	294.387	10(μ)M	DMSO
NCRC40872	BBP02185	1049674-06-7	8-Hydroxydigitoxigenin	25-D9	390.513	10(μ)M	DMSO
NCRC40873	BBP02186	376361-96-5	7,4'-Dihydroxy-3'-prenylflavan	25-D10	310.387	10(μ)M	DMSO
NCRC40874	BBP02187	99624-27-8	Kazinol B	25-D11	392.487	10(μ)M	DMSO
NCRC40875	BBP02188	32884-36-9	Cajanin	25-E2	300.263	10(μ)M	DMSO
NCRC40876	BBP02189	38242-02-3	β-Amyrenonol	25-E3	440.701	10(μ)M	DMSO
NCRC40877	BBP02190	62043-53-2	Onitisin 2'-O-glucoside	25-E4	426.457	10(μ)M	DMSO
NCRC40878	BBP02191	87686-86-0	6-Hydroxyrubiadin	25-E5	270.237	10(μ)M	DMSO
NCRC40879	BBP02192	521-32-4	Bilobetin	25-E6	552.485	10(μ)M	DMSO
NCRC40880	BBP02193	7622-53-9	5-Deoxycajanin	25-E7	284.263	10(μ)M	DMSO
NCRC40881	BBP02197	102036-29-3	Protosappanin B	25-E8	304.295	10(μ)M	DMSO
NCRC40882	BBP02198	41060-16-6	Skullcapflavone I	25-E9	314.289	10(μ)M	DMSO
NCRC40883	BBP02199	90902-21-9	Broussonin E	25-E10	288.338	10(μ)M	DMSO
NCRC40884	BBP02200	33429-83-3	Quercetin 3,4'-dimethyl ether	25-E11	330.289	10(μ)M	DMSO
NCRC40885	BBP02201	264234-05-1	6',7'-Dihydroxybergamottin	25-F2	372.412	10(μ)M	DMSO
NCRC40886	BBP02202	644967-44-2	Rubianthraquinone	25-F3	284.263	10(μ)M	DMSO
NCRC40887	BBP02203	91269-84-0	Melilotigenin B	25-F4	454.684	10(μ)M	DMSO
NCRC40888	BBP02204	188970-21-0	Melilotigenin C	25-F5	456.7	10(μ)M	DMSO
NCRC40889	BBP02205	10267-31-9	Labd-13-ene-8,15-diol	25-F6	308.499	10(μ)M	DMSO
NCRC40890	BBP02206	522-47-4	Lochnerine	25-F7	324.417	10(μ)M	DMSO
NCRC40891	BBP02207	76947-60-9	Onitin 2'-O-glucoside	25-F8	410.458	10(μ)M	DMSO
NCRC40892	BBP02208	529-55-5	Prunin	25-F9	434.393	10(μ)M	DMSO
NCRC40893	BBP02209	376362-03-7	2'-O-Methylbroussonin C	25-F10	326.429	10(μ)M	DMSO
NCRC40894	BBP02210	82427-77-8	Maglifloenone	25-F11	386.438	10(μ)M	DMSO
NCRC40895	BBP02211	68160-76-9	Nortetraphyllicine	25-G2	294.391	10(μ)M	DMSO
NCRC40896	BBP02213	65597-44-6	Cerberic acid	25-G3	220.178	10(μ)M	DMSO
NCRC40897	BBP02215	No	β-Amyrenonol methylthiomethyl ether	25-G4	500.819	10(μ)M	DMSO
NCRC40898	BBP02216	2935-32-2	Olean-12-ene-3,11-dione	25-G5	438.685	10(μ)M	DMSO
NCRC40899	BBP02217	No	Methyl mandelate glucoside	25-G6	328.315	10(μ)M	DMSO
NCRC40900	BBP02218	767-98-6	Mallorepine	25-G7	134.135	10(μ)M	DMSO
NCRC40901	BBP02219	133360-51-7	Daturataturin A	25-G8	616.739	10(μ)M	DMSO
NCRC40902	BBP02220	10178-31-1	Elliotinol	25-G9	288.467	10(μ)M	DMSO
NCRC40903	BBP02221	1244-58-2	Cannabidiolic acid	25-G10	358.471	10(μ)M	DMSO
NCRC40904	BBP02222	1227375-09-8	Bi-linderone	25-G11	600.612	10(μ)M	DMSO
NCRC40905	BBP02224	320624-68-8	Marmin acetonide	25-H2	372.455	10(μ)M	DMSO
NCRC40906	BBP02225	684217-08-1	6',7'-Dihydroxybergamottin acetonide	25-H3	412.476	10(μ)M	DMSO
NCRC40907	BBP02229	1221-43-8	Auraptenol	25-H4	260.285	10(μ)M	DMSO
NCRC40908	BBP02237	15527-80-7	Peraksine	25-H5	310.39	10(μ)M	DMSO
NCRC40909	BBP02238	51-55-8	Atropine	25-H6	289.369	10(μ)M	DMSO
NCRC40910	BBP02240	213912-46-0	Barbacarpan	25-H7	322.355	10(μ)M	DMSO
NCRC40911	BBP02241	126060-09-1	6-O-Methylcerevisterol	25-H8	444.69	10(μ)M	DMSO
NCRC40912	BBP02242	904667-65-8	Daturametelin I	25-H9	616.739	10(μ)M	DMSO

NCRC40913	BBP02244	138772-01-7	Eugenol rutinoside	25-H10	472.483	10(μ)M	DMSO
NCRC40914	BBP02247	36545-53-6	Cycloheterophyllin	25-H11	502.555	10(μ)M	DMSO
NCRC40915	BBP02248	5356-56-9	β-Amyrenonol acetate	26-A2	482.738	10(μ)M	DMSO
NCRC40916	BBP02249	163136-19-4	Cannabisin F	26-A3	624.68	10(μ)M	DMSO
NCRC40917	BBP02250	61217-80-9	Uzarigenin digitaloside	26-A4	534.681	10(μ)M	DMSO
NCRC40918	BBP02251	64052-90-0	Cannabispirol	26-A5	248.318	10(μ)M	DMSO
NCRC40919	BBP02253	358721-33-2	Apocynol A	26-A6	224.296	10(μ)M	DMSO
NCRC40920	BBP02254	1016974-78-9	Olivil monoacetate	26-A7	418.437	10(μ)M	DMSO
NCRC40921	BBP02255	256445-66-6	Schleicheol 1	26-A8	444.733	10(μ)M	DMSO
NCRC40922	BBP02256	150150-61-1	11,13-Dihydroivalin	26-A9	250.333	10(μ)M	DMSO
NCRC40923	BBP02257	2400-71-7	Pyrocatechol monoglucoside	26-A10	272.251	10(μ)M	DMSO
NCRC40924	BBP02258	20733-94-2	Methyl sinapate	26-A11	238.237	10(μ)M	DMSO
NCRC40925	BBP02259	61117-89-3	Epipterosin L 2'-O-glucoside	26-B2	426.457	10(μ)M	DMSO
NCRC40926	BBP02260	53505-68-3	4,9,9'-Trihydroxy-3,3'-dimethoxy-8,4'-oxyneolignan	26-B3	362.417	10(μ)M	DMSO
NCRC40927	BBP02261	529-59-9	Genistin	26-B4	432.378	10(μ)M	DMSO
NCRC40928	BBP02262	80510-06-1	Grossamide	26-B5	624.68	10(μ)M	DMSO
NCRC40929	BBP02263	33069-62-4	Paclitaxel	26-B6	853.906	10(μ)M	DMSO
NCRC40930	BBP02264	31282-07-2	Raucaffricine	26-B7	512.552	10(μ)M	DMSO
NCRC40931	BBP02265	1265908-20-0	8,14-Epoxyergosta-4,22-diene-3,6-dione	26-B8	424.615	10(μ)M	DMSO
NCRC40932	BBP02266	95456-43-2	Hydroxytuberosone	26-B9	354.353	10(μ)M	DMSO
NCRC40933	BBP02268	66648-44-0	N-Feruloyloctopamine	26-B10	329.347	10(μ)M	DMSO
NCRC40934	BBP02269	84299-80-9	Pterosin D 3-O-glucoside	26-B11	410.458	10(μ)M	DMSO
NCRC40935	BBP02270	858360-61-9	Marsdenoside F	26-C2	752.885	10(μ)M	DMSO
NCRC40936	BBP02271	467-81-2	Rehmannic acid	26-C3	552.784	10(μ)M	DMSO
NCRC40937	BBP02272	160242-09-1	14-Deoxy-11-hydroxyandrographolide	26-C4	350.449	10(μ)M	DMSO
NCRC40938	BBP02273	857297-90-6	3-Hydroxysarpagine	26-C5	326.39	10(μ)M	DMSO
NCRC40939	BBP02275	10236-47-2	Naringin	26-C6	580.535	10(μ)M	DMSO
NCRC40940	BBP02276	869807-57-8	Andropanolide	26-C7	350.449	10(μ)M	DMSO
NCRC40941	BBP02277	66648-45-1	N-p-Coumaroyloctopamine	26-C8	299.321	10(μ)M	DMSO
NCRC40942	BBP02279	125305-73-9	21,24-Epoxycycloartane-3,25-diol	26-C9	458.716	10(μ)M	DMSO
NCRC40943	BBP02280	115040-04-5	29-Norcycloart-23-ene-3,25-diol	26-C10	428.69	10(μ)M	DMSO
NCRC40944	BBP02281	69636-83-5	α-Cannabispiranol	26-C11	248.318	10(μ)M	DMSO
NCRC40945	BBP02282	27215-14-1	Neoandrographolide	26-D2	480.591	10(μ)M	DMSO
NCRC40946	BBP02284	76166-59-1	Derrone	26-D3	336.338	10(μ)M	DMSO
NCRC40947	BBP02285	77988-07-9	Secologanin dimethyl acetal	26-D4	434.435	10(μ)M	DMSO
NCRC40948	BBP02286	166547-20-2	Eurycarpin A	26-D5	338.354	10(μ)M	DMSO
NCRC40949	BBP02287	No	24-Hydroxy-25-ethoxy-3,4-seco-cycloart-4(28)-en-3-oic acid methyl ester	26-D6	516.795	10(μ)M	DMSO
NCRC40950	BBP02289	73731-87-0	Broussonin A	26-D7	258.312	10(μ)M	DMSO
NCRC40951	BBP02290	73731-86-9	Broussonin B	26-D8	258.312	10(μ)M	DMSO
NCRC40952	BBP02291	191729-44-9	Tenacissoside I	26-D9	814.955	10(μ)M	DMSO
NCRC40953	BBP02293	79114-77-5	Lariciresinol acetate	26-D10	402.438	10(μ)M	DMSO
NCRC40954	BBP02294	952485-00-6	Hydrangenoside A dimethyl acetal	26-D11	666.71	10(μ)M	DMSO
NCRC40955	BBP02295	29307-03-7	Deoxyelephantopin	26-E2	344.358	10(μ)M	DMSO
NCRC40956	BBP02297	135384-00-8	8-Prenyldaidzein	26-E3	322.355	10(μ)M	DMSO
NCRC40957	BBP02298	185213-52-9	Scabertopin	26-E4	358.385	10(μ)M	DMSO
NCRC40958	BBP02299	605-14-1	Serpentinic acid	26-E5	335.376	10(μ)M	DMSO
NCRC40959	BBP02302	7154-01-0	Safrolglycol	26-E6	196.2	10(μ)M	DMSO
NCRC40960	BBP02303	857897-01-9	11,12-Di-O-acetyltenacigenin B	26-E7	448.549	10(μ)M	DMSO
NCRC40961	BBP02304	869384-82-7	14-Deoxy-17-hydroxyandrographolide	26-E8	352.465	10(μ)M	DMSO

NCRC40962	BBP02305	219721-33-2	14-Deoxy-12-hydroxyandrographolide	26-E9	350.449	10(μ)M	DMSO
NCRC40963	BBP02306	No	25-Ethoxy-24-oxo-3,4-secocycloart -4(28)-en-3-oic acid methyl ester	26-E10	514.779	10(μ)M	DMSO
NCRC40964	BBP02307	101691-27-4	Barpisoflavone A	26-E11	300.263	10(μ)M	DMSO
NCRC40965	BBP02308	88-14-2	Furan-2-carboxylic acid	26-F2	112.083	10(μ)M	DMSO
NCRC40966	BBP02309	143212-60-6	Dehydroadynerigenin β-neritrioside	26-F3	838.931	10(μ)M	DMSO
NCRC40967	BBP02310	30452-60-9	Cyclomusalenone	26-F4	424.702	10(μ)M	DMSO
NCRC40968	BBP02311	160498-00-0	Bisandrographolide A	26-F5	664.868	10(μ)M	DMSO
NCRC40969	BBP02312	97411-50-2	2',3'-Dehydrosalannol	26-F6	554.671	10(μ)M	DMSO
NCRC40970	BBP02313	94805-83-1	Isolicoflavonol	26-F7	354.353	10(μ)M	DMSO
NCRC40971	BBP02314	205534-17-4	2",4"-Di-O-(Z-p-coumaroyl)afzelin	26-F8	724.663	10(μ)M	DMSO
NCRC40972	BBP02315	1238116-48-7	Kazinol U	26-F9	326.386	10(μ)M	DMSO
NCRC40973	BBP02316	1110-56-1	Deacetylsalannin	26-F10	554.671	10(μ)M	DMSO
NCRC40974	BBP02317	5945-86-8	Nimbin	26-F11	540.601	10(μ)M	DMSO
NCRC40975	BBP02318	22338-67-6	Grandiflorenic acid	26-G2	300.435	10(μ)M	DMSO
NCRC40976	BBP02319	52275-04-4	3,5-Diprenyl-4-hydroxybenzaldehyde	26-G3	258.355	10(μ)M	DMSO
NCRC40977	BBP02320	349534-70-9	Methyl dodonate A	26-G4	344.445	10(μ)M	DMSO
NCRC40978	BBP02321	7678-85-5	2'-Hydroxydaidzein	26-G5	270.237	10(μ)M	DMSO
NCRC40979	BBP02322	67023-80-7	Ohchinin	26-G6	602.714	10(μ)M	DMSO
NCRC40980	BBP02323	20460-33-7	Epitaraxerol	26-G7	426.717	10(μ)M	DMSO
NCRC40981	BBP02324	263368-91-8	4-Acetyl-3,6,8-trihydroxy-3-methyldihydronaphthalenone	26-G8	250.247	10(μ)M	DMSO
NCRC40982	BBP02325	511-15-9	Totarol	26-G9	286.452	10(μ)M	DMSO
NCRC40983	BBP02326	19941-83-4	Kolavenol	26-G10	290.483	10(μ)M	DMSO
NCRC40984	BBP02327	105608-27-3	Prostephanaberrine	26-G11	343.374	10(μ)M	DMSO
NCRC40985	BBP02328	53526-67-3	Durantoside I	26-H2	552.524	10(μ)M	DMSO
NCRC40986	BBP02329	53526-66-2	Durantoside II	26-H3	582.55	10(μ)M	DMSO
NCRC40987	BBP02330	27856-54-8	Lamiide	26-H4	422.381	10(μ)M	DMSO
NCRC40988	BBP02331	31721-94-5	5,7-Dihydroxychromone	26-H5	178.142	10(μ)M	DMSO
NCRC40989	BBP02332	117614-84-3	1-Hydroxy-1-(4-hydroxy-2-methoxyphenyl)-3-(4-hydroxyphenyl)propan-2-one	26-H6	288.295	10(μ)M	DMSO
NCRC40990	BBP02334	191729-43-8	Tenacissoside G	26-H7	792.949	10(μ)M	DMSO
NCRC40991	BBP02335	1268140-15-3	5,7,3'-Trihydroxy-4'-methoxy-8-prenylflavanone	26-H8	370.396	10(μ)M	DMSO
NCRC40992	BBP02336	136196-47-9	3,4'-Dihydroxy-3',5'-dimethoxypropiophenone	26-H9	226.226	10(μ)M	DMSO
NCRC40993	BBP02337	162290-05-3	7,3'-Dihydroxy-4'-methoxyflavan	26-H10	272.296	10(μ)M	DMSO
NCRC40994	BBP01998	110187-11-6	1,5,8-Trihydroxy-3-methoxy-2-prenylxanthone	26-H11	342.343	10(μ)M	DMSO
NCRC40995	BBP02338	61448-03-1	Lup-20(29)-ene-2α,3β-diol	27-A2	442.717	10(μ)M	DMSO
NCRC40996	BBP02339	502-69-2	Phytone	27-A3	268.478	10(μ)M	DMSO
NCRC40997	BBP02341	70387-38-1	6α-Hydroxynidorellol	27-A4	322.482	10(μ)M	DMSO
NCRC40998	BBP02343	263764-83-6	Buergerinin G	27-A5	184.189	10(μ)M	DMSO
NCRC40999	BBP02345	13895-92-6	Rutaretin	27-A6	262.258	10(μ)M	DMSO
NCRC41000	BBP02346	88721-09-9	Adynerigenin β-neritrioside	27-A7	840.947	10(μ)M	DMSO
NCRC41001	BBP02347	349487-98-5	Methyl dodonate A acetate	27-A8	386.481	10(μ)M	DMSO
NCRC41002	BBP02352	118555-84-3	Floribundone 1	27-A9	566.511	10(μ)M	DMSO
NCRC41003	BBP02353	77-60-1	Tigogenin	27-A10	416.636	10(μ)M	DMSO
NCRC41004	BBP02354	919769-83-8	Buergerinin B	27-A11	202.204	10(μ)M	DMSO

NCRC41005	BBP02355	117254-98-5	8α-Hydroxylabda-13(16),14-dien-19-yl p-hydroxycinnamate	27-B2	452.625	10(μ)M	DMSO
NCRC41006	BBP02356	No	6α,7β-Isopropylidenedioxy-abienol	27-B3	362.546	10(μ)M	DMSO
NCRC41007	BBP02357	141973-41-3	14-Deoxy-11,12-didehydroandrographiside	27-B4	494.575	10(μ)M	DMSO
NCRC41008	BBP02358	61854-36-2	Demethoxycapillarisin	27-B5	286.236	10(μ)M	DMSO
NCRC41009	BBP02359	99624-92-7	Uncinatone	27-B6	326.386	10(μ)M	DMSO
NCRC41010	BBP02360	78916-55-9	Deacetylnimbinene	27-B7	440.529	10(μ)M	DMSO
NCRC41011	BBP02361	72058-36-7	Lochnericine	27-B8	352.427	10(μ)M	DMSO
NCRC41012	BBP02362	18609-16-0	Deacetylnimbin	27-B9	498.565	10(μ)M	DMSO
NCRC41013	BBP02363	263368-92-9	4-(cis)-Acetyl-3,6,8-trihydroxy-3-methyldihydronaphthalenone	27-B10	250.247	10(μ)M	DMSO
NCRC41014	BBP02364	161753-49-7	12-Oxocalanolide A	27-B11	368.423	10(μ)M	DMSO
NCRC41015	BBP02365	126-17-0	Solasodine	27-C2	413.636	10(μ)M	DMSO
NCRC41016	BBP02367	76248-63-0	Piptocarphin A	27-C3	422.426	10(μ)M	DMSO
NCRC41017	BBP02368	126005-94-5	28-Deoxonimbolide	27-C4	452.539	10(μ)M	DMSO
NCRC41018	BBP02369	82209-72-1	Andropanoside	27-C5	496.59	10(μ)M	DMSO
NCRC41019	BBP02370	77658-38-9	Pterokaurene L3	27-C6	318.45	10(μ)M	DMSO
NCRC41020	BBP02373	103476-99-9	Diacetylpiptocarphol	27-C7	396.388	10(μ)M	DMSO
NCRC41021	BBP02374	152253-67-3	Mupinensisone	27-C8	440.701	10(μ)M	DMSO
NCRC41022	BBP02376	776-86-3	Isoscopoletin	27-C9	192.168	10(μ)M	DMSO
NCRC41023	BBP02377	165689-32-7	Torososide A	27-C10	728.652	10(μ)M	DMSO
NCRC41024	BBP02378	20065-99-0	Hennadiol	27-C11	442.717	10(μ)M	DMSO
NCRC41025	BBP02379	78516-69-5	Dulcioic acid	27-D2	456.7	10(μ)M	DMSO
NCRC41026	BBP02380	2679-65-4	Robtein	27-D3	288.252	10(μ)M	DMSO
NCRC41027	BBP02381	606-91-7	Homopterocarpin	27-D4	284.307	10(μ)M	DMSO
NCRC41028	BBP02382	130395-82-3	2-Oxokolavenol	27-D5	304.467	10(μ)M	DMSO
NCRC41029	BBP02384	2524-37-0	Ethyl orsellinate	27-D6	196.2	10(μ)M	DMSO
NCRC41030	BBP02385	15648-86-9	Myricetin 3-O-galactoside	27-D7	480.376	10(μ)M	DMSO
NCRC41031	BBP02386	381691-22-1	28-Hydroxy-3-oxoolean-12-en-29-oic acid	27-D8	470.684	10(μ)M	DMSO
NCRC41032	BBP02388	79406-13-6	3α-Cinnamoyloxypterokaurene L3	27-D9	464.593	10(μ)M	DMSO
NCRC41033	BBP02390	76045-49-3	Broussonin C	27-D10	312.403	10(μ)M	DMSO
NCRC41034	BBP02391	1222475-77-5	3-Epimeliasenin B	27-D11	468.668	10(μ)M	DMSO
NCRC41035	BBP02392	26194-57-0	Isotaxiresinol	27-E2	346.374	10(μ)M	DMSO
NCRC41036	BBP02393	252333-72-5	Isotaxiresinol 9,9'-acetonide	27-E3	386.438	10(μ)M	DMSO
NCRC41037	BBP02394	868409-19-2	(+)-Puerol B 2''-O-glucoside	27-E4	474.457	10(μ)M	DMSO
NCRC41038	BBP02395	2150-11-0	7,3',4'-Trihydroxyflavone	27-E5	270.237	10(μ)M	DMSO
NCRC41039	BBP02396	439923-16-7	Isoscabertopin	27-E6	358.385	10(μ)M	DMSO
NCRC41040	BBP02397	500-66-3	Olivetol	27-E7	180.244	10(μ)M	DMSO
NCRC41041	BBP02398	40951-69-7	Taxiresinol	27-E8	346.374	10(μ)M	DMSO
NCRC41042	BBP02400	24224-30-4	3-Methyl-9H-carbazol-2-ol	27-E9	197.233	10(μ)M	DMSO
NCRC41043	BBP02402	79406-10-3	ent-3β-Cinnamoyloxykaur-16-en-19-oic acid	27-E10	448.594	10(μ)M	DMSO
NCRC41044	BBP02403	78012-28-9	1,3-Diacetylvilasinin	27-E11	512.634	10(μ)M	DMSO
NCRC41045	BBP02404	480-11-5	Oroxylin A	27-F2	284.263	10(μ)M	DMSO
NCRC41046	BBP02405	471271-55-3	(E)-Aldosecologanin	27-F3	758.718	10(μ)M	DMSO
NCRC41047	BBP02406	3681-99-0	Puerarin	27-F4	416.378	10(μ)M	DMSO
NCRC41048	BBP02408	187110-72-1	Clauszoline M	27-F5	227.215	10(μ)M	DMSO
NCRC41049	BBP02409	1000676-45-8	Stilbostemin N	27-F6	258.312	10(μ)M	DMSO
NCRC41050	BBP02410	139101-67-0	Tristin	27-F7	260.285	10(μ)M	DMSO
NCRC41051	BBP02411	162411-67-8	Stilbostemin B	27-F8	228.286	10(μ)M	DMSO
NCRC41052	BBP02413	121064-78-6	3-O-trans-p-Coumaroyltormentic acid	27-F9	634.842	10(μ)M	DMSO

NCRC41053	BBP02414	123497-84-7	Demethylmurrayanine	27-F10	211.216	10(μ)M	DMSO
NCRC41054	BBP02415	142846-95-5	Clausine D	27-F11	279.333	10(μ)M	DMSO
NCRC41055	BBP02416	114542-44-8	Honyucitrin	27-G2	406.471	10(μ)M	DMSO
NCRC41056	BBP02420	22338-69-8	Grandifloric acid	27-G3	318.45	10(μ)M	DMSO
NCRC41057	BBP02421	35030-38-7	ent-17-Hydroxykaur-15-en-19-oic acid	27-G4	318.45	10(μ)M	DMSO
NCRC41058	BBP02422	1309362-77-3	Cerberic acid B	27-G5	210.183	10(μ)M	DMSO
NCRC41059	BBP02423	20230-41-5	Dregeoside Aa1	27-G6	939.133	10(μ)M	DMSO
NCRC41060	BBP02424	6730-83-2	Kaurenoic acid	27-G7	302.451	10(μ)M	DMSO
NCRC41061	BBP02425	113963-39-6	Andrographidine C	27-G8	460.431	10(μ)M	DMSO
NCRC41062	BBP02426	113963-41-0	Andrographidine E	27-G9	490.457	10(μ)M	DMSO
NCRC41063	BBP02427	942626-75-7	5-Hydroxy-7,8,2',5'-tetramethoxyflavone 5-O-	27-G10	520.483	10(μ)M	DMSO
NCRC41064	BBP02429	492-14-8	Butin	27-G11	272.253	10(μ)M	DMSO
NCRC41065	BBP02430	552-58-9	Eriodictyol	27-H2	288.252	10(μ)M	DMSO
NCRC41066	BBP02431	182261-94-5	Clausine I	27-H3	241.242	10(μ)M	DMSO
NCRC41067	BBP02432	139115-59-6	Isomurralonginol acetate	27-H4	302.322	10(μ)M	DMSO
NCRC41068	BBP02433	160498-02-2	Bisandrographolide C	27-H5	664.868	10(μ)M	DMSO
NCRC41069	BBP02434	642-18-2	Alstonine	27-H6	348.395	10(μ)M	DMSO
NCRC41070	BBP02435	97169-44-3	6-Epi-8-O-acetylharpagide	27-H7	406.382	10(μ)M	DMSO
NCRC41071	BBP02436	86362-16-5	6-Epiharpagide	27-H8	364.345	10(μ)M	DMSO
NCRC41072	BBP02438	18449-41-7	Madecassic acid	27-H9	504.699	10(μ)M	DMSO
NCRC41073	BBP02439	642-17-1	Akuammigine	27-H10	352.427	10(μ)M	DMSO
NCRC41074	BBP02441	4184-34-3	Mangiferolic acid	27-H11	456.7	10(μ)M	DMSO
NCRC41075	BBP02443	802909-72-4	Dehydro-δ-tocopherol	28-A2	400.637	10(μ)M	DMSO
NCRC41076	BBP02444	38927-54-7	Isodeoxyelephantopin	28-A3	344.358	10(μ)M	DMSO
NCRC41077	BBP02446	7560-49-8	Methyl 3,4,5-	28-A4	252.263	10(μ)M	DMSO
NCRC41078	BBP02447	82464-35-5	3-Oxotirucalla-7,24-dien-21-oic acid	28-A5	454.684	10(μ)M	DMSO
NCRC41079	BBP02448	80396-57-2	Protoplumericin A	28-A6	778.707	10(μ)M	DMSO
NCRC41080	BBP02449	116499-73-1	9α,13α-Epidioxyabiet-8(14)-en-18-oic acid	28-A7	334.45	10(μ)M	DMSO
NCRC41081	BBP02450	349534-73-2	Dodonolide	28-A8	312.403	10(μ)M	DMSO
NCRC41082	BBP02451	79406-11-4	3α-Angeloyloxypterokaurene L3	28-A9	416.55	10(μ)M	DMSO
NCRC41083	BBP02452	24694-79-9	Uvedalin	28-A10	448.463	10(μ)M	DMSO
NCRC41084	BBP02453	349112-30-7	Cryptomeridiol 11-rhamnoside	28-A11	386.523	10(μ)M	DMSO
NCRC41085	BBP02455	111035-65-5	Annonacin	28-B2	596.878	10(μ)M	DMSO
NCRC41086	BBP02456	17297-56-2	Biorobin	28-B3	594.518	10(μ)M	DMSO
NCRC41087	BBP02458	34020-07-0	Vinorine	28-B4	334.412	10(μ)M	DMSO
NCRC41088	BBP02459	24694-80-2	Chlorouvedalin	28-B5	484.924	10(μ)M	DMSO
NCRC41089	BBP02463	70-70-2	1-(4-Hydroxyphenyl)propan-1-one	28-B6	150.174	10(μ)M	DMSO
NCRC41090	BBP02465	98665-66-8	Dregeoside Ga1	28-B7	941.149	10(μ)M	DMSO
NCRC41091	BBP02466	66777-70-6	6,8-Diprenylorobol	28-B8	422.47	10(μ)M	DMSO
NCRC41092	BBP02467	1246926-08-8	5,7,4'-Trihydroxy-3,6-dimethoxy-3',5'-diprenylflavone	28-B9	466.523	10(μ)M	DMSO
NCRC41093	BBP02468	10163-83-4	Drevogenin A	28-B10	490.629	10(μ)M	DMSO
NCRC41094	BBP02469	62623-86-3	11S,12-Dihydroxyspirovetiv-1(10)-en-2-one	28-B11	252.349	10(μ)M	DMSO
NCRC41095	BBP02470	41997-41-5	Dihydropashanone	28-C2	302.322	10(μ)M	DMSO
NCRC41096	BBP02471	64121-98-8	Di-O-methylcrenatin	28-C3	346.33	10(μ)M	DMSO
NCRC41097	BBP02472	956384-55-7	Demethylsonchifolin	28-C4	360.401	10(μ)M	DMSO
NCRC41098	BBP02476	74690-89-4	Vibsanin C	28-C5	416.55	10(μ)M	DMSO
NCRC41099	BBP02477	133005-15-9	13-Epijhanol	28-C6	306.483	10(μ)M	DMSO
NCRC41100	BBP02478	221466-41-7	2-Oxokolavelool	28-C7	304.467	10(μ)M	DMSO
NCRC41101	BBP02479	5132-66-1	27-Hydroxymangiferonic acid	28-C8	470.684	10(μ)M	DMSO

NCRC41102	BBP02481	644-06-4	Precocene II	28-C9	220.264	10(μ)M	DMSO
NCRC41103	BBP02482	74474-76-3	Casegravol	28-C10	276.285	10(μ)M	DMSO
NCRC41104	BBP02483	41682-30-8	8-Acetoxypentadeca-1,9Z-diene-4,6-diyn-3-ol	28-C11	274.355	10(μ)M	DMSO
NCRC41105	BBP02485	17983-82-3	27-Hydroxymangiferolic acid	28-D2	472.7	10(μ)M	DMSO
NCRC41106	BBP02488	6880-91-7	Dihydrochelerythrine	28-D3	349.38	10(μ)M	DMSO
NCRC41107	BBP02489	232266-08-9	23-Hydroxymangiferonic acid	28-D4	470.684	10(μ)M	DMSO
NCRC41108	BBP02491	1318158-89-2	Cardenolide B-1	28-D5	532.666	10(μ)M	DMSO
NCRC41109	BBP02492	35109-93-4	Adynerin	28-D6	516.666	10(μ)M	DMSO
NCRC41110	BBP02493	52628-62-3	Dehydroadynerigenin digitaloside	28-D7	530.65	10(μ)M	DMSO
NCRC41111	BBP02494	864719-19-7	Sequosempervirin D	28-D8	356.412	10(μ)M	DMSO
NCRC41112	BBP02495	62596-34-3	Cyclomorusin	28-D9	418.439	10(μ)M	DMSO
NCRC41113	BBP02496	221466-42-8	2β-Hydroxykolavelool	28-D10	306.483	10(μ)M	DMSO
NCRC41114	BBP02497	62574-30-5	11R,12-Dihydroxyspirovetiv-1(10)-en-2-one	28-D11	252.349	10(μ)M	DMSO
NCRC41115	BBP02500	710952-13-9	5,8,4'-Trihydroxy-7-methoxyflavone 8-O-glucoside	28-E2	462.403	10(μ)M	DMSO
NCRC41116	BBP02501	5835-26-7	Isopimaric acid	28-E3	302.451	10(μ)M	DMSO
NCRC41117	BBP02502	220328-03-0	3,11,12-Trihydroxyspirovetiv-1(10)-en-2-one	28-E4	268.349	10(μ)M	DMSO
NCRC41118	BBP02503	220328-04-1	3,11,12-Trihydroxyspirovetiv-1(10)-en-2-one	28-E5	268.349	10(μ)M	DMSO
NCRC41119	BBP02504	219298-74-5	2-Hydroxy-3,4,5,6-tetramethoxychalcone	28-E6	344.358	10(μ)M	DMSO
NCRC41120	BBP02507	4431-42-9	Isopedicin	28-E7	330.332	10(μ)M	DMSO
NCRC41121	BBP02510	491-80-5	Biochanin A	28-E8	284.263	10(μ)M	DMSO
NCRC41122	BBP02512	122-69-0	Cinnamyl cinnamate	28-E9	264.318	10(μ)M	DMSO
NCRC41123	BBP02513	17303-67-2	Goniothalamin	28-E10	200.233	10(μ)M	DMSO
NCRC41124	BBP02516	87402-88-8	Denudatin B	28-E11	356.412	10(μ)M	DMSO
NCRC41125	BBP02517	194613-74-6	21αH-24-Norhopa-4(23),22(29)-diene-3β,6β-diol	28-F2	426.674	10(μ)M	DMSO
NCRC41126	BBP02518	No	5-(1-Hydroxyethyl)-2,4-dimethoxy tetrahydrofuran-3-ol	28-F3	192.21	10(μ)M	DMSO
NCRC41127	BBP02519	No	17-Epidregeoside Aa1	28-F4	939.133	10(μ)M	DMSO
NCRC41128	BBP02520	82209-76-5	Andrographoside	28-F5	512.59	10(μ)M	DMSO
NCRC41129	BBP02521	18194-29-1	Sequirin C	28-F6	302.322	10(μ)M	DMSO
NCRC41130	BBP02522	7288-11-1	Agatharesinol	28-F7	286.322	10(μ)M	DMSO
NCRC41131	BBP02524	800389-33-7	Agatharesinol acetonide	28-F8	326.386	10(μ)M	DMSO
NCRC41132	BBP02525	163634-05-7	Evofolin C	28-F9	218.292	10(μ)M	DMSO
NCRC41133	BBP02526	38230-99-8	Chloroenhydrin	28-F10	500.923	10(μ)M	DMSO
NCRC41134	BBP02527	36062-05-2	Hexahydrocurcumin	28-F11	374.428	10(μ)M	DMSO
NCRC41135	BBP02528	138870-96-9	5''-Methoxyhexahydrocurcumin	28-G2	404.454	10(μ)M	DMSO
NCRC41136	BBP02529	36062-04-1	Tetrahydrocurcumin	28-G3	372.412	10(μ)M	DMSO
NCRC41137	BBP02532	120-05-8	Sulfuretin	28-G4	270.237	10(μ)M	DMSO
NCRC41138	BBP02533	240122-32-1	Griffithinam	28-G5	295.289	10(μ)M	DMSO
NCRC41139	BBP02534	96405-62-8	Goniotriol	28-G6	250.247	10(μ)M	DMSO
NCRC41140	BBP02535	81122-95-4	(Z)-Lachnophyllum lactone	28-G7	162.185	10(μ)M	DMSO
NCRC41141	BBP02536	16830-15-2	Asiaticoside	28-G8	959.122	10(μ)M	DMSO
NCRC41142	BBP02539	79406-09-0	ent-3β-Tigloyloxykaur-16-en-19-oic acid	28-G9	400.551	10(μ)M	DMSO
NCRC41143	BBP02540	74635-61-3	ent-3β-Angeloyloxykaur-16-en-19-oic acid	28-G10	400.551	10(μ)M	DMSO
NCRC41144	BBP02541	129350-09-0	O-Geranylconiferyl alcohol	28-G11	316.435	10(μ)M	DMSO
NCRC41145	BBP02542	75513-81-4	Cedrin	28-H2	334.278	10(μ)M	DMSO
NCRC41146	BBP02544	77836-86-3	Nelumol A	28-H3	346.461	10(μ)M	DMSO

NCRC41147	BBP02545	41442-57-3	5-Heptadecylresorcinol	28-H4	348.563	10(μ)M	DMSO
NCRC41148	BBP02546	106894-43-3	5-Allyl-3-methoxy-6-methyl-7-(3,4,5-tri methoxyphenyl)bicyclo[3.2.1]oct-3-ene-2,8-dione	28-H5	386.438	10(μ)M	DMSO
NCRC41149	BBP02547	111843-10-8	Hancinone C	28-H6	400.465	10(μ)M	DMSO
NCRC41150	BBP02548	95839-45-5	Hedyotisol B	28-H7	810.837	10(μ)M	DMSO
NCRC41151	BBP02549	17020-27-8	24-Methylenecycloartane-3β,26-diol	28-H8	456.743	10(μ)M	DMSO
NCRC41152	BBP02550	174423-30-4	Sanggenol A	28-H9	424.486	10(μ)M	DMSO
NCRC41153	BBP02551	57726-26-8	4-(Ethoxymethyl)phenol	28-H10	152.19	10(μ)M	DMSO
NCRC41154	BBP02553	114027-39-3	Humantenidine	28-H11	342.389	10(μ)M	DMSO
NCRC41155	BBP02440	32811-40-8	Coniferyl alcohol	29-A2	180.2	10(μ)M	DMSO
NCRC41156	BBP02480	133882-79-8	Eicosyl ferulate	29-A3	474.716	10(μ)M	DMSO
NCRC41157	BBP02508	No	17-Epidrevogenin A	29-A4	490.629	10(μ)M	DMSO
NCRC41158	BBP02511	18411-75-1	Hautriwaic acid	29-A5	332.434	10(μ)M	DMSO
NCRC41159	BBP02530	569-92-6	Rhamnocitrin	29-A6	300.263	10(μ)M	DMSO
NCRC41160	BBP02554	178764-92-6	Agrocybenine	29-A7	206.284	10(μ)M	DMSO
NCRC41161	BBP02555	104021-39-8	8α-Methacryloyloxybalchanin	29-A8	332.391	10(μ)M	DMSO
NCRC41162	BBP02556	129145-51-3	Gancaonin M	29-A9	352.38	10(μ)M	DMSO
NCRC41163	BBP02559	6874-98-2	Vellosimine	29-A10	292.375	10(μ)M	DMSO
NCRC41164	BBP02560	56407-87-5	Tetrahydrolachnophyllum lactone	29-A11	166.217	10(μ)M	DMSO
NCRC41165	BBP02561	5273-85-8	Isoelemicin	29-B2	208.254	10(μ)M	DMSO
NCRC41166	BBP02562	83-95-4	Skimmianine	29-B3	259.257	10(μ)M	DMSO
NCRC41167	BBP02563	1195760-68-9	11-Hydroxygelsenicine	29-B4	342.389	10(μ)M	DMSO
NCRC41168	BBP02564	2671-32-1	Picraline	29-B5	410.463	10(μ)M	DMSO
NCRC41169	BBP02565	4030-51-7	Cassyfiline	29-B6	341.358	10(μ)M	DMSO
NCRC41170	BBP02566	98665-65-7	Dregeoside Da1	29-B7	814.996	10(μ)M	DMSO
NCRC41171	BBP02567	482-01-9	Homoferreirin	29-B8	316.305	10(μ)M	DMSO
NCRC41172	BBP02568	1334309-44-2	3'-Geranyl-3-prenyl-2',4',5,7-tetrahydroxyflavone	29-B9	490.587	10(μ)M	DMSO
NCRC41173	BBP02569	1351931-30-0	Sanggenol P	29-B10	492.603	10(μ)M	DMSO
NCRC41174	BBP02570	121747-90-8	Isochandalone	29-B11	404.455	10(μ)M	DMSO
NCRC41175	BBP02571	137809-97-3	2,3-Di(3',4'-methylenedioxybenzyl) -2-buten-4-olide	29-C2	352.337	10(μ)M	DMSO
NCRC41176	BBP02572	13878-92-7	Isomangiferolic acid	29-C3	456.7	10(μ)M	DMSO
NCRC41177	BBP02574	18465-71-9	2-C-Methyl-D-erythrono-1,4-lactone	29-C4	132.115	10(μ)M	DMSO
NCRC41178	BBP02577	32885-81-7	Lasiodiplodin	29-C5	292.37	10(μ)M	DMSO
NCRC41179	BBP02578	125002-91-7	8-Prenylluteone	29-C6	422.47	10(μ)M	DMSO
NCRC41180	BBP02579	1897-26-3	Akuammiline	29-C7	394.464	10(μ)M	DMSO
NCRC41181	BBP02580	5890-18-6	Laurolitsine	29-C8	313.348	10(μ)M	DMSO
NCRC41182	BBP02581	27270-89-9	Phyllostine	29-C9	154.12	10(μ)M	DMSO
NCRC41183	BBP02582	917-13-5	Enniatin B	29-C10	639.82	10(μ)M	DMSO
NCRC41184	BBP02583	1197-09-7	3',4'-Dihydroxyacetophenone	29-C11	152.147	10(μ)M	DMSO
NCRC41185	BBP02584	73483-88-2	Tetrachyrin	29-D2	300.435	10(μ)M	DMSO
NCRC41186	BBP02587	864719-17-5	Sequosempervirin B	29-D3	316.348	10(μ)M	DMSO
NCRC41187	BBP02589	3466-23-7	Dehydrodeguelin	29-D4	392.401	10(μ)M	DMSO
NCRC41188	BBP02590	77394-27-5	Sesartemin	29-D5	430.448	10(μ)M	DMSO
NCRC41189	BBP02591	28978-03-2	Persicoside	29-D6	478.446	10(μ)M	DMSO
NCRC41190	BBP02592	1264694-96-3	Metasequirin D	29-D7	346.374	10(μ)M	DMSO
NCRC41191	BBP02593	1188932-15-1	Alstoyunine E	29-D8	350.411	10(μ)M	DMSO
NCRC41192	BBP02594	1212148-58-7	Secodihydro-hydramicromelin B	29-D9	326.299	10(μ)M	DMSO
NCRC41193	BBP02595	31076-39-8	Cedeodarin	29-D10	318.278	10(μ)M	DMSO

NCRC41194	BBP02596	480-33-1	Mellein	29-D11	178.185	10(μ)M	DMSO
NCRC41195	BBP02597	65408-91-5	Altholactone	29-E2	232.232	10(μ)M	DMSO
NCRC41196	BBP02600	4449-55-2	Warangalone	29-E3	404.455	10(μ)M	DMSO
NCRC41197	BBP02601	482-53-1	Osajin	29-E4	404.455	10(μ)M	DMSO
NCRC41198	BBP02602	911004-72-3	Furowanin A	29-E5	438.47	10(μ)M	DMSO
NCRC41199	BBP02603	53734-74-0	Neorauflavane	29-E6	354.396	10(μ)M	DMSO
NCRC41200	BBP02604	123316-64-3	2'-O-Methylhelichrysetin	29-E7	300.306	10(μ)M	DMSO
NCRC41201	BBP02606	1309478-07-6	Wittifuran X	29-E8	272.253	10(μ)M	DMSO
NCRC41202	BBP02607	156-38-7	4-Hydroxyphenylacetic acid	29-E9	152.147	10(μ)M	DMSO
NCRC41203	BBP02608	88664-09-9	14-Deoxycoleon U	29-E10	330.418	10(μ)M	DMSO
NCRC41204	BBP02611	146-48-5	Yohimbine	29-E11	354.443	10(μ)M	DMSO
NCRC41205	BBP02612	28189-90-4	Sinensin	29-F2	450.393	10(μ)M	DMSO
NCRC41206	BBP02614	17811-32-4	3-Deoxyzinnolide	29-F3	262.301	10(μ)M	DMSO
NCRC41207	BBP02615	1083195-05-4	1,7-Bis(4-hydroxyphenyl)hept-6-en-3-ol	29-F4	298.376	10(μ)M	DMSO
NCRC41208	BBP02616	129214-59-1	15-Dihydroepioxylubimin	29-F5	254.365	10(μ)M	DMSO
NCRC41209	BBP02618	167958-89-6	Stigmasta-4,22-diene-3β,6β-diol	29-F6	428.69	10(μ)M	DMSO
NCRC41210	BBP02619	30368-42-4	Dalbergioidin	29-F7	288.252	10(μ)M	DMSO
NCRC41211	BBP02620	2030-53-7	Aporheine	29-F8	279.333	10(μ)M	DMSO
NCRC41212	BBP02621	150226-21-4	12-Deoxo-12α-acetoxyelliptone	29-F9	396.39	10(μ)M	DMSO
NCRC41213	BBP02622	522-17-8	Deguelin	29-F10	394.417	10(μ)M	DMSO
NCRC41214	BBP02623	51225-28-6	6,8-Diprenylgenistein	29-F11	406.471	10(μ)M	DMSO
NCRC41215	BBP02624	76-80-2	Tephrosin	29-G2	410.417	10(μ)M	DMSO
NCRC41216	BBP02625	120278-25-3	4-Hydroxysapriparaquinone	29-G3	330.418	10(μ)M	DMSO
NCRC41217	BBP02626	113900-75-7	Koumine N-oxide	29-G4	322.401	10(μ)M	DMSO
NCRC41218	BBP02627	20958-18-3	Dihydroisotanshinone I	29-G5	278.302	10(μ)M	DMSO
NCRC41219	BBP02628	15345-89-8	5,6-Dehydrokawain	29-G6	228.243	10(μ)M	DMSO
NCRC41220	BBP02629	24512-62-7	Gardenoside	29-G7	404.366	10(μ)M	DMSO
NCRC41221	BBP02630	85769-33-1	Alstolenine	29-G8	546.611	10(μ)M	DMSO
NCRC41222	BBP02631	135905-53-2	Lupinol C	29-G9	370.353	10(μ)M	DMSO
NCRC41223	BBP02633	1206734-95-3	2''-Acetylastragalin	29-G10	490.414	10(μ)M	DMSO
NCRC41224	BBP02634	6880-54-2	Norfluorocurarine	29-G11	292.375	10(μ)M	DMSO
NCRC41225	BBP02635	2447-70-3	Pseudoakuammigine	29-H2	366.453	10(μ)M	DMSO
NCRC41226	BBP02638	240122-30-9	Griffithazanone A	29-H3	257.241	10(μ)M	DMSO
NCRC41227	BBP02640	122590-03-8	11-Hydroxyrankinidine	29-H4	356.416	10(μ)M	DMSO
NCRC41228	BBP02641	552-59-0	Prunetin	29-H5	284.263	10(μ)M	DMSO
NCRC41229	BBP02642	370102-93-5	(-)-Variabilin	29-H6	300.306	10(μ)M	DMSO
NCRC41230	BBP02643	13401-40-6	Phaseollin	29-H7	322.355	10(μ)M	DMSO
NCRC41231	BBP02644	17990-42-0	Oleanonic acid	29-H8	454.684	10(μ)M	DMSO
NCRC41232	BBP02645	511-05-7	Sugiol	29-H9	300.435	10(μ)M	DMSO
NCRC41233	BBP02647	2466-42-4	Neolitsine	29-H10	323.343	10(μ)M	DMSO
NCRC41234	BBP02649	116064-76-7	Piperolactam C	29-H11	309.316	10(μ)M	DMSO
NCRC41235	BBP02454	51838-83-6	Allamandicin	30-A2	308.283	10(μ)M	DMSO
NCRC41236	BBP02598	144429-71-0	Goniodiol 8-acetate	30-A3	276.285	10(μ)M	DMSO
NCRC41237	BBP02650	118-71-8	Maltol	30-A4	126.11	10(μ)M	DMSO
NCRC41238	BBP02651	58124-18-8	2-Hydroxynaringenin	30-A5	288.252	10(μ)M	DMSO
NCRC41239	BBP02656	250691-57-7	Salvisyrianone	30-A6	312.403	10(μ)M	DMSO
NCRC41240	BBP02657	83348-22-5	10-O-Caffeoyl-6-epiferetoside	30-A7	566.508	10(μ)M	DMSO
NCRC41241	BBP02658	63786-17-2	12α-Hydroxygrandiflorenic acid	30-A8	316.435	10(μ)M	DMSO
NCRC41242	BBP02660	32262-18-3	4-Methoxy-N-methyl-2-quinolone	30-A9	189.211	10(μ)M	DMSO
NCRC41243	BBP02661	124868-11-7	Isoaltholactone	30-A10	232.232	10(μ)M	DMSO
NCRC41244	BBP02662	1038753-13-7	4'-O-Methyllicoflavanone	30-A11	354.396	10(μ)M	DMSO
NCRC41245	BBP02663	77263-06-0	Erythrabyssin II	30-B2	392.487	10(μ)M	DMSO
NCRC41246	BBP02664	1246926-09-9	5'-Prenylaliarin	30-B3	484.538	10(μ)M	DMSO

NCRC41247	BBP02665	22697-65-0	5,7,4'-Trihydroxy-3,6-dimethoxyflavone	30-B4	330.289	10(μ)M	DMSO
NCRC41248	BBP02667	75679-58-2	2',4'-Dihydroxy-4,6'-dimethoxydihydrochalcone	30-B5	302.322	10(μ)M	DMSO
NCRC41249	BBP02668	487-06-9	Citropten	30-B6	206.195	10(μ)M	DMSO
NCRC41250	BBP02669	87585-32-8	(+)-Lyoniresinol 9'-O-glucoside	30-B7	582.594	10(μ)M	DMSO
NCRC41251	BBP02672	165459-53-0	16-Hydroxy-2-oxocleroda-3,13-dien-15,16-olide	30-B8	332.434	10(μ)M	DMSO
NCRC41252	BBP02673	87440-56-0	Glepidotin B	30-B9	340.37	10(μ)M	DMSO
NCRC41253	BBP02674	521-51-7	Pedicin	30-B10	330.332	10(μ)M	DMSO
NCRC41254	BBP02675	No	Dodonaflavonol	30-B11	512.548	10(μ)M	DMSO
NCRC41255	BBP02676	92280-12-1	Sanggenon N	30-C2	422.47	10(μ)M	DMSO
NCRC41256	BBP02678	832-58-6	2',4',6'-Trimethoxyacetophenone	30-C3	210.226	10(μ)M	DMSO
NCRC41257	BBP02679	53734-75-1	Neorauflavene	30-C4	352.38	10(μ)M	DMSO
NCRC41258	BBP02680	126737-42-6	Acetylsventenic acid	30-C5	360.487	10(μ)M	DMSO
NCRC41259	BBP02681	82375-30-2	Humantenirine	30-C6	370.442	10(μ)M	DMSO
NCRC41260	BBP02682	23811-50-9	Trichokaurin	30-C7	434.523	10(μ)M	DMSO
NCRC41261	BBP02683	22149-65-1	Hopane-3β,22-diol	30-C8	444.733	10(μ)M	DMSO
NCRC41262	BBP02684	19914-20-6	Enniatin B1	30-C9	653.847	10(μ)M	DMSO
NCRC41263	BBP02685	97399-90-1	Aristolactam AIa	30-C10	281.263	10(μ)M	DMSO
NCRC41264	BBP02686	117469-56-4	Prionitin	30-C11	310.43	10(μ)M	DMSO
NCRC41265	BBP02687	125180-42-9	N-Methoxyanhydrovobasinediol	30-D2	338.443	10(μ)M	DMSO
NCRC41266	BBP02688	1017233-48-5	6α,16,18-Trihydroxycleroda -3,13-dien-15,16-olide	30-D3	350.449	10(μ)M	DMSO
NCRC41267	BBP02689	119309-02-3	Atalantoflavone	30-D4	336.338	10(μ)M	DMSO
NCRC41268	BBP02690	161099-42-9	Bidwillol A	30-D5	338.397	10(μ)M	DMSO
NCRC41269	BBP02691	926010-24-4	Dayecrystal A	30-D6	320.466	10(μ)M	DMSO
NCRC41270	BBP02694	20784-50-3	Isobavachalcone	30-D7	324.37	10(μ)M	DMSO
NCRC41271	BBP02695	4657-58-3	Cycloartanol	30-D8	428.733	10(μ)M	DMSO
NCRC41272	BBP02696	No	Oxyperaksine	30-D9	364.48	10(μ)M	DMSO
NCRC41273	BBP02697	959421-20-6	5,7,4'-Trihydroxy-3,6-dimethoxy-3'-prenylflavone	30-D10	398.406	10(μ)M	DMSO
NCRC41274	BBP02698	118-41-2	Eudesmic acid	30-D11	212.199	10(μ)M	DMSO
NCRC41275	BBP02699	59-02-9	α-Tocopherol	30-E2	430.706	10(μ)M	DMSO
NCRC41276	BBP02700	72458-85-6	11-Hydroxytephrosin	30-E3	426.416	10(μ)M	DMSO
NCRC41277	BBP02701	1438-62-6	13-Epimanool	30-E4	290.483	10(μ)M	DMSO
NCRC41278	BBP02702	122590-04-9	11-Hydroxyhumantenine	30-E5	370.442	10(μ)M	DMSO
NCRC41279	BBP02704	28342-33-8	Oxychelerythrine	30-E6	363.363	10(μ)M	DMSO
NCRC41280	BBP02705	20186-22-5	Pisatin	30-E7	314.289	10(μ)M	DMSO
NCRC41281	BBP02706	1092952-62-9	7,2',4'-Trihydroxy-5-methoxy-3-phenylcoumarin	30-E8	300.263	10(μ)M	DMSO
NCRC41282	BBP02708	210537-04-5	1,11b-Dihydro-11b-hydroxymedicarpin	30-E9	288.295	10(μ)M	DMSO
NCRC41283	BBP02709	68401-05-8	Kuwanon E	30-E10	424.486	10(μ)M	DMSO
NCRC41284	BBP02710	210537-05-6	1,11b-Dihydro-11b-hydroxymaackiain	30-E11	302.279	10(μ)M	DMSO
NCRC41285	BBP02711	124096-81-7	16-Epikoumidine	30-F2	294.391	10(μ)M	DMSO
NCRC41286	BBP02712	27510-33-4	O-Methylpallidine	30-F3	341.401	10(μ)M	DMSO
NCRC41287	BBP02713	6812-87-9	Royleanone	30-F4	316.435	10(μ)M	DMSO
NCRC41288	BBP02714	1372527-39-3	Dodoviscin H	30-F5	454.512	10(μ)M	DMSO
NCRC41289	BBP02715	84294-77-9	Aliarin	30-F6	416.421	10(μ)M	DMSO
NCRC41290	BBP02716	1219603-97-0	4,5-Diepipsidial A	30-F7	474.588	10(μ)M	DMSO
NCRC41291	BBP02717	5876-17-5	Haplopine	30-F8	245.231	10(μ)M	DMSO
NCRC41292	BBP02719	3690-05-9	p-Coumaryl alcohol	30-F9	150.174	10(μ)M	DMSO
NCRC41293	BBP02720	126778-79-8	Sventenic acid	30-F10	318.45	10(μ)M	DMSO

NCRC41294	BBP02721	53948-07-5	Aristolactam AII	30-F11	265.263	10(μ)M	DMSO
NCRC41295	BBP02722	145643-96-5	Cyclocommunol	30-G2	352.337	10(μ)M	DMSO
NCRC41296	BBP02724	488-17-5	3-Methylcatechol	30-G3	124.137	10(μ)M	DMSO
NCRC41297	BBP02725	1372527-40-6	Dodoviscin I	30-G4	386.395	10(μ)M	DMSO
NCRC41298	BBP02726	1372527-25-7	Dodoviscin A	30-G5	500.538	10(μ)M	DMSO
NCRC41299	BBP02728	71850-15-2	Camaldulenic acid	30-G6	470.684	10(μ)M	DMSO
NCRC41300	BBP02729	865187-17-3	Gelsempervine A	30-G7	382.453	10(μ)M	DMSO
NCRC41301	BBP02730	139954-00-0	Palbinone	30-G8	358.471	10(μ)M	DMSO
NCRC41302	BBP02731	136685-37-5	9-Deoxygoniopypyrone	30-G9	234.248	10(μ)M	DMSO
NCRC41303	BBP02732	20486-27-5	Procumbide	30-G10	362.329	10(μ)M	DMSO
NCRC41304	BBP02734	142763-37-9	3,4-Didehydrosapriparaquione	30-G11	312.403	10(μ)M	DMSO
NCRC41305	BBP02735	80454-42-8	Paeoniflorigenone	30-H2	318.321	10(μ)M	DMSO
NCRC41306	BBP02739	69651-80-5	Hesperetin 5-O-glucoside	30-H3	464.419	10(μ)M	DMSO
NCRC41307	BBP02740	74515-47-2	Cristacarpin	30-H4	354.396	10(μ)M	DMSO
NCRC41308	BBP02741	6935-99-5	Obtucarbamate A	30-H5	238.24	10(μ)M	DMSO
NCRC41309	BBP02742	20913-18-2	Obtucarbamate B	30-H6	238.24	10(μ)M	DMSO
NCRC41310	BBP02744	482-45-1	Isoimperatorin	30-H7	270.28	10(μ)M	DMSO
NCRC41311	BBP02747	771493-42-6	6α-Hydroxycleroda-3,13-dien-16,15-olid-18-oic acid	30-H8	348.433	10(μ)M	DMSO
NCRC41312	BBP02749	61135-92-0	6a-Hydroxymedicarpin	30-H9	286.279	10(μ)M	DMSO
NCRC41313	BBP02750	114027-38-2	16-Epivoacarpine	30-H10	368.426	10(μ)M	DMSO
NCRC41314	BBP02751	39986-86-2	4'-Hydroxy-5,6-dehydrokawain	30-H11	244.243	10(μ)M	DMSO
NCRC41315	BBP02605	96422-53-6	Goniodiol 7-acetate	31-A2	276.285	10(μ)M	DMSO
NCRC41316	BBP02752	123702-94-3	Kuwanol C	31-A3	422.47	10(μ)M	DMSO
NCRC41317	BBP02753	51593-96-5	Cuspidiol	31-A4	236.307	10(μ)M	DMSO
NCRC41318	BBP02754	200813-31-6	16-Nor-15-oxodehydroabietic acid	31-A5	300.392	10(μ)M	DMSO
NCRC41319	BBP02755	90468-72-7	Maoyerabdosin	31-A6	468.537	10(μ)M	DMSO
NCRC41320	BBP02757	91913-76-7	1-Methoxyindole-3-carboxylic acid	31-A7	191.183	10(μ)M	DMSO
NCRC41321	BBP02758	61218-44-8	6a-Hydroxymaackiain	31-A8	300.263	10(μ)M	DMSO
NCRC41322	BBP02760	206560-99-8	7-O-Methylporiol	31-A9	300.306	10(μ)M	DMSO
NCRC41323	BBP02764	63910-76-9	Panaxydiol	31-A10	260.371	10(μ)M	DMSO
NCRC41324	BBP02765	479-13-0	Coumestrol	31-A11	268.221	10(μ)M	DMSO
NCRC41325	BBP02766	55610-01-0	Cepharadione A	31-B2	305.284	10(μ)M	DMSO
NCRC41326	BBP02768	105330-59-4	5,7,4'-Tri-O-methylcatechin	31-B3	332.348	10(μ)M	DMSO
NCRC41327	BBP02769	151334-06-4	3-O-p-Coumaroyloleanolic acid	31-B4	602.843	10(μ)M	DMSO
NCRC41328	BBP02772	246870-75-7	Derrisisoflavone B	31-B5	422.47	10(μ)M	DMSO
NCRC41329	BBP02773	136778-40-0	Goniodiol diacetate	31-B6	318.321	10(μ)M	DMSO
NCRC41330	BBP02774	129578-07-0	Goniopypyrone	31-B7	250.247	10(μ)M	DMSO
NCRC41331	BBP02775	17948-42-4	Venoterpine	31-B8	149.19	10(μ)M	DMSO
NCRC41332	BBP02778	96422-52-5	Goniodiol	31-B9	234.248	10(μ)M	DMSO
NCRC41333	BBP02779	97534-10-6	3-O-Caffeoyloleanolic acid	31-B10	618.842	10(μ)M	DMSO
NCRC41334	BBP02780	227289-51-2	(3S,7S)-5,6-Dehydro-4''-de-O-methylcentrolobine	31-B11	296.36	10(μ)M	DMSO
NCRC41335	BBP02781	30536-48-2	Caulilexin C	31-C2	186.21	10(μ)M	DMSO
NCRC41336	BBP02783	883859-83-4	8-Lavandulylkaempferol	31-C3	422.47	10(μ)M	DMSO
NCRC41337	BBP02784	94596-28-8	Senkyunolide I	31-C4	224.253	10(μ)M	DMSO
NCRC41338	BBP02785	76122-57-1	Phaseollidin hydrate	31-C5	342.386	10(μ)M	DMSO
NCRC41339	BBP02786	60976-49-0	Geraniin	31-C6	952.645	10(μ)M	DMSO
NCRC41340	BBP02789	98751-78-1	Paeonilactone B	31-C7	196.2	10(μ)M	DMSO
NCRC41341	BBP02790	98751-77-0	Paeonilactone C	31-C8	318.321	10(μ)M	DMSO
NCRC41342	BBP02791	133442-54-3	Angophorol	31-C9	314.333	10(μ)M	DMSO
NCRC41343	BBP02792	27468-20-8	Deoxyneocryptotanshinone	31-C10	298.376	10(μ)M	DMSO
NCRC41344	BBP02793	221002-11-5	1''-Methoxyerythrinin C	31-C11	384.379	10(μ)M	DMSO

NCRC41345	BBP02794	35354-74-6	Honokiol	31-D2	266.334	10(μ)M	DMSO
NCRC41346	BBP02795	98751-79-2	Paeonilactone A	31-D3	198.216	10(μ)M	DMSO
NCRC41347	BBP02796	52151-92-5	Piperitol	31-D4	356.369	10(μ)M	DMSO
NCRC41348	BBP02797	1916-07-0	Methyl 3,4,5-trimethoxybenzoate	31-D5	226.226	10(μ)M	DMSO
NCRC41349	BBP02798	94451-48-6	Syzalterin	31-D6	298.29	10(μ)M	DMSO
NCRC41350	BBP02799	80621-54-1	8-Demethylsideroxylin	31-D7	298.29	10(μ)M	DMSO
NCRC41351	BBP02800	60297-37-2	Auriculasin	31-D8	420.454	10(μ)M	DMSO
NCRC41352	BBP02801	59901-98-3	3-Hydroxy-8,9-methylenedioxypterocarpene	31-D9	282.248	10(μ)M	DMSO
NCRC41353	BBP02803	754919-24-9	N-Methylnuciferine	31-D10	310.41	10(μ)M	DMSO
NCRC41354	BBP02804	53846-50-7	8-Prenylnaringenin	31-D11	340.37	10(μ)M	DMSO
NCRC41355	BBP02805	82373-94-2	2,3,5,4'-Tetrahydroxystilbene 2-O-glucoside	31-E2	406.383	10(μ)M	DMSO
NCRC41356	BBP02807	6178-44-5	Ethyl 3,4,5-trimethoxybenzoate	31-E3	240.252	10(μ)M	DMSO
NCRC41357	BBP02808	975-77-9	Pericyclivine	31-E4	322.401	10(μ)M	DMSO
NCRC41358	BBP02809	76-98-2	Conopharyngine	31-E5	398.495	10(μ)M	DMSO
NCRC41359	BBP02810	16790-93-5	19(S)-Hydroxyconopharyngine	31-E6	414.495	10(μ)M	DMSO
NCRC41360	BBP02811	220736-54-9	Curlignan	31-E7	360.358	10(μ)M	DMSO
NCRC41361	BBP02812	478158-77-9	Isoerysenegalensein E	31-E8	422.47	10(μ)M	DMSO
NCRC41362	BBP02813	154992-17-3	Erysenegalensein E	31-E9	422.47	10(μ)M	DMSO
NCRC41363	BBP02815	1151862-67-7	6-Epiharpagoside	31-E10	494.488	10(μ)M	DMSO
NCRC41364	BBP02818	1072-93-1	Epigoitrin	31-E11	129.18	10(μ)M	DMSO
NCRC41365	BBP02820	99217-63-7	Kushenol A	31-F2	408.487	10(μ)M	DMSO
NCRC41366	BBP02821	51-34-3	Scopolamine	31-F3	303.353	10(μ)M	DMSO
NCRC41367	BBP02822	4684-28-0	Norscopolamine	31-F4	289.326	10(μ)M	DMSO
NCRC41368	BBP02823	18642-23-4	Psoralidin	31-F5	336.338	10(μ)M	DMSO
NCRC41369	BBP02824	23313-21-5	Anthraglycoside B	31-F6	432.378	10(μ)M	DMSO
NCRC41370	BBP02825	89706-39-8	7-O-Acetyl-4-O-demethylpolysyphorin	31-F7	446.49	10(μ)M	DMSO
NCRC41371	BBP02826	1245636-01-4	ent-Labda-8(17),13Z-diene-15,16,19-triol 19-O-glucoside	31-F8	484.623	10(μ)M	DMSO
NCRC41372	BBP02827	147663-91-0	Magnolianin	31-F9	826.97	10(μ)M	DMSO
NCRC41373	BBP02828	56121-44-9	4-O-Methylhelichrysetin	31-F10	300.306	10(μ)M	DMSO
NCRC41374	BBP02831	61617-29-6	Songoroside A	31-F11	588.815	10(μ)M	DMSO
NCRC41375	BBP02832	118477-06-8	Cyclo(L-Phe-trans-4-hydroxy-L-Pro)	31-G2	260.288	10(μ)M	DMSO
NCRC41376	BBP02833	No	2-Ethoxy-3-acetonyltaxifolin	31-G3	404.367	10(μ)M	DMSO
NCRC41377	BBP02834	1044743-35-2	3'-Methylflavokawin	31-G4	314.333	10(μ)M	DMSO
NCRC41378	BBP02835	59086-93-0	Dehydrotoxicarol	31-G5	408.401	10(μ)M	DMSO
NCRC41379	BBP02836	174204-83-2	3-Chloro-4-hydroxypiperidin-2-one	31-G6	149.576	10(μ)M	DMSO
NCRC41380	BBP02838	89020-11-1	Dregeoside A11	31-G7	1101.27	10(μ)M	DMSO
NCRC41381	BBP02839	115006-86-5	Cyclo(L-Leu-trans-4-hydroxy-L-Pro)	31-G8	226.272	10(μ)M	DMSO
NCRC41382	BBP02840	84873-15-4	p-Hydroxyphenethyl trans-ferulate	31-G9	314.333	10(μ)M	DMSO
NCRC41383	BBP02841	480-66-0	2',4',6'-Trihydroxyacetophenone	31-G10	168.147	10(μ)M	DMSO
NCRC41384	BBP02842	90536-74-6	Ethyl 2,4,6-trihydroxybenzoate	31-G11	198.173	10(μ)M	DMSO
NCRC41385	BBP02843	486-84-0	Harman	31-H2	182.221	10(μ)M	DMSO
NCRC41386	BBP02847	63644-71-3	γ-Methoxyisoeugenol	31-H3	194.227	10(μ)M	DMSO
NCRC41387	BBP02848	No	Ethyl 9,12,13-trihydroxyoctadeca-10,15-dienoate	31-H4	356.497	10(μ)M	DMSO
NCRC41388	BBP02849	186140-36-3	11α,12α-Epoxy-3β,23-dihydroxy-30-norolean-20(29)-en-28,13β-olide	31-H5	470.641	10(μ)M	DMSO
NCRC41389	BBP02850	6519-27-3	(16R)-E-Isositsirikine	31-H6	354.443	10(μ)M	DMSO

NCRC41390	BBP02851	89199-99-5	Mulberrofuran H	31-H7	442.46	10(μ)M	DMSO
NCRC41391	BBP02853	464-45-9	Borneol	31-H8	154.249	10(μ)M	DMSO
NCRC41392	BBP02854	82513-70-0	10-Hydroxy-16-epiaffinine	31-H9	340.416	10(μ)M	DMSO
NCRC41393	BBP02855	87562-14-9	Randaiol	31-H10	242.27	10(μ)M	DMSO
NCRC41394	BBP02856	1280602-81-4	erythro-1-(4-Hydroxy-3-methoxyphenyl)propane-1,2-	31-H11	198.216	10(μ)M	DMSO
NCRC41395	BBP02857	848031-94-7	threo-1-(4-Hydroxy-3-methoxyphenyl)propane-1,2-	32-A2	198.216	10(μ)M	DMSO
NCRC41396	BBP02859	16049-28-8	Antirhine	32-A3	296.407	10(μ)M	DMSO
NCRC41397	BBP02860	123702-97-6	Moracin O	32-A4	326.343	10(μ)M	DMSO
NCRC41398	BBP02861	112448-69-8	3-Phenyl-1-(pyrrol-1-yl)propan-1-one	32-A5	199.248	10(μ)M	DMSO
NCRC41399	BBP02862	448905-82-6	3-(4-Methoxyphenyl)-1-(pyrrol-1-yl)propan-1-one	32-A6	229.274	10(μ)M	DMSO
NCRC41400	BBP02863	252248-89-8	1-Cinnamoylpyrrole	32-A7	197.233	10(μ)M	DMSO
NCRC41401	BBP02864	72917-31-8	(Z)-Butylidenephthalide	32-A8	188.222	10(μ)M	DMSO
NCRC41402	BBP02865	94596-27-7	Senkyunolide H	32-A9	224.253	10(μ)M	DMSO
NCRC41403	BBP02868	87085-00-5	Mulberrofuran G	32-A10	562.565	10(μ)M	DMSO
NCRC41404	BBP02869	102841-46-3	Moracin P	32-A11	326.343	10(μ)M	DMSO
NCRC41405	BBP02872	26488-24-4	Cyclo(D-Phe-L-Pro)	32-B2	244.289	10(μ)M	DMSO
NCRC41406	BBP02873	36357-32-1	Cyclo(L-Ala-L-Pro)	32-B3	168.193	10(μ)M	DMSO
NCRC41407	BBP02874	6052-73-9	5,6-Dihydropyridin-2(1H)-one	32-B4	97.1152	10(μ)M	DMSO
NCRC41408	BBP02875	19202-36-9	Hinokiflavone	32-B5	538.458	10(μ)M	DMSO
NCRC41409	BBP02876	41060-15-5	Neobavaisoflavone	32-B6	322.355	10(μ)M	DMSO
NCRC41410	BBP02877	19367-38-5	Methyl 4-hydroxycinnamate	32-B7	178.185	10(μ)M	DMSO
NCRC41411	BBP02882	124858-37-3	5-Dehydroxyparatocarpin K	32-B8	322.355	10(μ)M	DMSO
NCRC41412	BBP02885	548-19-6	Isoginkgetin	32-B9	566.511	10(μ)M	DMSO
NCRC41413	BBP02887	130263-10-4	3'-Demethoxypiplartine	32-B10	287.31	10(μ)M	DMSO
NCRC41414	BBP02888	514-62-5	Ferruginol	32-B11	286.452	10(μ)M	DMSO
NCRC41415	BBP02890	188894-19-1	Junipediol B 8-O-glucoside	32-C2	358.34	10(μ)M	DMSO
NCRC41416	BBP02891	22329-76-6	Methyl ferulate	32-C3	208.211	10(μ)M	DMSO
NCRC41417	BBP02892	2316-26-9	3,4-Dimethoxycinnamic acid	32-C4	208.211	10(μ)M	DMSO
NCRC41418	BBP02893	29376-68-9	Thevetiaflavone	32-C5	284.263	10(μ)M	DMSO
NCRC41419	BBP02894	865887-46-3	Oleuropeic acid 8-O-glucoside	32-C6	346.373	10(μ)M	DMSO
NCRC41420	BBP02895	66568-97-6	Tsugafolin	32-C7	300.306	10(μ)M	DMSO
NCRC41421	BBP02896	736140-70-8	1-(4-Methoxycinnamoyl)pyrrole	32-C8	227.259	10(μ)M	DMSO
NCRC41422	BBP02897	36238-67-2	Cyclo(D-Leu-L-Pro)	32-C9	210.273	10(μ)M	DMSO
NCRC41423	BBP02899	126640-98-0	16-Epinormacusine B	32-C10	294.391	10(μ)M	DMSO
NCRC41424	BBP02900	775351-88-7	Corylifol A	32-C11	390.472	10(μ)M	DMSO
NCRC41425	BBP02901	958631-84-0	(E)-1-(4-Hydroxyphenyl)dec-1-en-3-one	32-D2	246.345	10(μ)M	DMSO
NCRC41426	BBP02902	7727-79-9	Zederone	32-D3	246.302	10(μ)M	DMSO
NCRC41427	BBP02903	77996-04-4	Mulberrofuran C	32-D4	580.581	10(μ)M	DMSO
NCRC41428	BBP02904	72500-11-9	Methyl vanillate glucoside	32-D5	344.314	10(μ)M	DMSO
NCRC41429	BBP02907	34421-19-7	2,3-Dihydrosciadopitysin	32-D6	582.554	10(μ)M	DMSO
NCRC41430	BBP02908	828923-27-9	2,3-Dihydroisoginkgetin	32-D7	568.527	10(μ)M	DMSO
NCRC41431	BBP02910	128261-84-7	1-(3,4-Dimethoxycinnamoyl)piperidine	32-D8	275.343	10(μ)M	DMSO
NCRC41432	BBP02912	270249-38-2	2'-O-Methylkurarinone	32-D9	452.539	10(μ)M	DMSO
NCRC41433	BBP02913	22767-72-2	Ethyl 3-(4-methoxyphenyl)propanoate	32-D10	208.254	10(μ)M	DMSO
NCRC41434	BBP02915	20575-57-9	Calycosin	32-D11	284.263	10(μ)M	DMSO
NCRC41435	BBP02916	24338-53-2	Nagilactone C	32-E2	362.374	10(μ)M	DMSO
NCRC41436	BBP02917	116271-35-3	Pyrroside B	32-E3	566.508	10(μ)M	DMSO
NCRC41437	BBP02918	18836-52-7	Pellitorine	32-E4	223.354	10(μ)M	DMSO

NCRC41438	BBP02920	97938-30-2	Sophoraflavanone G	32-E5	424.486	10(μ)M	DMSO
NCRC41439	BBP02921	272122-56-2	Decursidate	32-E6	330.332	10(μ)M	DMSO
NCRC41440	BBP02922	104691-86-3	Lupiwighteone	32-E7	338.354	10(μ)M	DMSO
NCRC41441	BBP02923	27127-79-3	Thevetin B	32-E8	858.963	10(μ)M	DMSO
NCRC41442	BBP02925	331-39-5	Caffeic acid	32-E9	180.157	10(μ)M	DMSO
NCRC41443	BBP02926	38965-51-4	Eriodictyol 7-O-glucoside	32-E10	450.393	10(μ)M	DMSO
NCRC41444	BBP02929	28610-31-3	8-Prenylkaempferol	32-E11	354.353	10(μ)M	DMSO
NCRC41445	BBP02932	20188-85-6	Ombuoside	32-F2	638.571	10(μ)M	DMSO
NCRC41446	BBP02933	15823-04-8	Methyl 3-(4-methoxyphenyl)propanoate	32-F3	194.227	10(μ)M	DMSO
NCRC41447	BBP02934	5027-30-5	Phloracetophenone 4'-O-glucoside	32-F4	330.287	10(μ)M	DMSO
NCRC41448	BBP02935	67685-22-7	Anhydroglycinol	32-F5	254.238	10(μ)M	DMSO
NCRC41449	BBP02937	38642-49-8	Benzoylpaeoniflorin	32-F6	584.568	10(μ)M	DMSO
NCRC41450	BBP02939	572-32-7	Ayanin	32-F7	344.315	10(μ)M	DMSO
NCRC41451	BBP02940	501-52-0	Hydrocinnamic acid	32-F8	150.174	10(μ)M	DMSO
NCRC41452	BBP02942	23180-57-6	Paeoniflorin	32-F9	480.462	10(μ)M	DMSO
NCRC41453	BBP02952	190381-82-9	Orientanol A	32-F10	388.411	10(μ)M	DMSO
NCRC41454	BBP02953	514-10-3	Abietic acid	32-F11	302.451	10(μ)M	DMSO
NCRC41455	BBP02956	55102-39-1	4β-Carboxy-19-nortotarol	32-G2	316.435	10(μ)M	DMSO
NCRC41456	BBP02959	178765-54-3	3-Hydroxybakuchiol	32-G3	272.382	10(μ)M	DMSO
NCRC41457	BBP02962	47326-53-4	Spegatrine	32-G4	325.424	10(μ)M	DMSO
NCRC41458	BBP02963	489-32-7	Icariin	32-G5	676.662	10(μ)M	DMSO
NCRC41459	BBP02964	475-81-0	Glaucine	32-G6	355.428	10(μ)M	DMSO
NCRC41460	BBP02966	33417-17-3	3-Hydroxy-3-acetonyloxindole	32-G7	205.21	10(μ)M	DMSO
NCRC41461	BBP02968	6877-32-3	Corynoxine	32-G8	384.469	10(μ)M	DMSO
NCRC41462	BBP02969	93753-33-4	Magnaldehyde D	32-G9	254.281	10(μ)M	DMSO
NCRC41463	BBP02972	56083-03-5	Isobavachromene	32-G10	322.355	10(μ)M	DMSO
NCRC41464	BBP02974	20013-76-7	Dehydrochromolaenin	32-G11	210.271	10(μ)M	DMSO
NCRC41465	BBP02979	5041-82-7	Isorhamnetin 3-O-glucoside	32-H2	478.403	10(μ)M	DMSO
NCRC41466	BBP02980	93-15-2	Methyleugenol	32-H3	178.228	10(μ)M	DMSO
NCRC41467	BBP02981	143601-07-4	Acuminatin	32-H4	512.505	10(μ)M	DMSO
NCRC41468	BBP02986	101236-50-4	Kushenol L	32-H5	440.486	10(μ)M	DMSO
NCRC41469	BBP02990	65332-45-8	Demethylvestitol	32-H6	258.269	10(μ)M	DMSO
NCRC41470	BBP02993	3064-05-9	Cycloolivil	32-H7	376.4	10(μ)M	DMSO
NCRC41471	BBP02994	21913-98-4	3'-Methoxydaidzein	32-H8	284.263	10(μ)M	DMSO
NCRC41472	BBP02995	137787-00-9	Boeravinone E	32-H9	328.273	10(μ)M	DMSO
NCRC41473	BBP02996	93673-81-5	Magnolignan A	32-H10	300.349	10(μ)M	DMSO
NCRC41474	BBP02998	52438-21-8	1-Cinnamoylpyrrolidine	32-H11	201.264	10(μ)M	DMSO
27-A2	BBP02866	2122-36-3	Apparicine	33-A2	264.4	10(μ)M	DMSO
27-A3	BBP02870	63976-69-2	13-Oxopodocarp-8(14)-en-18-oic acid	33-A3	276.371	10(μ)M	DMSO
27-A4	BBP02871	852385-13-8	6-Methyl-7-O-	33-A4	316.31	10(μ)M	DMSO
27-A5	BBP02878	27483-18-7	Cyclo(D-Val-L-Pro)	33-A5	196.25	10(μ)M	DMSO
27-A6	BBP02879	7159-86-6	3α,6β-Ditigloyloxytropan-7β-ol	33-A6	337.416	10(μ)M	DMSO
27-A7	BBP02880	6711-69-9	Tubotaiwine	33-A7	324.4	10(μ)M	DMSO
27-A8	BBP02883	3778-25-4	7''-O-Methylsciadopitysin	33-A8	594.6	10(μ)M	DMSO
27-A9	BBP02884	110382-42-8	2,3-Dihydroheveaflavone	33-A9	582.6	10(μ)M	DMSO
27-A10	BBP02886	72959-46-7	6,7-Dihydroneridienone A	33-A10	328.5	10(μ)M	DMSO
27-A11	BBP02898	3705-26-8	Cyclo(L-Phe-L-Pro)	33-A11	244.3	10(μ)M	DMSO
27-B2	BBP02914	6831-17-0	Aristolone	33-B2	218.34	10(μ)M	DMSO
27-B3	BBP02924	19452-84-7	Taberpsychine	33-B3	308.425	10(μ)M	DMSO
27-B4	BBP02928	19891-51-1	Nagilactone B	33-B4	364.4	10(μ)M	DMSO
27-B5	BBP02930	59-48-3	Oxindole	33-B5	133.15	10(μ)M	DMSO
27-B6	BBP02936	107633-69-2	Dehydroformouregine	33-B6	337.4	10(μ)M	DMSO

27-B7	BBP02938	158642-42-3	Yixingensin	33-B7	492.43	10(μ)M	DMSO
27-B8	BBP02944	3705-27-9	Cyclo(Gly-L-Pro)	33-B8	154.169	10(μ)M	DMSO
27-B9	BBP02946	113773-90-3	Eudesm-4(15)-ene-3α,11-diol	33-B9	238.4	10(μ)M	DMSO
27-B10	BBP02947	873999-88-3	2,3-Dihydroamentoflavone 7,4'-dimethyl ether	33-B10	568.5	10(μ)M	DMSO
27-B11	BBP02949	109471-13-8	3'-Methyl-4-O-	33-B11	314.33	10(μ)M	DMSO
27-C2	BBP02951	333798-10-0	9-O-Methyl-4-hydroxyboeravinone B	33-C2	342.3	10(μ)M	DMSO
27-C3	BBP02957	913690-46-7	1''-Hydroxyerythrinin C	33-C3	370.4	10(μ)M	DMSO
27-C4	BBP02958	20013-75-6	Pyrocurzerenone	33-C4	212.292	10(μ)M	DMSO
27-C5	BBP02971	125072-69-7	Epinortrachelogenin	33-C5	374.4	10(μ)M	DMSO
27-C6	BBP02973	485811-84-5	Mirabijalone D	33-C6	342.3	10(μ)M	DMSO
27-C7	BBP02977	16790-92-4	Crassanine	33-C7	414.5	10(μ)M	DMSO
27-C8	BBP02982	1411629-26-9	Epiguajadial B	33-C8	474.6	10(μ)M	DMSO
27-C9	BBP02987	67349-43-3	Pterokaurane R	33-C9	322.5	10(μ)M	DMSO
27-C10	BBP02988	30435-26-8	Pelirine	33-C10	354.5	10(μ)M	DMSO
27-C11	BBP02989	1207861-69-5	Gynosaponin I	33-C11	769	10(μ)M	DMSO
27-D2	BBP02992	163060-07-9	Lup-20(29)-ene-3β,23-diol	33-D2	442.7	10(μ)M	DMSO
27-D3	BBP02997	93697-42-8	Magnolignan C	33-D3	300.4	10(μ)M	DMSO
27-D4	BBP02999	73069-13-3	Atractylenolide I	33-D4	230.3	10(μ)M	DMSO
27-D5	BBP03000	15486-34-7	Kaempferol 3,7,4'-trimethyl ether	33-D5	328.3	10(μ)M	DMSO
27-D6	BBP03001	171817-95-1	2-(1H-Indole-3-carboxamido)benzoic acid	33-D6	280.283	10(μ)M	DMSO
27-D7	BBP03002	162229-27-8	Dorsmanin A	33-D7	324.4	10(μ)M	DMSO
27-D8	BBP03003	160568-14-9	Zarzissine	33-D8	135.1	10(μ)M	DMSO
27-D9	BBP03005	118525-40-9	Icaritin	33-D9	368.38	10(μ)M	DMSO
27-D10	BBP03006	113558-15-9	Icariside II	33-D10	514.527	10(μ)M	DMSO
27-D11	BBP03010	152464-78-3	Leachianone G	33-D11	356.4	10(μ)M	DMSO
27-E2	BBP03012	6859-01-4	Isorhynchophylline	33-E2	384.47	10(μ)M	DMSO
27-E3	BBP03013	41682-21-7	8-Methyleugenitol	33-E3	220.2	10(μ)M	DMSO
27-E4	BBP03014	70872-29-6	Isoxanthohumol	33-E4	354.402	10(μ)M	DMSO
27-E5	BBP03015	118266-99-2	Isodorsmanin A	33-E5	324.4	10(μ)M	DMSO
27-E6	BBP03016	874303-33-0	Millewanin G	33-E6	438.5	10(μ)M	DMSO
27-E7	BBP03019	1190070-91-7	Abiesinol F	33-E7	542.5	10(μ)M	DMSO
27-E8	BBP03022	122855-49-6	Panaxyne	33-E8	220.3	10(μ)M	DMSO
27-E9	BBP03024	552-66-9	Daidzin	33-E9	416.38	10(μ)M	DMSO
27-E10	BBP03025	84414-40-4	23-Hydroxybetulin	33-E10	458.7	10(μ)M	DMSO
27-E11	BBP03027	135626-13-0	Coccineone B	33-E11	298.25	10(μ)M	DMSO
27-F2	BBP03031	874303-34-1	Millewanin H	33-F2	438.5	10(μ)M	DMSO
27-F3	BBP03032	114567-34-9	Boeravinone B	33-F3	312.3	10(μ)M	DMSO
27-F4	BBP03033	82-09-7	α-Toxicarol	33-F4	410.4	10(μ)M	DMSO
27-F5	BBP03034	3044-60-8	Toxicarolisoflavone	33-F5	410.42	10(μ)M	DMSO
27-F6	BBP03035	61854-37-3	Demethoxy-7-O-	33-F6	300.27	10(μ)M	DMSO
27-F7	BBP03037	775351-91-2	Corylifol C	33-F7	338.4	10(μ)M	DMSO
27-F8	BBP03039	207792-17-4	1,7-Bis(4-hydroxyphenyl)-3-hydroxy -1,3-heptadien-5-one	33-F8	310.35	10(μ)M	DMSO
27-F9	BBP03041	6792-07-0	Macusine B	33-F9	309.4	10(μ)M	DMSO
27-F10	BBP03042	66556-91-0	ent-3β-Hydroxykaur-16-en-19-oic acid	33-F10	318.5	10(μ)M	DMSO
27-F11	BBP03043	717901-03-6	Verbenacine	33-F11	318.5	10(μ)M	DMSO
27-G2	BBP03047	23179-78-4	Songoramine	33-G2	355.5	10(μ)M	DMSO
27-G3	BBP03048	389572-70-7	Piperlotine A	33-G3	231.3	10(μ)M	DMSO
27-G4	BBP03049	34981-26-5	Kurarinone	33-G4	438.51	10(μ)M	DMSO
27-G5	BBP03054	955135-37-2	8,3'-Diprenylapigenin	33-G5	406.5	10(μ)M	DMSO

27-G6	BBP03055	161068-53-7	Epimedokoreanin B	33-G6	422.5	10(μ)M	DMSO
27-G7	BBP03057	1245-00-7	Sitsirikine	33-G7	354.5	10(μ)M	DMSO
27-G8	BBP03059	101046-79-1	Larixinol	33-G8	542.5	10(μ)M	DMSO
27-G9	BBP03060	52328-96-8	Bisdemethoxycurcumin	33-G9	308.33	10(μ)M	DMSO
27-G10	BBP03063	886989-88-4	Piperlotine C	33-G10	291.4	10(μ)M	DMSO
27-G11	BBP03064	2068-02-2	3,7-Di-O-methylquercetin	33-G11	330.3	10(μ)M	DMSO
27-H2	BBP03065	13323-48-3	Heliangin	33-H2	362.4	10(μ)M	DMSO
27-H3	BBP03066	50333-13-6	N-Methylflindersine	33-H3	241.3	10(μ)M	DMSO
27-H4	BBP03067	485-72-3	Formononetin	33-H4	268.26	10(μ)M	DMSO
27-H5	BBP03068	21293-29-8	Abscisic acid	33-H5	264.32	10(μ)M	DMSO
27-H6	BBP03070	654663-85-1	Acantrifoic acid A	33-H6	544.7	10(μ)M	DMSO
27-H7	BBP03071	20196-89-8	Kaempferol 7-O-rhamnoside	33-H7	432.4	10(μ)M	DMSO
27-H8	BBP03072	518-17-2	Evodiamine	33-H8	303.36	10(μ)M	DMSO
27-H9	BBP03073	20493-56-5	Curzerenone	33-H9	230.3	10(μ)M	DMSO
27-H10	BBP03074	99633-05-3	14-Benzoylneoline	33-H10	541.7	10(μ)M	DMSO
27-H11	BBP03075	958296-13-4	Piperlotine D	33-H11	291.4	10(μ)M	DMSO
28-A2	BBP03076	807372-38-9	4'-Demethoxypiperlotine C	34-A2	261.321	10(μ)M	DMSO
28-A3	BBP03081	55395-07-8	Baohuoside II	34-A3	500.5	10(μ)M	DMSO
28-A4	BBP03082	1207671-28-0	3,2'-Epilarixinol	34-A4	542.5	10(μ)M	DMSO
28-A5	BBP03083	332371-82-1	1,7-Bis(4-hydroxyphenyl)hepta-4,6-dien-3-one	34-A5	294.34	10(μ)M	DMSO
28-A6	BBP03084	2450-53-5	3,5-Di-O-caffeoylquinic acid	34-A6	516.45	10(μ)M	DMSO
28-A7	BBP03085	1344876-77-2	1-Cinnamoyl-3-	34-A7	217.3	10(μ)M	DMSO
28-A8	BBP03086	574-84-5	Fraxetin	34-A8	208.17	10(μ)M	DMSO
28-A9	BBP03087	135293-13-9	2''-O-Rhamnosylicariside II	34-A9	660.7	10(μ)M	DMSO
28-A10	BBP03088	187539-57-7	Ethyl rutinoside	34-A10	354.35	10(μ)M	DMSO
28-A11	BBP03090	1309920-99-7	15-Methoxymkapwanin	34-A11	360.5	10(μ)M	DMSO
28-B2	BBP03091	40918-90-9	3,4-Dimethoxycinnamyl alcohol	34-B2	194.2	10(μ)M	DMSO
28-B3	BBP03093	466-26-2	Neoline	34-B3	337.57	10(μ)M	DMSO
28-B4	BBP03094	6902-91-6	Germacrone	34-B4	218.33	10(μ)M	DMSO
28-B5	BBP03096	163815-35-8	7β-Hydroxyrutaecarpine	34-B5	303.3	10(μ)M	DMSO
28-B6	BBP03097	87064-61-7	Euphohelioscopin A	34-B6	498.66	10(μ)M	DMSO
28-B7	BBP03098	156974-99-1	Isosalicifolin	34-B7	356.4	10(μ)M	DMSO
28-B8	BBP03100	56973-51-4	Alnusdiol	34-B8	314.4	10(μ)M	DMSO
28-B9	BBP03101	549-32-6	Reynoutrin	34-B9	434.4	10(μ)M	DMSO
28-B10	BBP03102	133568-79-3	2-Ethyl-2,6,6-trimethylpiperidin-4-one	34-B10	169.3	10(μ)M	DMSO
28-B11	BBP03103	509-24-0	Songorine	34-B11	357.49	10(μ)M	DMSO
28-C2	BBP03104	5041-67-8	Juglanin	34-C2	418.4	10(μ)M	DMSO
28-C3	BBP03105	22368-21-4	Eupatilin	34-C3	344.31	10(μ)M	DMSO
28-C4	BBP03106	14103-09-4	1-Hydroxy-2,3,4,7-tetramethoxyxanthone	34-C4	332.3	10(μ)M	DMSO
28-C5	BBP03107	262272-76-4	3α-Acetoxy-20-oxo -29-norlupane-23,28-dioic acid	34-C5	530.7	10(μ)M	DMSO
28-C6	BBP03108	4931-66-2	Methyl L-pyroglutamate	34-C6	143.14	10(μ)M	DMSO
28-C7	BBP03109	21422-04-8	Demethylsuberosin	34-C7	230.3	10(μ)M	DMSO
28-C8	BBP03110	639-36-1	Akuammidine	34-C8	352.434	10(μ)M	DMSO
28-C9	BBP03111	132362-42-6	10-Hydroxyneoline	34-C9	453.6	10(μ)M	DMSO
28-C10	BBP03112	180164-14-1	Megastigm-7-ene-3,4,6,9-tetrol	34-C10	244.3	10(μ)M	DMSO
28-C11	BBP03114	116424-69-2	Fargesone A	34-C11	372.4	10(μ)M	DMSO
28-D2	BBP03116	83-46-5	β-Sitosterol	34-D2	414.69	10(μ)M	DMSO
28-D3	BBP03117	163434-73-9	2'',4''-Di-O-(E-p-coumaroyl)afzelin	34-D3	724.7	10(μ)M	DMSO
28-D4	BBP03118	74730-10-2	Pterolactone A	34-D4	260.289	10(μ)M	DMSO
28-D5	BBP03119	79120-40-4	Hannokinol	34-D5	316.4	10(μ)M	DMSO

28-D6	BBP03121	28178-92-9	Futoquinol	34-D6	354.4	10(μ)M	DMSO
28-D7	BBP03122	254886-77-6	Kushenol X	34-D7	440.5	10(μ)M	DMSO
28-D8	BBP03124	99340-07-5	Kadsurin A	34-D8	372.4	10(μ)M	DMSO
28-D9	BBP03125	870480-56-1	Bis-5,5-nortrachelogenin	34-D9	746.8	10(μ)M	DMSO
28-D10	BBP03127	868405-37-2	Protosappanin A dimethyl acetal	34-D10	318.32	10(μ)M	DMSO
28-D11	BBP03128	99217-75-1	Mulberrofuran G pentaacetate	34-D11	772.8	10(μ)M	DMSO
28-E2	BBP03129	171438-55-4	Dammarenediol II 3-O-caffeate	34-E2	606.9	10(μ)M	DMSO
28-E3	BBP03131	4046-02-0	Ethyl ferulate	34-E3	222.24	10(μ)M	DMSO
28-E4	BBP03132	90411-12-4	Neochamaejasmin B	34-E4	542.5	10(μ)M	DMSO
28-E5	BBP03133	87355-32-6	Lupeolic acid	34-E5	456.7	10(μ)M	DMSO
28-E6	BBP03134	67879-81-6	Glycosolone	34-E6	456.7	10(μ)M	DMSO
28-E7	BBP03135	288259-72-3	Denudanolide A	34-E7	356.4	10(μ)M	DMSO
28-E8	BBP03136	93859-63-3	Isochamaejasmin	34-E8	542.5	10(μ)M	DMSO
28-E9	BBP03137	2034-69-7	Daphnoretin	34-E9	352.3	10(μ)M	DMSO
28-E10	BBP03139	455255-15-9	Isovouacapenol C	34-E10	438.56	10(μ)M	DMSO
28-E11	BBP03141	6379-72-2	trans-Methylisoeugenol	34-E11	178.23	10(μ)M	DMSO
28-F2	BBP03142	32507-66-7	Isorhapontigenin	34-F2	258.3	10(μ)M	DMSO
28-F3	BBP03143	116424-70-5	Fargesone B	34-F3	372.41	10(μ)M	DMSO
28-F4	BBP03145	76376-43-7	Euphorbia factor L1	34-F4	552.7	10(μ)M	DMSO
28-F5	BBP03146	67214-05-5	2''-O-Coumaroyljuglanin	34-F5	564.5	10(μ)M	DMSO
28-F6	BBP03147	16805-10-0	Mearnsetin	34-F6	332.26	10(μ)M	DMSO
28-F7	BBP03148	120028-43-5	Edgeworin	34-F7	322.272	10(μ)M	DMSO
28-F8	BBP03150	26509-45-5	Methyleugenolglycol	34-F8	212.24	10(μ)M	DMSO
28-F9	BBP03151	57475-62-4	16-Deoxysaikogenin F	34-F9	456.711	10(μ)M	DMSO
28-F10	BBP03155	99-24-1	Methyl gallate	34-F10	184.15	10(μ)M	DMSO
28-F11	BBP03156	526-06-7	Eudesmin	34-F11	386.44	10(μ)M	DMSO
28-G2	BBP03157	76843-23-7	Przewaquinone A	34-G2	310.4	10(μ)M	DMSO
28-G3	BBP03159	112515-37-4	Neolinine	34-G3	423.54	10(μ)M	DMSO
28-G4	BBP03160	1521-41-1	3,4-Dimethoxybenzamide	34-G4	181.19	10(μ)M	DMSO
28-G5	BBP03161	152784-32-2	Puerol A	34-G5	298.3	10(μ)M	DMSO
28-G6	BBP03164	150033-85-5	Cycloshizukaol A	34-G6	548.62	10(μ)M	DMSO
28-G7	BBP03165	39945-41-0	Norglaucine hydrochloride	34-G7	377.86	10(μ)M	DMSO
28-G8	BBP03166	22804-49-5	1-Hydroxy-2,3,5-trimethoxyxanthone	34-G8	302.28	10(μ)M	DMSO
28-G9	BBP03168	78749-47-0	Shizukanolide C	34-G9	246.3	10(μ)M	DMSO
28-G10	BBP03169	327-97-9	Chlorogenic acid	34-G10	354.31	10(μ)M	DMSO
28-G11	BBP03170	151200-49-6	Borapetoside E	34-G11	536.57	10(μ)M	DMSO
28-H2	BBP03171	1269839-26-0	1-(3,4-Dihydroxyphenyl)-7-(4-hydroxyphenyl)heptane-3,5-diyl diacetate	34-H2	416.46	10(μ)M	DMSO
28-H3	BBP03172	16265-56-8	6-Deoxyjacareubin	34-H3	310.3	10(μ)M	DMSO
28-H4	BBP03173	24211-30-1	Farrerol	34-H4	300.3	10(μ)M	DMSO
28-H5	BBP03174	480-64-8	Orsellinic acid	34-H5	168.15	10(μ)M	DMSO
28-H6	BBP03175	27770-13-4	5-Hydroxy-1-methoxyxanthone	34-H6	242.23	10(μ)M	DMSO
28-H7	BBP03176	39012-04-9	Epimedoside A	34-H7	662.7	10(μ)M	DMSO
28-H8	BBP03178	305-01-1	Esculetin	34-H8	178.14	10(μ)M	DMSO
28-H9	BBP03179	151200-50-9	Borapetoside F	34-H9	534.55	10(μ)M	DMSO
28-H10	BBP03181	88668-99-9	8-Epideoxyloganic acid	34-H10	360.36	10(μ)M	DMSO
28-H11	BBP03182	885044-12-2	Farrerol 7-O-glucoside	34-H11	462.45	10(μ)M	DMSO
29-A2	BBP03184	54854-91-0	Sanshodiol	35-A2	358.39	10(μ)M	DMSO
29-A3	BBP03185	57625-31-7	Piperenone	35-A3	388.454	10(μ)M	DMSO
29-A4	BBP03186	84-99-1	Xanthoxyletin	35-A4	258.269	10(μ)M	DMSO
29-A5	BBP03187	120211-98-5	1β-Hydroxyeuscaphic acid	35-A5	504.7	10(μ)M	DMSO
29-A6	BBP03188	486-21-5	Isofraxidin	35-A6	222.19	10(μ)M	DMSO

29-A7	BBP03189	122537-59-1	Gelomulide A	35-A7	374.471	10(μ)M	DMSO
29-A8	BBP03190	62163-24-0	Mirandin B	35-A8	386.438	10(μ)M	DMSO
29-A9	BBP03191	916236-79-8	Gopherenediol	35-A9	306.483	10(μ)M	DMSO
29-A10	BBP03192	16981-20-7	Mesuol	35-A10	392.451	10(μ)M	DMSO
29-A11	BBP03193	218916-52-0	Euphorbia factor L3	35-A11	522.63	10(μ)M	DMSO
29-B2	BBP03194	67920-48-3	1,6-Dihydro-4,7'-epoxy-1-methoxy-3',4'-methylenedioxy-6-oxo-3,8'-lignan	35-B2	340.37	10(μ)M	DMSO
29-B3	BBP03195	68353-24-2	1-Methyl-2-nonylquinolin-4(1H)-one	35-B3	285.424	10(μ)M	DMSO
29-B4	BBP03197	498-02-2	Acetovanillone	35-B4	166.17	10(μ)M	DMSO
29-B5	BBP03199	1269839-24-8	5-Hydroxy-1,7-bis(4-hydroxyphenyl) heptan-3-yl acetate	35-B5	358.428	10(μ)M	DMSO
29-B6	BBP03200	1098-92-6	Kaempferol 5,7,4'-trimethyl ether	35-B6	328.316	10(μ)M	DMSO
29-B7	BBP03201	62820-11-5	Jolkinol A	35-B7	480.6	10(μ)M	DMSO
29-B8	BBP03202	118-10-5	Cinchonine	35-B8	294.39	10(μ)M	DMSO
29-B9	BBP03203	130-95-0	Quinine	35-B9	324.42	10(μ)M	DMSO
29-B10	BBP03204	122537-60-4	Gelomulide B	35-B10	388.454	10(μ)M	DMSO
29-B11	BBP03205	128988-55-6	Icariside I hydrate	35-B11	548.5	10(μ)M	DMSO
29-C2	BBP03206	438536-34-6	3(20)-Phytene-1,2-diol	35-C2	312.53	10(μ)M	DMSO
29-C3	BBP03207	464-85-7	Quinamine	35-C3	312.406	10(μ)M	DMSO
29-C4	BBP03208	59443-02-6	1-Methyl-2-undecylquinolin-4(1H)-one	35-C4	313.477	10(μ)M	DMSO
29-C5	BBP03209	15266-35-0	Dihydroevocarpine	35-C5	341.5	10(μ)M	DMSO
29-C6	BBP03210	244277-75-6	3,5,7,15-Tetraacetoxy-9-nicotinoyloxy-6(17),11-jatrophadien-14-one	35-C6	641.705	10(μ)M	DMSO
29-C7	BBP03211	104901-05-5	Borapetoside B	35-C7	552.568	10(μ)M	DMSO
29-C8	BBP03212	269739-78-8	Aglaxiflorin D	35-C8	646.727	10(μ)M	DMSO
29-C9	BBP03213	119642-82-9	Yucalexin P-17	35-C9	318.45	10(μ)M	DMSO
29-C10	BBP03214	28808-62-0	Fraxinellone	35-C10	232.28	10(μ)M	DMSO
29-C11	BBP03215	40421-52-1	erythro-1-Phenylpropane-1,2-diol	35-C11	152.19	10(μ)M	DMSO
29-D2	BBP03216	1251830-57-5	1,7-Bis(4-hydroxyphenyl)hept-6-en-3-one	35-D2	296.36	10(μ)M	DMSO
29-D3	BBP03217	174819-51-3	Rabdoketone B	35-D3	164.201	10(μ)M	DMSO
29-D4	BBP03218	111830-77-4	10-O-Methylprotosappanin B	35-D4	318.321	10(μ)M	DMSO
29-D5	BBP03219	99-20-7	Trehalose	35-D5	342.297	10(μ)M	DMSO
29-D6	BBP03220	1092103-22-4	ent-11β-Hydroxyatis-16-ene-3,14-dione	35-D6	316.435	10(μ)M	DMSO
29-D7	BBP03221	1221178-16-0	Dehydroborapetoside B	35-D7	550.552	10(μ)M	DMSO
29-D8	BBP03222	125292-97-9	Wallichinine	35-D8	370.439	10(μ)M	DMSO
29-D9	BBP03224	140669-89-2	Kadsurenin D	35-D9	356.412	10(μ)M	DMSO
29-D10	BBP03225	501-96-2	(-)-Rhododendrol	35-D10	166.2	10(μ)M	DMSO
29-D11	BBP03226	177468-85-8	Aglain C	35-D11	630.727	10(μ)M	DMSO
29-E2	BBP03228	57430-03-2	1,6-Dihydro-4,7'-epoxy-1-methoxy-3',4'-methylenedioxy-6-oxo-3,8'-lignan	35-E2	340.37	10(μ)M	DMSO
29-E3	BBP03229	112523-91-8	ent-16α,17-Dihydroxyatisan-3-	35-E3	320.466	10(μ)M	DMSO
29-E4	BBP03230	61240-34-4	Denudadione C	35-E4	340.37	10(μ)M	DMSO
29-E5	BBP03232	69573-60-0	Octadecyl caffeate	35-E5	432.636	10(μ)M	DMSO
29-E6	BBP03233	137018-33-8	3'',4''-Di-O-acetyl-2'',6''-di-O-p-coumaroylastragalin	35-E6	824.736	10(μ)M	DMSO
29-E7	BBP03235	13018-10-5	Torilin	35-E7	824.736	10(μ)M	DMSO

29-E8	BBP03236	151200-48-5	Borapetoside D	35-E8	698.709	10(μ)M	DMSO
29-E9	BBP03237	74805-92-8	Methylophiopogonanone A	35-E9	342.34	10(μ)M	DMSO
29-E10	BBP03238	120061-96-3	Shizukanolide F	35-E10	262.301	10(μ)M	DMSO
29-E11	BBP03239	74805-91-7	Methylophiopogonanone B	35-E11	328.36	10(μ)M	DMSO
29-F2	BBP03240	1228964-10-0	12-Demethylneocaesalpin F	35-F2	470.555	10(μ)M	DMSO
29-F3	BBP03241	1000995-47-0	Chloramultilide B	35-F3	734.742	10(μ)M	DMSO
29-F4	BBP03242	263249-77-0	Perilloxin	35-F4	274.312	10(μ)M	DMSO
29-F5	BBP03243	14017-71-1	(-)-Praeruptorin A	35-F5	386.395	10(μ)M	DMSO
29-F6	BBP03244	147976-35-0	Isokadsurenin D	35-F6	356.412	10(μ)M	DMSO
29-F7	BBP03245	83708-70-7	Dihydrosesamin	35-F7	356.369	10(μ)M	DMSO
29-F8	BBP03247	7047-54-3	Corymbol	35-F8	322.482	10(μ)M	DMSO
29-F9	BBP03248	80510-09-4	N-cis-Feruloyltyramine	35-F9	313.4	10(μ)M	DMSO
29-F10	BBP03250	1000995-48-1	Chloramultilide C	35-F10	734.7	10(μ)M	DMSO
29-F11	BBP03251	489-86-1	Guaiol	35-F11	222.37	10(μ)M	DMSO
29-G2	BBP03252	477336-75-7	Ophiopogonanone C	35-G2	356.33	10(μ)M	DMSO
29-G3	BBP03254	65907-75-7	Danshenxinkun A	35-G3	296.32	10(μ)M	DMSO
29-G4	BBP03256	1207185-03-2	Sarcandrolide D	35-G4	678.72	10(μ)M	DMSO
29-G5	BBP03257	18085-97-7	Jaceosidin	35-G5	330.29	10(μ)M	DMSO
29-G6	BBP03258	485-49-4	(+)-Bicuculline	35-G6	367.35	10(μ)M	DMSO
29-G7	BBP03259	86639-52-3	7-Ethyl-10-hydroxycamptothecin	35-G7	397.45	10(μ)M	DMSO
29-G8	BBP03260	349545-02-4	3'',4''-Di-O-acetyl-2'',6''-di-O-p-coumaroylastragalin	35-G8	824.74	10(μ)M	DMSO
29-G9	BBP03261	464-86-8	Conquinamine	35-G9	312.41	10(μ)M	DMSO
29-G10	BBP03263	6451-73-6	Scoulerine	35-G10	327.37	10(μ)M	DMSO
29-G11	BBP03264	27208-80-6	Polydatin	35-G11	390.4	10(μ)M	DMSO
29-H2	BBP03266	921211-29-2	ent-16-Kaurene-3β,15β,18-triol	35-H2	320.47	10(μ)M	DMSO
29-H3	BBP03267	588706-66-5	Ophiopogonanone E	35-H3	360.36	10(μ)M	DMSO
29-H4	BBP03268	115783-44-3	ent-Atisane-3β,16α,17-triol	35-H4	322.48	10(μ)M	DMSO
29-H5	BBP03269	133369-42-3	4-Hydroxy-11,12,13-trinor-5-eudesmen-7-one	35-H5	194.27	10(μ)M	DMSO
29-H6	BBP03272	24405-57-0	β-Rotunol	35-H6	234.33	10(μ)M	DMSO
29-H7	BBP03273	112500-90-0	6-Aldehydo-isoophiopogonone A	35-H7	354.31	10(μ)M	DMSO
29-H8	BBP03274	75239-63-3	Ophiopogonanone A	35-H8	328.32	10(μ)M	DMSO
29-H9	BBP03275	588706-67-6	Ophiopogonanone F	35-H9	374.38	10(μ)M	DMSO
29-H10	BBP03276	945619-74-9	Ophiopogonin D	35-H10	855.02	10(μ)M	DMSO
29-H11	BBP03277	212201-12-2	5,7-Dihydroxy-3-(4-hydroxy-3,5-dimethoxybenzyl)-6,8-dimethylchroman-4-one	35-H11	374.39	10(μ)M	DMSO
30-A2	BBP03278	79082-64-7	(-)-Corlumine	36-A2	383.39	10(μ)M	DMSO
30-A3	BBP03279	76994-07-5	Euphroside	36-A3	376.36	10(μ)M	DMSO
30-A4	BBP03280	149180-48-3	3-(2,4-Dihydroxybenzyl)-5-hydroxy -7,8-dimethoxy-6-methylchroman-4-one	36-A4	360.36	10(μ)M	DMSO
30-A5	BBP03282	24778-48-1	β-Peltoboykinolic acid	36-A5	456.71	10(μ)M	DMSO
30-A6	BBP03283	102115-79-7	Pseudoprotodioscin	36-A6	1031.18	10(μ)M	DMSO
30-A7	BBP03284	177262-32-7	Aglain B	36-A7	630.73	10(μ)M	DMSO
30-A8	BBP03285	82958-44-9	11-O-Galloylbergenin	36-A8	480.38	10(μ)M	DMSO
30-A9	BBP03288	105108-20-1	Ligucyperonol	36-A9	234.33	10(μ)M	DMSO
30-A10	BBP03289	149998-38-9	Gelomuloside A	36-A10	622.57	10(μ)M	DMSO
30-A11	BBP03290	149998-39-0	Gelomuloside B	36-A11	608.54	10(μ)M	DMSO
30-B2	BBP03291	1005212-02-1	Gelomulide N	36-B2	432.51	10(μ)M	DMSO
30-B3	BBP03292	75567-38-3	20-Deoxyingenol 3-angelate	36-B3	414.54	10(μ)M	DMSO
30-B4	BBP03293	4970-26-7	(-)-Praeruptorin B	36-B4	426.465	10(μ)M	DMSO
30-B5	BBP03294	82958-45-0	4-O-Galloylbergenin	36-B5	480.378	10(μ)M	DMSO

30-B6	BBP03296	1011762-93-8	Longipedlactone J	36-B6	536.665	10(μ)M	DMSO
30-B7	BBP03298	3520-14-7	Tetrahydropalmatine	36-B7	355.41	10(μ)M	DMSO
30-B8	BBP03299	19057-60-4	Dioscin	36-B8	869.04	10(μ)M	DMSO
30-B9	BBP03300	19083-00-2	Gracillin	36-B9	885.04	10(μ)M	DMSO
30-B10	BBP03301	666250-52-8	2',4'-Dihydroxy-3,7':4,8'-diepoxylign-7-ene	36-B10	298.338	10(μ)M	DMSO
30-B11	BBP03302	645414-25-1	Acantrifoside E	36-B11	356.37	10(μ)M	DMSO
30-C2	BBP03303	85022-66-8	Shikonofuran A	36-C2	316.35	10(μ)M	DMSO
30-C3	BBP03304	81969-41-7	Splendoside	36-C3	406.38	10(μ)M	DMSO
30-C4	BBP03305	541-15-1	L-Carnitine	36-C4	161.201	10(μ)M	DMSO
30-C5	BBP03306	70-18-8	Glutathione	36-C5	307.32	10(μ)M	DMSO
30-C6	BBP03307	53-43-0	Dehydroepiandrosterone	36-C6	288.424	10(μ)M	DMSO
30-C7	BBP03308	73-31-4	Melatonin	36-C7	232.28	10(μ)M	DMSO
30-C8	BBP03312	887147-75-3	1α-Hydroxytorilin	36-C8	392.492	10(μ)M	DMSO
30-C9	BBP03313	127179-70-8	4-O-Demethylkadsurenin D	36-C9	342.39	10(μ)M	DMSO
30-C10	BBP03314	89104-59-6	4-O-Demethylisokadsurenin D	36-C10	342.39	10(μ)M	DMSO
30-C11	BBP03315	75853-60-0	Dehydroevodiamine	36-C11	337.8	10(μ)M	DMSO
30-D2	BBP03317	113626-22-5	Metasequoic acid A	36-D2	302.458	10(μ)M	DMSO
30-D3	BBP03318	484-29-7	Dictamnine	36-D3	199.21	10(μ)M	DMSO
30-D4	BBP03319	2255-50-7	Robustine	36-D4	215.205	10(μ)M	DMSO
30-D5	BBP03320	62517-34-4	Ethyl p-hydroxyphenyllactate	36-D5	210.23	10(μ)M	DMSO
30-D6	BBP03321	19427-82-8	Selinidin	36-D6	328.36	10(μ)M	DMSO
30-D7	BBP03322	1220707-33-4	2''-O-Acetylsprengerinin C	36-D7	897.05	10(μ)M	DMSO
30-D8	BBP03323	19057-67-1	Prosapogenin A	36-D8	740.92	10(μ)M	DMSO
30-D9	BBP03324	57498-69-8	Angeflorin	36-D9	476.43	10(μ)M	DMSO
30-D10	BBP03325	88861-91-0	Sprengerinin C	36-D10	855.02	10(μ)M	DMSO
30-D11	BBP03326	88866-99-3	Sprengerinin A	36-D11	708.886	10(μ)M	DMSO
30-E2	BBP03327	13018-09-2	Torilolone	36-E2	252.35	10(μ)M	DMSO
30-E3	BBP03328	21764-41-0	Taxoquinone	36-E3	332.44	10(μ)M	DMSO
30-E4	BBP03329	19913-01-0	Futoenone	36-E4	340.37	10(μ)M	DMSO
30-E5	BBP03330	226904-40-1	4-Hydroxy-1,10-secocadin-5-ene-1,10-dione	36-E5	252.35	10(μ)M	DMSO
30-E6	BBP03331	129314-37-0	Licorisoflavan A	36-E6	438.56	10(μ)M	DMSO
30-E7	BBP03332	5088-75-5	Neoliquiritin	36-E7	418.39	10(μ)M	DMSO
30-E8	BBP03333	35738-25-1	3β-Acetoxy-11α,12α-epoxyoleanan-28,13β-olide	36-E8	512.731	10(μ)M	DMSO
30-E9	BBP03335	477-33-8	Samidin	36-E9	386.395	10(μ)M	DMSO
30-E10	BBP03336	20105-22-0	Ravenine	36-E10	243.3	10(μ)M	DMSO
30-E11	BBP03337	38695-41-9	Preskimmianine	36-E11	303.35	10(μ)M	DMSO
30-F2	BBP03338	852875-96-8	2,3-Dihydropodocarpusflavone A	36-F2	554.5	10(μ)M	DMSO
30-F3	BBP03339	51095-47-7	Methyl p-hydroxyphenyllactate	36-F3	196.2	10(μ)M	DMSO
30-F4	BBP03340	10176-71-3	Ladanein	36-F4	314.29	10(μ)M	DMSO
30-F5	BBP03341	20137-37-5	Rotundic acid	36-F5	488.7	10(μ)M	DMSO
30-F6	BBP03342	54656-47-2	1-O-Methyljatamanin D	36-F6	212.242	10(μ)M	DMSO
30-F7	BBP03343	124960-89-0	Tectoroside	36-F7	588.6	10(μ)M	DMSO
30-F8	BBP03344	64181-07-3	30-Oxolupeol	36-F8	440.7	10(μ)M	DMSO
30-F9	BBP03345	97856-19-4	8,9-Didehydro-7-hydroxydolichodial	36-F9	180.203	10(μ)M	DMSO
30-F10	BBP03346	6426-44-4	Glutinol acetate	36-F10	468.75	10(μ)M	DMSO
30-F11	BBP03347	38276-59-4	Burchellin	36-F11	340.37	10(μ)M	DMSO
30-G2	BBP03349	13323-67-6	4-O-Methylbutein	36-G2	286.28	10(μ)M	DMSO
30-G3	BBP03351	19685-09-7	10-Hydroxycamptothecin	36-G3	364.36	10(μ)M	DMSO
30-G4	BBP03352	19891-85-1	29-Nor-20-oxolupeol	36-G4	428.69	10(μ)M	DMSO
30-G5	BBP03353	30508-27-1	Licoricidin	36-G5	424.53	10(μ)M	DMSO
30-G6	BBP03354	34292-87-0	2,3-Dihydrohinokiflavone	36-G6	540.47	10(μ)M	DMSO

30-G7	BBP03355	910856-25-6	Danshenol C	36-G7	336.38	10(μ)M	DMSO
30-G8	BBP03356	93395-31-4	8β-(2-Hydroxy-2-methyl -3-oxobutyryloxy)glucozaluzanin	36-G8	538.54	10(μ)M	DMSO
30-G9	BBP03357	148-03-8	β-Tocopherol	36-G9	416.68	10(μ)M	DMSO
30-G10	BBP03358	24144-61-4	Khellactone	36-G10	262.26	10(μ)M	DMSO
30-G11	BBP03359	64190-94-9	Zanthobungeanine	36-G11	271.31	10(μ)M	DMSO
30-H2	BBP03360	121747-89-5	Isoderrone	36-H2	336.34	10(μ)M	DMSO
30-H3	BBP03361	108885-61-6	Lupeol 3-hydroxyoctadecanoate	36-H3	709.18	10(μ)M	DMSO
30-H4	BBP03362	2111-46-8	Marsformoxide B	36-H4	482.74	10(μ)M	DMSO
30-H5	BBP03363	4263-88-1	(±)-Pinoresinol	36-H5	358.39	10(μ)M	DMSO
30-H6	BBP03365	70360-12-2	Sideritoflavone	36-H6	360.31	10(μ)M	DMSO
30-H7	BBP03366	28957-04-2	Oridonin	36-H7	364.43	10(μ)M	DMSO
30-H8	BBP03367	62499-71-2	Isodihydrofutoquinol B	36-H8	356.41	10(μ)M	DMSO
30-H9	BBP03368	62560-95-6	Isodihydrofutoquinol A	36-H9	356.41	10(μ)M	DMSO
30-H10	BBP03370	60008-03-9	Glabrene	36-H10	322.35	10(μ)M	DMSO
30-H11	BBP03372	64199-78-6	Barbinervic acid	36-H11	488.7	10(μ)M	DMSO
